# Expedient Synthesis of a Library of Heparan Sulfate‐Like “Head‐to‐Tail” Linked Multimers for Structure and Activity Relationship Studies**

**DOI:** 10.1002/anie.202209730

**Published:** 2022-10-26

**Authors:** Jicheng Zhang, Li Liang, Weizhun Yang, Sherif Ramadan, Kedar Baryal, Chang‐Xin Huo, Jamie J. Bernard, Jian Liu, Linda Hsieh‐Wilson, Fuming Zhang, Robert J. Linhardt, Xuefei Huang

**Affiliations:** ^1^ Department of Chemistry Michigan State University East Lansing MI 48824 USA; ^2^ Department of Chemistry & Chemical Biology Center for Biotechnology and Interdisciplinary Studies Rensselaer Polytechnic Institute Troy NY 12180 USA; ^3^ Chemistry Department Faculty of Science Benha University Benha Qaliobiya 13518 Egypt; ^4^ Department of Pharmacology & Toxicology Michigan State University East Lansing MI 48824 USA; ^5^ Division of Chemical Biology and Medicinal Chemistry Eshelman School of Pharmacy University of North Carolina Chapel Hill NC 27599 USA; ^6^ Division of Chemistry and Chemical Engineering California Institute of Technology Pasadena CA 91125 USA; ^7^ Institute for Quantitative Health Science and Engineering Michigan State University East Lansing MI 48824 USA; ^8^ Department of Biomedical Engineering Michigan State University East Lansing MI 48824 USA

**Keywords:** Heparan Sulfate, Library Synthesis, Mimetic Design, Structure and Activity Relationship

## Abstract

Heparan sulfate (HS) plays important roles in many biological processes. The inherent complexity of naturally existing HS has severely hindered the thorough understanding of their structure‐activity relationship. To facilitate biological studies, a new strategy has been developed to synthesize a HS‐like pseudo‐hexasaccharide library, where HS disaccharides were linked in a “head‐to‐tail” fashion from the reducing end of a disaccharide module to the non‐reducing end of a neighboring module. Combinatorial syntheses of 27 HS‐like pseudo‐hexasaccharides were achieved. This new class of compounds bound with fibroblast growth factor 2 (FGF‐2) with similar structure‐activity trends as HS oligosaccharides bearing native glycosyl linkages. The ease of synthesis and the ability to mirror natural HS activity trends suggest that the new head‐to‐tail linked pseudo‐oligosaccharides could be an exciting tool to facilitate the understanding of HS biology.

## Graphical abstract

New heparan sulfate (HS) mimetics were synthesized where the disaccharide modules were connected from the reducing end to the non‐reducing end to mimic the structures of native polysaccharides more closely. A library of 27 such “head‐to‐tail” pseudo‐hexasaccharides was prepared, which was found to exhibit similar structure‐activity relationship trends as HS hexasaccharides in the binding to fibroblast growth factor 2.

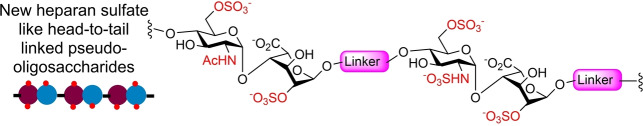

## Introduction

Heparan sulfate (HS) is a highly sulfated complex linear polysaccharide that belongs to the family of glycosaminoglycans. It is composed of disaccharide repeating units of a d‐glucosamine (GlcN) α‐(1–4)‐linked to a uronic acid (l‐iduronic acid (IdoA) or d‐glucuronic acid (GlcA)).^[1]^ In nature, multiple hydroxyl groups within the disaccharide units of HS can be *O*‐sulfated and these can include the 2‐OH of the uronic acid residue, 6‐OH and 3‐OH of GlcN, respectively. Furthermore, the GlcN unit can be *N*‐acetylated (GlcNAc) or *N*‐sulfated (GlcNS). Due to its diverse structures in nature, HS can interact with a variety of proteins including fibroblast growth factors, serine protease inhibitor antithrombin III, and amyloid β, explaining its multifaceted role in important biological events such as cell proliferation, anti‐coagulation, cancer, and Alzheimer's disease development.[^2^, ^3^]

The establishment of a detailed structure‐activity relationship (SAR) of HS has been stymied by the complexity of HS structures. It is critical that diverse structures of HS can be readily accessed to better understand the SAR of HS.[^4^, ^5^, ^6^] While significant advances in HS synthesis have been made in the past two decades, with some of the targets prepared approaching the full length of HS polysaccharides,[^7^, ^8^] the synthesis of HS oligosaccharides remains a challenging task.[^9^, ^10^, ^11^] Glycosylation reactions to form the backbone of HS can lead to low yields and low stereoselectivities with extensive efforts on synthetic method screening and optimization often needed. It is also possible that the reaction conditions/protective groups established for synthesizing shorter oligosaccharides fail in forming longer glycans.[^12^, ^13^, ^14^] In addition, with traditional synthetic designs more geared toward specific HS structures, it is tedious to generate a large number of oligosaccharides, as it is not straightforward to adjust the sulfation patterns of the oligosaccharide post glycan‐assembly.

**Figure 1:**
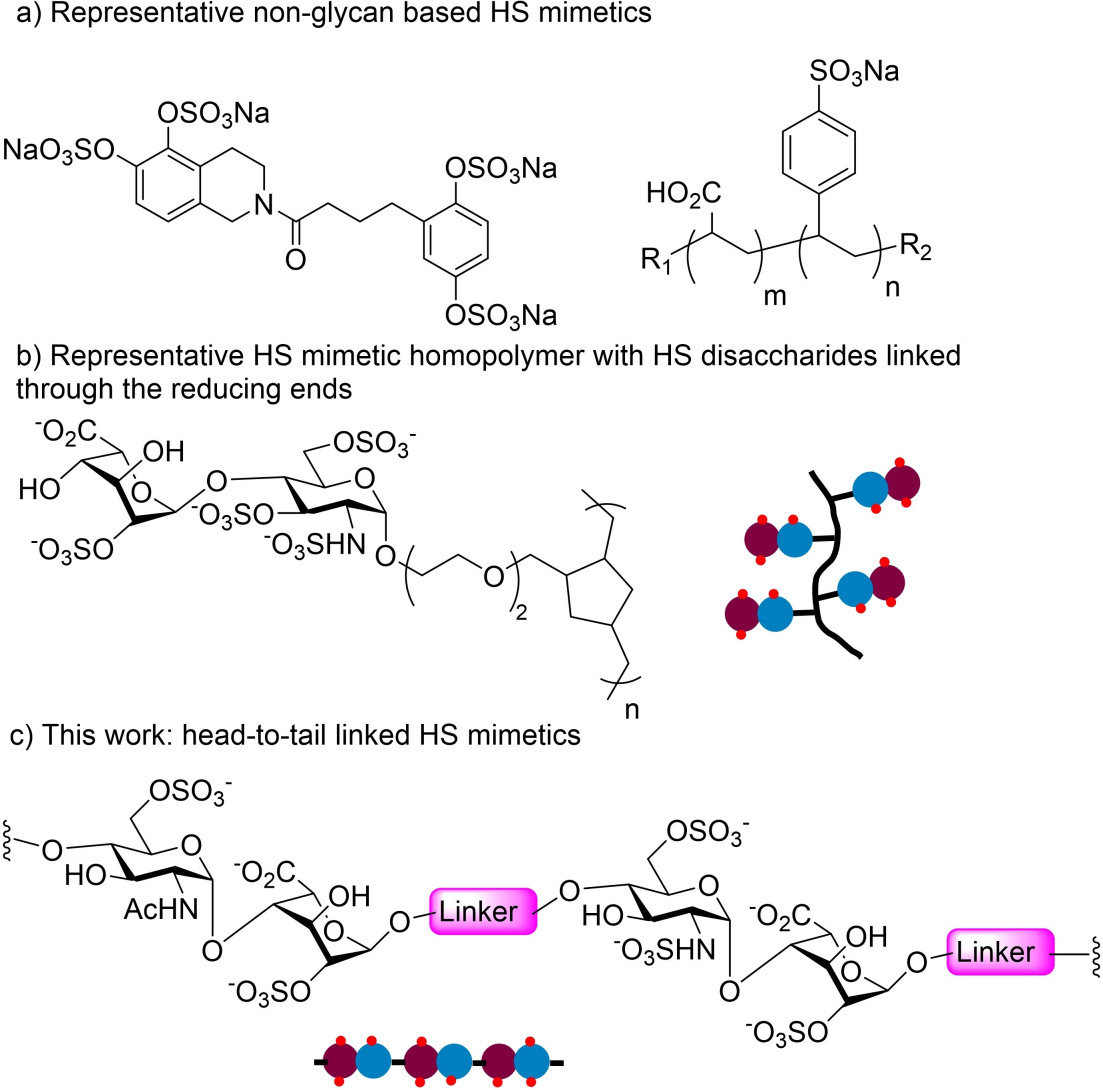
a) Representative non‐glycan‐based HS mimetics; b) A representative HS‐based branched homopolymer with disaccharide units linked through the reducing ends; c) Schematic demonstration of head‐to‐tail linked linear HS mimetics reported in this work.

One strategy to address the obstacles to accessing HS oligosaccharides is to prepare glycomimetics,[^15^, ^16^] which include non‐glycan‐based HS mimetics such as sulfated phenols and phenolic polymers (Figure 1a).[^17^, ^18^, ^19^] While they can be more easily prepared, it is challenging to closely imitate the structural subtlety of HS through this approach. As a parallel strategy, glycopolymers have been constructed with monosaccharides or disaccharides connected through the reducing ends (Figure 1b).[^20^, ^21^, ^22^, ^23^] Although these glycopolymers can be biologically active, the mono‐ and di‐saccharides are linked through the reducing end forming branches off the backbone, which are not representative of the linear structure of HS. In addition, it is difficult to precisely control the polymer sequence if more than one type of disaccharide monomer is used for polymerization to mimic the diverse sulfation patterns within a naturally occurring HS.

In this work, we report an alternative strategy for the design of HS‐like mimetics with HS disaccharides connected in a more native‐like linear “head‐to‐tail” fashion with a disaccharide module connected from its reducing end to the non‐reducing end of a neighboring unit (Figure 1c). A significant advantage of this approach is that mimetics with diverse sulfation patterns can be readily prepared with precise control of the structures. Amide chemistry was utilized to link the disaccharide modules, thus avoiding the challenging glycosylation reactions to form the hexasaccharide backbone and simplifying the overall synthetic processes. A library of 27 HS pseudo‐hexasaccharides was designed and synthesized using this method. Biological activities of these new mimetics were studied in fibroblast growth factor‐2 (FGF‐2) binding, which exhibited SAR similar to that of HS hexasaccharides with all native glycosidic linkages.

## Results and Discussion

**Scheme 1:**
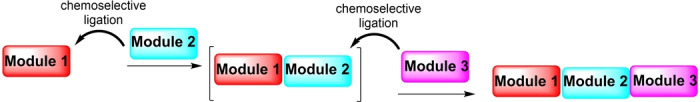
Schematic demonstration of iterative ligation to form the head‐to‐tail multimer HS mimetics.

**Figure 2:**
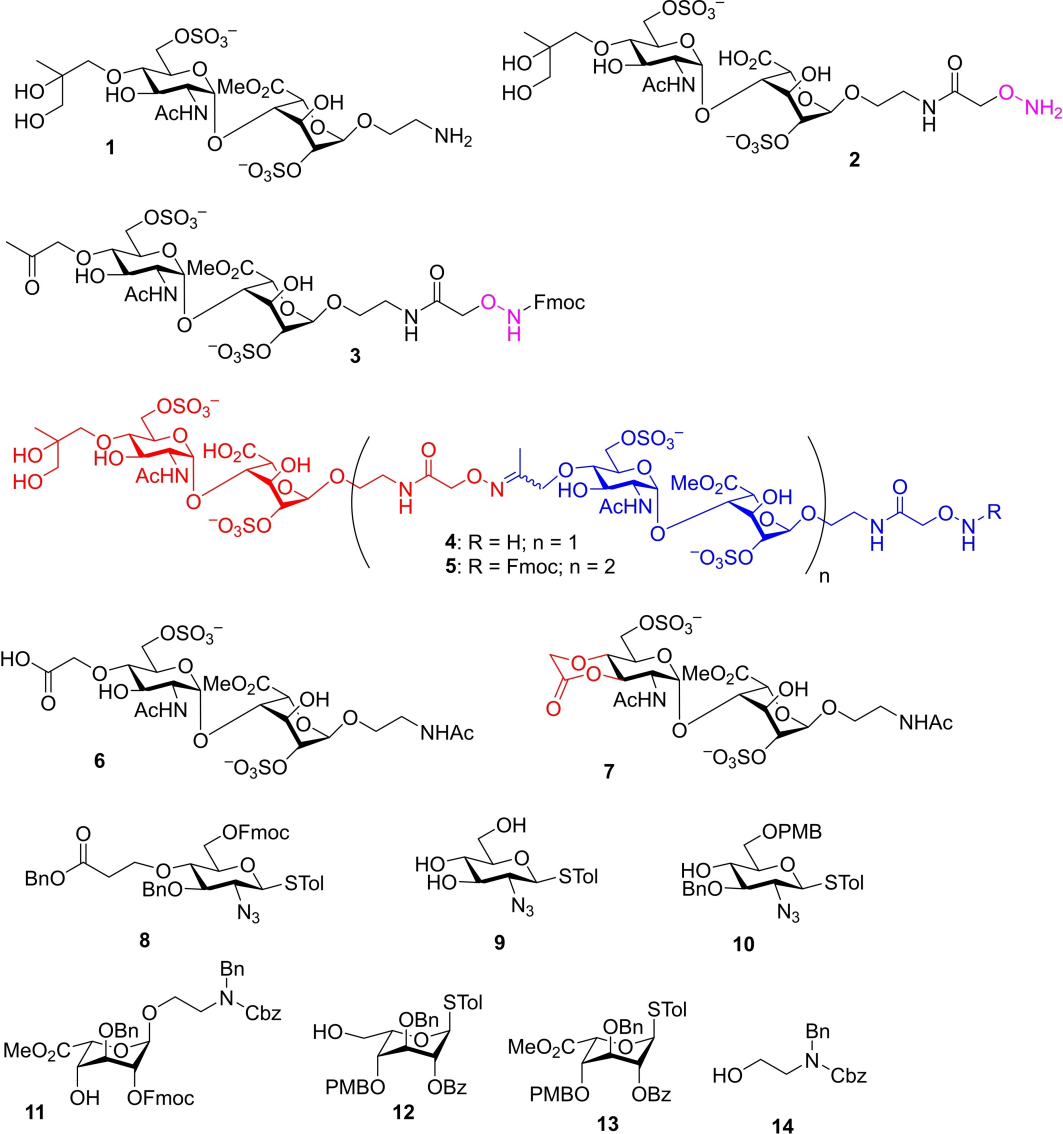
Structures of compounds **1**–**14**.

Our HS mimetics library is based on iterative ligation of disaccharide modules in a head‐to‐tail fashion (Scheme 1). To accomplish this, we first explored oxime ligation between a disaccharide bearing an alkoxyamine at the reducing end with another disaccharide having a carbonyl functionalized linker at the non‐reducing end (Section S1). Oxime formation is a powerful method for chemoselective ligation due to the high nucleophilicity of the alkoxyamine, which has been applied for the conjugation of biomolecules including carbohydrates in aqueous media.[^24^, ^25^, ^26^, ^27^] Our efforts started from disaccharide **1** (Figure 2), which was converted to alkoxyamine **2** and ketone **3** (Scheme S1). However, despite the successful formation of the pseudo‐tetrasaccharide **4** from compounds **2** and **3** (Scheme S1), further chain extension failed to lead to a high yield of the desired pseudo‐hexasaccharide **5** due to the scrambling of the oxime bond. Attempts to reduce the oxime or ligation of amine **1** and ketone **3** through the reductive amination method were not successful either.

The challenges encountered with the oxime ligation and reductive amination prompted us to shift our focus to exploring amide bonds to link the disaccharide modules. A glycolic acid moiety was installed first to the non‐reducing end of the disaccharide module (**6**). However, when **6** was subjected to amidation with amine **1**, little desired pseudo‐tetrasaccharide was obtained. Rather, the six‐membered lactone **7** was formed through intramolecular cyclization as the major product under the amide coupling condition.

We envisioned that if the carboxylic acid linker in **6** was extended by one additional methylene unit (compound **8**), it would slow down the lactonization due to the formation of the seven‐membered ring, and thus, favor the desired intermolecular amide coupling. Based on this consideration, glucosamine building block **8** was synthesized from the glucosamine thioglycoside **9** ^[28]^ through intermediate **10** (Scheme S2a). The corresponding IdoA acceptor **11** was prepared from idosyl thioglycoside **12** through conversion to donor **13** and glycosylation with acceptor **14** (Scheme S2b).

**Scheme 2:**
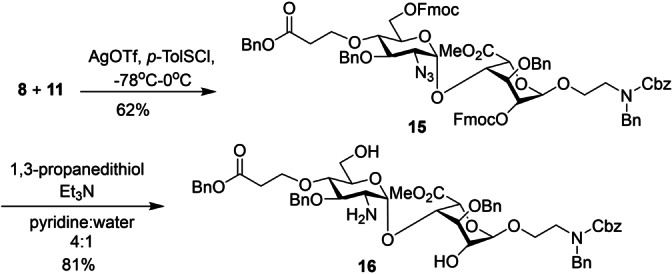
Synthesis of the key disaccharide building block **16**.

With the building blocks in hand, the glycosylation of IdoA acceptor **11** by donor **8** was carried out with the promoter of *p*‐TolSCl and AgOTf^[29]^ (Scheme 2). The presence of the non‐participating azide group at C2 of GlcN donor **8** facilitated the formation of the desired 1,2‐*cis* disaccharide **15** (^1^ *J*_C1H1_=173 Hz for the newly formed glycosidic linkage^[30]^). Treatment of **15** with 1,3‐propanedithiol in Et_3_N^[31]^ furnished **16** with azide reduction and Fmoc removal in 81 % yield.

**Scheme 3:**
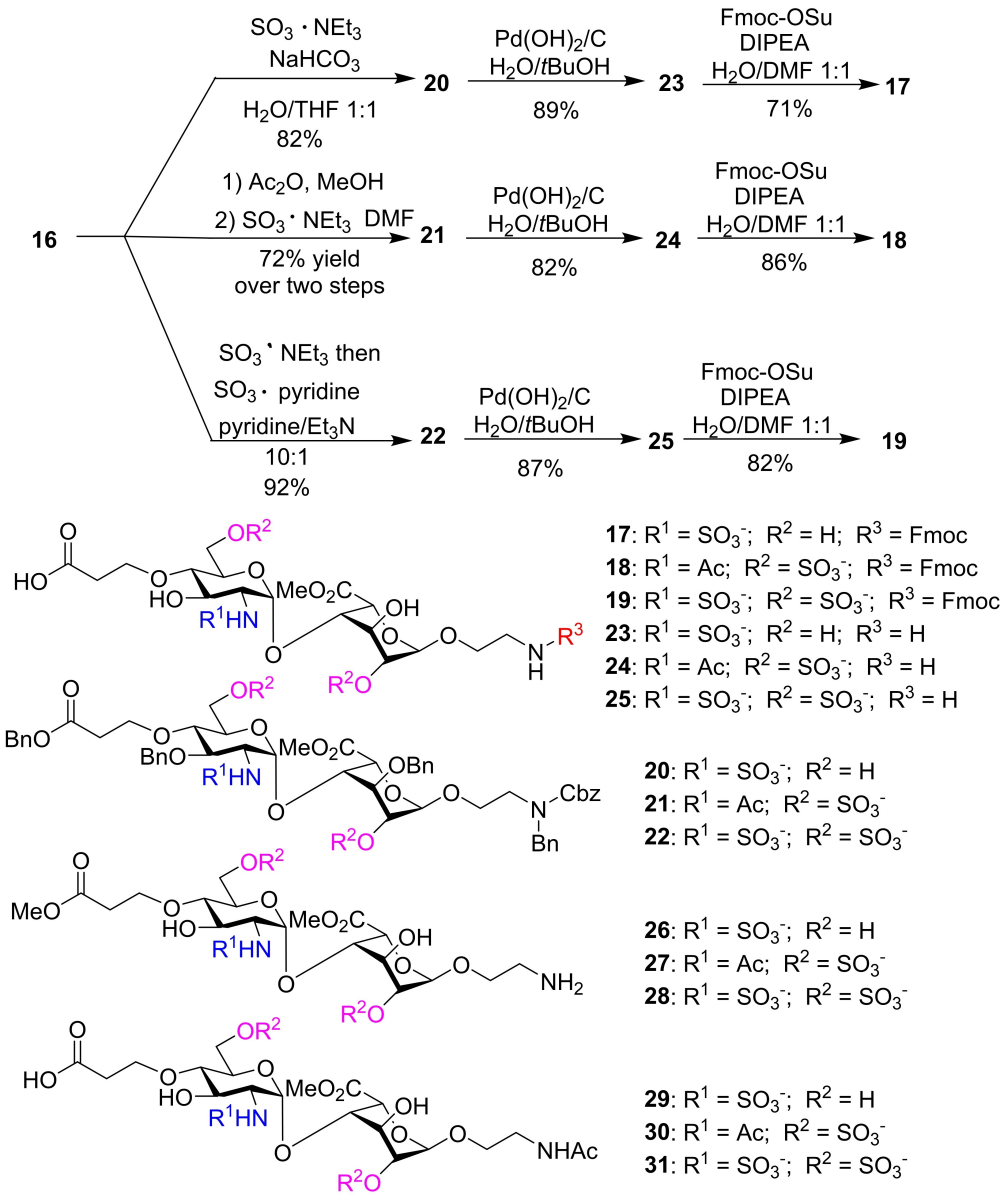
Divergent syntheses of building blocks **17**–**19** from disaccharide **16**.

With disaccharide **16** in hand, it was divergently modified to generate disaccharide modules **17**–**19** having diverse sulfation patterns (Scheme 3). *N*‐sulfation of **16** was performed with SO_3_⋅Et_3_N in a water and tetrahydrofuran (THF) cosolvent system leading to disaccharide **20**. The amine in **16** was converted to acetamide followed by *O*‐sulfation using SO_3_⋅Et_3_N at 55 °C in *N*,*N*‐dimethylformamide (DMF) to produce disaccharide **21**. In parallel, SO_3_⋅pyridine complex in pyridine was tested to sulfate all *N*‐, 6‐*O* and 2‐*O* positions of compound **16** in one pot. However, incomplete sulfation was observed even with extended reaction times and excess sulfation reagents. Two‐step sulfation was performed next with *O*‐sulfation using SO_3_⋅Et_3_N followed by *N*‐sulfation using SO_3_⋅pyridine under basic conditions to furnish **22** in 92 % yield. The benzyl (Bn) and benzyloxycarbonyl (Cbz) protective groups of **20**–**22** were removed by hydrogenolysis with Pd/C, followed by fluorenylmethyloxycarbonyl (Fmoc) protection^[32]^ of the free amine affording compounds **17**–**19** (Scheme 3). Compounds **17**–**19**, having free carboxylic acids at their non‐reducing ends and protected amines at their reducing ends, are bifunctional affording an elongation module for modular synthesis of the mimetics. The non‐reducing end modules **26**–**28** were prepared by methylating the free carboxylic acids in **17**–**19** followed by Fmoc deprotection (Scheme S3a). Disaccharides **29**–**31** were prepared by acetylating the amine moieties of **23**–**25** and were used as reducing end modules (Scheme S3b).

**Scheme 4:**
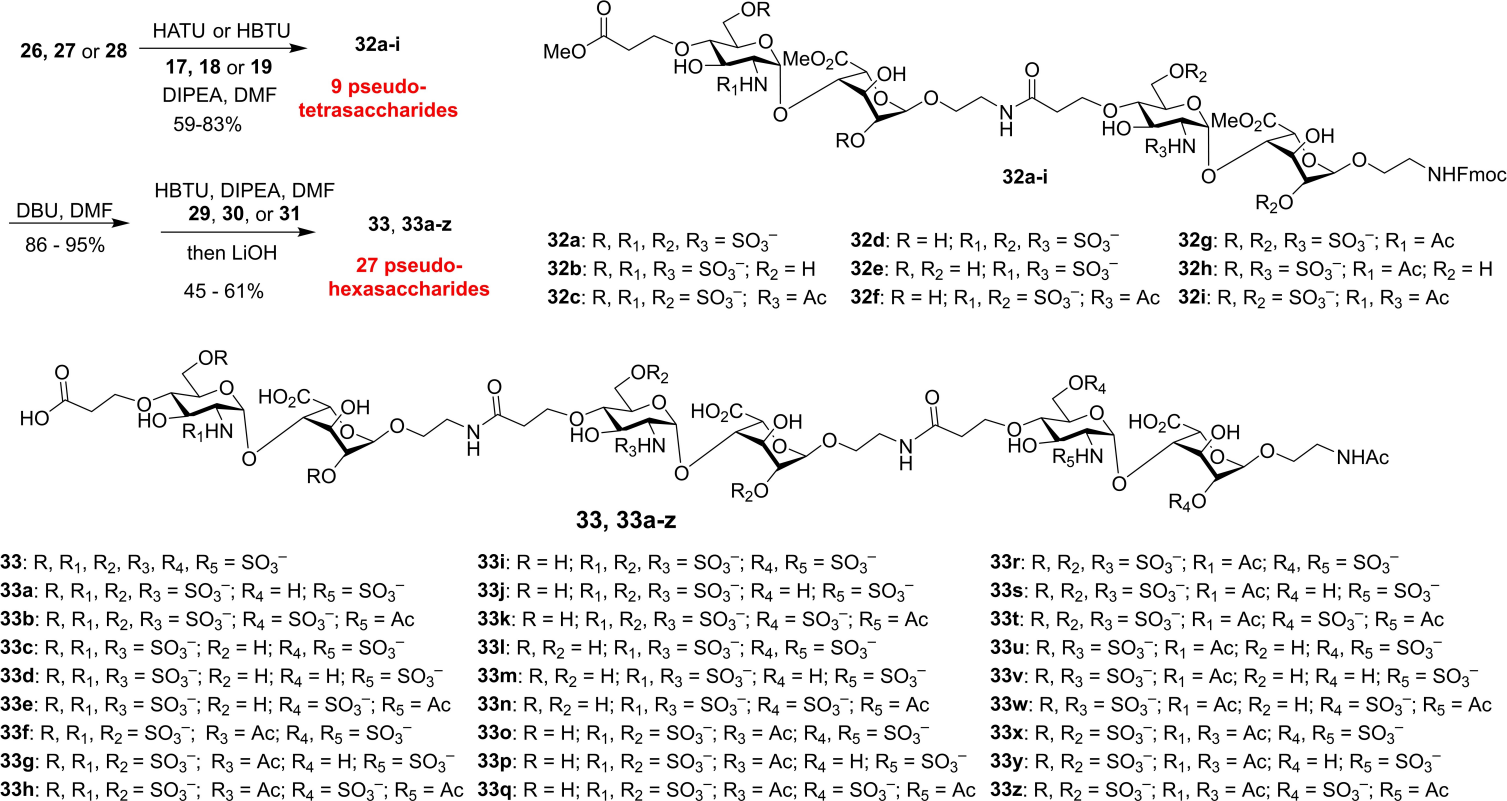
Formation of 27 HS‐like pseudo‐hexasaccharides **33**, **33** **a**–**z**.

With three sets of disaccharide modules available having different sulfation patterns, including *N*‐sulfation only, *O*‐sulfation only and *N*‐ and *O*‐ sulfation, a library of HS mimetics could be rapidly built up. Amide coupling of the non‐reducing end modules **26**–**28** with the elongation modules **17**–**19** produced 9 pseudo‐tetrasaccharides (**32** **a**–**i**) in 59–83 % yields (Scheme 4). These results suggested elongation of the carboxylic acid linker by one methylene moiety as in **17**–**19** can effectively reduce the lactone formation following the activation of the carboxylic acid. The pseudo‐tetrasaccharides (**32** **a**–**i**) were then treated with 1,8‐diazabicyclo(5.4.0)undec‐7‐ene (DBU) to deprotect the *N*‐Fmoc groups followed by a second round of amide coupling reactions with the reducing end disaccharide modules **29**–**31**, respectively. Saponification of the methyl esters afforded the fully deprotected 27 pseudo‐hexasaccharides (**33**, **33** **a**–**z**).

With the successful preparation of a library of HS‐like pseudo‐hexasaccharides, we explored whether these pseudo‐hexasaccharides could mimic natural HS glycans. We examined binding with FGF‐2, which is essential for normal physiology and cancer biology.^[33]^ FGF‐2 is one of the most extensively studied heparin‐binding proteins, which can mediate cell growth, differentiation, survival, and patterning.^[34]^

**Figure 3:**
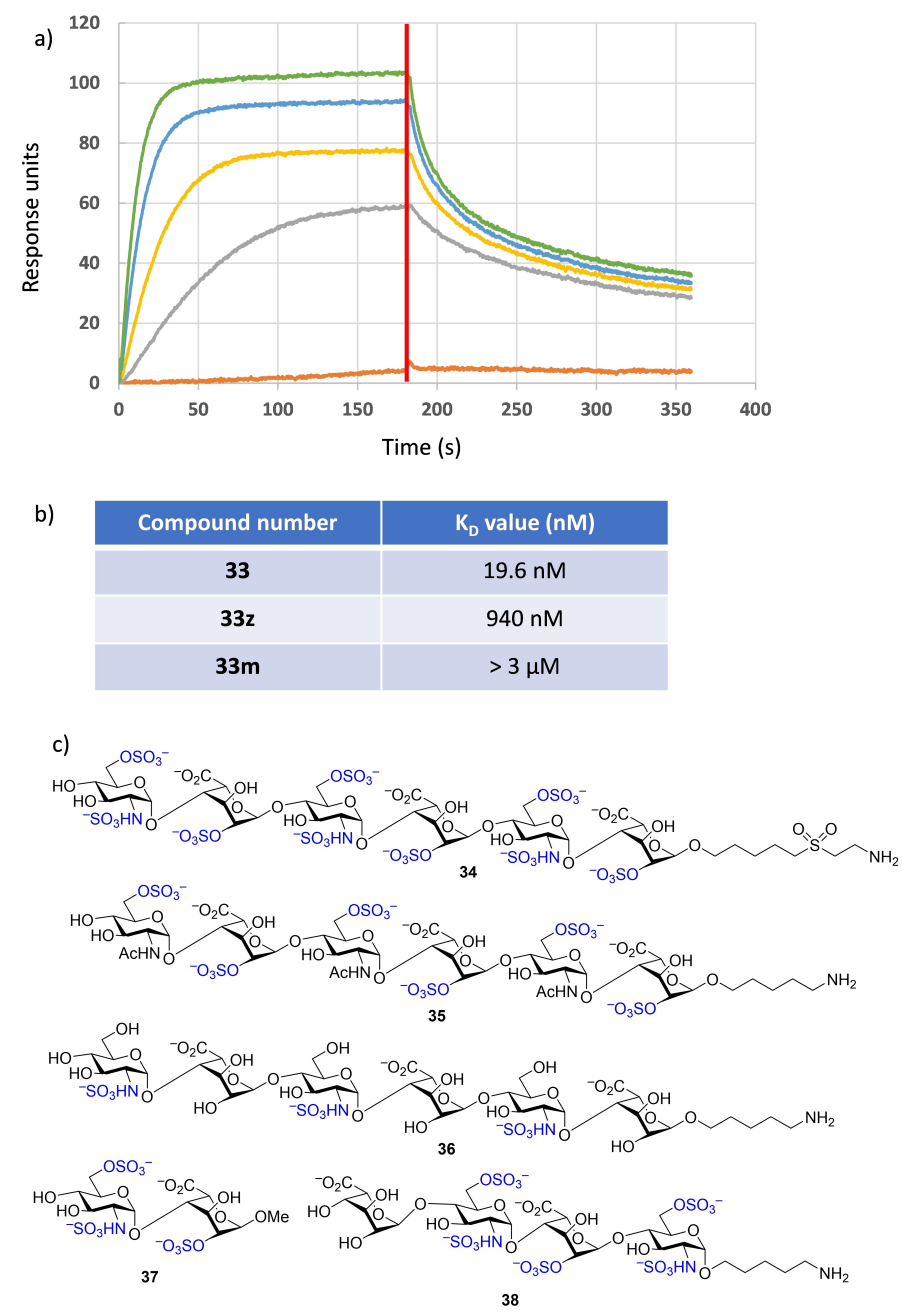
a) The SPR sensorgram of FGF‐2 binding with compound **33**. The concentrations of **33** from top to bottom curves were 750 nM, 500 nM, 250 nM, 100 nM, 10 nM, and 1 nM. Each experiment was repeated at least three times with the representative data shown. The vertical line indicates the time when the dissociation process started. b) Table of *K*_D_ values determined for **33**, **33** **z**, and **33** **m** through the FGF‐2 SPR binding assay. c) Structures of compounds **35**–**38**.

The direct binding of FGF‐2 to the HS mimetics was measured by surface plasmon resonance (SPR). FGF‐2 was immobilized on a carboxymethyl dextran high capacity (CDH) sensor chip surface through carbodiimide mediated coupling. *O*‐/*N*‐Sulfated (**33**), *O*‐sulfated (**33** **z**) and *N*‐sulfated (**33** **m**) pseudo‐hexasaccharides were selected as analytes in addition to the heparin polysaccharide (MW: 17.2 kDa). The dissociation constant (*K*_D_) of heparin binding to FGF‐2 was measured to be 6.8 nM, which is comparable with the reported value.^[35]^ The *O*‐/*N*‐ sulfated **33** exhibited strong binding to FGF‐2 with a *K*_D_ value of 19.6 nM (Figure 3). In comparison, *O*‐sulfated **33** **z** had a weaker affinity than **33** (*K*_D_=940 nM) (Figures 3b and S1a), while **33** **m** bearing only *N*‐sulfate was the weakest binder with little binding at 3 μM (Figures 3b and S1b). Seeberger and co‐workers studied the binding of HS hexasaccharides with FGF‐2 through a powerful glycan microarray technology.^[36]^ The *O*‐/*N*‐ sulfated hexasaccharide **34** exhibited ≈60 % binding to FGF‐2 at the glycan concentration of 16 μM as compared to heparin polysaccharide. The *N*‐acetylated, *O*‐sulfated hexasaccharide **35** gave binding of ≈20 % of that of heparin, while the hexasaccharide **36** having three *N*‐sulfates only gave the lowest signal (<2%) binding to FGF‐2. The trend of decreasing FGF‐2 affinity from *N*‐ and *O*‐sulfated, to *O*‐sulfated only, to *N*‐sulfated only sequences of HS hexasaccharides (**34**, **35**, **36**) correlates well with those of the pseudo‐hexasaccharides **33**, **33** **z**, and **33** **m**. In addition, the pseudo‐hexasaccharide **33** exhibits a much stronger FGF‐2 binding affinity than HS disaccharide **37** (*K*_D_=17.5 μM) and tetrasaccharide **38** (*K*_D_=1.2 μM) bearing similar sulfation patterns.[^4^, ^5^]

**Figure 4:**
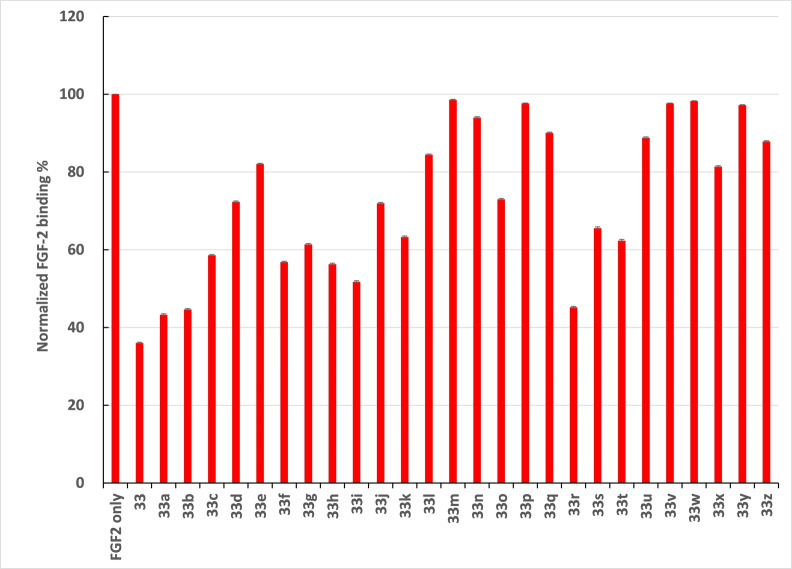
Inhibition of 27 pseudo‐hexasaccharides (**33**, **33** **a**–**z**) on FGF‐2 interaction with heparin through a competition SPR assay. Biotinylated heparin was immobilized on the SPR biosensor. For the control well, FGF‐2 (50 nM) was flown over the sensor and the intensity of the signal due to FGF‐2 binding was set as a reference (100 %). The pseudo‐hexasaccharides (10 μM) were mixed with FGF‐2 (50 nM) individually, and each solution was flown over the sensor respectively. Pseudo‐hexasaccharide capable of binding with FGF‐2 would compete with heparin for FGF‐2 and reduce the signal intensity. Normalized FGF‐2 binding % was calculated based on the following formula: (signal intensity of FGF‐2/pseudo‐hexasaccharide)/(signal intensity of FGF‐2 only)×100 %. Each binding experiment was performed three times with the standard deviations (all <0.4 %) shown.

A competition SPR assay was performed to establish the SAR between the full panel of 27 HS‐like pseudo‐hexasaccharides and FGF‐2 (Figure 4). Biotinylated heparin was immobilized on the biosensor, and the pseudo‐hexasaccharides (10 μM) were mixed with FGF‐2 (50 nM) individually. Among 27 pseudo‐hexasaccharides, the best inhibitors are **33**, **33** **a**, **33** **b**, **33** **i**, and **33** **r**, which had 50–65 % inhibition of binding between FGF‐2 and heparin at 10 μM concentrations. The IC_50_ value (the amount of the mimetic needed to reduce the FGF‐2 binding by 50 %) of **33** was determined to be 1.7 μM. **33** **z** had weaker binding with an IC_50_ value around 100 μM, while **33** **m** did not exhibit any inhibition under the experimental condition. The trend of **33**, **33** **z** and **33** **m** in the order of decreasing FGF‐2 affinity is consistent with that observed in the direct binding SPR study (Figures 3 and S1). The IC_50_ values of several disaccharides and pseudo‐tetrasaccharides with FGF‐2 have also been measured. The disaccharides bound weakly with FGF‐2, and the fully sulfated pseudo‐tetrasaccharide gave the strongest binding among the pseudo‐tetrasaccharides tested, which in turn was much weaker than that of the pseudo‐hexasaccharide **33** (Figure S2).

Based on the crystal structures of the complex formed by FGF‐2 with HS oligosaccharides,^[37]^ it is known that HS tetrasaccharides can bind with FGF‐2 with the main polar interactions (ion pair and hydrogen bonding) coming from the internal disaccharide and their 6‐*O*‐ and *N*‐sulfates interacting with the FGF‐2 surface containing residues Asn‐28, Arg‐121, Lys‐126, and Gln‐135. Longer HS oligosaccharides can extend out of this region making additional interactions with FGF‐2 and increasing the affinity. The stronger binding of **33** vs the pseudo‐tetrasaccharides, and the activity trend observed in the pseudo‐hexasaccharides mirrored that of the HS oligosaccharides with native glycosidic linkages.[^5^, ^36^, ^38^, ^39^] All the pseudo‐hexasaccharides **33**, **33** **a**, **33** **b**, **33** **i**, and **33** **r** binding FGF‐2 the strongest contain two adjacent disaccharide modules with *N*‐ and *O*‐sulfations. Neighboring disaccharide modules with *N*‐ and *O*‐sulfations in the pseudo‐hexasaccharides **33**, **33** **a**, **33** **b**, **33** **i**, and **33** **r** may mimic *N*‐ and *O*‐sulfated HS tetrasaccharides for FGF‐2 binding.

It should be pointed out that in the pseudo‐oligosaccharides prepared in this study, the disaccharides are connected through a flexible linker rather than an oxygen atom as in the native HS oligosaccharides. Thus, there should be considerable conformational flexibilities within the pseudo‐oligosaccharides, which may not adopt the same conformation as the native HS. Furthermore, the linker may gain additional interactions with the protein, or facilitate the binding with two FGF‐2 molecules, thereby increasing the avidity.^[40]^ Further studies are needed to better understand how the pseudo‐hexasaccharides interact with FGF‐2 with high affinity and the generality of the biological functions of the pseudo‐oligosaccharides.

Compared to the traditional synthetic methods, the mimetics approach simplifies the glycosylation reactions to the preparation of disaccharides. In the classical modular synthesis of HS oligosaccharides such as hexasaccharides,[^10^, ^13^, ^28^, ^41^] differentially protected advanced building blocks are prepared first, which are then sequentially glycosylated to form the hexasaccharide backbone. This is followed by selective deprotection to expose the free hydroxyl groups to be sulfated, deprotection of the amines, *O*‐sulfation, *N*‐sulfation, deacylation, and hydrogenolysis. The overall process on average takes 8–10 synthetic steps from the disaccharide building blocks. Furthermore, each hexasaccharide would individually require a similar number of synthetic operations to be carried out. Thus, it is tremendously labor intensive to prepare a HS library via the traditional approach. In comparison, with the glycomimetics approach, it takes three synthetic steps to prepare a pseudo‐hexasaccharide from the disaccharide modules, which renders a library of pseudo‐oligosaccharides much more accessible.

## Conclusion

The availability of well‐defined HS structures is critical to aid in the understanding of their important biological functions with oligosaccharides longer than disaccharides often needed to better recapitulate the biological activities of HS. Synthesis of HS is challenging and time‐consuming, with unexpected side reactions and reactivities reported for glycosylation and synthetic manipulations on longer sequences.[^12^, ^13^, ^14^] Furthermore, the preparation of a library of HS oligosaccharides is tedious requiring a large number of total synthetic steps. To overcome these limitations, glycomimetics can be an attractive strategy. We prepared a class of HS mimetics with HS disaccharide modules linked in a more native‐like “head‐to‐tail” linear configuration. Among many potential chemoselective ligation methods tested, amide chemistry was the most effective, enabling the formation of 27 pseudo‐hexasaccharide HS‐like compounds bearing precisely controlled and extensively varied sulfation patterns. The deprotection and sulfation reactions were applied on disaccharide modules instead of on longer oligosaccharides, simplifying the overall operations. Binding experiments with FGF‐2 showed the synthetic pseudo‐oligosaccharides could mirror the trends observed for native HS oligosaccharides, and compounds with adjacent *N*‐/*O*‐sulfated disaccharide modules exhibited the most potent binding to FGF‐2 (*K*_D_≈20 nM). In addition to our study, other “head‐to‐tail” HS mimetics have also been reported (Section S2).[^42^, ^43^] Thus, reducing the complexity of synthesis through the “head‐to‐tail” linear mimetics formation represents a new direction to expedite the preparation of HS mimics and facilitate the understanding of their exciting biological functions.

## Conflict of interest

J.L. is a founder and chief scientific officer for Glycan Therapeutics. He has equity in the company and serves as a paid consultant.

1

## Supporting information

As a service to our authors and readers, this journal provides supporting information supplied by the authors. Such materials are peer reviewed and may be re‐organized for online delivery, but are not copy‐edited or typeset. Technical support issues arising from supporting information (other than missing files) should be addressed to the authors.

Supporting Information

## Data Availability

The data that support the findings of this study are available in the Supporting Information of this article.
