# Supplementary material for: Expedient Synthesis of a Library of Heparan Sulfate‐Like “Head‐to‐Tail” Linked Multimers for Structure and Activity Relationship Studies
Source: Angew Chem Int Ed Engl. 2022 Oct 26;61(48):e202209730. doi: 10.1002/anie.202209730 (PMC9675719; doi:10.1002/anie.202209730)
Supplement: Supplementary file 1 — Supporting Information [file ANIE-61-0-s001.pdf]

Angewandte Chemie
Eine Zeitschrift der Gesellschaft Deutscher Chemiker

Supporting Information

Expedient Synthesis of a Library of Heparan Sulfate-Like "Head-to-Tail" Linked Multimers for Structure and Activity Relationship Studies

*J. Zhang, L. Liang, W. Yang, S. Ramadan, K. Baryal, C.-X. Huo, J. J. Bernard, J. Liu, L. Hsieh-Wilson, F. Zhang, R. J. Linhardt, X. Huang**

Table of Contents

Section S1. Exploration of oxime ligation chemistry and **Scheme S1** S3

Section S2. Comparison with other head-to-tail HS mimetics approaches S4

Materials and methods and general synthetic procedures S4

Experimental procedure for the preparation and characterization data of monosaccharides **8-13** and **Scheme S2** S7

Experimental procedure for the preparation and characterization data of disaccharides **15-19** S11

Scheme S3 S18

Experimental procedure for the preparation and characterization data of disaccharides **29-31** S18

Experimental procedure for the preparation and characterization data of pseudo-tetrasaccharides **32a**-**32i** S20

Experimental procedure for the preparation and characterization data of pseudo-hexasaccharides **33-33z** S24

Experimental procedure for SPR FGF-2 binding assays S38

Figure S1 S39

Figure S2 S40

Experimental procedure for the preparation and characterization data of **25**'-**27**', **32a**', **32b**', **32g**', and **32i**' S41

References S42

NMR spectra of compounds **8-13** S43

NMR spectra of compounds **15-31** S51

NMR spectra of compounds **32a-32i** S72

NMR spectra of compounds **33-33z** S88

Section S1. Exploration of oxime ligation chemistry to form the head-to-tail multimers

Oxime formation is a powerful method for chemoselective ligation due to the high nucleophilicity of the alkoxyamine, which has been applied for conjugation of biomolecules including carbohydrates in aqueous media.[1-4] For our mimetics approach, we first explored oxime ligation between a disaccharide bearing an alkoxyamine at the reducing end with another disaccharide having a carbonyl functionalized linker at the non-reducing end (**Scheme S1a**). Initially, we installed a protected alkoxyamine directly to the reducing end of the glycan (e.g., compound **S1**). However, in preparation of the alkoxyamine containing module, we found that the alkoxyamine moiety underwent N-O cleavage under the Pd/C catalyzed hydrogenolysis reaction in methanol, which was typically utilized to remove benzyl protective groups (**Scheme S1b**). Performing the hydrogenolysis reaction with additives such as DMSO and triethylamine,[5] a condition reported for syntheses of hydroxylamine containing compounds, also led to significant O-N bond cleavage in our study.

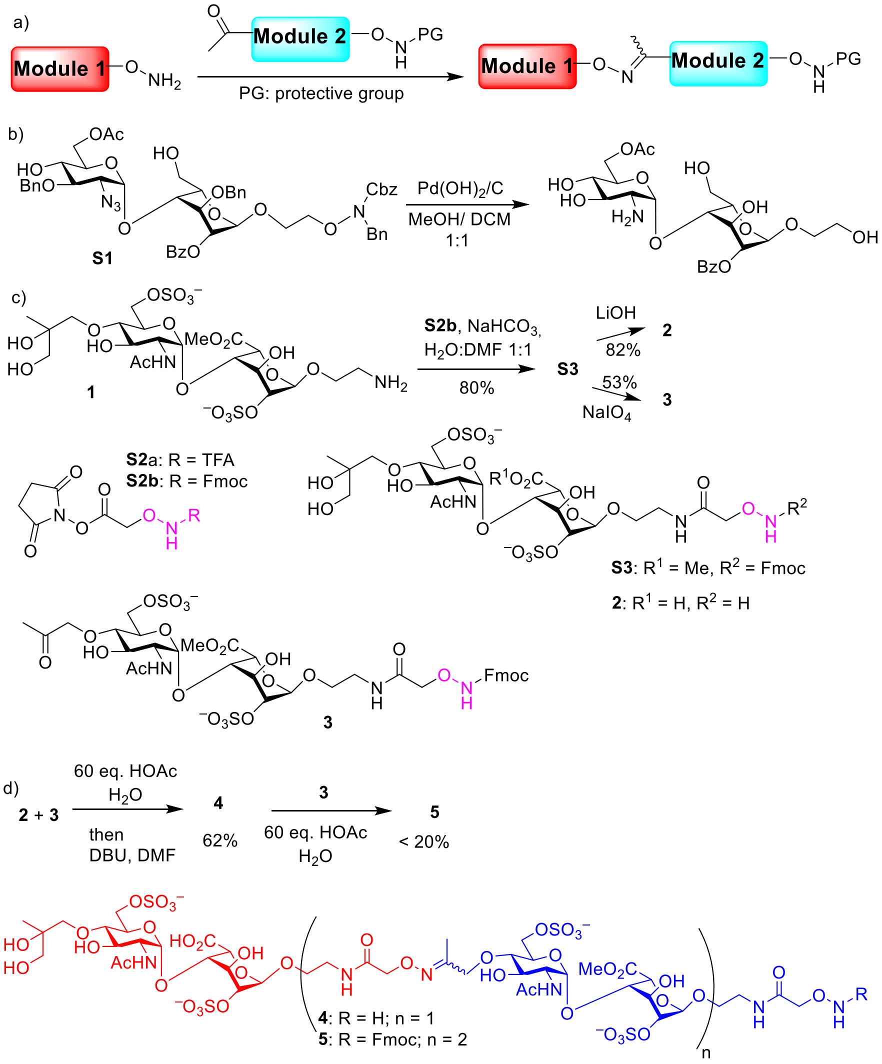

Scheme S1. Attempts to form the head-to-tail multimers through oxime bond formation.

We next installed the alkoxyamine through the amidation reaction by coupling disaccharide **1** with a free amine at the reducing end to the *N*-hydroxysuccinimide (NHS) ester of alkoxyamine **S2a** protected as a trifluoro-*N*-alkoxyacetamide (TFA) to overcome the N-O cleavage problem during hydrogenolysis. While the amidation went smoothly, surprisingly, the TFA group could not be removed in high yields through basic hydrolysis or by $NaBH_4$ reduction.[6] The major byproduct obtained was the free amine **1** as the newly formed amide was presumably more labile than the TFA amide group. We next explored the 9-fluorenylmethoxycarbonyl (Fmoc) as the protective group by conjugating Fmoc protected alkoxyamine **S2b** with disaccharide **S3** (**Scheme S1c**). The Fmoc moiety in the resulting disaccharide product **S3** was readily removed to afford the desired disaccharide **2**. The vicinal diol at the non-reducing end of disaccharide **S3** was oxidized to ketone with sodium periodate yielding ketone **3**, which was coupled with **2** followed by Fmoc deprotection to give the oxime linked pseudo-tetrasaccharide **4** (**Scheme S1d**). However, chain elongation of **4** with the disaccharide ketone **3** led to a low yield (<20%) of the desired pseudo-hexasaccharide **5**. Instead, several pseudo-tetrasaccharides were isolated, which were formed due to scrambling of the oxime bond. We attempted to reduce the oxime in the pseudo-tetrasaccharide **4** to eliminate oxime reversibility. Neither $NaBH_4$ nor $NaCNBH_3$ reduction afforded the desired reduced alkoxyamine products.

Since the oxime formation presented a significant hurdle for pseudo-hexasaccharide synthesis, we explored the alternative of ligating disaccharide amine **1** with the keto disaccharide **3** through reductive amination.[7] Unfortunately, under a variety of conditions including $NaBH_4$, $NaCNBH_3$ or $NaBH(OAc)_3$,[8] none of the desired pseudo-tetrasaccharides were obtained. These failures prompted us to utilize the amide coupling chemistry as presented in the main text.

Section S2. Comparison with other head-to-tail HS mimetics approaches.

It should be pointed out that besides our approach reported here, Revuelta et. al. synthesized sulfated monosaccharide serine conjugates forming glyco-amino acid oligomers,[9] which to the best of our knowledge was the first report of using linear oligomers to mimic HS. However, only sulfated glycosamine was utilized with no uronic acids in the structures, and homo-oligomers were prepared without varying sulfation patterns in the molecules. FGF-2 binding was studied with these mimetics by SPR through the direct binding assay with immobilized FGF-2. The binding affinity was modest with the strongest binder having a K_D value of 448 µM. In parallel to our work, Niu and coworkers recently described the synthesis of head-to-tail HS mimetics through the alkyne azide [3+2] cycloaddition reaction.[10] In the competitive SPR assay using immobilized biotinylated heparin for FGF-2 binding, the strongest mimetic showed an IC_{50} value of 6.5 µM. These reports cross-validate the utility of the linear HS mimetics for FGF-2 studies.

Materials and Methods

All reactions were performed under a nitrogen atmosphere with anhydrous solvents. Solvents were dried using a solvent purification system. Glycosylation reactions were performed with 4Å molecular sieves that were flamed dried under high vacuum. Chemicals used were reagent grade

unless noted. Reactions were visualized by UV light (254 nm) and by staining with either $Ce(NH_4)_2(NO_3)_6$ (0.5g) and $(NH_4)Mo_7O_{24}\cdot4H_2O$ (24.0 g) in 6% H_2SO_4 (500 mL), or 5% H_2SO_4 in EtOH. Flash chromatography was performed on silica gel (230-400 Mesh). NMR spectra were referenced using residual $CHCl_3$, CHD_2OD, or HDO. Peak and coupling constants assignments are based on ^{1}H-NMR, ^{1}H-^{1}H gCOSY, ^{1}H and ^{1}H-^{1}H TOCSY, ^{1}H-^{1}H NOESY, ^{1}H-^{13}C gHSQC, ^{1}H-^{13}C gHMBC. Chemical shifts values of ^{13}C-NMR reported were obtained either directly from 1D ^{13}C-NMR spectra or ^{1}H-^{13}C gHSQC.

General Synthetic Procedures

Pre-activation Based Single-step Glycosylation. A solution of the donor (1.0 equiv) and freshly activated molecular sieve MS 4 Å in dichloromethane (DCM) was stirred for 20 minutes under room temperature and then cooled to -78 °C. A solution of AgOTf (3.0 equiv) in anhydrous Et_2O/DCM (10/1, v/v) was added to the reaction solution without touching the wall of the flask. After 5 minutes, orange-colored *p*-TolSCl (1.0 equiv) was added to the solution through a microsyringe. *p*-TolSCl should be added directly to the reaction solution to prevent it from freezing on the flask wall and stir bar. The characteristic orange color of *p*-TolSCl should dissipate within a few seconds indicating the consumption of *p*-TolSCl promoter. TLC analysis could confirm the complete activation of the donor in 5 minutes. A solution of the acceptor (1.0 equiv) with tri-^{t}butyl pyrimidine (TTBP) (1.0 equiv) in DCM was added to the reaction solution (0.3 M). The reaction mixture was warmed up to 0 °C under stirring in 2 hours. The reaction mixture was quenched by Et_3N (saturated aqueous $NaHCO_3$ solution was used for Fmoc protected compounds to avoid Fmoc cleavage by Et_3N) and filtered over Celite with DCM. The DCM solution was washed with a saturated aqueous $NaHCO_3$ solution followed by washing with a saturated aqueous NaCl solution. The organic layer was collected and dried over Na_2SO_4, concentrated and purified by silica gel flash chromatography.

Deprotection of PMB. The PMB-protected compound (1.0 equiv) was dissolved in DCM/H_2O (0.1 M, 10:1, v/v). The mixture was cooled to 0 °C, followed by the addition of 2,3-dichloro-5,6-dicyano-1,4-benzoquinone (DDQ) (1.5 equiv). The mixture was stirred at room temperature for 1 hour. The residue was diluted with DCM and washed with a saturated aqueous $NaHCO_3$ solution followed by washing with a saturated aqueous NaCl solution. The organic layer was collected, dried over Na_2SO_4, and filtered. The filtrate was concentrated *in vacuo*, and the residue was purified by silica gel flash chromatography.

Protection of 6-OH with Lev. The compound containing 6-OH (1.0 equiv) was dissolved in DCM (0.3 M), followed by the addition of 1-ethyl-3-(3-dimethylaminopropyl)carbodiimide hydrochloride (EDC-HCl) (3.0 equiv), levulinic acid (1.4 equiv) and 4-dimethylamino pyridine (DMAP) (1.0 equiv). The mixture was stirred under room temperature for 1 hour. The residue was diluted with DCM and washed with an aqueous 10% HCl solution, saturated aqueous solutions of $NaHCO_3$ and NaCl. The organic layer was collected, dried over Na_2SO_4, concentrated *in vacuo*, and purified by silica gel flash chromatography.

Deprotection of Lev. The PMB-protected compound (1.0 equiv) was dissolved in a mixture of THF

and MeOH (10/1, v/v, 0.02 M), followed by the addition of hydrazine acetate (3 equiv per Lev group). Stirring was continued until TLC indicated the complete disappearance of the starting material in ~3 hours. The reaction mixture was diluted with DCM, and washed with water and brine. The organic layer was collected, dried over Na_2SO_4, concentrated *in vacuo*, and purified by silica gel flash chromatography.

***O*-Acetylation.** The compound containing -OH (1.0 equiv) was dissolved in pyridine (1.2 M), followed by the addition of acetic anhydride (7.0 equiv for each hydroxyl group) at 0 °C. The mixture was stirred at room temperature overnight and then washed with 1.0 M hydrochloric acid three times followed by saturated aqueous solutions of $NaHCO_3$ and NaCl. The organic layer was collected, dried over Na_2SO_4, filtered, concentrated *in vacuo*, and then purified by silica gel flash chromatography to afford the desired compound.

***N*-Acetylation.** The compound containing -NH_2 (1.0 equiv) was dissolved in MeOH (0.04 M), followed by the addition of acetic anhydride (3.0 equiv) and Et_3N (3.0 equiv). The mixture was stirred at room temperature for 1 h and then was passed through an LH-20 gel column with MeOH as the eluent to afford the desired compound.

Protection of OH with Fmoc. To a solution of starting material in DCM (0.3 M), FmocCl (3.0 equiv) and pyridine (4.0 equiv) were added. The reaction mixture was stirred at room temperature for 1 h and diluted with DCM. The organic mixture was washed with 1.0 M hydrochloric acid three times followed by saturated aqueous solutions of $NaHCO_3$ and NaCl. The organic layer was collected, dried over Na_2SO_4, filtered, concentrated *in vacuo*, and then purified by silica gel flash chromatography to afford the desired compound.

Protection of NH_2 with Fmoc. To a solution of starting material in H_2O/DMF (1/1, v/v, 0.3 M), Fmoc-OSu (3.0 equiv) and DIPEA (1.5 equiv) were added. The reaction mixture was stirred at room temperature for 2 h. The organic mixture was washed with 1.0 M hydrochloric acid three times followed by saturated aqueous solutions of $NaHCO_3$ and NaCl. The organic layer was collected, dried over Na_2SO_4, filtered, concentrated *in vacuo*, and then purified by silica gel flash chromatography to afford the desired compound.

For Fmoc protection of partially deprotected disaccharide, after the reaction was completed as indicated by TLC (EtOAc/MeOH/H_2O 3/1/1), the reaction mixture was passed through an LH-20 gel column with MeOH as the eluent. The compound was then passed through a column of Dowex 50WX4 Na^+ resin for ion exchange giving the product as sodium salts.

Preparation of Methyl Ester. K_2CO_3 (10.0 equiv) and CH_3I (6.0 equiv) were added to a solution of starting material (1.0 equiv) in DMF (0.13 M). The reaction mixture was stirred at room temperature for 4 h. After completion, the mixture was passed through an LH-20 gel column to afford the pure methyl ester product.

NH-Fmoc Deprotection. A solution of the starting material (1.0 equiv, 0.15 M) and 1,8-diazabicyclo[5.4.0]undec-7-ene (DBU, 5.0 equiv) in DMF was stirred for 1 h at room temperature.

After TLC (EtOAc/MeOH/H_2O, 3/1/1, v/v/v) indicating that the reaction was complete, the reaction mixture was directly loaded on an LH-20 gel column with MeOH as the eluent to give the pure compound.

Preparation of Pseudo-tetrasaccharides/hexasaccharides. A solution of carboxyethyl disaccharides (1.0 equiv), HATU or HBTU (1.0 equiv) and DIPEA (2.0 equiv) in DMF was added to starting material (1.0 equiv) in DMF (0.013 M). After the solution was stirred for 1 h at room temperature, TLC (EtOAc/MeOH/H_2O, 2.5/1/1, v/v/v) indicated completion of the reaction. The reaction mixture was passed through an LH-20 gel column with MeOH as the eluent, and the fractions containing product were collected and concentrated under a reduced pressure. The compound was then passed through a column of Dowex 50WX4 Na^+ resin for ion exchange with water as the eluent giving the product as sodium salts.

Hydrogenolysis. A solution of the compound in mixed *t*BuOH and water [1/1 (v/v), 3 mL] in the presence of 10% $Pd(OH)_2/C$ (80 mg) at room temperature was exposed to an atmosphere of H_2 using a hydrogen balloon. After overnight, the suspension was filtered through Celite, and the filtrate was concentrated *in vacuo*. The residue was passed through an LH-20 gel column using MeOH as the eluent. The product fractions were combined, concentrated under reduced pressure to give the target molecule.

Saponification of Methyl Esters. The starting material (1.0 equiv) was added to a LiOH (10.0 equiv per CO_2Me) aqueous solution (0.006 M) at 0 °C. The reaction mixture was stirred at 0 °C for 8 h. After TLC (EtOAc/MeOH/H_2O, 1.5/1/1, v/v/v) indicated the completion of the reaction, Amberlite H^+ resin was added until pH ~ 7. The reaction mixture was passed through a G-15 column, followed by a column of Dowex 50WX4 Na^+ resin for ion exchange to give the pure product as sodium salts.

Syntheses of GlcN 8 and IdoA 11 building blocks

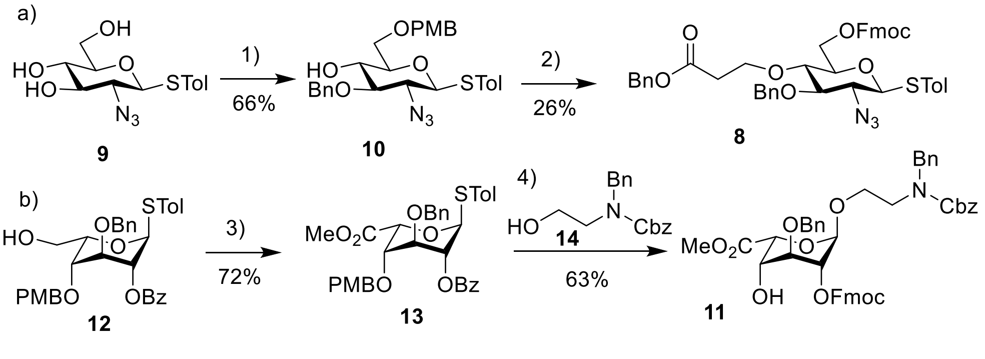

Scheme S2. Synthesis of building blocks **8** and **11**. Reagents and conditions: 1) CSA, anisaldehyde dimethyl acetal, CH_3CN; NaH, BnBr, DMF; $NaCNBH_3$, TFA, DMF. 66% yield from **9**. 2) allyl bromide, NaH; 9-BBN, 3M NaOH, H_2O_2; TEMPO, BAIB, DCM/H_2O; BnBr, DMF; DDQ, DCM/H_2O; FmocCl, pyridine, DCM. 26% from **10**. 3) TEMPO, BAIB, DCM/H_2O; 2. K_2CO_3, MeI, DMF. 72% yield from **12**. 4) AgOTf, *p*-TolSCl, -78 ^oC-0 ^oC, compound **14**; NaOMe, DCM/MeOH; FmocCl, pyridine, DCM; DDQ, DCM/H_2O. 63% yield from **13**.

In order to prepare building block **8**, thioglycosyl donor **9**[11] was protected with 4-methoxybenzylidene acetal at its 4-*O* and 6-*O* positions followed by the 3-*O* protection by the Bn group (**Scheme S2a**). $NaCNBH_3$-mediated regioselective reductive 4-methoxybenzylidene opening, allyl group installation at the 4-*O* position followed by a hydroboration-oxidation reaction to obtain the primary alcohol at the non-reducing end. The alcohol was then oxidized to a carboxylate group, which was subsequently protected as a benzyl ester. The 6-*O*-PMB protecting group was replaced with Fmoc as a potential *O*-sulfation site to form the GlcN donor **8**.

For the preparation of L-IdoA acceptor **11**, 6-OH of idoside **12** was oxidized by a catalytic amount of 2,2,6,6-tetramethyl-1-piperidinyloxy (TEMPO) with bis(acetoxy)iodobenzene (BAIB), followed by methyl ester formation to afford compound **13** (**Scheme S2b**). Glycosylation of **13** with *N*-Cbz and *N*-Bn protected ethanolamine linker **14** provided the α anomer product. The stereochemistry of the glycosidic linkage was confirmed NMR analysis ($^1J_{C1\text{-}H1}$ = 172 Hz).[12] 2-*O*-Bz was removed by NaOMe followed by 2-*O*-Fmoc protection and 4-*O*-PMB deprotection provided the L-IdoA acceptor **11**.

***p*-Tolyl 2-azido-3-*O*-benzyl-2-deoxy-6-*O*-*para*-methoxylbenzyl-1-thio-*β*-D-glucopyranoside (10)**

The thioglycosyl donor **9**[11] (3.8 g, 12.2 mmol) was dissolved in CH_3CN (0.4 M, 32.0 ml). Anisaldehyde dimethyl acetal (4.1 ml, 24.4 mmol) and camphor sulfonic acid (CSA) (1.1 g, 4.9 mmol) were added to the reaction. After stirring at room temperature overnight, the reaction mixture was neutralized with Et_3N and concentrated under a reduced pressure. The residue was diluted with DCM and washed with a saturated aqueous $NaHCO_3$ solution and brine. The organic layer was collected, dried over Na_2SO_4 and filtered. The filtrate was concentrated under reduced pressure, and the residue was purified by silica gel column chromatography using a gradient of DCM and methanol (from 20/1 to 12/1, v/v). A mixture of the above compound (4.2 g, 9.9 mmol), NaH (0.6 g, 14.8 mmol) and BnBr (2.3 ml, 19.7 mmol) in DMF (15 ml, 0.7 M) was stirred at room temperature for 2 h. After the starting material was completely consumed, the reaction mixture was neutralized with a saturated aqueous NH_4Cl solution, diluted with ethyl acetate, and sequentially washed with a saturated aqueous $NaHCO_3$ solution (3 x 70 ml) and brine (75 ml). The organic layer was collected, dried over anhydrous Na_2SO_4 and concentrated *in vacuo*. The residue was purified by flash column chromatography to afford the desired product. The product (4.9 g, 9.5 mmol) was dissolved in DMF (30 ml, 0.3 M), followed by the addition of $NaCNBH_3$ (6.0 g, 95 mmol) and TFA (7.3 ml, 95 mmol) at 0 °C. The reaction mixture was warmed to room temperature and stirred overnight, and then diluted with EtOAc. The organic mixture was washed with water (3 x 70 ml), a saturated $NaHCO_3$ aqueous solution (3 x 70 ml) and brine (50 ml). The organic layer was collected, concentrated under reduced pressure, and the residue was purified by silica gel column chromatography using a gradient of hexanes and EtOAc (from 3/1 to 2/1, v/v) to give the desired product **10** (4.2 g, 66% from **9**). ^1H NMR (500 MHz, $CDCl_3$) δ 7.51 – 7.47 (m, 2H; $ArCH_2$-), 7.43 – 7.31 (m, 5H; Ar-H), 7.29 – 7.25 (m, 2H; $ArCH_2$-), 7.11 (d, *J* = 8.0 Hz, 2H; ArCH-2-), 6.95 – 6.88 (m, 2H; $ArCH_2$-), 4.92 – 4.83 (m, 2H; $BnCH_2$), 4.57 – 4.48 (m, 2H; $PMBCH_2$), 4.39 (d, *J* = 9.0 Hz, 1H; 1-H), 3.84 (s, 3H; OCH_3), 3.78 (dd, *J* = 10.3, 4.9 Hz, 1H; 6-H), 3.73 (dd, *J* = 10.3, 4.9 Hz, 1H; 6'-H), 3.65 – 3.59 (m, 1H; 4-H), 3.47 –

3.41 (m, 1H; 5-H), 3.38 (t, *J* = 9.0 Hz, 1H; 3-H), 3.30 (t, *J* = 9.0 Hz, 1H; 2-H), 2.85 (d, *J* = 2.5 Hz, 1H; OH), 2.35 (s, 3H; $SPhCH_3$). ^{13}C NMR (125 MHz, $CDCl_3$) δ 159.37, 138.75, 137.88, 134.20, 129.81, 129.74, 129.44, 128.63, 128.27, 128.12, 127.07, 113.88, 86.14, 84.57, 77.86, 75.50, 73.44, 72.24, 70.11, 64.30, 55.32, 21.22. HRMS: m/z calc. for $C_{28}H_{31}N_3O_5S$ $[M+NH_4^+]^+$: 539.2323; found: 539.2336.

_p_-Tolyl 2-azido-3-_O_-benzyl-4-_O_-benzyl propionate-2-deoxy-6-_O_-fluorenylmethyloxycarbonyl-1-thio-_β_-D-glucopyranoside (8)

The starting material **10** (11 g, 21 mmol) was dissolved in DMF (30 ml, 0.7 M), then NaH (1.6 g, 63 mmol) and allyl bromide (3.8 ml, 42 mmol) were added at 0 °C. The reaction mixture was warmed to room temperature and stirred for 1 h until TLC (Hexanes/EtOAc 3:1, v/v) indicated the completion of the reaction. After neutralization with a saturated aqueous NH_4Cl solution, the reaction mixture was diluted with DCM, washed with a saturated aqueous $NaHCO_3$ solution (3 x 200 ml) and brine (200 ml), and dried (Na_2SO_4). The organic layer was filtered, and the filtrate was concentrated *in vacuo*, then purified by silica gel column chromatography using a gradient of hexanes and EtOAc (from 15/1 to 12/1, v/v). The above compound (11 g, 20 mmol) was dissolved in THF (87 ml, 0.2 M) and cooled to 0 °C. A solution of 9-BBN in THF (0.5 M, 77 ml, 38 mmol) was added and the reaction mixture was warmed to room temperature and stirred overnight. After hydroboration was complete, ethanol (50 ml) was added slowly to the reaction mixture at 0 °C, followed by the addition of NaOH (1.0 M aq, 98 ml) and H_2O_2 (30 wt% aq, 98 ml). The reaction mixture was warmed to 60 °C for 3 h, then quenched with a saturated aqueous NH_4Cl solution and extracted into EtOAc (3 x 100 ml). The combined organic extracts were washed with a saturated aqueous $NaHCO_3$ solution (3 x 100 ml) and brine (100 ml), dried over Na_2SO_4 and filtered. The filtrate was concentrated by rotary evaporation, and the residue was purified by silica gel column chromatography from pure hexanes to hexanes/EtOAc (10/1, v/v). The above compound (6.7 g, 11.6 mmol), BAIB (8.2 g, 25.5 mmol) and TEMPO (0.5 g, 3.5 mmol) were dissolved in DCM/H_2O (4/1, v/v, 1.2 M). After stirring at room temperature for 2 h, the reaction mixture was diluted in DCM and washed with water (3 x 75 ml) and a saturated aqueous $Na_2S_2O_3$ solution (40 ml). The organic solvent was evaporated under reduced pressure. The residue (6.8 g, 11.5 mmol) was dissolved in DMF (23 ml, 0.5 M), followed by the addition of K_2CO_3 (9.5 g, 69 mmol) and BnBr (8.2 ml, 69 mmol). The reaction mixture was stirred at room temperature for 4 h and diluted with EtOAc and washed with 1M HCl (3 x 75 ml), saturated aqueous $NaHCO_3$ solution (3 x 75 ml) and brine (75 ml). The organic solution was dried over Na_2SO_4, and the filtrate was concentrated by rotary evaporation. The residue was purified by silica gel column chromatography using a gradient of hexanes/EtOAc (15/1 to 12/1, v/v). The above compound (5.4 g, 8.0 mmol) was then treated according to the general procedures of PMB deprotection and Fmoc protection to give compound **8** (4.3 g, 26% yield from **10**). ^1H NMR (500 MHz, $CDCl_3$) δ 7.82 – 7.76 (m, 2H; $ArCH_2$-), 7.65 (ddd, *J* = 7.6, 4.5, 1.0 Hz, 2H; $ArCH_2$-), 7.52 – 7.22 (m, 16H; ArCH-), 7.12 – 7.06 (m, 2H; $ArCH_2$-), 5.14 (s, 2H; $BnCH_2$-), 4.85 – 4.76 (m, 2H; $BnCH_2$-), 4.54 – 4.41 (m, 3H; 6-H, $FmocCH_2$-), 4.36 – 4.26 (m, 3H; 1-H, 6'-H, $FmocCH_2C$*H*-), 4.14 – 4.04 (m, 1H; -OCH_2-), 3.88 – 3.81 (m, 1H; -OCH_2-), 3.47 – 3.39 (m, 2H; 4-H,

5-H), 3.34 – 3.21 (m, 2H; 2-H, 3-H), 2.64 – 2.51 (m, 2H; CO_2BnCH_2-), 2.30 (s, 3H; $SPhCH_3$). ^{13}C NMR (125 MHz, $CDCl_3$) δ 171.02, 154.96, 143.40, 143.33, 141.29, 138.88, 137.44, 135.72, 134.31, 129.79, 128.57, 128.54, 128.37, 128.31, 128.11, 127.91, 127.23, 127.21, 125.22, 120.07, 85.85, 84.74, 77.62, 76.93, 75.79, 70.04, 68.22, 66.50, 66.27, 64.70, 46.77, 35.36, 21.19. HRMS: m/z calc. for $C_{45}H_{43}N_3O_8S$ $[M+NH_4^+]^+$: 803.3109; found: 803.3099.

_p_-Tolyl methyl-2-_O_-benzoyl-3-_O_-benzyl-4-_O_-para-methoxylbenzyl-1-thio-_α_-L-idopyranosiduronate (13)

A mixture of starting material **12**[11] (9.6 g, 16.0 mmol), BAIB (11.3 g, 35.2 mmol) and TEMPO (0.74 g, 4.8 mmol) was dissolved in DCM/H_2O (4/1, 0.64 M). After stirring at room temperature for 4 h, the reaction mixture was diluted in DCM and washed with water (3 x 100 ml) and a saturated aqueous $Na_2S_2O_3$ (50 ml) solution. The organic solvent was evaporated under reduced pressure. The residue (9.8 g, 15.9 mmol) was dissolved in DMF (40 ml, 0.4 M), followed by the addition of K_2CO_3 (13.2 g, 95.4 mmol) and CH_3I (6.0 ml, 95.4 mmol). The reaction mixture was stirred at room temperature for 4 h, diluted with EtOAc and washed with 1M HCl (3 x 100 ml), a saturated aqueous $NaHCO_3$ solution (3 x 100 ml) and brine (100 ml). The organic layer was collected, dried over Na_2SO_4, filtered, and concentrated by rotary evaporation. The residue was purified by silica gel column chromatography using a solvent gradient from pure hexanes to hexanes/EtOAc (9/1 to 8/1) to yield compound **13** (7.2 g, 72% yield from **12**). ^{1}H NMR (500 MHz, $CDCl_3$) δ 7.99 (dd, J = 8.2, 1.5 Hz, 2H; $ArCH_2$-), 7.55 – 7.19 (m, 10H; ArCH-), 7.11 (d, J = 7.9 Hz, 2H; $ArCH_2$-), 7.07 – 7.00 (m, 2H; $ArCH_2$-), 6.83 – 6.66 (m, 2H; $ArCH_2$-), 5.77 – 5.65 (m, 1H; 1-H), 5.45 – 5.43 (m, 1H; 2-H), 5.34 – 5.25 (m, 1H; 5-H), 4.90 (d, J = 11.9 Hz, 1H; $BnCH_2$-), 4.66 (d, J = 11.9 Hz, 1H; $BnCH_2$-), 4.48 – 4.30 (m, 2H; $PMBCH_2$-), 3.99 – 3.92 (m, 3H; 3-H, 4-H, 5-H), 3.84 – 3.76 (m, 6H; CO_2CH_3, $PMBCH_3$), 2.32 (s, 3H; $SPhCH_3$). HRMS: m/z calc. for $C_{36}H_{36}O_8S$ $[M+NH_4^+]^+$: 646.2469; found: 646.2472.

_N_-(Benzyl)-benzyloxycarbonyl-2-aminoethyl methyl-3-_O_-benzyl-2-_O_-fluorenylmethyloxycarbonyl-4-_O_-_para_-methoxylbenzyl-_α_-L-idopyranosiduronate (11)

Compound **13** (2.4 g, 3.7 mmol) was treated according to the general procedures of pre-activation based single-step glycosylation with the commercially available acceptor **14** (1.5 equiv) to give the desired compound. The stereochemistry of the newly formed glycosidic linkage in the product was confirmed NMR analysis ($^1J_{C1\text{-}H1}$ = 172 Hz).[12] A mixture of the product (2.66 g, 3.37 mmol) and NaOMe (25% in MeOH) (0.31 ml, 1.7 mmol) in DCM/MeOH (1/2, v/v, 0.2M) was stirred at room temperature for 1 h. Then the reaction mixture was neutralized with Amberlite-H^+ resin and filtered. The crude was evaporated under reduced pressure and subjected to 2-OH Fmoc protection and 4-OH PMB deprotection according to the general procedures to give compound **11** (1.9 g, 72% yield from **13**). ^{1}H NMR (500 MHz, $CDCl_3$) δ 7.79 – 7.65 (m, 2H; $ArCH_2$-), 7.62 – 7.51 (m, 2H; $ArCH_2$-), 7.48 – 7.40 (m, 2H; $ArCH_2$-), 7.38 – 7.19 (m, 15H; ArCH-), 7.16 – 6.99 (m, 2H; $ArCH_2$-), 5.22 – 5.11 (m, 2H; 2-H, $CbzCH_2$-), 5.03 – 4.95 (m, 1H; 1-H), 4.83 – 4.76 (m, 2H; 5-H, $CbzCH_2$-), 4.75 – 4.70 (m, 1H; $OBnCH_2$-), 4.66 – 4.59 (m, 1H; $OBnCH_2$-), 4.57 – 4.37 (m, 4H; $FmocCH_2$-, $NBnCH_2$-), 4.24 (t,

J = 7.4 Hz, 1H; FmocCH$_2$C*H*-), 4.07 (br, 1H; 3-H), 3.99 – 3.87 (m, 1H; -OCH$_2$-), 3.86 – 3.81 (m, 3H), 3.74 – 3.51 (m, 2H; -CH$_2$NBnCbz, -OCH$_2$-), 3.46 – 3.35 (m, 2H; 4-H, -CH$_2$NBnCbz), 2.78 (br, 1H; -OH). ^{13}C NMR (125 MHz, CDCl$_3$) δ 169.68, 156.34, 153.75, 143.04, 141.33, 137.82, 137.23, 136.62, 129.06, 128.55, 128.53, 128.25, 128.08, 127.98, 127.87, 127.76, 127.32, 127.23, 125.33, 125.17, 125.13, 120.16, 98.49, 74.39, 72.32, 70.77, 70.60, 68.02, 67.74, 67.45, 52.46, 51.61, 46.69, 45.67. HRMS: m/z calc. for $C_{46}H_{45}NO_{11}$ $[M+NH_4^+]^+$: 805.3331; found: 805.3311.

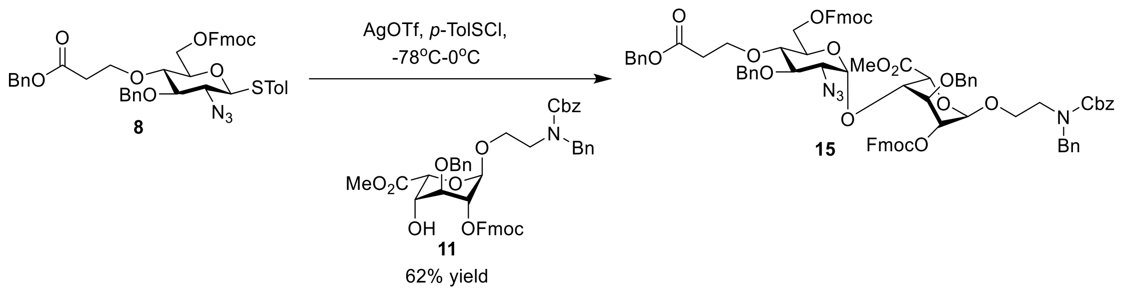

N-(Benzyl)-benzyloxycarbonyl-2-aminoethyl 2-azido-3-*O*-benzyl-4-*O*-benzylpropionate-2-deoxy-6-*O*-fluorenylmethyloxycarbonyl-*α*-D-glucopyranosyl-(1→4)-methyl-3-*O*-benzyl-2-*O*-fluorenylmethyloxycarbonyl-*α*-L-idopyranosiduronate (15).

Donor **8** (1.8 g, 2.3 mmol) and acceptor **11** (1.8 g, 2.3 mmol) were treated according to the general procedures of pre-activation based single-step glycosylation to give compound **15** (2.05 g, 62% α; 21% β). For the desired α anomer, ^{1}H NMR (500 MHz, CDCl$_3$) δ 7.81 – 7.66 (m, 5H; Ar-H), 7.66 – 7.50 (m, 5H; Ar-H), 7.46 – 7.18 (m, 27H; Ar-H), 7.18 – 7.01 (m, 4H; Ar-H), 5.24 – 5.04 (m, 5H, 1-H, 5-H, CbzCH$_2$-), 4.93 – 4.86 (m, 1H, 1'-H), 4.86 – 4.69 (m, 5H), 4.63 – 4.44 (m, 4H; 2-H), 4.43 – 4.29 (m, 4H), 4.27 – 4.01 (m, 5H), 4.01 – 3.81 (m, 4H; anomeric linker-OCH$_2$-), 3.78 – 3.71 (m, 3H; -CO$_2$CH$_3$), 3.68 – 3.59 (m, 1H), 3.57 – 3.35 (m, 4H; 2'-H, 3'-H, 4-H, 5'-H), 3.34 – 3.21 (m, 1H), 2.60 – 2.49 (m, 2H; BnO$_2$CCH$_2$-). ^{13}C NMR (125 MHz, CDCl$_3$) δ 171.00, 169.39, 156.18, 154.99, 154.58, 144.24, 143.35, 143.26, 141.27, 140.22, 137.75, 137.67, 137.34, 135.46, 131.39, 130.39, 130.20, 130.08, 128.77, 128.58, 128.47, 128.45, 128.35, 128.25, 128.00, 127.97, 127.91, 127.84, 127.80, 127.40, 127.29, 127.23, 127.19, 127.15, 125.36, 125.26, 125.15, 120.60, 120.09, 120.04, 98.50, 79.62, 76.78, 75.09, 73.89, 73.24, 70.42, 70.00, 69.61, 68.07, 67.70, 67.32, 66.44, 65.81, 63.30, 52.36, 51.59, 46.72, 46.56, 35.34. HRMS: m/z calc. for $C_{84}H_{80}N_4O_{19}$ $[M+NH_4^+]^+$: 1466.5755; found: 1466.5762.

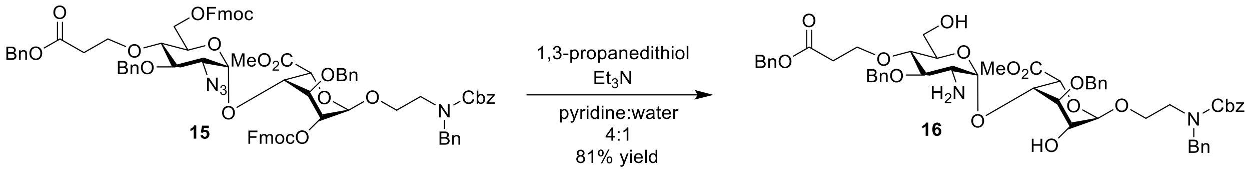

N-(Benzyl)-benzyloxycarbonyl-2-aminoethyl 2-amino-3-O-benzyl-4-O-benzylpropionate-2-deoxy-α-D-glucopyranosyl-(1→4)-methyl-3-O-benzyl-α-L-idopyranosiduronate (16).

At 50 °C under N_2 atmosphere, 1.3-propanedithiol (2.8 ml, 27.0 mmol) and Et_3N (3.7 ml, 27.0 mmol) were added to a solution of starting material **15** (0.7 g, 0.48 mmol) in pyridine/H_2O (4/1, 0.04 M) and the reaction mixture was stirred for 2 h. The reaction mixture was concentrated by rotary evaporation and purified by silica gel column chromatography using a gradient of DCM and MeOH (from 50/1 to 40/1 to 20/1, v/v) to give the desired product **16** (0.38 g, 81%). ^{1}H NMR (500 MHz, $CDCl_3$) δ 7.45 – 7.15 (m, 23H; Ar-H), 7.12 – 7.04 (m, 2H; $ArCH_2$-), 5.21 – 5.06 (m, 4H; 1-H, 1'-H), 5.00 – 4.90 (m, 2H), 4.88 – 4.80 (m, 2H; BnCH-), 4.70 (d, J = 11.9 Hz, 1H; BnCH-), 4.62 – 4.43 (m, 4H), 4.15 (s, 1H), 4.05 – 3.98 (m, 1H), 3.96 – 3.84 (m, 3H), 3.78 – 3.64 (m, 6H; -CO_2Me, -NCH_2-), 3.64 – 3.51 (m, 2H), 3.46 – 3.32 (m, 4H), 2.83 (dd, J = 10.1, 3.7 Hz, 1H), 2.63 – 2.52 (m, 2H; BnO_2CCH_2-). ^{13}C NMR (125 MHz, $CDCl_3$) δ 171.56, 138.09, 135.60, 128.60, 128.53, 128.49, 128.46, 128.42, 128.36, 128.32, 127.94, 127.82, 127.74, 127.66, 127.18, 101.88, 96.50, 82.19, 78.58, 76.77, 75.57, 72.23, 72.03, 7.87, 67.80, 67.36, 67.22, 66.54, 66.43, 61.22, 55.04, 52.37, 51.61, 46.68, 45.70, 35.41. HRMS: m/z calc. for $C_{54}H_{62}N_2O_{15}$ $[M+H^+]^+$: 978.4150; found: 978.4205.

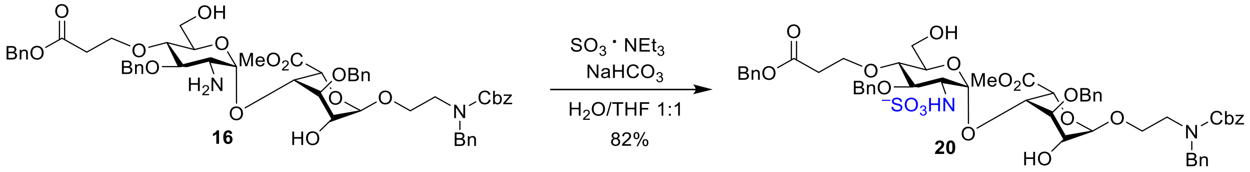

N-(Benzyl)-benzyloxycarbonyl-2-aminoethyl 3-O-benzyl-4-O-benzylpropionate-2-deoxy-2-sulfonatamido-α-D-glucopyranosyl-(1→4)-methyl-3-O-benzyl-1-thio-α-L-idopyranosiduronate (20).

The starting material **16** (90 mg, 0.09 mmol) was dissolved in mixed solvent of H_2O/THF (1/1, v/v, 0.05 M), followed by the addition of $NaHCO_3$ (115 mg, 1.37 mmol) and sulfur trioxide triethylamine complex (142 mg, 0.9 mmol). The reaction mixture was stirred at room temperature for 2 h and diluted with DCM. The organic mixture was washed with water and brine, and then filtered. The filtrate was concentrated under reduced pressure and the residue was purified by silica gel column chromatography using a mixture of DCM and methanol (20/1, v/v) to give the desired product **20**. ^{1}H NMR (500 MHz, CD_3OD) δ 7.45 (d, J = 7.4 Hz, 2H; $ArCH_2$-), 7.40 – 7.10 (m, 21H; Ar-H), 7.01-6.91 (m, 2H; $ArCH_2$-), 5.41 (d, J = 3.4 Hz, 1H; 1'-H), 5.16 – 5.06 (m, 3H; CO_2BnCH_2-, CbzCH-),

4.99 (d, J = 10.8 Hz, 1H; CbzCH-), 4.88 (s, 2H; 1-H, 5-H), 4.81 (d, J = 9.5 Hz, 1H; OBnCH-), 4.75 – 4.59 (m, 3H; $OBnCH_2$-), 4.58 – 4.33 (m, 2H; $NBnCH_2$-), 4.18 (s, 2H; 2-H, 4-H), 4.05 – 3.97 (m, 1H; 4'-linker-OCH-), 3.95 – 3.80 (m, 5H; 4'-linker-OCH-, 6'-H, $-CO_2CH_3$), 3.78 – 3.51 (m, 5H; 5'-H, 3-H, 4'-H, anomeric linker-OCH_2-), 3.49 – 3.25 (m, 5H; NCH_2-, 2'-H, 3'-H), 2.54 – 2.48 (m, 2H; $BnCO_2CH_2$-). ^{13}C NMR (125 MHz, CD_3OD) δ 171.81, 170.46, 156.65, 139.00, 138.10, 137.68, 128.24, 128.13, 128.10, 128.06, 128.01, 127.86, 127.84, 127.79, 127.77, 127.56, 127.52, 127.47, 127.38, 127.34, 127.26, 127.02, 126.90, 126.87, 101.38, 96.25, 79.92, 77.36, 74.71, 73.59, 72.15, 71.56, 67.64, 67.42, 66.47, 65.88, 60.36, 58.11, 51.55, 51.28, 46.67, 45.78, 35.15. HRMS: m/z calc. for $C_{54}H_{61}N_2O_{18}S^-$ $[M]^-$: 1057.3646; found: 1057.3646.

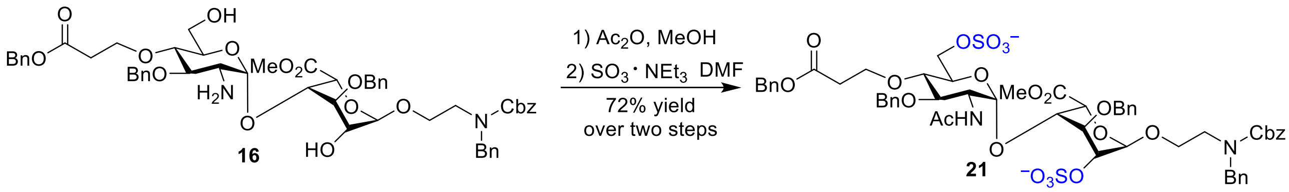

N-(Benzyl)-benzyloxycarbonyl-2-aminoethyl 2-acetamido-3-O-benzyl-4-O-benzylpropionate-2-deoxy-6-O-sulfonato-α-D-glucopyranosyl-(1→4)-methyl-3-O-benzyl-2-O-sulfonato-α-L-idopyranosiduronate (21).

The starting material **16** (225 mg, 0.22 mmol) was treated according to the general procedures of N-acetylation**.** To a solution of the product after N-acetylation in DMF (0.08 M) was added sulfur trioxide triethylamine complex (800 mg, 4.4 mmol) and the resulting reaction mixture was stirred at 55 °C for 2 h. The reaction mixture was purified by a LH-20 gel column with CH_3OH as the eluent. Fractions containing the product were collected and the solvent was removed under reduced pressure. The residue was passed through a column of Dowex 50WX4 Na^+ resin using H_2O as the eluent to give sodium form product of **21** (195 mg, 72% over two steps). ^{1}H NMR (500 MHz, CD_3OD) δ 7.60 – 7.49 (m, 1H; Ar-H), 7.41 – 7.14 (m, 21H; Ar-H), 7.12 – 6.96 (m, 2H; $ArCH_2$-), 5.22 – 5.09 (m, 4H; CO_2BnCH_2-, 1-H, 1'-H), 4.83 – 4.73 (m, 3H; 5-H, $CbzCH_2$-), 4.73 – 4.57 (m, 4H; $OBnCH_2$-, $OBnCH_2$-), 4.56 – 4.41 (m, 3H; $NBnCH_2$-), 4.29 – 4.13 (m, 3H; 2-H, 6'-H), 4.12 – 4.03 (m, 4H; 4-H, 7-H), 4.00 – 3.85 (m, 2H; NCH_2-), 3.80 – 3.74 (m, 3H, $-CO_2CH_3$), 3.70 – 3.61 (m, 3H; 5'-H, 3-H, 4'-H), 3.45 – 3.36 (m, 2H; 2'-H, 3'-H), 2.64 – 2.52 (m, 2H; $BnCO_2CH_2$-) 1.98 – 2.01 (s, 3H; $-NHCOCH_3$). HRMS: m/z calc. for $C_{56}H_{62}N_2O_{22}S_2^{2-}$ $[M]^{2-}$: 589.1623; found: 589.1611.

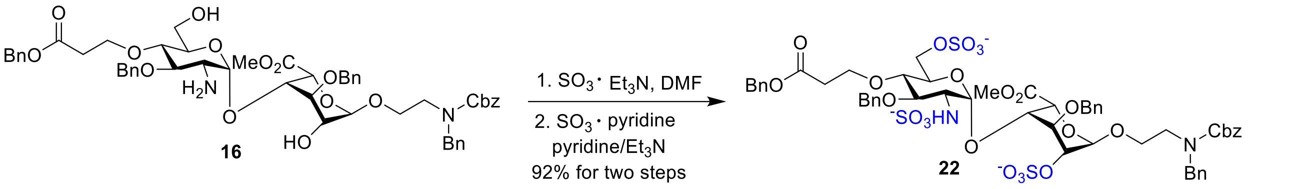

N-(Benzyl)-benzyloxycarbonyl-2-aminoethyl 3-*O*-benzyl-4-*O*-benzylpropionate-2-deoxy-2-sulfonatamido-6-*O*-sulfonato-*α*-D-glucopyranosyl-(1→4)-methyl-3-*O*-benzyl-2-*O*-sulfonato-1-thio-*α*-L-idopyranosiduronate (22).

To a solution of starting material **16** (0.3 g, 0.3 mmol) in DMF (2.0 ml, 0.15 M) was added sulfur trioxide triethylamine complex (1.12 g, 6.0 mmol) and the resulting reaction mixture was stirred at 55 °C for 2 h. The reaction mixture was purified by a LH-20 gel column with CH_3OH. Fractions containing product were collected, and the solvent was removed under reduced pressure. The crude product was dissolved in pyridine/Et_3N (10/1, v/v, 0.15M). Sulfur trioxide pyridine complex (0.45 g, 3 mmol) was added to the reaction mixture at 55 °C and stirred for 2 h. After reaction completion as indicated by TLC (EtOAc/MeOH/H_2O 6/1/1), the reaction mixture was passed through LH-20 gel column with CH_3OH as the eluent. A column of Dowex 50WX4 Na^+ resin was used for ion exchange to give sodium form of the product **22** (0.34 g, 92% for two steps). ^1H NMR (500 MHz, CD_3OD) δ 7.42 – 7.38 (d, *J* = 7.5 Hz, 2H; $ArCH_2$-), 7.41 – 7.27 (m, 11H; Ar-H), 7.25 – 7.07 (m, 10H; Ar-H), 6.98 – 6.85 (m, 2H; $ArCH_2$-), 5.40 (d, *J* = 3.4 Hz, 1H; 1'-H), 5.21 – 5.16 (m, 1H), 5.13 – 5.07 (m, 4H; 1-H), 5.05 – 5.01 (m, 1H), 4.81 – 4.71 (m, 3H; 5-H), 4.66 (d, *J* = 11.4, 1H), 4.50 – 4.37 (m, 1H; 6'-H), 4.34 – 4.19 (m, 4H; 2-H, 6'-H), 4.07 – 4.01 (m, 2H), 3.95 – 3.85 (m, 2H), 3.82 – 3.71 (m, 4H; 4'-linker-OCH_2-, -CO_2CH_3), 3.70 – 3.56 (m, 3H), 3.46 – 3.38 (m, 4H; 2'-H), 2.51 (t, *J* = 6.4 Hz, 2H). ^{13}C NMR (125 MHz, CD_3OD) δ 172.29, 170.58, 156.68, 138.91, 137.97, 137.64, 137.43, 136.15, 128.34, 128.32, 128.24, 128.18, 128.11, 128.07, 128.00, 127.97, 127.89, 127.87, 127.61, 127.54, 127.46, 127.43, 127.32, 126.97, 99.33, 98.72, 79.70, 77.42, 75.01, 73.47, 73.06, 72.41, 70.87, 70.03, 67.90, 67.25, 66.75, 66.37, 66.13, 65.90, 58.15, 58.07, 52.18, 51.45, 51.38, 46.69, 45.72, 35.26. HRMS: m/z calc. for $C_{54}H_{59}N_2O_{24}S_3^{3-}$ $[M+H^+]^{2-}$: 608.1355; found: 608.1370.

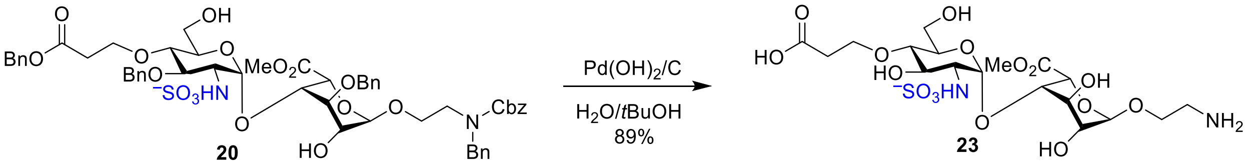

Aminoethyl 4-*O*-carboxyethyl-2-deoxy-2-sulfonatamido-*α*-D-glucopyranosyl-(1→4)-methyl-*α*-L-idopyranosiduronate (23).

Compound **20** (170 mg, 0.16 mmol) was treated according to the general procedure of

hydrogenolysis to give compound **23** (80.5 mg, 87%). ^{1}H NMR (500 MHz, CD_3OD) δ 5.36 (d, *J* = 3.7 Hz, 1H; 1'-H), 4.95 (d, *J* = 3.0 Hz, 1H; 1-H), 4.80 (d, *J* = 3.2 Hz, 1H; 5-H), 4.18 (t, *J* = 4.3 Hz, 1H; 3-H), 4.06 – 3.97 (m, 2H; 2-H, 4-H), 3.92 – 3.84 (m, 2H; 6'-CH_2-), 3.75 (s, 3H; -CO_2CH_3), 3.73 – 3.68 (m, 4H; 5'-H, 4'-linker-OCH_2-), 3.67 – 3.58 (m, 3H; 4'-linker-OCH_2-, anomeric linker-OCH_2-), 3.57 – 3.53 (m, 1H; 4'-H), 3.44 – 3.38 (m, 1H, 3'-H), 3.27 – 3.22 (m, 1H; 2'-H), 3.07 – 2.99 (m, 2H; NH_2CH_2-), 2.39 (t, *J* = 5.9 Hz, 2H; CO_2HCH_2-). ^{13}C NMR (125 MHz, CD_3OD) δ 170.39, 101.45, 96.63, 78.53, 74.10, 71.71, 69.08, 68.65, 68.01, 67.12, 64.71, 60.57, 58.06, 51.62, 46.32, 42.68, 39.26, 36.85. HRMS: m/z calc. for $C_{18}H_{31}N_2O_{16}S^-$ $[M]^-$: 563.1400; found: 563.1401.

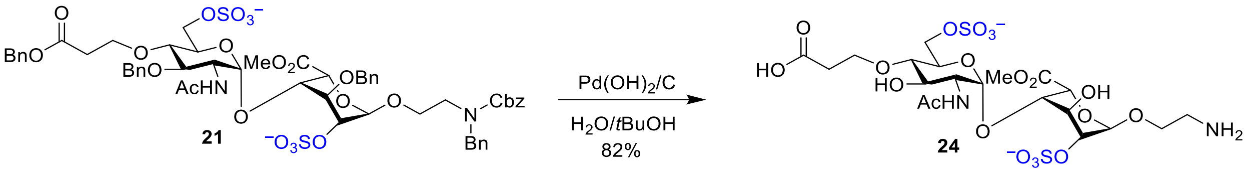

Aminoethyl 2-acetamido-4-*O*-carboxyethyl-2-deoxy-6-*O*-sulfonato-*α*-D-glucopyranosyl-(1→4)-methyl-2-*O*-sulfonato-*α*-L-idopyranosiduronate (24).

Compound **21** (290 mg, 0.25 mmol) was treated according to the general procedure of hydrogenolysis to give compound **24** (138 mg, 82%). ^{1}H NMR (500 MHz, D_2O) δ 7.72 (d, *J* = 9.6 Hz, 1H; NHAc), 5.02 (s, 1H; 1'-H), 4.95 (d, *J* = 3.4 Hz, 1H, 1-H), 4.83 (d, *J* = 1.9 Hz, 1H; 5-H), 4.20 (d, *J* = 3.0 Hz, 1H; 3-H), 4.15 – 4.06 (m, 3H; 2-H, 6'-CH_2-), 3.99 – 3.94 (m, 1H; 4-H), 3.92 – 3.82 (m, 3H; 5'-H, 4'-linker-OCH_2-), 3.80 – 3.74 (m, 1H; anomeric linker-OCH_2-), 3.66 (s, 3H; -CO_2CH_3), 3.64 – 3.59 (m, 1H; anomeric linker-OCH_2-), 3.50 – 3.41 (m, 2H; 3'-H, 4'-H), 3.25 – 3.16 (m, 1H; 2'-H), 3.25 – 3.00 (m, 2H; NH_2CH_2-), 2.47 (t, *J* = 5.9 Hz, 2H; CO_2H-$\underline{CH_2}$-), 1.88 (s, 3H; -$NHCOCH_3$). ^{13}C NMR (125 MHz, D_2O) δ 171.11, 98.96, 94.39, 77.66, 72.93, 71.10, 70.95, 69.64, 68.33, 66.58, 66.42, 64.67, 62.93, 52.96, 52.85, 38.83, 22.03. HRMS: m/z calc. for $C_{20}H_{32}N_2O_{20}S_2^{2-}$ $[M+H^+]^-$: 685.1074; found: 685.1086.

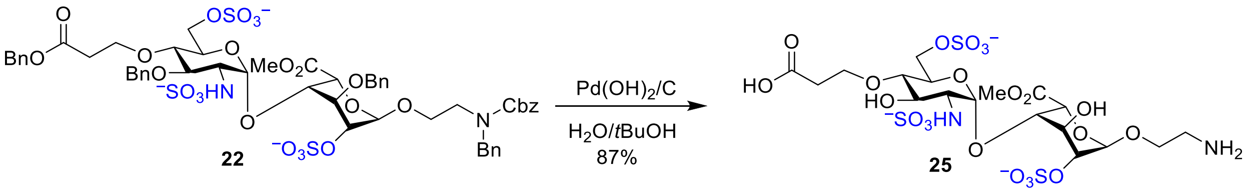

Aminoethyl 4-*O*-carboxyethyl-2-deoxy-2-sulfonatamido-6-*O*-sulfonato-*α*-D-glucopyranosyl-(1→4)-methyl-2-*O*-sulfonato-*α*-L-idopyranosiduronate (25).

Compound **22** (350 mg, 0.29 mmol) was treated according to the general procedure of hydrogenolysis to give compound **25** (180 mg, 87%). ^{1}H NMR (500 MHz, D_2O) δ 5.19 (d, *J* = 3.4 Hz, 1H, 1'-H), 5.02 (d, *J* = 2.0 Hz, 1H, 1-H), 4.77 (d, *J* = 2.1 Hz, 1H; 5-H), 4.26 (t, *J* = 4.0 Hz, 1H; 3-H), 4.19 – 4.05 (m, 3H; 2-H, 6'-CH_2-), 4.04 – 4.00 (m, 1H, 4-H), 3.90 – 3.83 (m, 2H; 5'-H, 4'-linker-OCH_2-), 3.81 – 3.75 (m, 1H; 4'-linker-OCH_2-), 3.69 (s, 3H; -CO_2CH_3), 3.65 – 3.56 (m, 2H; anomeric linker-OCH_2-), 3.48 (t, *J* = 9.8 Hz, 1H, 4'-H), 3.25 (t, *J* = 9.8 Hz, 1H, 3'-H), 3.16 – 3.05 (m, 3H; 2'-H, NH_2CH_2-), 2.43 – 2.29 (m, 2H; CO_2HCH_2-). ^{13}C NMR (125 MHz, D_2O) δ 170.99, 99.30, 98.96, 77.76, 76.90, 74.53, 70.46, 69.25, 69.07, 67.26, 67.11, 66.16, 64.60, 57.61, 53.01, 38.89. HRMS: m/z calc. for $C_{18}H_{29}N_2O_{22}S_3^{3-}$ $[M+H^+]^{2-}$: 361.0232; found: 361.0238.

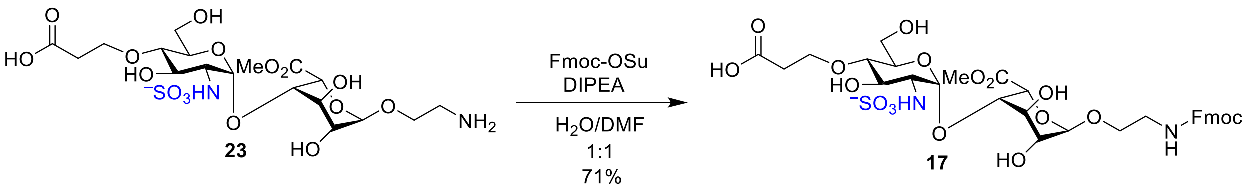

N-Fluorenylmethyloxycarbonyl-2-aminoethyl 4-*O*-carboxyethyl-2-deoxy-2-sulfonatamido-*α*-D-glucopyranosyl-(1→4)-methyl-1-thio-*α*-L-idopyranosiduronate (17).

Compound **23** (80 mg, 0.14 mmol) was treated according to the general procedure of protection of NH_2 with Fmoc to give compound **17** (79 mg, 71%). ^{1}H NMR (500 MHz, CD_3OD) δ 7.79 (d, *J* = 7.5 Hz, 2H, Ar-H), 7.64 (d, *J* = 7.5 Hz, 2H, Ar-H), 7.42 – 7.34 (m, 2H, Ar-H), 7.33 – 7.28 (m, 2H, Ar-H), 5.35 (d, *J* = 3.7 Hz, 1H; 1'-H), 4.91 – 4.89 (m, 1H; 1-H), 4.79 (d, *J* = 2.9 Hz, 1H; 5-H), 4.37 – 4.28 (m, 2H; FmocCH_2-), 4.22 – 4.13 (m, 2H; FmocCH_2C*H*-, 3-H), 4.04 – 3.95 (m, 2H; 2-H, 6'-CH_2-), 3.90 – 3.82 (m, 1H; 6'-CH_2-), 3.78 – 3.70 (m, 3H; 5'-H, 4'-linker-OCH_2-), 3.68 (s, 3H; -CO_2CH_3), 3.61 – 3.47 (m, 3H; 4'-H, anomeric linker-OCH_2-), 3.43 – 3.35 (m, 1H; 3'-H), 3.35 – 3.32 (m, 1H; -CH_2NHFmoc), 3.28 – 3.21 (m, 3H; 2'-H, -CH_2NHFmoc), 2.40 (t, *J* = 5.9 Hz, 2H; CO_2HCH_2-). ^{13}C NMR (125 MHz, CD_3OD) δ 178.87, 170.39, 157.50, 143.88, 141.15, 127.36, 126.77, 124.76, 119.48, 101.33, 96.54, 78.64, 74.02, 71.71, 71.48, 69.30, 69.10, 67.67, 67.59, 67.00, 66.37, 60.46, 57.79, 51.53, 51.51, 40.21, 37.97. HRMS: m/z calc. for $C_{33}H_{41}N_2O_{18}S^-$ $[M]^-$: 785.2081; found: 785.2103.

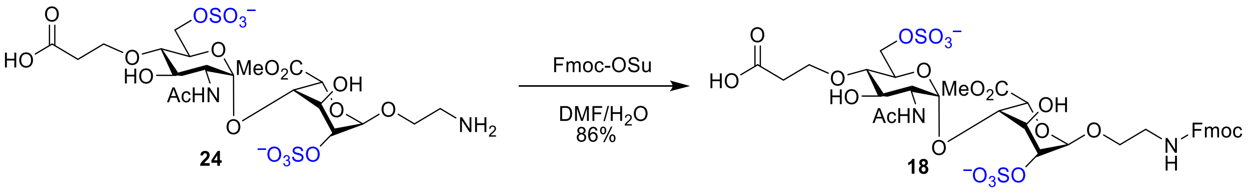

N-Fluorenylmethyloxycarbonyl-2-aminoethyl 2-acetamido-4-*O*-carboxyethyl-2-deoxy-6-*O*-

sulfonato-*α*-D-glucopyranosyl-(1→4)-methyl-2-*O*-sulfonato-1-thio-*α*-L-idopyranosiduronate (18).

Compound **24** (167 mg, 0.24 mmol) was treated according to the general procedures of protection of NH_2 with Fmoc to give compound **18** (190 mg, 86%). ^{1}H NMR (500 MHz, CD_3OD) δ 7.80 (d, *J* = 7.5 Hz, 2H; Ar-CH), 7.67 (d, *J* = 7.5 Hz, 2H; Ar-CH), 7.50 (d, *J* = 9.2 Hz, 1H; NHAc), 7.43 – 7.35 (m, 2H; Ar-CH), 7.34 – 7.29 (m, 2H; Ar-CH), 5.16 (s, 1H; 1'-H), 5.03 (d, *J* = 3.4 Hz, 1H; 1-H), 4.86 (d, *J* = 2.1 Hz, 1H; 5-H), 4.34 – 4.28 (m, 4H; 3-H, 2-H, FmocCH$_2$-), 4.25 – 4.17 (m, 3H; 6'-CH$_2$-, FmocCH$_2$C*H*-), 4.07 – 3.91 (m, 5H; 4-H, 5'-H, 4'-linker-OCH$_2$-, anomeric linker-OCH$_2$-), 3.84 – 3.75 (m, 2H; anomeric linker-OCH$_2$-, -CH$_2$NHFmoc), 3.73 (s, 3H; CO_2CH_3), 3.69 – 3.65 (m, 1H; 3'-H), 3.64 – 3.55 (m, 2H; 4'-H, -CH$_2$NHFmoc), 3.28 – 3.24 (m, 1H, 2'-H), 2.44 (dd, *J* = 6.8, 5.1 Hz, 2H; CO$_2$HCH$_2$-), 2.07 (s, 3H, NHCOCH$_3$). ^{13}C NMR (125 MHz, CD_3OD) δ 178.30, 172.95, 170.30, 157.48, 143.88, 141.14, 127.37, 126.80, 124.86, 119.46, 99.57, 95.93, 78.75, 73.28, 72.93, 71.48, 69.91, 69.14, 67.55, 66.48, 66.36, 66.03, 64.34, 52.95, 51.75, 40.18, 37.44, 21.75. HRMS: m/z calc. for $C_{35}H_{42}N_2O_{22}S_2^{2-}$ [M]$^{2-}$: 453.0841; found: 453.0840.

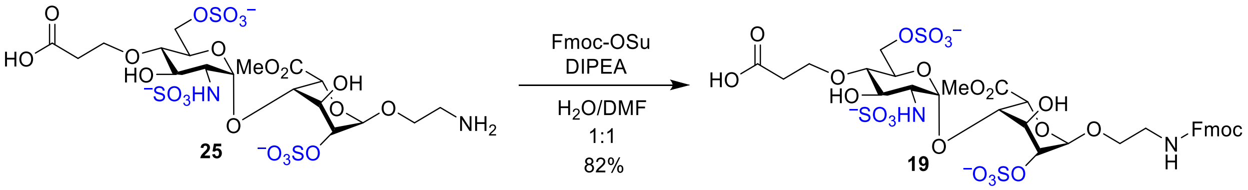

***N*-Fluorenylmethyloxycarbonyl-2-aminoethyl 2-acetamido-4-*O*-carboxyethyl-2-deoxy-6-*O*-sulfonato-*α*-D-glucopyranosyl-(1→4)-methyl-2-*O*-sulfonato-*α*-L-idopyranosiduronate (19).**

Compound **25** (220 mg, 0.31 mmol) was treated according to the general procedure of protection of NH_2 with Fmoc to give compound **19** (235 mg, 82%). ^{1}H NMR (500 MHz, CD_3OD) δ 7.80 (d, *J* = 7.8 Hz, 2H; ArCH-), 7.67 (d, *J* = 7.5 Hz, 2H; ArCH-), 7.40 (dt, *J* = 7.8, 2.6 Hz, 2H; ArCH-), 7.33 (td, *J* = 7.5 Hz, 2.6 Hz, 2H; ArCH-), 5.26 (d, *J* = 3.4 Hz, 1H, 1'-H), 5.21 (s, 1H, 1-H), 4.84 (d, *J* = 2.2 Hz, 1H; 5-H), 4.43 – 4.39 (m, 1H; 3-H), 4.34 – 4.28 (m, 3H; 2-H, FmocCH$_2$-), 4.25 – 4.17 (m, 3H; 6'-CH$_2$-, FmocCH$_2$C*H*-), 4.10 – 4.04 (m, 1H, 4-H), 4.01 – 3.94 (m, 2H, 5'-H, 4'-linker-OCH$_2$-), 3.82 – 3.76 (m, 1H, 4'-linker-OCH$_2$-), 3.74 (s, 3H, -CO$_2$CH$_3$), 3.68 – 3.61 (m, 2H; anomeric linker-OCH$_2$-), 3.60 – 3.53 (m, 1H; 4'-H), 3.37 – 3.34 (m, 3H; -CH$_2$NHFmoc), 3.30 – 3.25 (m, 1H; 2'-H), 2.61 – 2.50 (m, 2H; CO$_2$H$\underline{CH_2}$-). ^{13}C NMR (125 MHz, CD_3OD) δ 170.51, 143.88, 141.13, 127.39, 126.80, 124.88, 124.85, 119.48, 99.31, 98.68, 78.87, 75.80, 73.53, 71.03, 69.75, 67.70, 66.60, 66.54, 66.14, 65.95, 57.62, 51.88, 40.12, 37.32. HRMS: m/z calc. for $C_{33}H_{39}N_2O_{24}S_3^{3-}$ [M]$^{3-}$: 314.3690; found: 314.3696.

Scheme S3. Synthesis of a) the non-reducing end modules **26-28**; and b) the reducing end modules **29-31**.

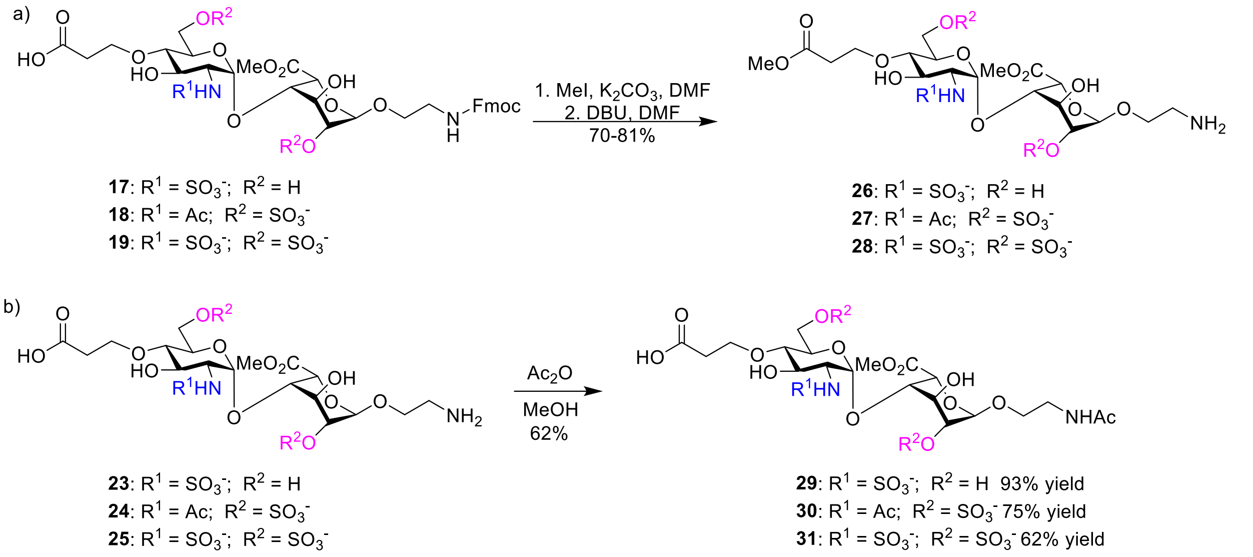

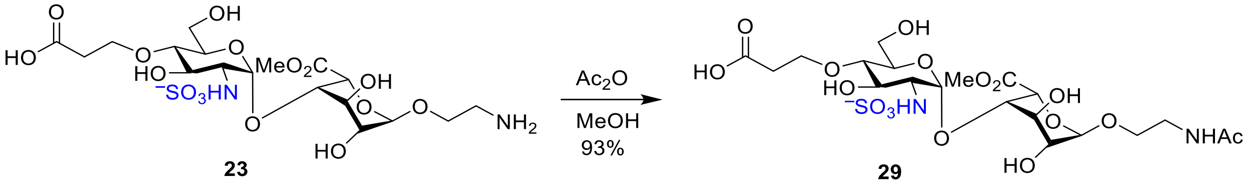

Acetamidoethyl 4-*O*-carboxyethyl-2-deoxy-2-sulfonatamido-*α*-D-glucopyranosyl-(1→4)-methyl-*α*-L-idopyranosiduronate (29).

Compound **23** (40 mg, 0.07 mmol) was treated according to the general procedure of *N*-acetylation to give compound **29** (40 mg, 93%). ^{1}H NMR (500 MHz, CD_3OD) δ 5.36 (d, J = 3.6 Hz, 1H, 1'-H), 4.89 (s, 1H, 1-H), 4.80 (d, J = 3.0 Hz, 1H; 5-H), 4.16 (t, J = 4.3 Hz, 1H; 3-H), 4.08 – 3.97 (m, 2H; 2-H, 4-H), 3.93 – 3.85 (m, 1H; 6'-CH_2-), 3.80 – 3.69 (m, 6H; -CO_2CH_3, 5'-H, 6'-CH_2-, 4'-linker-OCH_2-), 3.62 – 3.50 (m, 3H; 4'-linker-OCH_2-, 4'-H, anomeric linker-OCH_2-), 3.45 – 3.34 (m, 3H; anomeric linker-OCH_2-, 3'-H, -CH_2NHAc), 3.30 – 3.22 (m, 2H; 2'-H, -CH_2NHAc), 2.49 – 2.42 (m, 2H; CO_2HCH_2-), 1.94 (s, 3H; -$NHCOCH_3$). ^{13}C NMR (125 MHz, CD_3OD) δ 177.09, 171.96, 170.45, 101.41, 96.58, 78.48, 74.12, 71.67, 71.62, 69.30, 68.55, 67.89, 67.27, 67.22, 60.53, 57.92, 51.55, 38.94, 36.65, 21.13. HRMS: m/z calc. for $C_{20}H_{33}N_2O_{17}S^-$ [M]$^-$: 605.1505; found: 605.1513.

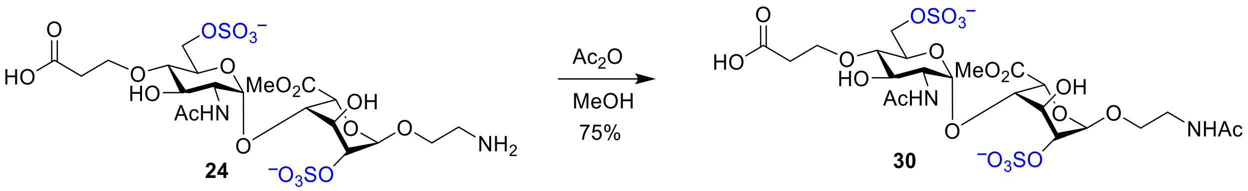

Acetamidoethyl 2-acetamido-4-*O*-carboxyethyl-2-deoxy-6-*O*-sulfonato-*α*-D-glucopyranosyl-(1→4)-methyl-2-*O*-sulfonato-*α*-L-idopyranosiduronate (30).

Compound **24** (19 mg, 0.03 mmol) was treated according to the general procedure of *N*-acetylation to give compound **30** (15 mg, 75%). ^{1}H NMR (500 MHz, D_2O) δ 4.98 (s, 1H, 1'-H), 4.96 (d, *J* = 3.5 Hz, 1H, 1-H), 4.81 (d, *J* = 2.2 Hz, 1H; 5-H), 4.18 (d, *J* = 3.0 Hz, 1H, 3-H), 4.14 – 4.10 (m, 3H; 2-H, 6'-CH2-), 3.93 (m, 1H; 4-H), 3.91 – 3.82 (m, 2H; 4'-linker-OCH2-), 3.79 – 3.72 (m, 1H; anomeric linker-OCH2-), 3.71 – 3.63 (m, 4H; -CO2CH3, 5'-H), 3.58 – 3.46 (m, 3H; 3'-H, 4'-H, anomeric linker-OCH2-), 3.26 – 3.19 (m, 3H; 2'-H, -CH2NHAc), 2.37 – 2.31 (m, 2H; CO2HCH2-), 1.89 (s, 3H; NHAc on linker), 1.82 (s, 3H; NHAc). ^{13}C NMR (125 MHz, D_2O) δ 174.64, 174.16, 171.21, 99.07, 94.35, 77.88, 73.08, 71.02, 69.63, 69.41, 67.33, 66.56, 66.32, 63.00, 53.07, 52.71, 48.75, 39.00, 37.35, 22.07, 21.68. HRMS: m/z calc. for $C_{22}H_{34}N_2O_{21}S_2^{2-}$ $[M]^{2-}$: 363.0553; found: 363.0555.

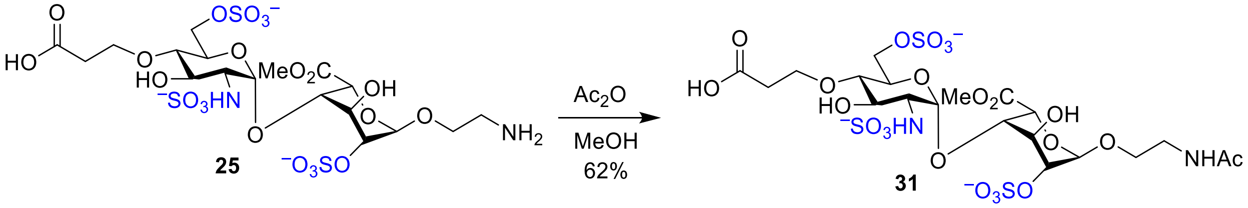

Acetamidoethyl 4-*O*-carboxyethyl-2-deoxy-2-sulfonatamido-6-*O*-sulfonato-*α*-D-glucopyranosyl-(1→4)-methyl-2-*O*-sulfonato-*α*-L-idopyranosiduronate (31).

Compound **25** (20 mg, 0.03 mmol) was treated according to the general procedure of *N*-acetylation to give compound **31** (13 mg, 62%). ^{1}H NMR (500 MHz, CD_3OD) δ 5.32 (d, *J* = 3.5 Hz, 1H, 1'-H), 5.15 (d, *J* = 2.7 Hz, 1H, 1-H), 4.80 (d, *J* = 2.9 Hz, 1H; 5-H), 4.36 – 4.32 (m, 1H; 3-H), 4.30 – 4.26 (m, 1H; 2-H), 4.21 (d, *J* = 2.8 Hz, 2H; 6'-CH2-), 4.14 – 3.92 (m, 3H; 4-H, 4'-linker-OCH2-), 3.81 (s, 4H; CO2CH3, anomeric linker-OCH2-), 3.72 – 3.67 (m, 1H; 5'-H), 3.64 – 3.59 (m, 1H; 4'-H), 3.58 – 3.49 (m, 1H; anomeric linker-OCH2-), 3.47 – 3.38 (m, 1H; 3'-H), 3.37 – 3.30 (m, 2H; -CH2NHAc), 3.28 – 3.23 (m, 1H, 2'-H), 2.64 – 2.47 (m, 2H, CO2HCH2-), 1.95 (s, 3H; -NHCOCH3). ^{13}C NMR (125 MHz, CD_3OD) δ 175.49, 171.98, 170.26, 99.76, 98.97, 78.53, 76.58, 75.02, 71.53, 69.71, 67.96, 67.85, 67.54, 67.41, 66.03, 58.04, 51.76, 38.81, 35.41, 21.13. HRMS: m/z calc. for $C_{20}H_{31}N_2O_{23}S_3^{3-}$ $[M+Na^+]^{2-}$: 393.0194; found: 393.0200.

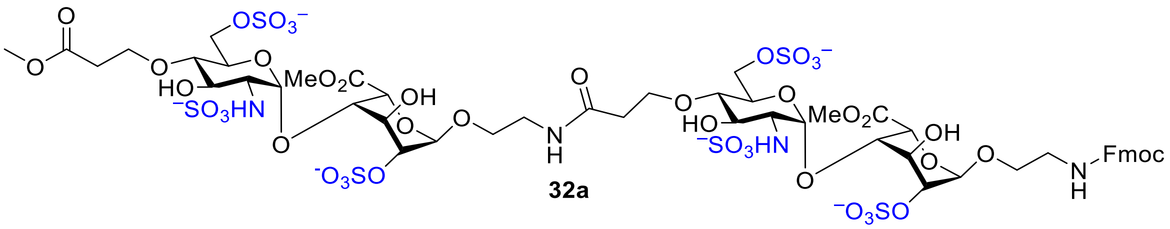

32a

32a. Starting material (9 mg, 0.012 mmol) was treated according to the general procedure of pseudo-tetrasaccharide preparation with HBTU to give compound **32a** (15 mg, 76%). ^{1}H NMR (500 MHz, D_2O) δ 7.79 (d, *J* = 7.5 Hz, 2H), 7.56 (t, *J* = 7.5 Hz, 2H), 7.40 – 7.33 (m, 2H), 7.32 – 7.23 (m, 2H), 5.17 – 5.08 (m, 2H; A-1, C-1), 4.94 (s, 2H; B-1), 4.89 (s, 1H; D-1), 4.54 (s, 1H), 4.42 – 4.34 (m, 2H), 4.31 – 4.24 (m, 2H), 4.22 – 4.00 (m, 9H), 3.95 – 3.79 (m, 5H), 3.75 (s, 2H), 3.67 – 3.61 (m, 3H), 3.55 (s, 3H), 3.52 – 3.38 (m, 8H), 3.28 – 3.22 (m, 2H), 3.19 (s, 3H), 3.12 – 3.03 (m, 3H), 2.55 – 2.47 (m, 2H), 2.39 – 2.29 (m, 2H). ^{13}C NMR (125 MHz, D_2O) δ 174.62, 173.99, 171.17, 148.60, 143.64, 140.89, 128.16, 128.02, 127.44, 125.17, 124.96, 120.70, 120.10, 99.61, 99.20, 77.44, 77.36, 77.18, 73.69, 70.62, 70.27, 69.13, 69.05, 68.27, 67.48, 67.31, 67.03, 66.40, 66.26, 66.05, 65.86, 57.91, 52.90, 52.67, 52.18, 46.91, 40.09, 38.89, 36.46, 34.74.

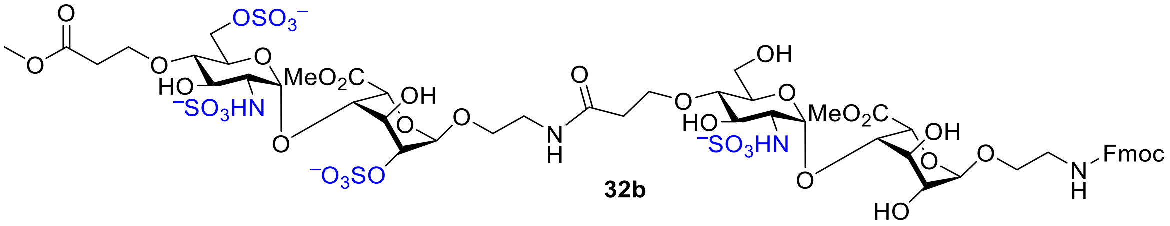

32b

32b. Starting material (13.5 mg, 0.018 mmol) was treated according to the general procedure of pseudo-tetrasaccharide preparation with HBTU to give compound **32b** (17 mg, 65%). ^{1}H NMR (500 MHz, D_2O) δ 7.77 (d, *J* = 7.5 Hz, 2H), 7.55 (t, *J* = 6.9 Hz, 2H), 7.36 (t, *J* = 7.5 Hz, 2H), 7.29 – 7.22 (m, 2H), 5.18 (d, *J* = 3.5 Hz, 1H; A-1), 5.13 (d, *J* = 3.5 Hz, 1H; C-1), 4.95 (s, 1H; B-1), 4.74 – 4.71 (m, 1H; D-1), 4.68 – 4.67 (m, 1H), 4.58 (d, *J* = 2.6 Hz, 1H), 4.42 – 4.35 (m, 1H), 4.31 – 4.23 (m, 2H), 4.16 – 4.00 (m, 5H), 3.95 (t, *J* = 2.6 Hz, 2H), 3.92 – 3.78 (m, 5H), 3.76 – 3.65 (m, 3H), 3.64 (s, 3H), 3.61 – 3.51 (m, 7H), 3.51 – 3.45 (m, 3H), 3.31 – 3.22 (m, 3H), 3.21 – 3.15 (m, 3H), 3.13 – 3.00 (m, 5H), 2.53 – 2.45 (m, 2H), 2.37 – 2.26 (m, 2H). ^{13}C NMR (151 MHz, D_2O) δ 98.93, 98.77, 97.07, 93.53, 77.98, 75.97, 75.40, 73.95, 70.97, 70.73, 69.36, 69.28, 68.88, 68.72, 68.64, 68.39, 66.94, 66.86, 66.78, 66.38, 66.30, 64.12, 57.84, 53.09, 38.99, 39.07, 36.25, 22.15, 21.83. HRMS: m/z calc. for $C_{37}H_{60}N_4O_{37}S_4^{4-}$ $[M+H^+]^{3-}$: 427.0638; found: 427.0630.

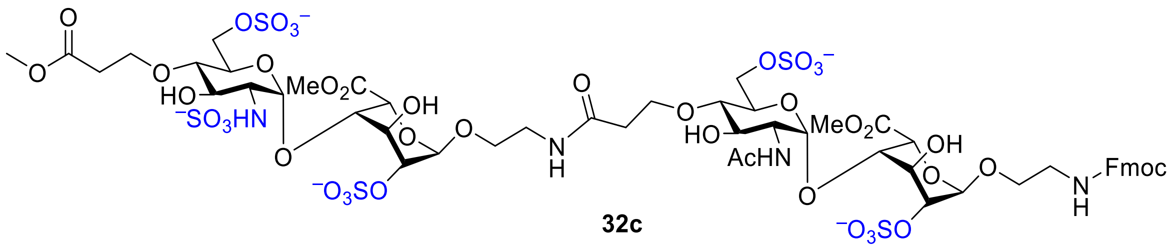

32c

32c. Starting material (18 mg, 0.024 mmol) was treated according to the general procedures of pseudo-tetrasaccharide preparation with HBTU to give compound **32c** (25 mg, 63%). ^{1}H NMR (500 MHz, CD_3OD) δ 7.80 (d, *J* = 7.5 Hz, 2H), 7.67 (d, *J* = 7.5 Hz, 2H), 7.44 – 7.37 (m, 2H), 7.35 – 7.29 (m, 2H), 5.28 (d, *J* = 3.6 Hz, 1H; A-1), 5.18 (d, *J* = 4.4 Hz, 2H; C-1, B-1), 5.00 (d, *J* = 3.4 Hz, 1H; D-1), 4.86 (d, *J* = 1.9 Hz, 1H), 4.84 (d, *J* = 2.4 Hz, 1H), 4.40 (t, *J* = 3.7 Hz, 1H), 4.35 – 4.27 (m, 5H), 4.25 – 4.15 (m, 5H), 4.13 – 4.05 (m, 2H), 4.03 – 3.94 (m, 5H), 3.84 – 3.76 (m, 6H), 3.74 – 3.71 (m, 2H), 3.69 (s, 3H), 3.66 – 3.54 (m, 6H), 3.48 – 3.34 (m, 6H), 3.26 (dd, *J* = 10.6, 3.4 Hz, 1H), 2.66 – 2.56 (m, 2H), 2.48 (t, *J* = 5.9 Hz, 2H), 2.08 (s, 3H). ^{13}C NMR (151 MHz, D_2O) δ 100.78, 99.17, 99.01, 97.07, 96.75, 95.79, 78.22, 77.90, 76.45, 75.97, 75.64, 74.52, 70.81, 69.20, 68.88, 68.72, 68.55, 68.39, 68.23, 67.59, 66.94, 66.30, 59.85, 57.92, 39.07, 36.33, 21.83. HRMS: m/z calc. for $C_{39}H_{61}N_4O_{41}S_5^{5-}$ $[M+H^+]^{4-}$: 350.5379; found: 350.5379.

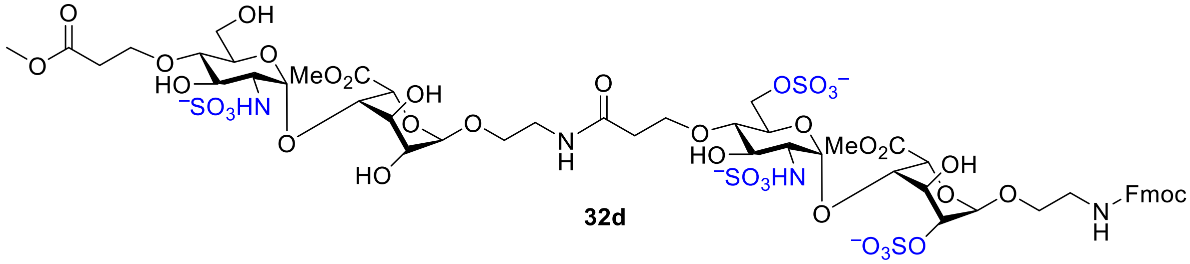

32d

32d. Starting material (21 mg, 0.036 mmol) was treated according to the general procedure of pseudo-tetrasaccharide preparation with HATU to give compound **32d** (32 mg, 59%). ^{1}H NMR (500 MHz, CD_3OD) δ 7.80 (d, *J* = 7.5 Hz, 2H), 7.67 (d, *J* = 7.4 Hz, 2H), 7.43 – 7.38 (m, 2H), 7.33 (td, *J* = 7.5, 1.2 Hz, 2H), 5.41 (d, *J* = 3.6 Hz, 1H; A-1), 5.28 – 5.19 (m, 2H; C-1, B-1), 4.97 (d, *J* = 2.8 Hz, 1H; D-1), 4.84 (t, *J* = 2.9 Hz, 2H), 4.43 (d, *J* = 3.2 Hz, 1H), 4.35 – 4.28 (m, 3H), 4.23 – 4.15 (m, 4H), 4.10 – 4.03 (m, 2H), 4.02 – 3.96 (m, 3H), 3.93 – 3.86 (m, 2H), 3.81 – 3.77 (m, 3H), 3.76 – 3.73 (m, 6H), 3.73 – 3.70 (m, 2H), 3.68 (s, 3H), 3.66 – 3.55 (m, 6H), 3.50 – 3.45 (m, 1H), 3.43 – 3.34 (m, 5H), 3.30 – 3.21 (m, 3H), 2.65 – 2.50 (m, 2H), 2.46 – 2.36 (m, 2H). ^{13}C NMR (125 MHz, D_2O) δ 100.70, 99.01, 96.91, 95.71, 78.14, 77.98, 75.97, 75.81, 74.60, 70.73, 70.65, 69.20, 68.88, 68.80, 68.64, 68.39, 68.31, 68.23, 68.07, 66.78, 66.70, 67.67, 66.30, 59.93, 57.84, 57.76, 52.93, 39.07, 37.13, 36.33, 34.48, 21.83. HRMS: m/z calc. for $C_{37}H_{60}N_4O_{37}S_4^{4-}$ $[M]^{4-}$: 320.0460; found: 320.0446.

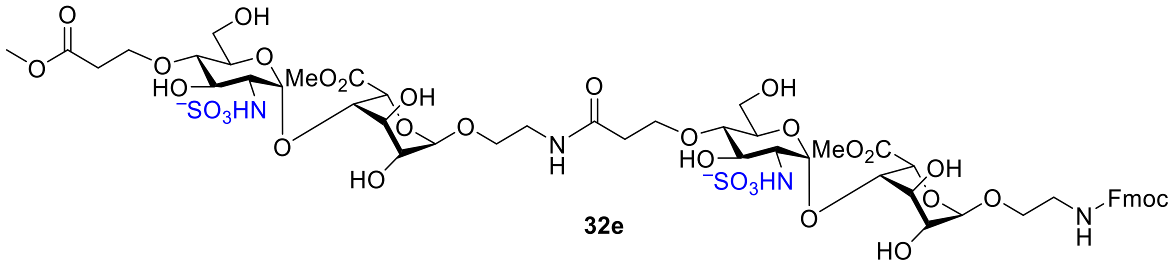

32e

32e. Starting material (12 mg, 0.020 mmol) was treated according to the general procedure of pseudo-tetrasaccharide preparation with HATU to give compound **32e** (20 mg, 70%). ^{1}H NMR (500 MHz, CD_3OD) δ 7.81 (d, *J* = 7.5 Hz, 2H), 7.66 (d, *J* = 7.5 Hz, 2H), 7.40 (t, *J* = 7.5 Hz, 2H), 7.33 (t, *J* = 7.5 Hz, 2H), 5.35 (d, *J* = 3.5 Hz, 1H; A-1), 5.32 (d, *J* = 3.5 Hz, 1H; C-1), 4.97 (d, *J* = 2.6 Hz, 1H; B-1), 4.82 – 4.77 (m, 2H; D-1), 4.37 – 4.27 (m, 2H), 4.22 – 4.16 (m, 3H), 4.10 – 3.95 (m, 5H), 3.92 – 3.85 (m, 3H), 3.76 – 3.72 (m, 5H), 3.73 – 3.68 (m, 8H), 3.67 (s, 3H), 3.63 – 3.59 (m, 4H), 3.58 – 3.53 (m, 4H), 3.48 – 3.43 (m, 3H), 3.32 – 3.29 (m, 4H), 2.62 – 2.47 (m, 2H), 2.41 – 2.32 (m, 2H). ^{13}C NMR (125 MHz, CD_3OD) δ 173.03, 172.75, 170.43, 143.93, 141.17, 127.38, 126.80, 124.84, 119.50, 101.54, 101.37, 96.93, 96.39, 78.14, 78.08, 74.57, 74.04, 72.13, 71.63, 71.44, 69.30, 69.01, 67.92, 67.88, 67.46, 67.27, 67.21, 66.40, 60.62, 60.46, 58.45, 58.30, 54.42, 53.98, 51.50, 51.41, 50.66, 46.48, 42.39, 40.27, 38.88, 37.97, 36.57, 34.78. HRMS: m/z calc. for $C_{37}H_{62}N_4O_{31}S_2^{2-}$ $[M]^{2-}$: 561.1425; found: 561.1426.

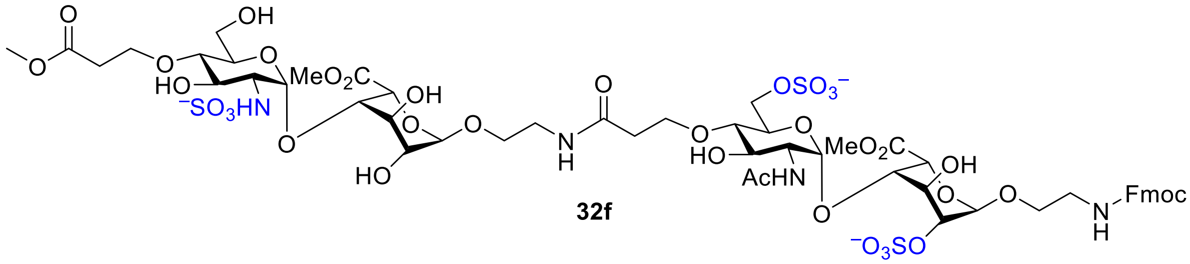

32f

32f. Starting material (8 mg, 0.013 mmol) was treated according to the general procedure of pseudo-tetrasaccharide preparation with HATU to give compound **32f** (20 mg, 61%). ^{1}H NMR (500 MHz, CD_3OD) δ 7.81 (d, *J* = 7.5 Hz, 2H), 7.67 (d, *J* = 7.6 Hz, 2H), 7.40 (tt, *J* = 7.6, 1.6 Hz, 2H), 7.33 (tt, *J* = 7.5, 1.4 Hz, 2H), 5.37 (d, *J* = 3.6 Hz, 1H; A-1), 5.18 (s, 1H; C-1), 4.96 (d, *J* = 3.4 Hz, 1H; B-1), 4.92 (d, *J* = 2.9 Hz, 1H; D-1), 4.85 (d, *J* = 2.0 Hz, 1H), 4.83 (d, *J* = 3.0 Hz, 1H), 4.35 – 4.27 (m, 4H), 4.24 – 4.15 (m, 4H), 4.11 – 4.04 (m, 2H), 4.03 – 3.94 (m, 5H), 3.85 – 3.82 (m, 2H), 3.81 – 3.74 (m, 5H), 3.73 – 3.66 (m, 9H), 3.64 – 3.52 (m, 5H), 3.43 – 3.37 (m, 2H), 3.29 – 3.19 (m, 2H), 2.64 – 2.52 (m, 3H), 2.49 – 2.39 (m, 3H), 2.09 (s, 3H). ^{13}C NMR (125 MHz, CD_3OD) δ 173.03, 172.97, 172.79,

170.47, 170.40, 143.90, 141.14, 127.37, 126.81, 124.90, 124.87, 119.47, 101.36, 99.53, 97.08, 96.29, 77.28, 78.12, 74.22, 73.87, 73.06, 71.87, 71.48, 71.42, 69.92, 69.17, 68.31, 67.87, 67.50, 67.29, 67.16, 66.48, 66.10, 65.84, 64.69, 60.55, 58.16, 53.60, 51.76, 51.51, 50.69, 40.18, 38.75, 36.62, 34.74, 21.75. HRMS: m/z calc. for $C_{39}H_{63}N_4O_{35}S_3^{3-}$ $[M+H^+]^{2-}$: 622.1262; found: 622.1260.

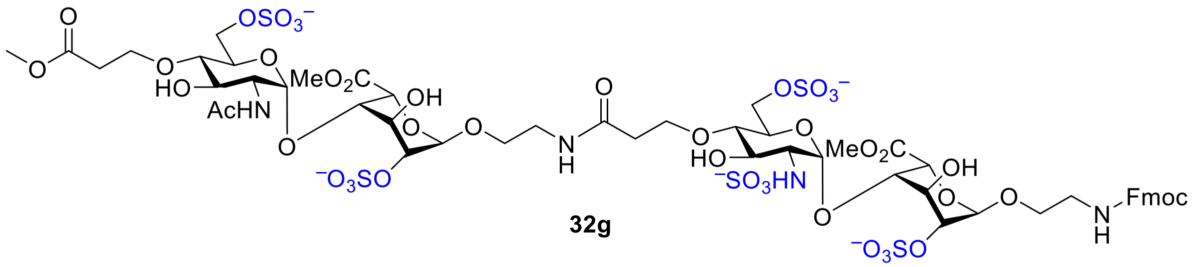

32g

32g. Starting material (22 mg, 0.03 mmol) was treated according to the general procedures of pseudo-tetrasaccharide preparation with HATU to give compound **32g** (26 mg, 62%). ^{1}H NMR (500 MHz, CD_3OD) δ 8.00 (d, *J* = 7.6 Hz, 2H), 7.78 (t, *J* = 7.0 Hz, 2H), 7.58 (td, *J* = 7.6, 3.6 Hz, 2H), 7.52 – 7.46 (m, 2H), 5.37 (d, *J* = 3.6 Hz, 1H; A-1), 5.19 (s, 1H; C-1), 5.15 – 5.07 (m, 2H; B-1, D-1), 4.98 (s, 1H), 4.76 (d, *J* = 2.2 Hz, 1H), 4.64 – 4.59 (m, 1H), 4.52 – 4.47 (m, 1H), 4.45 – 4.20 (m, 9H), 4.18 – 3.94 (m, 8H), 3.89 – 4.81 (m, 5H), 3.76 (s, 4H), 3.72 – 3.55 (m, 7H), 3.51 – 3.41 (m, 4H), 3.33 – 3.22 (m, 3H), 2.77 – 2.66 (m, 2H), 2.56 (t, *J* = 5.9 Hz, 2H), 2.10 (s, 3H). ^{13}C NMR (125 MHz, CD_3OD) δ 173.39, 173.31, 172.68, 169.77, 169.41, 142.34, 139.55, 126.65, 126.07, 123.81, 123.58, 118.73, 98.01, 97.65, 97.22, 93.48, 76.05, 75.93, 75.20, 73.28, 71.48, 70.13, 69.61, 69.24, 68.15, 67.75, 66.88, 66.52, 65.98, 65.82, 65.42, 64.86, 64.74, 64.66, 61.61, 56.36, 51.32, 51.62, 51.49, 50.81, 47.38, 45.57, 38.71, 37.54, 35.12, 33.52, 20.69. HRMS: m/z calc. for $C_{39}H_{61}N_4O_{41}S_5^{5-}$ $[M]^{5-}$: 280.2288; found: 280.2278.

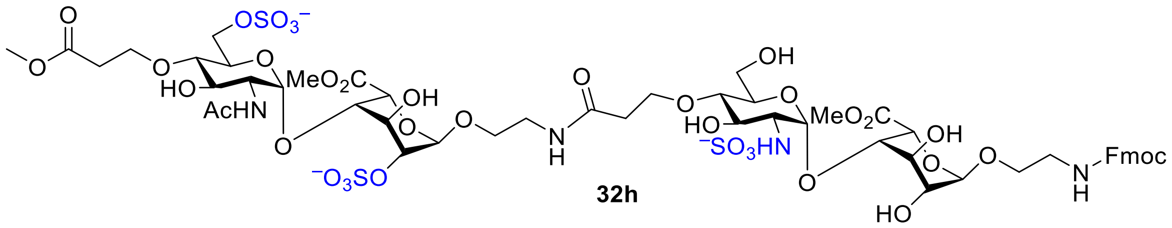

32h

32h. Starting material (8.5 mg, 0.01 mmol) was treated according to the general procedure of pseudo-tetrasaccharide preparation with HATU to give compound **32h** (11.7 mg, 80%). ^{1}H NMR (500 MHz, CD_3OD) δ 7.81 (d, *J* = 7.5 Hz, 2H), 7.66 (d, *J* = 7.5 Hz, 2H), 7.44 – 7.38 (m, 2H), 7.33 (td, *J* = 7.5, 1.2 Hz, 2H), 5.35 (d, *J* = 3.7 Hz, 1H; A-1), 5.16 (s, 1H; C-1), 4.99 (d, *J* = 3.5 Hz, 1H; B-

1), 4.92 (d, *J* = 2.7 Hz, 2H; D-1), 4.87 (d, *J* = 2.0 Hz, 1H), 4.80 (d, *J* = 2.9 Hz, 1H), 4.36 – 4.24 (m, 5H), 4.22 – 4.13 (m, 4H), 4.12 – 3.93 (m, 7H), 3.93 – 3.85 (m, 2H), 3.85 – 3.74 (m, 9H), 3.65 – 3.52 (m, 8H), 3.48 – 3.38 (m, 4H), 3.27 – 3.19 (m, 1H), 2.63 – 2.56 (m, 2H), 2.55 – 2.38 (m, 3H), 2.08 (s, 3H). ^{13}C NMR (125 MHz, CD_3OD) δ 173.03, 172.96, 172.89, 170.43, 143.92, 141.18, 127.38, 126.79, 124.84, 119.50, 101.37, 99.69, 96.98, 96.69, 78.32, 78.27, 74.30, 74.12, 73.31, 71.59, 71.56, 71.50, 69.98, 69.13, 67.87, 67.78, 67.66, 67.60, 67.27, 66.39, 66.34, 65.93, 64.82, 60.48, 58.19, 53.59, 51.78, 51.60, 40.24, 38.79, 36.43, 34.86, 21.74. HRMS: m/z calc. for $C_{39}H_{63}N_4O_{35}S_3^{3-}$ $[M+H^+]^{2-}$: 622.1262; found: 622.1260.

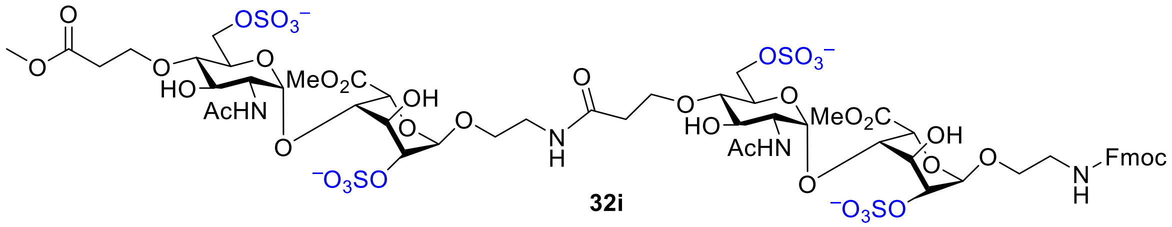

32i

32i. Starting material (8.0 mg, 0.01 mmol) was treated according to the general procedure of pseudo-tetrasaccharide preparation with HATU to give compound **32i** (12.7 mg, 80%). ^{1}H NMR (500 MHz, CD_3OD) δ 7.81 (d, *J* = 7.5 Hz, 2H), 7.67 (d, *J* = 7.5 Hz, 2H), 7.40 (tt, *J* = 7.5, 1.5 Hz, 2H), 7.33 (tt, *J* = 7.5, 1.5 Hz, 2H), 5.16 (d, *J* = 4.8 Hz, 2H; A-1, C-1), 4.99 (d, *J* = 3.5 Hz, 1H; B-1), 4.97 (d, *J* = 3.4 Hz, 1H; D-1), 4.86 (dd, *J* = 5.6, 1.9 Hz, 2H), 4.34 – 4.25 (m, 6H), 4.22 – 4.18 (m, 5H), 4.11 – 4.05 (m, 3H), 4.02 – 3.93 (m, 6H), 3.82 (s, 3H), 3.74 – 3.66 (m, 7H), 3.66 – 3.54 (m, 6H), 3.43 – 3.37 (m, 3H), 3.35 – 3.33 (m, 2H), 3.30 (d, *J* = 2.7 Hz, 1H), 2.62 – 2.56 (m, 2H), 2.49 – 2.43 (m, 2H), 2.07 (d, *J* = 1.7 Hz, 6H). ^{13}C NMR (125 MHz, CD_3OD) δ 172.96, 172.92, 170.49, 170.33, 157.48, 143.90, 141.14, 127.38, 126.81, 124.89, 119.48, 99.61, 99.56, 97.23, 96.43, 78.34, 78.26, 74.36, 73.47, 73.09, 71.52, 71.42, 69.99, 69.91, 68.22, 67.76, 67.55, 67.25, 66.49, 66.27, 66.22, 65.91, 64.79, 64.40, 53.60, 53.40, 51.83, 51.75, 50.82, 40.17, 38.67, 36.46, 34.85, 21.74. HRMS: m/z calc. for $C_{41}H_{64}N_4O_{39}S_4^{4-}$ $[M+H^+]^{3-}$: 455.0708; found: 455.0688.

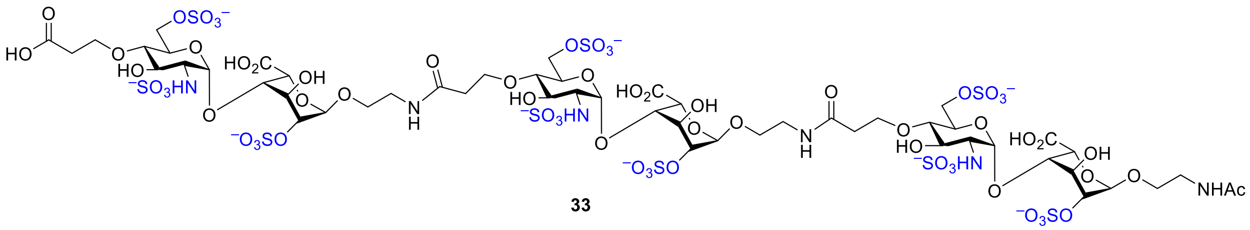

33

33. The starting material (5.8 mg, 0.004 mmol) was treated according to the general procedures of NH-Fmoc deprotection, pseudo-hexasaccharide preparation with HBTU and saponification of

methyl esters to give compound **33** (3.8 mg, 51% for 3 steps). $[\alpha]_D^{20}$ +50º (c 0.05, D_2O); ^1H NMR (500 MHz, D_2O) δ 5.22 (d, *J* = 3.6 Hz, 1H; A-1), 5.16 (t, *J* = 4.2 Hz, 2H; C-1, F-1), 5.00 (d, *J* = 3.6 Hz, 2H; B-1, D-1), 4.95 (d, *J* = 2.8 Hz, 1H; E-1), 4.45 (dd, *J* = 11.5, 2.3 Hz, 1H), 4.41 (d, *J* = 2.8 Hz, 1H), 4.17 (dd, *J* = 11.0, 2.1 Hz, 1H), 4.13 – 4.08 (m, 6H), 4.07 – 3.99 (m, 6H), 3.94 – 3.62 (m, 15H), 3.57 – 3.44 (m, 6H), 3.32 – 3.16 (m, 9H), 3.13 – 3.05 (m, 3H), 2.53 – 2.34 (m, 6H), 1.82 (s, 3H). ^{13}C NMR (151 MHz, D_2O, from HSQC) δ 98.85, 98.53, 96.75, 96.59, 96.11, 77.74, 76.13, 75.48, 74.84, 70.33, 69.52, 68.72, 68.55, 68.39, 67.75, 67.59, 66.62, 66.46, 65.98, 57.44, 38.75, 37.46, 35.85, 21.34. HRMS: m/z calc. for $C_{53}H_{79}N_6O_{65}S_9^{9-}$ $[M+4Na^+]^{5-}$: 443.8033; found: 443.8041.

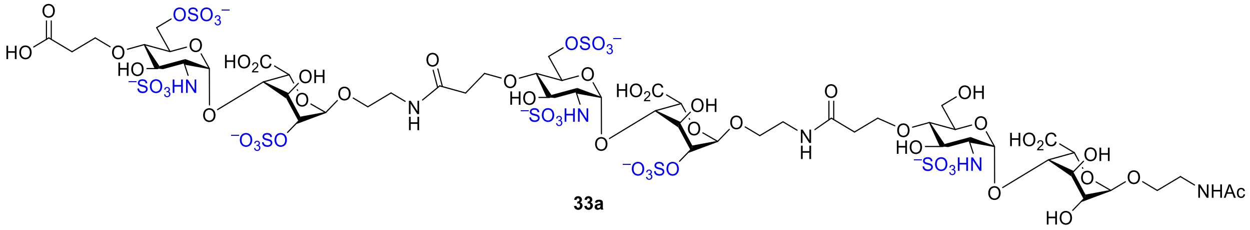

33a

33a. The starting material (6.5 mg, 0.004 mmol) was treated according to the general procedures of NH-Fmoc deprotection, pseudo-hexasaccharide preparation with HBTU and saponification of methyl esters to give compound **33a** (3.7 mg, 48% for 3 steps). $[\alpha]_D^{20}$ +44º (c 0.1, D_2O); ^1H NMR (500 MHz, D_2O) δ 5.19 (d, *J* = 3.6 Hz, 1H; A-1), 5.15 (dd, *J* = 6.2, 3.6 Hz, 2H; C-1, F-1), 5.01 – 4.96 (m, 2H; B-1, D-1), 4.73 – 4.70 (m, 1H; E-1), 4.44 – 4.33 (m, 3H), 4.18 – 4.13 (m, 1H), 4.12 – 4.05 (m, 5H), 4.04 – 3.99 (m, 2H), 3.98 – 3.93 (m, 1H), 3.92 – 3.71 (m, 11H), 3.70 – 3.44 (m, 13H), 3.32 – 3.15 (m, 8H), 3.12 – 3.00 (m, 4H), 2.50 – 2.35 (m, 6H), 1.82 (s, 3H). ^{13}C NMR (125 MHz, D_2O) δ 173.84, 100.61, 98.90, 98.70, 97.47, 97.09, 95.60, 78.04, 77.82, 77.64, 75.98, 75.95, 75.38, 74.69, 74.43, 70.68, 70.54, 70.47, 68.79, 68.46, 68.27, 68.22, 68.18, 67.78, 67.66, 67.52, 66.77, 66.71, 66.16, 59.75, 57.77, 57.75, 57.72, 39.05, 38.97, 38.91, 36.19, 21.70. HRMS: m/z calc. for $C_{53}H_{81}N_6O_{59}S_7^{7-}$ $[M+2Na^+]^{5-}$: 403.0278; found: 403.0291.

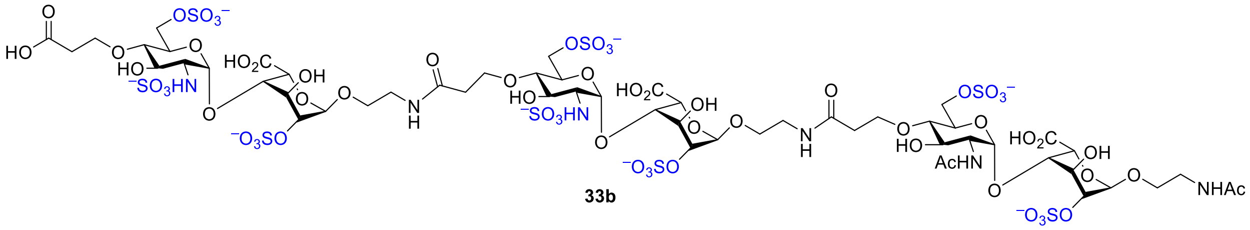

33b

33b. The starting material (5.8 mg, 0.004 mmol) was treated according to the general procedures of NH-Fmoc deprotection, pseudo-hexasaccharide preparation with HBTU and saponification of

methyl esters to give compound **33b** (1.4 mg, 19% for 3 steps). $[\alpha]_D^{20}$ +24.1° (c 0.17, D_2O); ^1H NMR (500 MHz, D_2O) δ 5.17 (dd, *J* = 8.9, 3.5 Hz, 2H; A-1, C-1), 4.98 (s, 2H; F-1, B-1), 4.94 (d, *J* = 3.7 Hz, 1H; D-1), 4.91 (s, 1H; E-1), 4.43 – 4.34 (m, 3H), 4.19 – 3.99 (m, 11H), 3.94 – 3.72 (m, 10H), 3.71 – 3.43 (m, 11H), 3.35 – 3.16 (m, 10H), 3.13 – 3.03 (m, 3H), 2.47 – 2.35 (m, 6H), 1.88 (s, 3H), 1.82 (s, 3H). ^{13}C NMR (151 MHz, D_2O, from HSQC) δ 98.93, 98.77, 97.07, 93.53, 77.98, 75.97, 75.40, 73.95, 70.97, 70.73, 69.36, 69.28, 68.88, 68.72, 68.64, 68.39, 66.94, 66.86, 66.78, 66.38, 66.30, 64.12, 57.84, 53.09, 39.07, 38.99, 36.25, 22.15, 21.83. HRMS: m/z calc. for $C_{55}H_{82}N_6O_{63}S_8^{8-}$ $[M+3Na^+]^{5-}$: 431.8177; found: 431.8189.

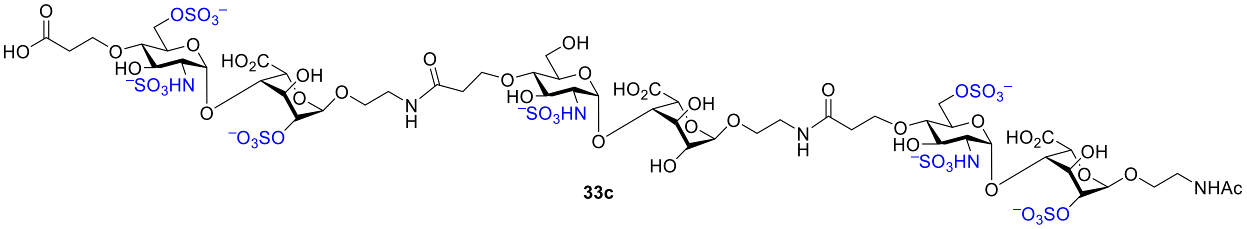

33c

33c. The starting material (4.5 mg, 0.003 mmol) was treated according to the general procedures of NH-Fmoc deprotection, pseudo-hexasaccharide preparation with HBTU and saponification of methyl esters to give compound **33c** (1.4 mg, 51% for 3 steps). $[\alpha]_D^{20}$ +15.9° (c 0.27, D_2O); ^1H NMR (500 MHz, D_2O) δ 5.22 (d, *J* = 3.5 Hz, 1H; A-1), 5.18 (d, *J* = 3.6 Hz, 1H; C-1), 5.14 (d, *J* = 3.9 Hz, 1H; E-1), 4.97 (d, *J* = 2.4 Hz, 1H; B-1), 4.94 (d, *J* = 2.9 Hz, 1H; D-1), 4.73 (d, *J* = 2.7 Hz, 1H; F-1), 4.41 – 4.32 (m, 3H), 4.19 – 3.99 (m, 8H), 3.97 – 3.92 (m, 1H), 3.92 – 3.73 (m, 11H), 3.71 – 3.42 (m, 14H), 3.37 – 3.16 (m, 8H), 3.12 – 3.01 (m, 3H), 2.52 – 2.30 (m, 6H), 1.82 (s, 3H). ^{13}C NMR (151 MHz, D_2O, from HSQC) δ 100.78, 99.17, 99.01, 97.07, 96.75, 95.79, 78.22, 77.90, 76.45, 75.97, 75.64, 74.52, 70.81, 69.20, 68.88, 68.72, 68.55, 68.39, 68.23, 67.59, 66.94, 66.30, 59.85, 57.92, 39.07, 36.33, 21.83. HRMS: m/z calc. for $C_{53}H_{81}N_6O_{59}S_7^{7-}$ $[M+2Na^+]^{5-}$: 403.0278; found: 403.0296.

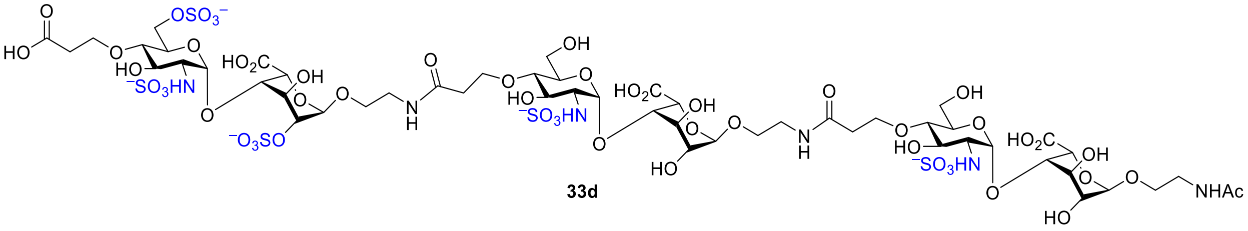

33d

33d. The starting material (4.5 mg, 0.003 mmol) was treated according to the general procedures of NH-Fmoc deprotection, pseudo-hexasaccharide preparation with HBTU and saponification of methyl esters to give compound **33d** (3.0 mg, 56% for 3 steps). $[\alpha]_D^{20}$ +40.0° (c 0.15, D_2O); ^1H NMR (500 MHz, D_2O) δ 5.18 (d, *J* = 3.7 Hz, 1H; A-1), 5.14 (dd, *J* = 3.9, 1.4 Hz, 2H; C-1, E-1), 4.97 (d, *J* = 2.5 Hz, 1H; B-1), 4.74 – 4.71 (m, 2H; D-1, F-1), 4.39 – 4.32 (m, 3H), 4.18 – 4.14 (m, 1H), 4.12 –

3.99 (m, 4H), 3.97 – 3.92 (m, 2H), 3.91 – 3.72 (m, 10H), 3.70 – 3.45 (m, 14H), 3.31 – 3.16 (m, 10H), 3.10 – 2.98 (m, 4H), 2.48 – 2.33 (m, 6H), 1.82 (s, 3H). ^{13}C NMR (151 MHz, D_2O, from HSQC) δ 100.70, 99.01, 96.91, 95.71, 78.14, 77.98, 75.97, 75.81, 74.60, 70.73, 70.65, 69.20, 68.88, 68.80, 68.64, 68.39, 68.31, 68.23, 68.07, 67.67, 66.78, 66.70, 66.30, 59.93, 57.84, 57.76, 52.93, 39.07, 37.13, 36.33, 34.48, 21.83. HRMS: m/z calc. for $C_{53}H_{83}N_6O_{53}S_5^{5-}$ $[M+Na^+]^{4-}$: 458.5627; found: 458.5641.

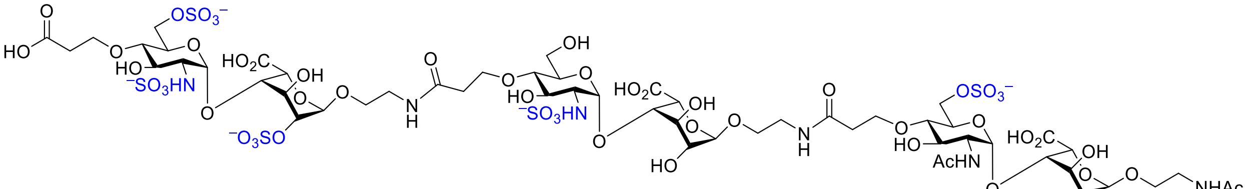

33e

33e. The starting material (4.5 mg, 0.003 mmol) was treated according to the general procedures of NH-Fmoc deprotection, pseudo-hexasaccharide preparation with HBTU and saponification of methyl esters to give compound **33e** (2.9 mg, 50% for 3 steps). $[\alpha]_D^{20}$ +55.7° (c 0.07, D_2O); ^{1}H NMR (500 MHz, D_2O) δ 7.96 (d, J = 9.8 Hz, 1H; NHAc), 5.18 (d, J = 3.7 Hz, 1H; A-1), 5.14 (d, J = 3.7 Hz, 1H; C-1), 4.98 – 4.90 (m, 3H; D-1, B-1, E-1), 4.75 – 4.71 (m, 1H; F-1), 4.39 (d, J = 2.3 Hz, 1H), 4.35 (dd, J = 6.9, 2.4 Hz, 2H), 4.17 – 4.00 (m, 7H), 3.97 – 3.74 (m, 12H), 3.70 – 3.44 (m, 14H), 3.35 – 3.17 (m, 9H), 3.10 – 3.01 (m, 3H), 2.46 – 2.34 (m, 6H), 1.88 (s, 3H), 1.83 (s, 3H). ^{13}C NMR (126 MHz, D_2O) δ 190.14, 173.85, 109.99, 100.55, 98.85, 98.64, 96.97, 95.61, 93.51, 77.77, 75.88, 75.41, 74.39, 73.67, 70.85, 70.81, 70.73, 70.53, 68.83, 68.72, 68.59, 68.27, 68.23, 68.10, 67.42, 66.85, 66.71, 66.44, 66.23, 66.18, 63.82, 59.76, 57.71, 53.04, 39.00, 38.87, 36.28, 36.13, 22.07, 21.70. HRMS: m/z calc. for $C_{55}H_{84}N_6O_{57}S_6^{6-}$ $[M+Na^+]^{5-}$: 391.0422; found: 391.0433.

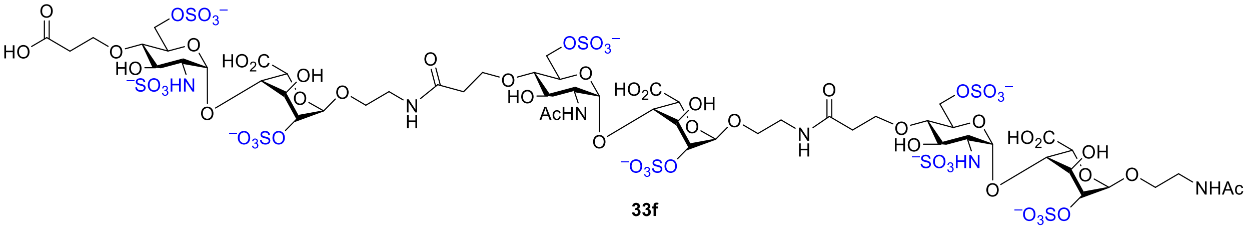

33f

33f. The starting material (5.2 mg, 0.003 mmol) was treated according to the general procedures of NH-Fmoc deprotection, pseudo-hexasaccharide preparation with HBTU and saponification of methyl esters to give compound **33f** (3.7 mg, 55% for 3 steps). $[\alpha]_D^{20}$ +24.2° (c 0.31, D_2O); ^{1}H NMR (500 MHz, D_2O) δ 5.22 (d, J = 3.5 Hz, 1H; A-1), 5.16 (d, J = 3.5 Hz, 1H; C-1), 5.00 – 4.97 (m, 1H;

E-1), 4.94 – 4.86 (m, 3H; B-1, D-1, F-1), 4.46 – 4.38 (m, 3H), 4.20 – 3.98 (m, 11H), 3.97 – 3.71 (m, 12H), 3.66 – 3.58 (m, 3H), 3.60 – 3.44 (m, 6H), 3.38 – 3.15 (m, 9H), 3.09 – 2.96 (m, 2H), 2.54 – 2.45 (m, 2H), 2.44 – 2.35 (m, 6H), 1.88 (s, 3H), 1.82 (s, 3H). ^{13}C NMR (151 MHz, D_2O, from HSQC) δ 99.25, 98.85, 98.69, 97.24, 96.67, 93.69, 77.90, 76.45, 76.05, 75.24, 74.27, 73.39, 72.91, 70.89, 70.65, 69.12, 69.04, 68.88, 68.72, 68.23, 68.15, 66.86, 66.78, 66.38, 66.30, 64.37, 63.64, 57.84, 53.09, 39.07, 37.62, 36.33, 35.44, 34.56. HRMS: m/z calc. for $C_{55}H_{82}N_6O_{63}S_8^{8-}$ $[M+Na^+]^{7-}$: 301.8728; found: 301.8744.

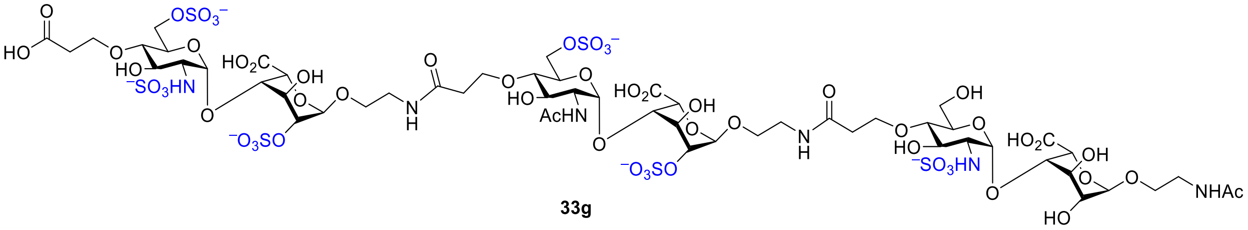

33g

33g. The starting material (4.8 mg, 0.003 mmol) was treated according to the general procedures of NH-Fmoc deprotection, pseudo-hexasaccharide preparation with HBTU and saponification of methyl esters to give compound **33g** (2.5 mg, 44% for 3 steps). $[\alpha]_D^{20}$ +20.8º (c 0.25, D_2O); ^{1}H NMR (500 MHz, D_2O) δ 5.17 (d, *J* = 3.7 Hz, 1H; A-1), 5.14 (d, *J* = 3.6 Hz, 1H; C-1), 4.99 – 4.97 (m, 1H; E-1), 4.93 (d, *J* = 3.1 Hz, 2H; B-1, D-1), 4.73 – 4.69 (m, 1H; F-1), 4.39 – 4.35 (m, 2H), 4.33 (d, *J* = 2.4 Hz, 1H), 4.18 – 3.99 (m, 8H), 3.95 (t, *J* = 3.9 Hz, 2H), 3.92 – 3.72 (m, 11H), 3.70 – 3.42 (m, 12H), 3.36 – 3.17 (m, 9H), 3.13 – 3.01 (m, 3H), 2.51 – 2.32 (m, 6H), 1.88 (s, 3H), 1.81 (s, 3H). ^{13}C NMR (151 MHz, D_2O, from HSQC) δ 100.70, 98.93, 98.77, 97.16, 95.62, 93.61, 78.14, 77.98, 75.97, 75.32, 74.60, 73.63, 70.89, 70.73, 70.41, 70.09, 69.44, 68.96, 68.88, 68.80, 68.55, 68.31, 68.07, 67.83, 66.78, 66.30, 63.80, 59.93, 57.84, 53.09, 39.15, 36.25, 22.23, 21.83. HRMS: m/z calc. for $C_{55}H_{84}N_6O_{57}S_6^{6-}$ $[M+Na^+]^{5-}$: 391.0422; found: 391.0435.

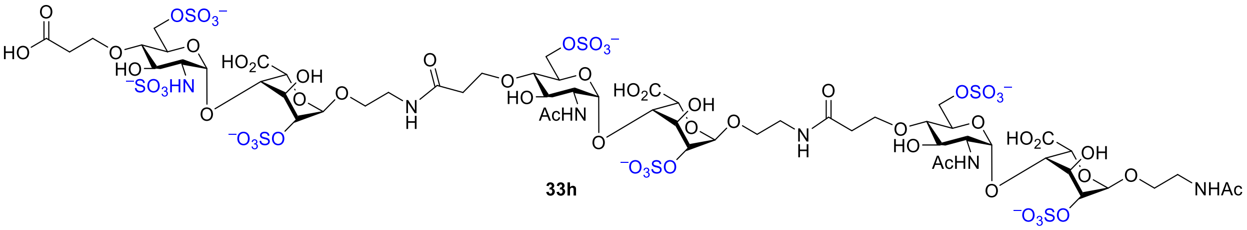

33h

33h. The starting material (5.2 mg, 0.003 mmol) was treated according to the general procedures of NH-Fmoc deprotection, pseudo-hexasaccharide preparation with HBTU and saponification of methyl esters to give compound **33h** (2.8 mg, 42% for 3 steps). $[\alpha]_D^{20}$ +30º (c 0.21, D_2O); ^{1}H NMR

(500 MHz, D_2O) δ 5.16 (d, *J* = 3.7 Hz, 1H; A-1), 4.98 (s, 1H; C-1), 4.93 – 4.89 (m, 4H; B-1, D-1, E-1, F-1), 4.46 – 4.38 (m, 3H), 4.22 – 3.99 (m, 12H), 3.94 – 3.71 (m, 13H), 3.69 – 3.62 (m, 3H), 3.60 – 3.41 (m, 6H), 3.35 – 3.19 (m, 9H), 3.17 (s, 1H), 3.08 (dd, *J* = 10.6, 3.5 Hz, 1H), 2.52 – 2.28 (m, 6H), 1.88 (s, 6H), 1.82 (s, 3H). ^{13}C NMR (125 MHz, D_2O) δ 174.62, 174.15, 173.87, 98.73, 98.67, 98.58, 97.46, 93.65, 93.54, 77.82, 77.62, 76.01, 74.77, 73.65, 73.26, 70.83, 70.78, 70.45, 68.88, 68.79, 68.62, 68.58, 68.48, 68.15, 67.72, 66.87, 66.79, 66.70, 66.22, 66.10, 63.79, 57.77, 53.01, 39.00, 38.93, 38.86, 36.20, 36.15, 35.39, 22.09, 21.69. HRMS: m/z calc. for $C_{57}H_{85}N_6O_{61}S_7^{7-}$ $[M+2Na^+]^{5-}$: 419.8320; found: 419.8341.

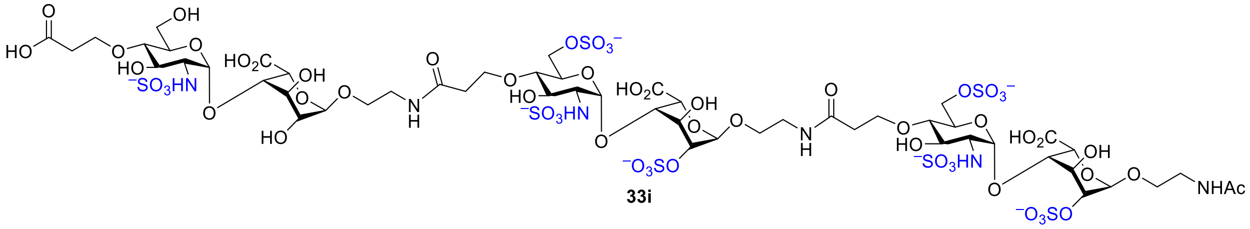

33i

33i. The starting material (4.6 mg, 0.003 mmol) was treated according to the general procedures of NH-Fmoc deprotection, pseudo-hexasaccharide preparation with HBTU and saponification of methyl esters to give compound **33i** (1.2 mg, 20% for 3 steps). $[\boldsymbol{\alpha}]_{\mathbf{D}}^{\mathbf{20}}$ +22.0º (c 0.1, D_2O); ^{1}H NMR (500 MHz, D_2O) δ 5.24 (s, 1H; A-1), 5.15 – 5.10 (m, 2H; C-1, E-1), 4.99 (s, 1H; B-1), 4.93 (s, 2H; D-1, F-1), 4.38 – 4.31 (m, 3H), 4.14 – 3.99 (m, 8H), 3.96 – 3.71 (m, 12H), 3.69 – 3.42 (m, 13H), 3.36 – 3.15 (m, 9H), 3.12 – 3.01 (m, 4H), 2.44 – 2.32 (m, 5H), 1.82 (d, *J* = 1.8 Hz, 3H). ^{13}C NMR (151 MHz, D_2O, from HSQC) δ 100.62, 99.33, 97.07, 96.43, 95.71, 78.22, 77.98, 76.85, 76.05, 75.97, 75.48, 74.60, 70.81, 69.60, 69.44, 68.80, 68.55, 68.47, 68.31, 68.15, 67.67, 66.86, 66.62, 66.38, 66.30, 59.93, 57.84, 57.76, 39.07, 38.99, 37.94, 36.81, 36.33, 21.83. HRMS: m/z calc. for $C_{53}H_{81}N_6O_{59}S_7^{7-}$ $[M+2Na^+]^{5-}$: 403.0278; found: 403.0296.

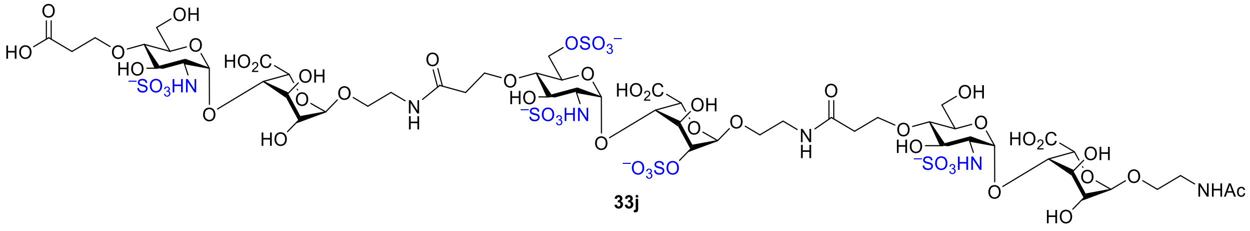

33j

33j. The starting material (4.2 mg, 0.003 mmol) was treated according to the general procedures of NH-Fmoc deprotection, pseudo-hexasaccharide preparation with HBTU and saponification of methyl esters to give compound **33j** (2.4 mg, 48% for 3 steps). $[\boldsymbol{\alpha}]_{\mathbf{D}}^{\mathbf{20}}$ +110º (c 0.05, D_2O); ^{1}H NMR

(500 MHz, D_2O) δ 5.17 (d, *J* = 2.9 Hz, 1H; A-1), 5.14 (t, *J* = 2.5 Hz, 2H; C-1, E-1), 4.98 (s, 1H; B-1), 4.72 (dd, *J* = 5.4, 2.5 Hz, 2H; D-1, F-1), 4.46 – 4.35 (m, 3H), 4.12 – 4.05 (m, 3H), 4.03 – 3.83 (m, 9H), 3.83 – 3.69 (m, 6H), 3.68 – 3.44 (m, 15H), 3.36 – 3.13 (m, 9H), 3.11 – 2.99 (m, 3H), 2.46 – 2.34 (m, 6H), 1.81 (d, *J* = 1.9 Hz, 3H). ^{13}C NMR (125 MHz, D_2O) δ 173.88, 170.07, 100.62, 100.60, 98.86, 97.38, 95.63, 77.98, 77.81, 77.66, 76.06, 75.08, 74.38, 74.22, 70.75, 70.66, 70.57, 70.51, 68.79, 68.19, 68.15, 68.09, 67.97, 67.91, 67.64, 67.41, 67.28, 66.82, 66.73, 66.54, 66.11, 59.73, 57.81, 57.70, 39.04, 38.98, 38.88, 36.33, 36.18, 35.58, 21.69. HRMS: m/z calc. for $C_{53}H_{83}N_6O_{53}S_5^{5-}$ $[M+Na^+]^{4-}$: 458.5627; found: 458.5641.

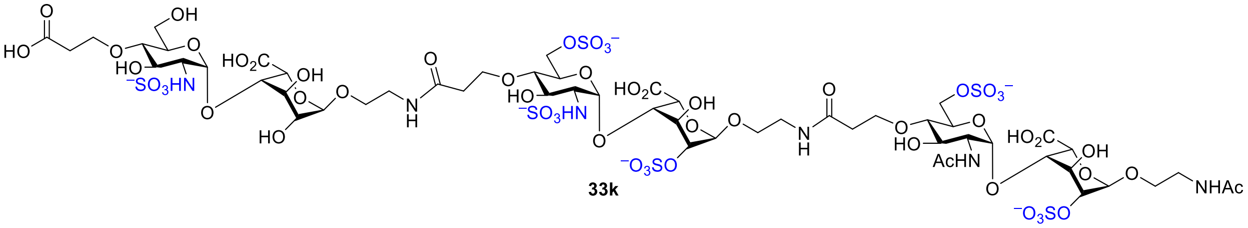

33k

33k. The starting material (6.6 mg, 0.004 mmol) was treated according to the general procedures of NH-Fmoc deprotection, pseudo-hexasaccharide preparation with HBTU and saponification of methyl esters to give compound **33k** (5.0 mg, 59% for 3 steps). $[\alpha]_D^{20}$ +36.3° (c 0.3, D_2O); ^1H NMR (500 MHz, D_2O) δ 5.17 – 5.13 (m, 2H; A-1, C-1), 4.99 (s, 1H; E-1), 4.94 – 4.90 (m, 2H; D-1, B-1), 4.72 (s, 1H; F-1), 4.45 – 4.36 (m, 3H), 4.13 – 3.99 (m, 8H), 3.95 (t, *J* = 3.7 Hz, 1H), 3.91 – 3.70 (m, 12H), 3.70 – 3.60 (m, 5H), 3.60 – 3.43 (m, 8H), 3.36 – 3.17 (m, 8H), 3.12 – 2.96 (m, 3H), 2.44 – 2.34 (m, 6H), 1.88 (s, 3H), 1.82 (s, 3H). ^{13}C NMR (125 MHz, D_2O) δ 174.62, 173.88, 100.60, 98.77, 98.68, 95.61, 93.54, 77.84, 77.68, 76.04, 74.81, 74.70, 74.23, 73.63, 70.82, 70.76, 70.56, 70.49, 68.88, 68.80, 68.58, 68.40, 68.20, 68.00, 67.72, 67.32, 66.88, 66.81, 66.53, 66.25, 66.14, 63.77, 59.70, 57.82, 57.69, 53.00, 39.00, 38.88, 36.33, 36.17, 22.07, 21.69. HRMS: m/z calc. for $C_{55}H_{84}N_6O_{57}S_6^{6-}$ $[M+Na^+]^{5-}$: 391.0422; found: 391.0430.

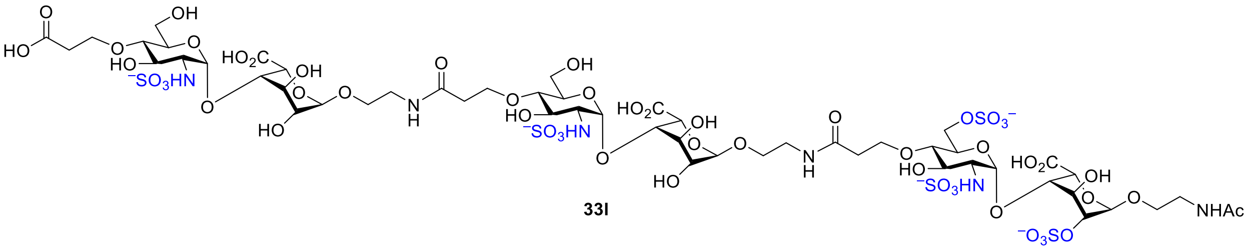

33l

33l. The starting material (2.5 mg, 0.002 mmol) was treated according to the general procedures of NH-Fmoc deprotection, pseudo-hexasaccharide preparation with HBTU and saponification of

methyl esters to give compound **33l** (1.4 mg, 42% for 3 steps). $[\alpha]_D^{20}$ +56º (c 0.05, D_2O); ^1H NMR (500 MHz, D_2O) δ 5.22 (d, *J* = 3.5 Hz, 1H; A-1), 5.19 – 5.06 (m, 2H; C-1, E-1), 4.94 (s, 1H; B-1), 4.77 – 4.71 (m, 2H; D-1, F-1), 4.38 – 4.30 (m, 3H), 4.13 – 4.07 (m, 2H), 4.05 – 3.99 (m, 2H), 3.95 (t, *J* = 3.9 Hz, 2H), 3.93 – 3.83 (m, 6H), 3.82 – 3.71 (m, 4H), 3.70 – 3.41 (m, 16H), 3.34 – 3.15 (m, 9H), 3.11 – 3.01 (m, 4H), 2.44 – 2.31 (m, 6H), 1.82 (s, 3H). HRMS: m/z calc. for $C_{53}H_{83}N_6O_{53}S_5^{5-}$ $[M+Na+]^{4-}$: 458.5627; found: 458.5623.

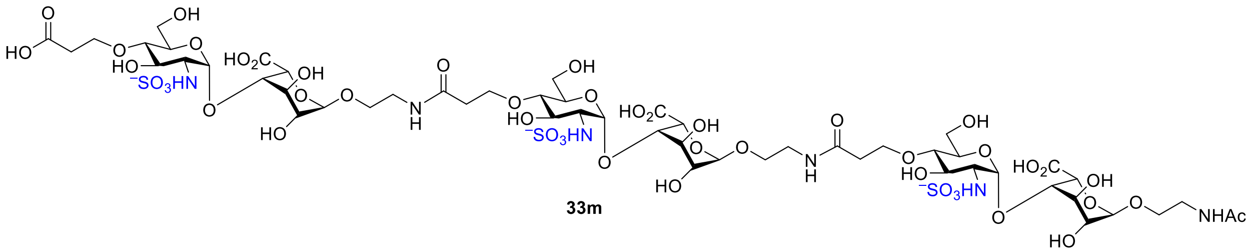

33m

33m. The starting material (3.0 mg, 0.002 mmol) was treated according to the general procedures of NH-Fmoc deprotection, pseudo-hexasaccharides preparation with HATU and saponification of methyl esters to give compound **33m** (0.8 mg, 22% for 3 steps). $[\alpha]_D^{20}$ +32.5º (c 0.08, D_2O); ^1H NMR (500 MHz, D_2O) δ 5.15 (d, *J* = 3.6 Hz, 3H; A-1, C-1, E-1), 4.73 (d, *J* = 4.1 Hz, 3H; B-1, D-1, F-1), 4.38 – 4.28 (m, 3H), 3.99 – 3.93 (m, 3H), 3.91 – 3.82 (m, 6H), 3.80 – 3.73 (m, 4H), 3.70 – 3.44 (m, 20H), 3.33 – 3.17 (m, 9H), 3.11 – 2.99 (m, 4H), 2.46 – 2.33 (m, 6H), 1.82 (s, 2H). ^{13}C NMR (151 MHz, D_2O, from HSQC) δ 100.70, 95.71, 78.14, 74.60, 74.52, 70.81, 70.73, 70.65, 69.60, 68.39, 68.23, 68.07, 67.91, 67.75, 66.78, 66.62, 59.93, 57.84, 39.07, 37.94, 36.33, 21.83. HRMS: m/z calc. for $C_{53}H_{85}N_6O_{47}S_3^{3-}$ $[M]^{3-}$: 551.1208; found: 551.1201.

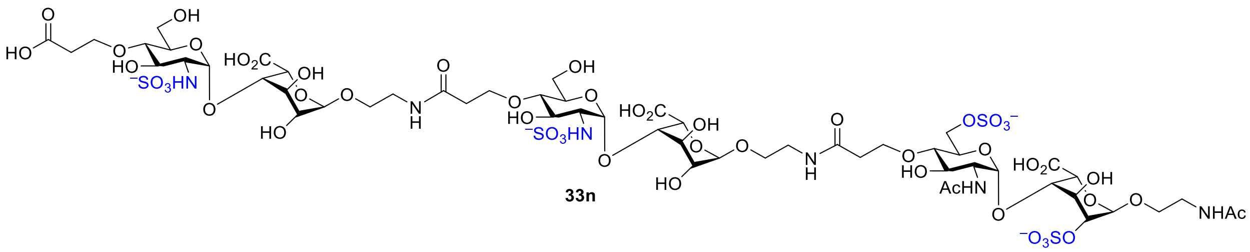

33n

33n. The starting material (3.8 mg, 0.003 mmol) was treated according to the general procedures of NH-Fmoc deprotection, pseudo-hexasaccharide preparation with HATU and saponification of methyl esters to give compound **33n** (2.6 mg, 52% for 3 steps). $[\alpha]_D^{20}$ +32.7º (c 0.11, D_2O); ^1H NMR (500 MHz, D_2O) δ 5.19 – 5.05 (m, 2H; A-1, C-1), 4.95 – 4.91 (m, 2H; E-1, B-1), 4.73 – 4.69 (m, 2H; D-1, F-1), 4.43 – 4.35 (m, 3H), 4.12 – 4.01 (m, 4H), 3.95 (d, *J* = 4.0 Hz, 2H), 3.92 – 3.72 (m, 11H),

3.70 – 3.42 (m, 17H), 3.32 – 3.19 (m, 8H), 3.08 – 2.98 (m, 3H), 2.47 – 2.33 (m, 6H), 1.88 (s, 3H), 1.81 (s, 3H). ^{13}C NMR (125 MHz, D_2O) δ 174.62, 173.88, 100.61, 98.65, 95.67, 95.59, 93.61, 77.90, 77.86, 77.75, 74.30, 74.27, 73.59, 70.85, 70.75, 70.71, 70.55, 68.87, 68.56, 68.18, 68.08, 67.37, 67.28, 66.88, 66.52, 63.77, 59.72, 57.77, 57.70, 53.04, 39.00, 38.89, 36.29, 36.23, 22.07, 21.70. HRMS: m/z calc. for $C_{55}H_{86}N_6O_{51}S_4^{4-}$ $[M]^{4-}$: 443.5806; found: 443.5810.

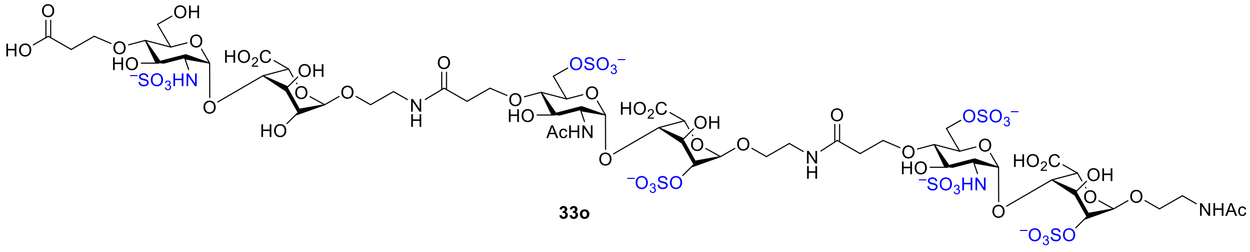

33o

33o. The starting material (3.9 mg, 0.003 mmol) was treated according to the general procedures of NH-Fmoc deprotection, pseudo-hexasaccharide preparation with HATU and saponification of methyl esters to give compound **33o** (1.7 mg, 34% for 3 steps). $[\alpha]_D^{20}$ +30º (c 0.11, D_2O); ^{1}H NMR (500 MHz, D_2O) δ 5.21 (d, *J* = 3.7 Hz, 1H; A-1), 5.14 (d, *J* = 3.7 Hz, 1H; C-1), 4.97 – 4.91 (m, 3H; B-1, D-1, E-1), 4.73 (s, 1H; F-1), 4.48 – 4.36 (m, 3H), 4.14 – 3.99 (m, 8H), 3.98 – 3.71 (m, 12H), 3.70 – 3.44 (m, 13H), 3.36 – 3.15 (m, 9H), 3.14 – 3.00 (m, 3H), 2.47 – 2.33 (m, 6H), 1.88 (s, 3H), 1.82 (s, 3H). HRMS: m/z calc. for $C_{55}H_{84}N_6O_{57}S_6^{6-}$ $[M+2Na^+]^{4-}$: 494.5500; found: 494.5502.

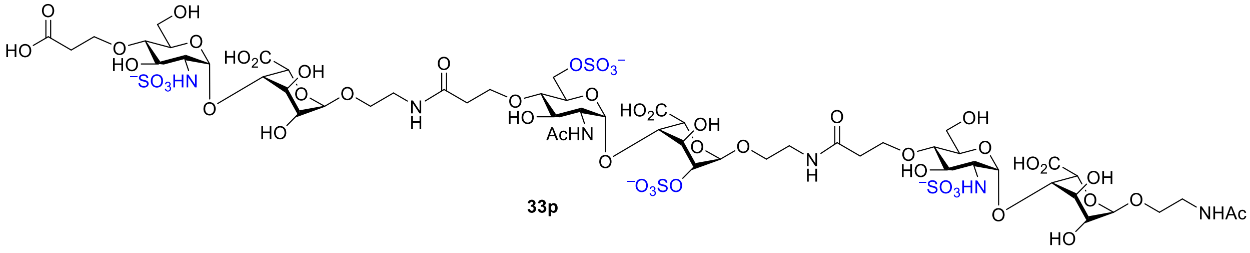

33p

33p. The starting material (7.1 mg, 0.005 mmol) was treated according to the general procedures of NH-Fmoc deprotection, pseudo-hexasaccharide preparation with HATU and saponification of methyl esters to give compound **33p** (4.4 mg, 51% for 3 steps). $[\alpha]_D^{20}$ +37.5º (c 0.24, D_2O); ^{1}H NMR (500 MHz, D_2O) δ 5.19 – 5.04 (m, 2H; A-1, C-1), 4.96 – 4.92 (m, 2H; B-1, E-1), 4.74 – 4.71 (m, 2H; D-1, F-1), 4.45 (d, *J* = 2.1 Hz, 1H), 4.40 (dd, *J* = 4.4, 2.5 Hz, 2H), 4.12 – 4.00 (m, 4H), 3.95 (d, *J* = 4.3 Hz, 2H), 3.91 – 3.73 (m, 10H), 3.71 – 3.44 (m, 17H), 3.38 – 3.17 (m, 9H), 3.07 – 3.01 (m, 3H), 2.47 – 2.36 (m, 6H), 1.89 (s, 3H), 1.81 (s, 3H). ^{13}C NMR (125 MHz, D_2O) δ 174.65, 174.47, 174.15, 173.94, 100.65, 98.65, 95.66, 93.79, 77.94, 77.78, 77.71, 74.37, 74.22, 73.35, 70.92, 70.82, 70.73,

70.67, 70.59, 68.92, 68.54, 68.17, 68.13, 67.98, 67.40, 67.27, 66.79, 66.75, 66.59, 66.19, 63.57, 59.73, 59.70, 57.72, 53.05, 39.04, 38.93, 36.28, 36.21, 35.40, 22.09, 21.69. HRMS: m/z calc. for $C_{55}H_{86}N_6O_{51}S_4^{4-}$ $[M]^{4-}$: 443.5806; found: 443.5805.

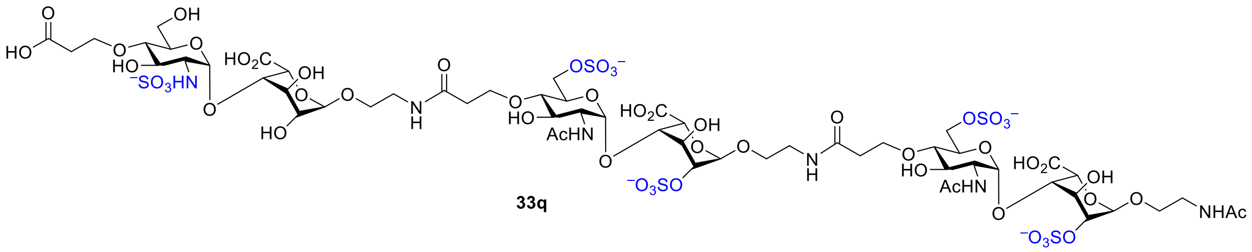

33q

33q. The starting material (6.9 mg, 0.005 mmol) was treated according to the general procedures of NH-Fmoc deprotection, pseudo-hexasaccharide preparation with HATU and saponification of methyl esters to give compound **33q** (4.0 mg, 45% for 3 steps). $[\boldsymbol{\alpha}]_{\mathbf{D}}^{\mathbf{20}}$ +20.0º (c 0.13, D_2O); ^{1}H NMR (500 MHz, D_2O) δ 7.99 – 7.88 (m, 3H, NHAc), 5.14 (d, *J* = 3.6 Hz, 1H; A-1), 4.96 – 4.91 (m, 4H; B-1, C-1, D-1, E-1), 4.39 (d, *J* = 2.4 Hz, 1H; F-1), 4.32 (s, 1H), 4.15 – 4.02 (m, 7H), 3.96 – 3.73 (m, 14H), 3.69 – 3.42 (m, 14H), 3.38 – 3.19 (m, 10H), 3.05 (dd, *J* = 10.4, 3.5 Hz, 2H), 2.46 – 2.31 (m, 6H), 1.89 (s, 6H), 1.83 (s, 3H). ^{13}C NMR (151 MHz, D_2O, from HSQC) δ 100.70, 98.69, 95.71, 93.61, 78.22, 77.90, 74.52, 73.87, 73.47, 70.89, 69.60, 68.96, 68.72, 68.31, 68.07, 67.67, 67.10, 66.86, 66.78, 66.70, 66.30, 64.04, 63.64, 59.93, 57.76, 53.17, 52.28, 39.07, 38.99, 37.94, 36.33, 22.23, 21.83. HRMS: m/z calc. for $C_{57}H_{87}N_6O_{55}S_5^{5-}$ $[M+Li^+]^{4-}$: 475.5745; found: 475.5755.

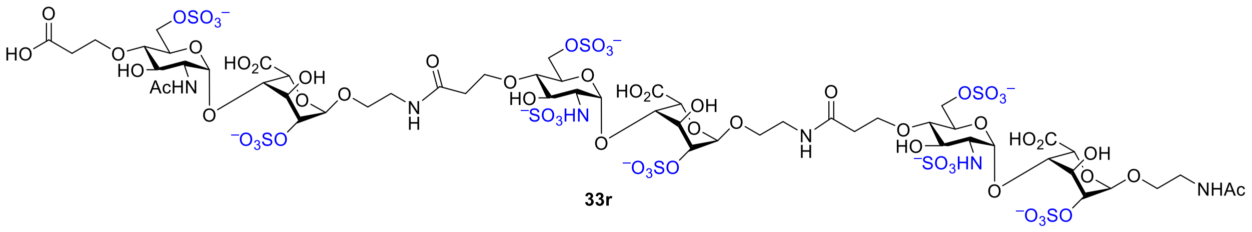

33r

33r. The starting material (5.5 mg, 0.003 mmol) was treated according to the general procedures of NH-Fmoc deprotection, pseudo-hexasaccharide preparation with HBTU and saponification of methyl esters to give compound **33r** (2.4 mg, 34% for 3 steps). $[\boldsymbol{\alpha}]_{\mathbf{D}}^{\mathbf{20}}$ +47.5º (c 0.16, D_2O); ^{1}H NMR (500 MHz, D_2O) δ 5.22 (d, *J* = 3.3 Hz, 1H; A-1), 5.16 (d, *J* = 3.6 Hz, 1H; C-1), 5.00 (s, 2H; E-1), 4.96 – 4.89 (m, 3H; B-1, D-1, F-1), 4.48 – 4.39 (m, 3H), 4.19 – 3.98 (m, 11H), 3.93 – 3.45 (m, 21H), 3.34 – 3.04 (m, 12H), 2.52 – 2.33 (m, 6H), 1.89 (s, 3H), 1.82 (s, 3H). ^{13}C NMR (125 MHz, D_2O) δ 173.79, 171.71, 109.99, 99.08, 98.56, 97.59, 93.80, 91.93, 91.61, 77.81, 77.76, 77.61, 76.07, 75.96, 74.67, 74.22, 73.18, 72.85, 70.86, 70.79, 70.49, 68.91, 68.78, 68.37, 67.62, 66.93, 66.13, 57.72,

53.00, 45.75, 38.87, 57.72, 39.05, 38.87, 36.23, 22.10, 21.70. HRMS: m/z calc. for $C_{55}H_{82}N_6O_{63}S_8^{8-}$ $[M+3Na^+]^{5-}$: 431.8177; found: 431.8196.

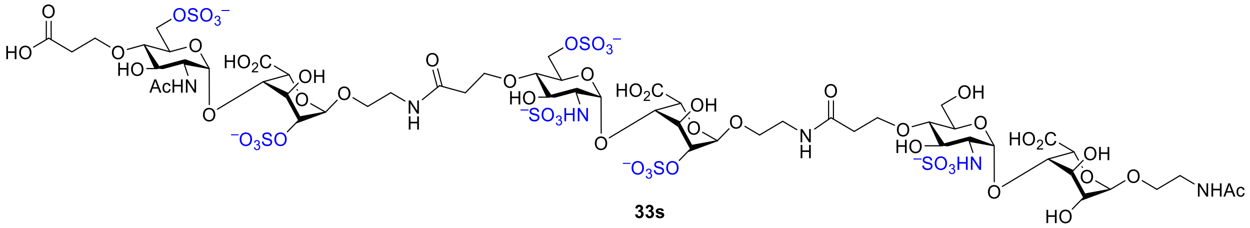

33s

33s. The starting material (8.2 mg, 0.005 mmol) was treated according to the general procedures of NH-Fmoc deprotection, pseudo-hexasaccharide preparation with HBTU and saponification of methyl esters to give compound **33s** (5.0 mg, 51% for 3 steps). $[\alpha]_D^{20}$ +130º (c 0.05, D_2O); ^1H NMR (500 MHz, D_2O) δ 5.19 (d, *J* = 3.6 Hz, 1H; A-1), 5.14 (d, *J* = 3.7 Hz, 1H; C-1), 4.98 (d, *J* = 2.1 Hz, 1H; E-1), 4.96 – 4.91 (m, 2H; B-1, D-1), 4.73 – 4.70 (m, 1H; F-1), 4.43 (d, *J* = 2.0 Hz, 2H), 4.37 (d, *J* = 2.4 Hz, 1H), 4.19 – 3.99 (m, 9H), 3.96 (t, *J* = 3.9 Hz, 2H), 3.92 – 3.44 (m, 22H), 3.35 – 3.16 (m, 9H), 3.12 – 3.02 (m, 3H), 2.50 – 2.33 (m, 6H), 1.89 (s, 3H), 1.81 (s, 3H). ^{13}C NMR (125 MHz, D_2O) δ 174.66, 174.16, 173.87, 100.63, 98.91, 98.56, 97.22, 95.62, 93.76, 78.01, 77.77, 77.61, 76.01, 75.29, 74.41, 73.18, 70.83, 70.79, 70.67, 70.56, 68.90, 68.81, 68.41, 68.24, 68.17, 68.09, 67.72, 67.48, 66.81, 66.73, 66.16, 66.13, 63.42, 59.74, 57.77, 57.72, 52.99, 39.05, 38.97, 38.87, 36.25, 36.18, 35.51, 22.09, 21.70. HRMS: m/z calc. for $C_{55}H_{84}N_6O_{57}S_6^{6-}$ $[M+2Na^+]^{4-}$: 494.5500; found: 494.5519.

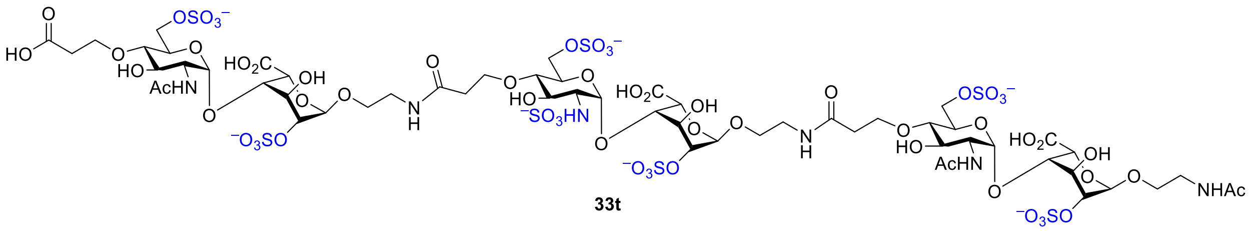

33t

33t. The starting material (7.1 mg, 0.004 mmol) was treated according to the general procedures of NH-Fmoc deprotection, pseudo-hexasaccharide preparation with HBTU and saponification of methyl esters to give compound **33t** (4.5 mg, 50% for 3 steps). $[\alpha]_D^{20}$ +34.6º (c 0.28, D_2O); ^1H NMR (500 MHz, D_2O) δ 5.17 (d, *J* = 3.5 Hz, 1H; A-1), 4.99 (s, 1H; C-1), 4.97 – 4.90 (m, 4H; B-1, D-1, E-1, F-1), 4.47 – 4.40 (m, 3H), 4.16 (dd, *J* = 11.1, 2.7 Hz, 1H), 4.13 – 3.99 (m, 10H), 3.95 – 3.42 (m, 23H), 3.35 – 3.18 (m, 9H), 3.09 (dd, *J* = 10.5, 3.5 Hz, 2H), 2.53 – 2.32 (m, 6H), 1.88 (s, 6H), 1.82 (s, 3H). ^{13}C NMR (125 MHz, D_2O) δ 199.58, 177.66, 174.62, 174.16, 173.84, 98.77, 98.68, 98.56,

97.48, 93.80, 93.57, 77.84, 77.75, 77.61, 76.04, 74.83, 73.63, 73.17, 70.85, 70.78, 70.46, 68.90, 68.84, 68.58, 68.44, 68.36, 67.74, 66.89, 66.83, 66.75, 66.26, 66.13, 63.79, 63.43, 57.78, 53.00, 39.00, 38.93, 36.24, 36.18, 35.43, 22.09, 21.70. HRMS: m/z calc. for $C_{57}H_{85}N_6O_{61}S_7^{7-}$ $[M+2Na^+]^{5-}$: 419.8320; found: 419.8333.

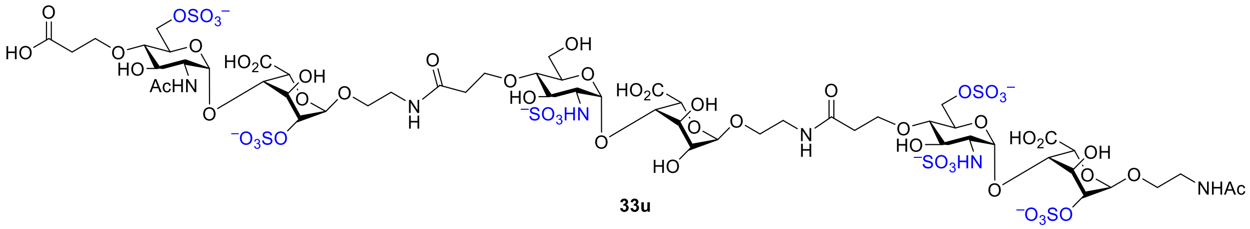

33u

33u. The starting material (6.8 mg, 0.005 mmol) was treated according to the general procedures of NH-Fmoc deprotection, pseudo-hexasaccharide preparation with HBTU and saponification of methyl esters to give compound **33u** (4.0 mg, 45% for 3 steps). $[\alpha]_D^{20}$ +32.3º (c 0.31, D_2O); ^{1}H NMR (500 MHz, D_2O) δ 5.21 (d, *J* = 3.5 Hz, 1H; A-1), 5.14 (d, *J* = 3.7 Hz, 1H; C-1), 4.96 – 4.91 (m, 3H; E-1, B-1, D-1), 4.75 – 4.71 (m, 1H; F-1), 4.39 (dd, *J* = 13.1, 2.4 Hz, 3H), 4.20 – 3.99 (m, 8H), 3.96 (t, *J* = 3.8 Hz, 1H), 3.93 – 3.73 (m, 10H), 3.70 – 3.43 (m, 14H), 3.39 – 3.16 (m, 9H), 3.09 – 3.02 (m, 3H), 2.49 – 2.33 (m, 6H), 1.89 (s, 3H), 1.82 (s, 3H). ^{13}C NMR (125 MHz, D_2O) δ 174.64, 173.89, 99.03, 98.60, 96.80, 95.65, 93.68, 78.02, 77.74, 77.66, 75.95, 74.31, 73.36, 70.84, 70.57, 68.86, 68.72, 68.57, 68.41, 68.19, 66.89, 66.74, 66.48, 66.15, 63.58, 59.74, 57.78, 57.74, 52.97, 39.05, 36.35, 35.78, 22.09, 21.69. HRMS: m/z calc. for $C_{55}H_{84}N_6O_{57}S_6^{6-}$ $[M+Na^+]^{5-}$: 391.0422; found: 391.0437.

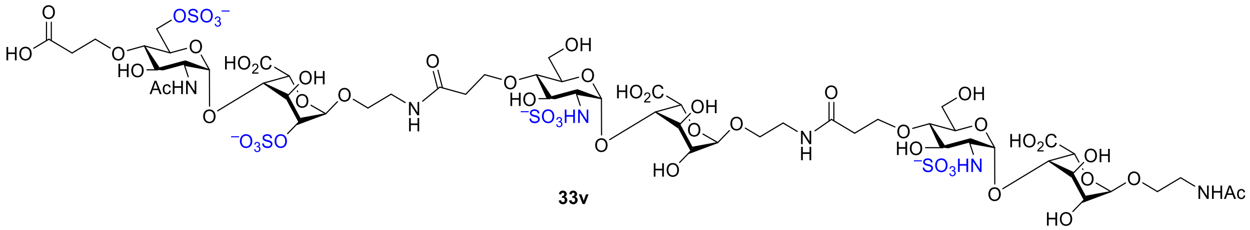

33v

33v. The starting material (7.3 mg, 0.005 mmol) was treated according to the general procedures of NH-Fmoc deprotection, pseudo-hexasaccharide preparation with HBTU and saponification of methyl esters to give compound **33v** (4.1 mg, 46% for 3 steps). $[\alpha]_D^{20}$ +33.3º (c 0.21, D_2O); ^{1}H NMR (500 MHz, D_2O) δ 5.14 (s, 2H; A-1, C-1), 4.93 (s, 2H; B-1, E-1), 4.75 – 4.70 (m, 2H; D-1, F-1), 4.43 – 4.30 (m, 3H), 4.20 – 4.02 (m, 5H), 3.95 (q, *J* = 4.1 Hz, 2H), 3.91 – 3.71 (m, 11H), 3.69 – 3.42 (m, 16H), 3.33 – 3.13 (m, 9H), 3.04 (dd, *J* = 10.5, 3.7 Hz, 2H), 2.48 – 2.30 (m, 6H), 1.88 (s, 3H), 1.81 (s,

3H). ^{13}C NMR (125 MHz, D_2O) δ 174.63, 173.93, 100.60, 98.60, 95.63, 95.58, 93.67, 78.00, 77.94, 77.65, 74.42, 74.33, 73.36, 70.79, 70.69, 70.50, 68.86, 68.54, 68.23, 68.10, 67.52, 66.69, 66.51, 66.14, 63.57, 59.74, 57.77, 57.74, 52.96, 39.05, 38.95, 36.25, 36.17, 22.08, 21.70. HRMS: m/z calc. for $C_{55}H_{86}N_6O_{51}S_4^{4-}$ $[M]^{4-}$: 443.5806; found: 443.5793.

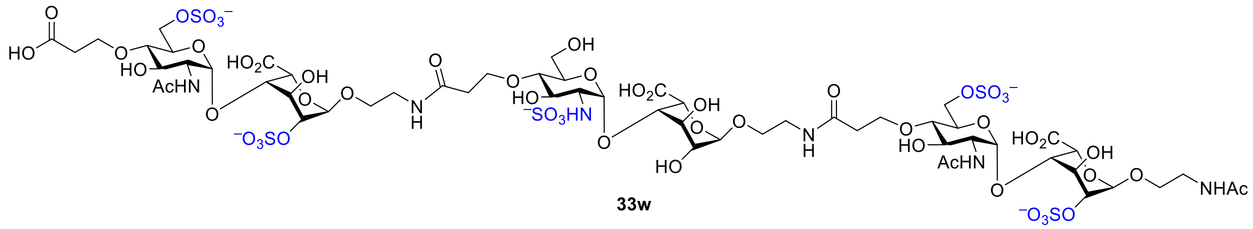

33w

33w. The starting material (7.0 mg, 0.005 mmol) was treated according to the general procedures of NH-Fmoc deprotection, pseudo-hexasaccharide preparation with HBTU and saponification of methyl esters to give compound **33w** (4.5 mg, 50% for 3 steps). $[\alpha]_D^{20}$ +29° (c 0.21, D_2O); ^1H NMR (500 MHz, D_2O) δ 5.14 (d, *J* = 3.7 Hz, 1H; A-1), 4.95 – 4.90 (m, 4H; B-1, C-1, D-1, E-1), 4.75 – 4.71 (m, 1H; F-1), 4.42 – 4.35 (m, 3H), 4.18 – 4.01 (m, 8H), 3.96 (t, *J* = 3.7 Hz, 1H), 3.92 – 3.73 (m, 13H), 3.70 – 3.44 (m, 12H), 3.37 – 3.16 (m, 10H), 3.04 (dd, *J* = 10.4, 3.6 Hz, 1H), 2.51 – 2.30 (m, 6H), 1.88 (s, 6H), 1.82 (s, 3H). ^{13}C NMR (125 MHz, D_2O) δ 174.63, 173.87, 100.56, 98.65, 98.60, 95.66, 93.68, 93.56, 78.00, 77.75, 77.65, 74.32, 73.61, 73.35, 70.84, 70.80, 70.72, 70.58, 68.85, 68.51, 68.16, 66.86, 66.74, 66.49, 66.14, 63.78, 63.57, 59.73, 57.74, 53.04, 52.97, 39.00, 36.29, 36.16, 35.67, 22.07, 21.70. HRMS: m/z calc. for $C_{57}H_{87}N_6O_{55}S_5^{5-}$ $[M]^{5-}$: 379.0565; found: 379.0584.

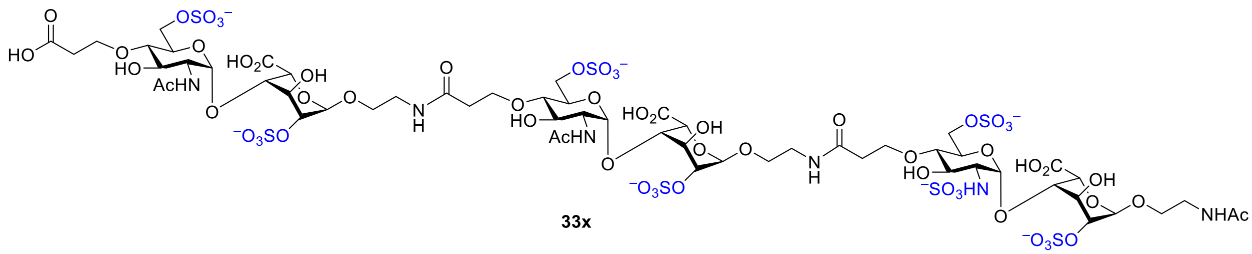

33x

33x. The starting material (5.3 mg, 0.003 mmol) was treated according to the general procedures of NH-Fmoc deprotection, pseudo-hexasaccharide preparation with HBTU and saponification of methyl esters to give compound **33x** (1.0 mg, 15% for 3 steps). $[\alpha]_D^{20}$ +20.0° (c 0.21, D_2O); ^1H NMR (500 MHz, D_2O) δ 5.24 (d, *J* = 3.6 Hz, 1H; A-1), 4.92 (d, *J* = 3.4 Hz, 4H; C-1, B-1, D-1, E-1), 4.40 – 4.32 (m, 4H), 4.16 – 3.97 (m, 10H), 3.95 – 3.73 (m, 12H), 3.70 – 3.43 (m, 11H), 3.39 – 3.13 (m, 12H), 2.42 – 2.31 (m, 6H), 1.85 (m, 9H). ^{13}C NMR (151 MHz, D_2O, from HSQC) δ 100.62, 99.01, 98.77, 96.91, 95.71, 93.61, 78.22, 77.98, 75.97, 75.81, 74.60, 73.87, 70.97, 70.81, 70.17, 69.60,

68.96, 68.64, 68.55, 68.39, 68.31, 68.07, 67.67, 67.10, 66.94, 66.86, 66.78, 66.70, 66.30, 64.04, 59.93, 57.84, 57.76, 53.17, 39.07, 38.99, 37.70, 36.33, 34.56, 28.67, 21.83, 22.15. HRMS: m/z calc. for $C_{57}H_{85}N_6O_{61}S_7^{7-}$ $[M+3Na^+]^{4-}$: 530.5373; found: 530.5389.

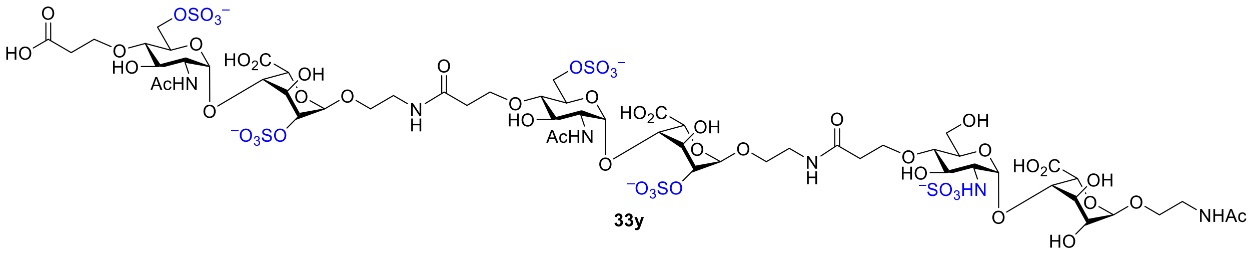

33y. The starting material (6.7 mg, 0.004 mmol) was treated according to the general procedures of NH-Fmoc deprotection, pseudo-hexasaccharide preparation with HBTU and saponification of methyl esters to give compound **33y** (4.4 mg, 55% for 3 steps). $[\alpha]_D^{20}$ +31.3º (c 0.32, D_2O); ^{1}H NMR (500 MHz, D_2O) δ 5.13 (d, *J* = 3.8 Hz, 1H; A-1), 4.92 (s, 4H; B-1, C-1, E-1), 4.73 – 4.69 (m, 1H; D-1), 4.46 – 4.32 (m, 3H), 4.17 – 3.99 (m, 9H), 3.94 (t, *J* = 3.9 Hz, 1H), 3.91 – 3.70 (m, 12H), 3.69 – 3.43 (m, 12H), 3.34 – 3.14 (m, 9H), 3.03 (dd, *J* = 10.5, 3.6 Hz, 2H), 2.50 – 2.31 (m, 6H), 1.87 (s, 6H), 1.80 (s, 3H). ^{13}C NMR (125 MHz, D_2O) δ 174.64, 173.91, 100.61, 98.63, 98.55, 95.61, 93.74, 93.65, 77.96, 77.78, 77.59, 74.38, 73.37, 73.20, 70.81, 70.66, 70.54, 68.89, 68.33, 68.20, 67.45, 66.73, 66.11, 63.53, 63.42, 59.71, 57.71, 52.98, 39.03, 38.92, 36.19, 35.39, 22.07, 21.68. HRMS: m/z calc. for $C_{57}H_{87}N_6O_{55}S_5^{5-}$ $[M]^{5-}$: 379.0565; found: 379.0574.

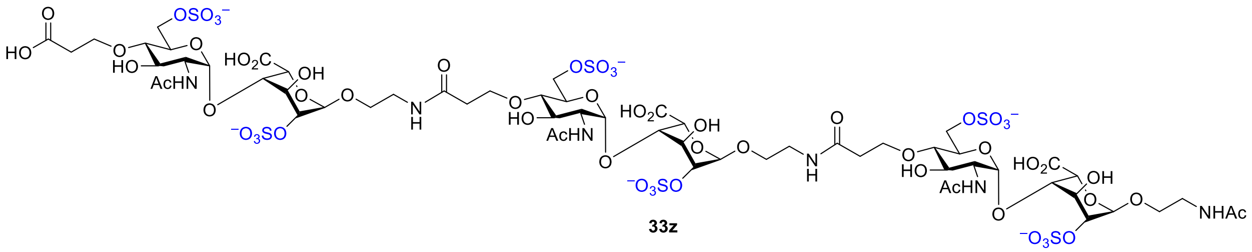

33z. The starting material (8.0 mg, 0.005 mmol) was treated according to the general procedures of NH-Fmoc deprotection, pseudo-hexasaccharide preparation with HBTU and saponification of methyl esters to give compound **33z** (4.5 mg, 44% for 3 steps). $[\alpha]_D^{20}$ +43.6º (c 0.11, D_2O); ^{1}H NMR (500 MHz, D_2O) δ 4.98 – 4.90 (m, 6H; A-1, B-1, C-1, D-1, E-1, F-1), 4.42 – 4.35 (m, 3H), 4.17 – 4.01 (m, 12H), 3.93 – 3.74 (m, 14H), 3.70 – 3.45 (m, 10H), 3.36 – 3.21 (m, 9H), 2.44 – 2.31 (m, 6H), 1.89 (s, 9H), 1.83 (s, 3H). ^{13}C NMR (151 MHz, D_2O, from HSQC) δ 98.36, 93.05, 77.58, 73.07, 70.65, 70.17, 68.55, 66.62, 66.46, 65.98, 65.82, 52.76, 38.75, 36.01, 21.83, 21.50. HRMS: m/z calc.

for $C_{59}H_{88}N_6O_{59}S_6^{6-}$ $[M]^{6-}$: 336.0405; found: 336.0412.

Measurements of binding constants of pseudo-hexasaccharides 33, 33z, and 33m with FGF-2 by SPR.

FGF-2 was immobilized on sensor chip by primary amine coupling reaction. The surface of a CDH sensor chip (FortéBio, Inc., Molecular Devices, USA) was activated using freshly mixed *N*-hydroxysuccimide (NHS; 0.05 M) and 1-(3-(dimethylamino)propyl)-ethylcarbodiimide (EDC; 0.2 M) (1/1,v/v) in water, followed by the injection of FGF-2 (50 µg/ml) in PBS buffer. The remaining active esters were quenched by ethanolamine (1.0 M). Solutions of various concentrations of pseudo-hexasaccharides **33**, **33z**, and **33m** dissolved in 0.01% Tween 20 PBS buffer were injected over the FGF-2 chip at a flow rate of 30 µL/min, and the responses to binding were recorded. Aqueous NaCl solution (2.0 M) was employed for regeneration to achieve baseline status after each run. Sensorgrams were evaluated using the Qdat program. The response data due to association and dissociation were processed in Qdat using a reference surface to correct for any bulk refractive index changes and blank injections for double referencing. The binding profiles were fit globally into a 1:2 interaction model. The K_D values were calculated by the Qdat software.

Analysis of the inhibition of pseudo-hexasaccharides on heparin/FGF-2 interaction using competition SPR

Heparin SPR chip was prepared by immobilizing biotinylated heparin on SA sensor chip (Cytiva, Uppsala, Sweden).[13] Samples of FGF-2 (50 nM) mixed with individual pseudo-hexasaccharides (1000 nM) respectively in HBS-EP buffer (0.01 M 4-(2-hydroxyethyl)-1-piperazineethanesulfonic acid, 0.15 M NaCl, 3 mM EDTA, 0.005% surfactant P20, pH 7.4) were injected over the heparin chip at a flow rate of 30 µL/min. After each run, the heparin sensor surface was regenerated by injecting with 30 µL of 2 M aqueous solution of NaCl. For each set of competition experiments, a control experiment (only injecting FGF-2) was performed to ensure the surface was completely regenerated. If the pseudo-hexasaccharide could bind with FGF-2, it should compete against the surface-immobilized heparin for FGF-2 binding resulting in a reduction in signal. Normalized FGF-2 binding % was calculated based on the following formula: (signal intensity of FGF-2/pseudohexasaccharide)/(signal intensity of FGF-2 only) x 100%. Each binding experiment was performed three times with the standard deviations calculated (all < 0.4%)

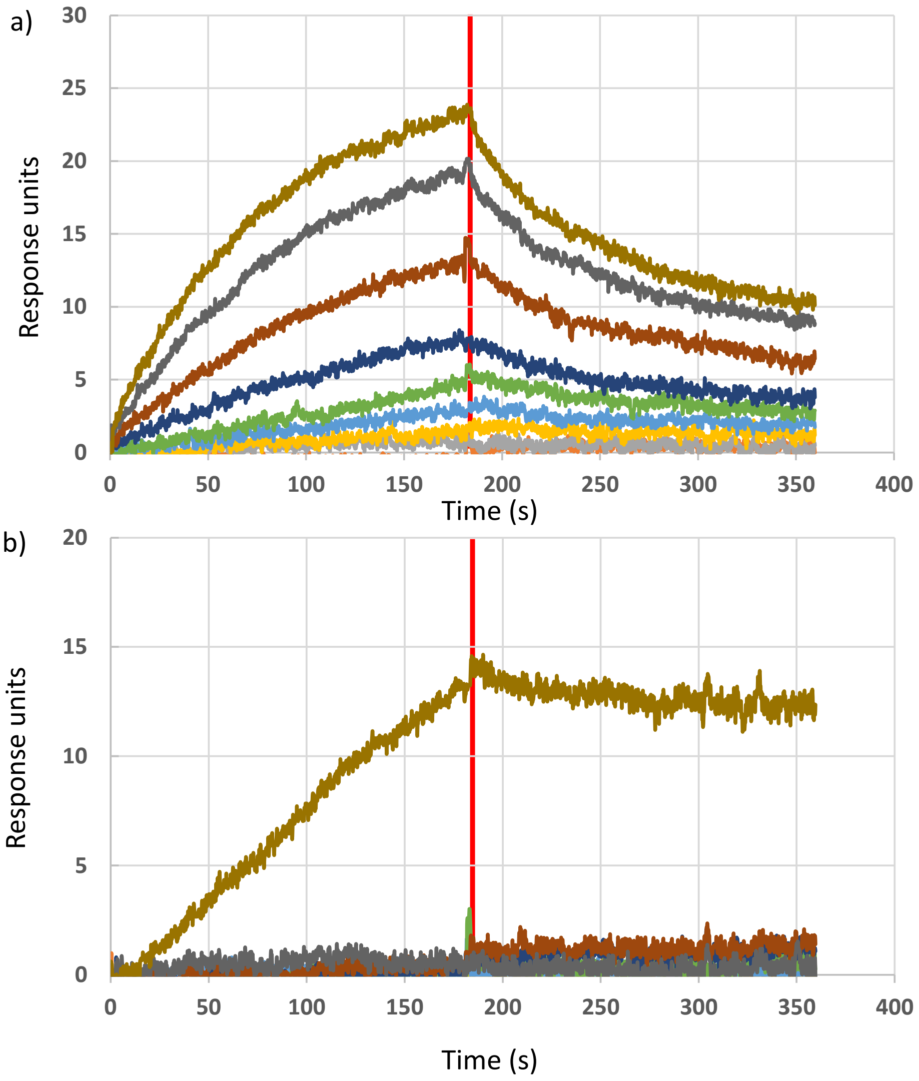

Figure S1. The SPR sensorgrams of FGF-2 binding with a) compound **33z**. The concentrations of **33z** from top to bottom curves were 4 µM, 3 µM, 2 µM, 750 nM, 500 nM, 250 nM, 100 nM, 10 nM, and 1 nM; b) compound **33m**. The concentrations of **33m** from top to bottom curves were 4 µM, 3 µM, 2 µM, 750 nM, 500 nM, 250 nM, 100 nM, 10 nM, and 1 nM. Each experiment was repeated at least three times with the representative data shown. The red vertical line indicates the time the dissociation process started.

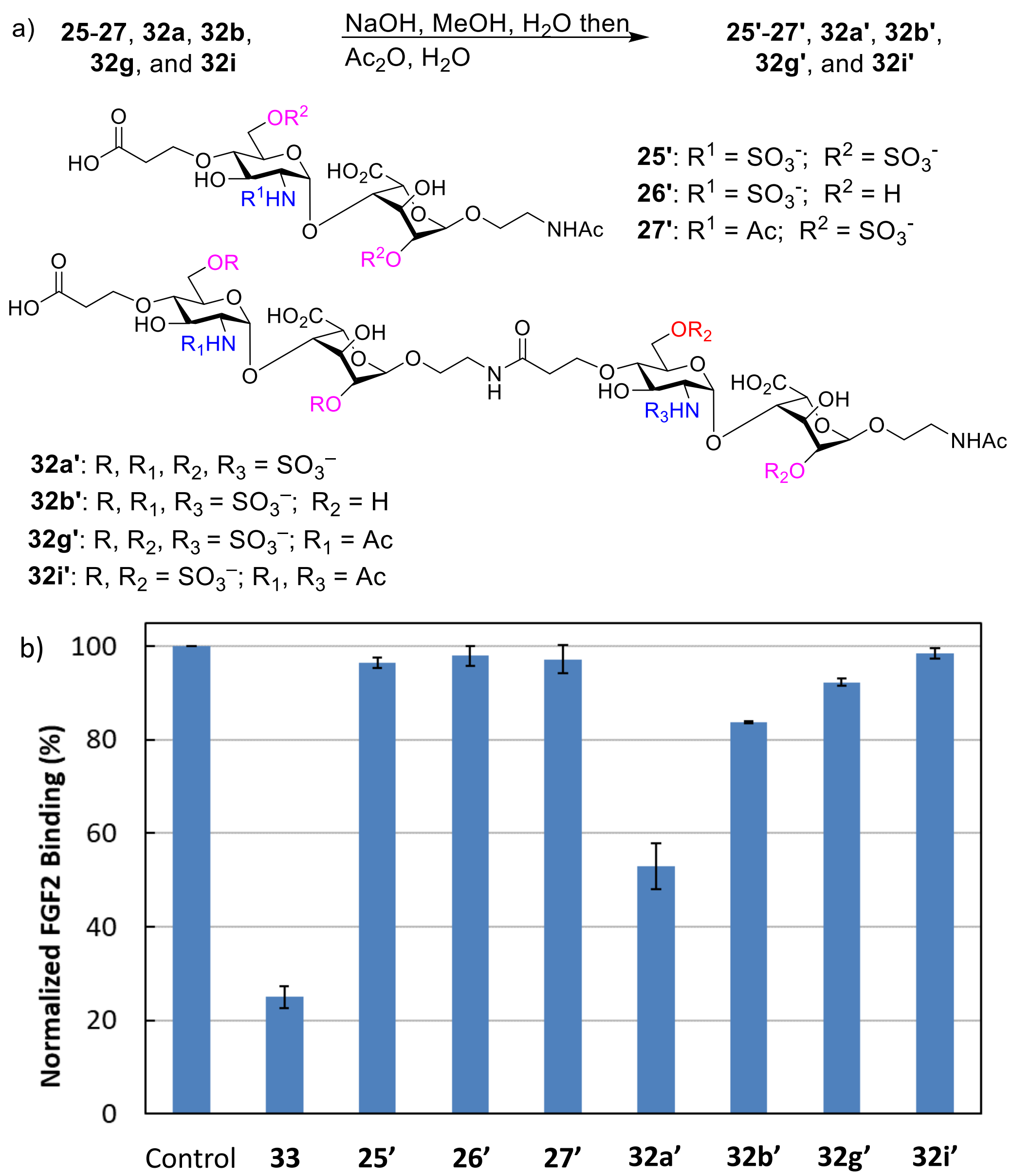

Figure S2. a) Preparation and structures of disaccharides **25**’ – **27**’ and **32a**’, **32b**’, **32g**’, and **32i**’. b) Inhibition of disaccharides **25**’-**27**’ and representative pseudo-tetrasaccharides **32a**’, **32b**’, **32g**’, and **32i**’ on FGF-2 interaction with heparin through a competition SPR assay. Biotinylated heparin was immobilized on the SPR biosensor. For the control well, FGF-2 (50 nM) was flown over the sensor and the intensity of the signal due to FGF-2 binding was set as a reference (control) (100%). The disaccharides and pseudo-hexasaccharides (100 μM) were mixed with FGF-2 (50 nM) individually, and each solution was flown over the sensor respectively. Pseudo-hexasaccharide **33** was measured for comparison. Normalized FGF-2 binding % was calculated based on the following formula: (signal intensity of FGF-2/HS mimetics)/(signal intensity of FGF-2 only) x 100%. The trend of increasing inhibition from **25’**, **32a’**, to **33** (bearing the same sulfation patterns on the disaccharide units) suggested longer mimetics have stronger binding to FGF-2, similar to that for HS di-, tetra-, and hexa-saccharides with native glycosyl linkages.

To a solution of **25** (7.1 mg, 9.7 μmol), **26** (0.23 mg, 0.4 μmol), **27** (5.2 mg, 7.5 μmol), **32a** (0.5 mg, 0.3 μmol), **32b** (1.5 mg, 1 μmol), **32g** (9.8 mg, 6.6 μmol), or **32i** (2 mg, 1.4 μmol) respectively in MeOH (1 mL), NaOH (1N, 0.2 mL) was added and the reaction was stirred and monitored by mass spectrometry. Upon completion, Amberlite H^+ resin was added until pH ~ 7 and the mixture was filtered and concentrated. The crude reaction mixtures were treated with Ac_2O/MeOH/Et_3N/H_2O (1/3/1/0.5, 1 mL). The mixtures were stirred at room temperature overnight, concentrated and then passed through G-10 gel column with H_2O to afford the desired compounds **25'-27'**, **32a'**, **32b'**, **32g'**, and **32i'**.

25' (6.9 mg, 94%): ^{1}H NMR (500 MHz, D_2O) δ 5.21 (d, *J* = 3.6 Hz, 1H), 4.96 (d, *J* = 2.7 Hz, 1H), 4.33 (d, *J* = 2.7 Hz, 1H), 4.14 (dd, *J* = 11.0, 2.3 Hz, 1H), 4.10 (dd, *J* = 5.4, 2.8 Hz, 1H), 4.05 – 4.02 (m, 2H), 3.89 (t, *J* = 3.1 Hz, 1H), 3.86 – 3.75 (m, 5H), 3.68 – 3.64 (m, 1H), 3.55 – 3.48 (m, 3H), 3.28 – 3.20 (m, 4H), 3.11 (dd, *J* = 10.6, 3.5 Hz, 1H), 2.37 – 2.33 (m, 2H), 1.82 (s, 3H). ^{13}C NMR (125 MHz, D_2O) δ 98.94, 96.69, 78.03, 75.79, 75.74, 70.56, 69.44, 68.76, 68.51, 68.41, 66.95, 66.90, 66.31, 57.57, 46.54, 39.06, 37.50, 21.78. HRMS: m/z calc. for $C_{19}H_{31}N_2O_{23}S_3^-$ $[M-H]^-$: 751.0485; found: 751.0600.

26' (0.2 mg, 85%): ^{1}H NMR (600 MHz, D_2O) δ 5.24 (d, *J* = 3.7 Hz, 1H), 4.41 (d, *J* = 2.4 Hz, 1H), 4.05 – 4.02 (m, 1H), 3.94 (d, *J* = 3.0 Hz, 1H), 3.93 – 3.89 (m, 2H), 3.87 (d, *J* = 12.1 Hz, 1H), 3.82 (dt, *J* = 9.6, 6.5 Hz, 3H), 3.73 – 3.69 (m, 4H), 3.63 – 3.55 (m, 9H), 3.50 – 3.46 (m, 3H), 3.32 – 3.27 (m, 3H), 3.15 (dd, *J* = 10.5, 3.7 Hz, 1H), 2.41 – 2.39 (m, 2H), 1.91 (s, 3H). ^{13}C NMR (150 MHz, D_2O) δ 96.11, 78.30, 75.71, 74.52, 70.73, 70.33, 69.68, 69.60, 68.39, 67.99, 67.67, 62.59, 62.43, 59.93, 57.68, 38.56, 37.94, 21.83. HRMS: m/z calc. for $C_{19}H_{31}N_2O_{17}S^-$ $[M-H]^-$: 591.1349; found: 591.1427.

27' (5 mg, 94%): ^{1}H NMR (600 MHz, D_2O) δ 5.04 – 5.01 (m, 2H), 4.47 (d, *J* = 2.2 Hz, 1H), 4.23 (dd, *J* = 11.0, 3.0 Hz, 1H), 4.20 – 4.14 (m, 3H), 3.97 – 3.87 (m, 6H), 3.75 – 3.67 (m, 3H), 3.62 – 3.56 (m, 2H), 3.51 – 3.45 (m, 1H), 3.39 – 3.32 (m, 3H), 2.44 – 2.39 (m, 2H), 1.98 (s, 3H), 1.92 (s, 3H). ^{13}C NMR (150 MHz, D_2O) δ 98.69, 93.93, 77.98, 73.79, 71.05, 70.81, 69.76, 68.88, 67.02, 66.94, 66.70, 66.30, 64.12, 52.93, 39.43, 37.86, 22.23, 21.83. HRMS: m/z calc. for $C_{21}H_{33}N_2O_{21}S_2^-$ $[M-H]^-$: 713.1023; found: 713.1149.

32a' (0.4 mg, 82%): ^{1}H NMR (600 MHz, D_2O) δ 5.34 – 5.26 (m, 2H), 5.04 (d, *J* = 20.7 Hz, 2H), 4.49 – 4.42 (m, 2H), 4.26 – 4.09 (m, 8H), 4.04 – 3.84 (m, 9H), 3.80 – 3.55 (m, 8H), 3.42 – 3.22 (m, 8H), 3.20 – 3.16 (m, 3H), 3.03 – 2.98 (m, 1H), 2.56 – 2.44 (m, 4H), 1.92 (s, 3H). ^{13}C NMR (150 MHz, D_2O) δ 99.33, 97.32, 96.67, 77.90, 76.69, 76.13, 75.48, 70.73, 69.28, 68.80, 68.55, 68.31, 66.94, 66.30, 57.92, 39.15, 36.33, 21.83. HRMS: m/z calc. for $C_{48}H_{88}N_6O_{44}S_6^{2-}$ $[M+2Et_3N-2H]^{-2}$: 822.6601; found: 822.6718.

32b' (1.2 mg, 92%): ^{1}H NMR (600 MHz, D_2O) δ 5.26 – 5.23 (m, 2H), 5.12 (s, 1H), 4.86 (s, 1H), 4.30 (t, *J* = 3.4 Hz, 1H), 4.26 – 4.21 (m, 2H), 4.13 – 4.09 (m, 2H), 4.04 – 4.02 (m, 1H), 4.01 – 3.91 (m, 5H), 3.87 – 3.81 (m, 2H), 3.77 – 3.72 (m, 3H), 3.70 – 3.58 (m, 9H), 3.53 – 3.49 (m, 1H), 3.41 – 3.30 (m, 8H), 3.19 (dt, *J* = 10.6, 2.9 Hz, 1H), 2.89 (s, 2H), 2.64 – 2.59 (m, 2H), 2.50 – 2.42 (m, 2H),

1.91 (s, 3H). ^{13}C NMR (150 MHz, D_2O) δ 100.94, 99.09, 96.19, 77.74, 77.58, 76.93, 74.27, 74.03, 71.05, 70.65, 69.20, 68.15, 67.99, 67.91, 67.83, 67.27, 67.10, 66.94, 66.78, 66.54, 66.22, 66.14, 59.77, 58.00, 57.76, 39.15, 39.15, 36.33, 34.80, 21.75. HRMS: m/z calc. for $C_{36}H_{58}N_4O_{38}S_4^{2-}$ [M-2H]$^{-2}$: 641.0811; found: 641.0916.

32g' (9 mg, 96%): ^{1}H NMR (600 MHz, D_2O) δ 5.26 (t, *J* = 4.0 Hz, 1H), 5.02 – 4.96 (m, 3H), 4.43 (d, *J* = 2.0 Hz, 1H), 4.39 (d, *J* = 2.8 Hz, 1H), 4.20 – 4.05 (m, 8H), 3.96 – 3.82 (m, 10H), 3.73 – 3.62 (m, 4H), 3.59 – 3.51 (m, 4H), 3.35 – 3.25 (m, 7H), 2.49 – 2.40 (m, 5H), 1.96 (s, 3H), 1.95 (s, 3H). ^{13}C NMR (150 MHz, D_2O) δ 99.09, 98.69, 96.83, 93.85, 78.06, 76.13, 76.05, 73.47, 71.05, 68.88, 68.72, 66.86, 66.30, 65.98, 63.88, 53.09, 38.77, 37.05, 36.41. HRMS: m/z calc. for $C_{38}H_{60}N_4O_{42}S_5^{2-}$ [M-2H]$^{-2}$: 702.0648; found: 702.0759.

32i' (1.7 mg, 90%): ^{1}H NMR (600 MHz, D_2O) δ 5.04 – 5.00 (m, 4H), 4.47 (t, *J* = 2.2 Hz, 1H), 4.25 – 4.12 (m, 9H), 3.96 – 3.93 (m, 5H), 3.91 – 3.86 (m, 6H), 3.75 – 3.66 (m, 9H), 3.61 – 3.55 (m, 7H), 3.49 – 3.46 (m, 3H), 3.38 – 3.32 (m, 7H), 2.51 – 2.47 (m, 2H), 2.44 – 2.39 (m, 2H), 1.98 (s, 6H), 1.92 (s, 3H). ^{13}C NMR (150 MHz, D_2O) δ 98.85, 93.69, 78.14, 73.63, 71.05, 70.89, 69.76, 68.88, 68.55, 66.94, 66.78, 66.30, 66.22, 63.96, 62.43, 53.01, 38.99, 37.86, 36.33, 22.23, 21.91. HRMS: m/z calc. for $C_{40}H_{62}N_4O_{40}S_4^{2-}$ [M-2H]$^{-2}$: 683.0917; found: 683.1003.

References:

[1] A. Hoang, E. Laigre, D. Goyard, E. Defrancq, F. Vinet, P. Dumy, O. Renaudet, *Org. Biomol. Chem.* **2017**, *15*, 5135-5139.

[2] S. Ulrich, D. Boturyn, A. Marra, O. Renaudet, P. Dumy, *Chem. Eur. J.* **2014**, *20*, 34-41.

[3] Y. Liu, T. Feizi, M. A. Campanero-Rhodes, R. A. Childs, Y. Zhang, B. Mulloy, P. G. Evans, H. M. I. Osborn, D. Otto, P. R. Crocker, W. Chai, *Chem. Biol.* **2007**, *14*, 847-859.

[4] A. Dirksen, T. M. Hackeng, P. E. Dawson, *Angew. Chem. Int. Ed.* **2006**, *45*, 7581-7584.

[5] Y. Takenaka, T. Kiyosu, J. C. Choi, T. Sakakura, H. Yasuda, *Green Chem.* **2009**, *11*, 1385–1390.

[6] F. Weygand, E. Frauendorfer, *Chem. Ber.* **1970**, *103*, 2437-2449.

[7] S. Gomez, J. A. Peter, T. Maschmeyer, *Adv. Synth. Catal.* **2002**, *344*, 1037-1057.

[8] A. F. Abdel-Magid, K. G. Carson, B. D. Harris, C. A. Maryanoff, R. D. Shah, *J. Org. Chem.* **1996**, *61*, 3849-3862.

[9] J. Revuelta, R. Fuentes, L. Lagartera, M. José Hernáiz, A. Bastida, E. García-Junceda, A. Fernández-Mayoralas, *Chem. Commun.* **2018**, *54*, 13455-13458.

[10] C. Yang, Y. Deng, Y. Wang, C. Xia, P. Booneimsri, C. Lertmaneedang, A. Y. Mehta, K. J. Baker, S. Hwang, J. P. Flynn, M. Cao, C. Liu, A. C. Zhu, R. D. Cummings, C. Lin, U. Mohanty, J. Niu, *ChemRxiv* **2022**, 10.26434/chemrxiv-22022-cfrfk.

[11] Z. Wang, Y. Xu, B. Yang, G. Tiruchinapally, B. Sun, R. Liu, S. Dulaney, J. Liu, X. Huang, *Chem. Eur. J.* **2010**, *16*, 8365-8375.

[12] K. Bock, C. Pedersen, *J. Chem. Soc., Perkin Trans. 2* **1974**, 293-297.

[13] S. Y. Kim, F. Zhang, W. Gong, K. Chen, K. Xia, F. Liu, R. Gross, J. M. Wang, R. J. Linhardt, M. L. Cotten, *J. Biol. Chem.* **2018**, *293*, 15381-15396.

Product Characterization Spectra

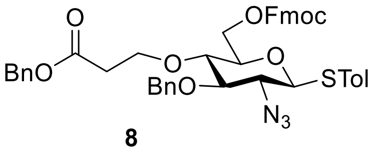

8

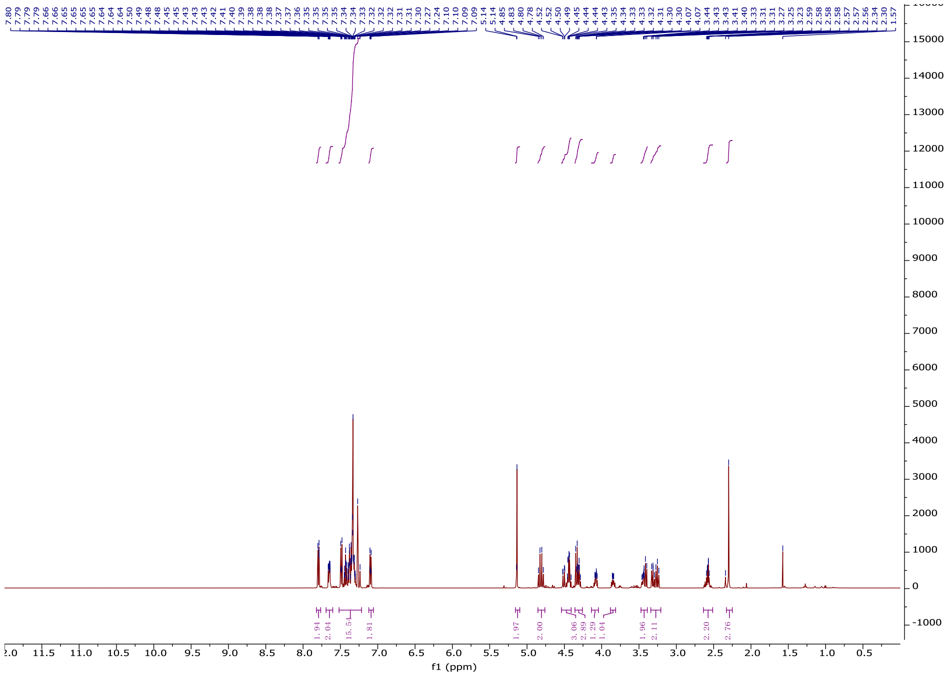

^{1}H-NMR of **8** (500 MHz $CDCl_3$)

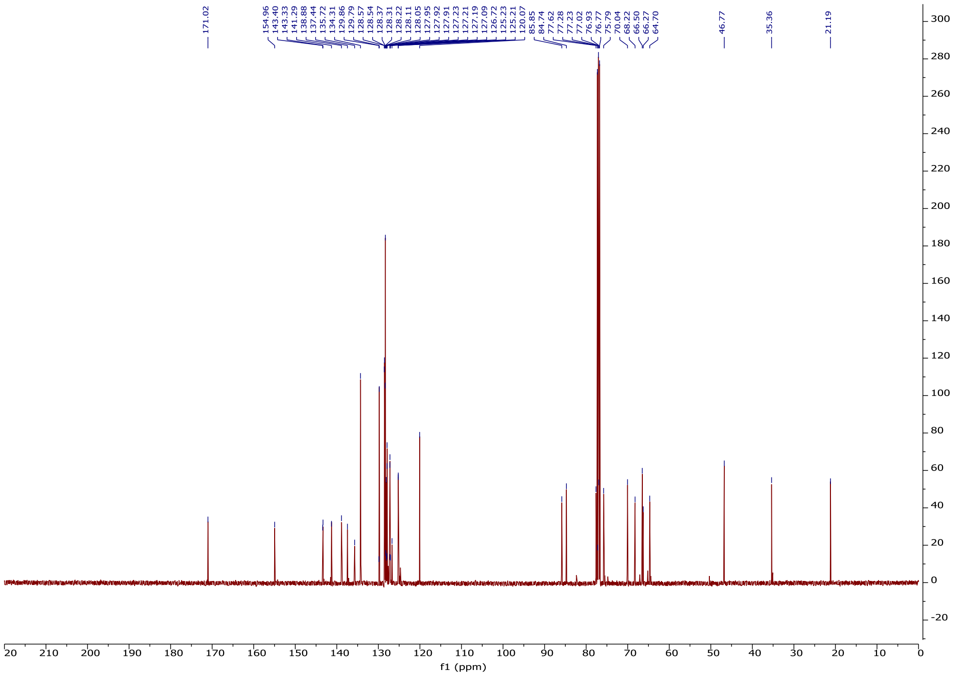

^{13}C-NMR of **8** (125 MHz $CDCl_3$)

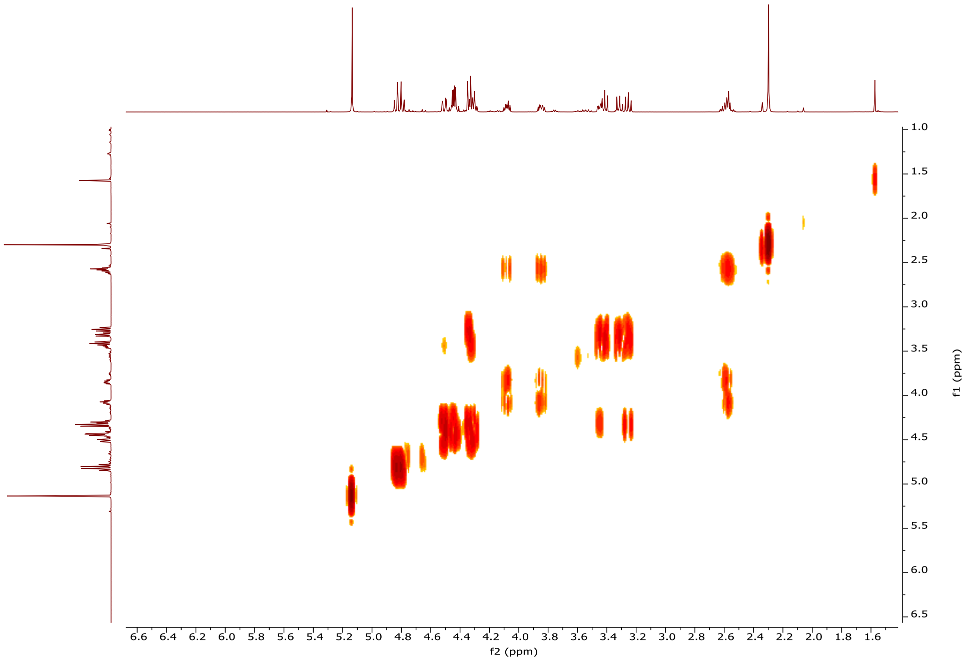

^{1}H-^{1}H gCOSY of **8** (500 MHz $CDCl_3$)

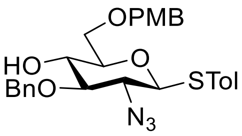

10

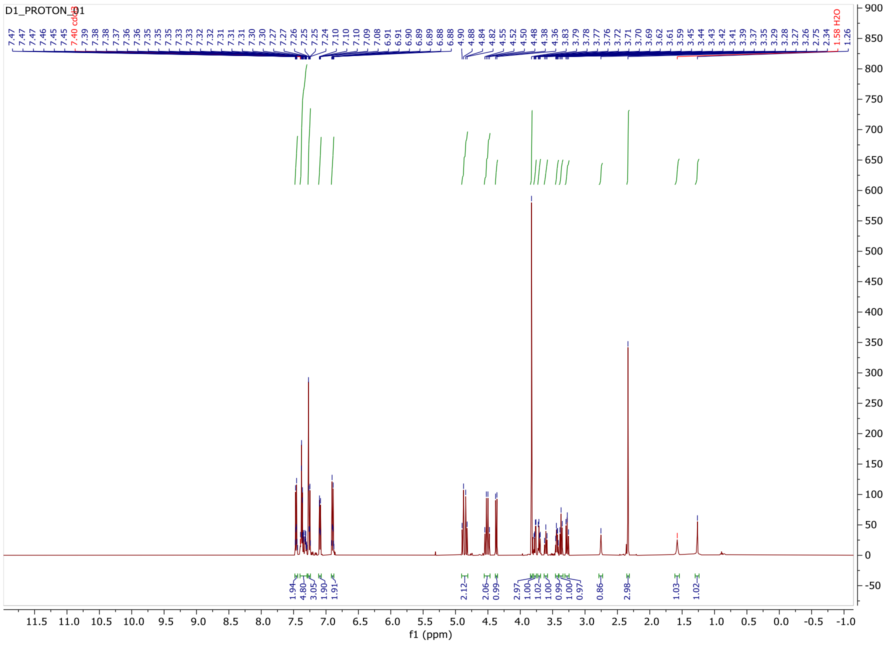

^{1}H-NMR of **10** (500 MHz $CDCl_3$)

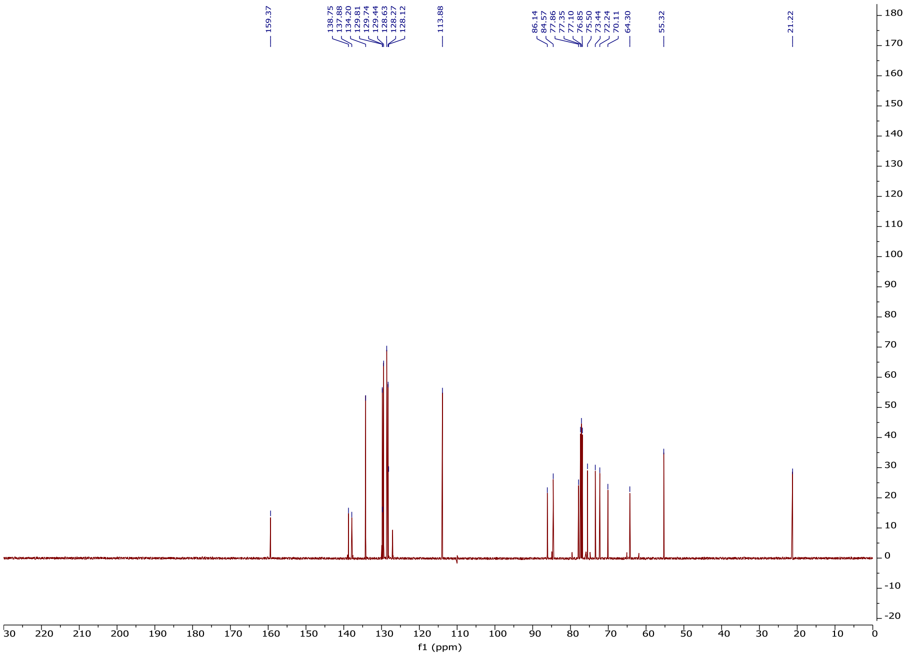

^{13}C-NMR of **10** (125 MHz $CDCl_3$)

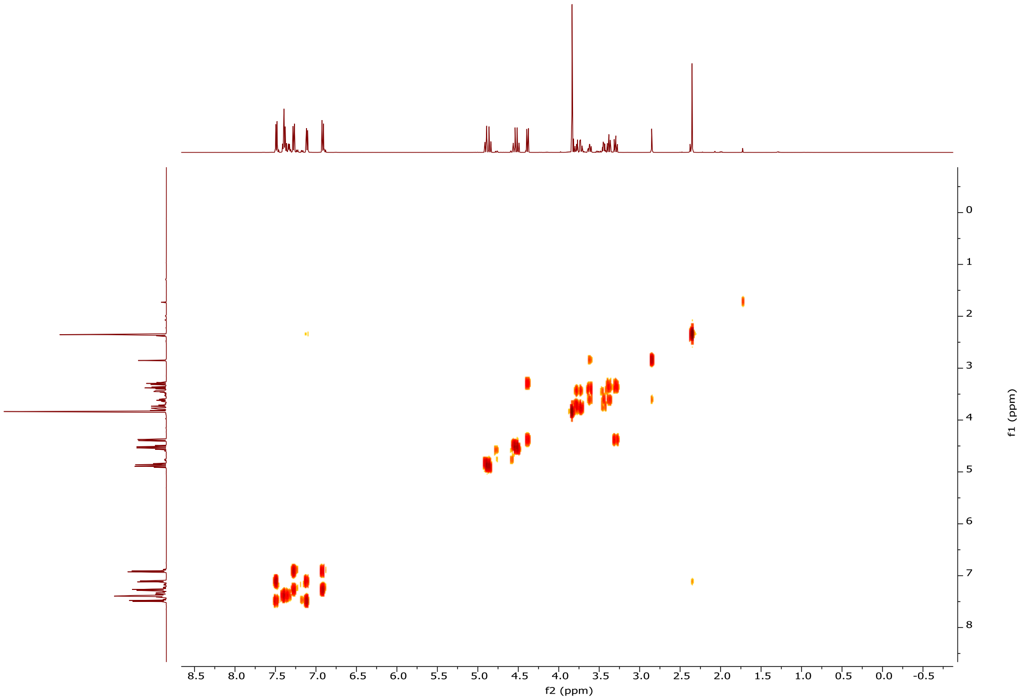

^{1}H-^{1}H gCOSY of **10** (500 MHz $CDCl_3$)

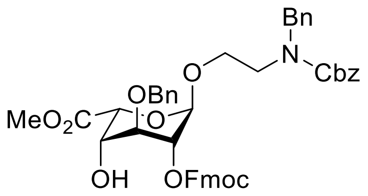

11

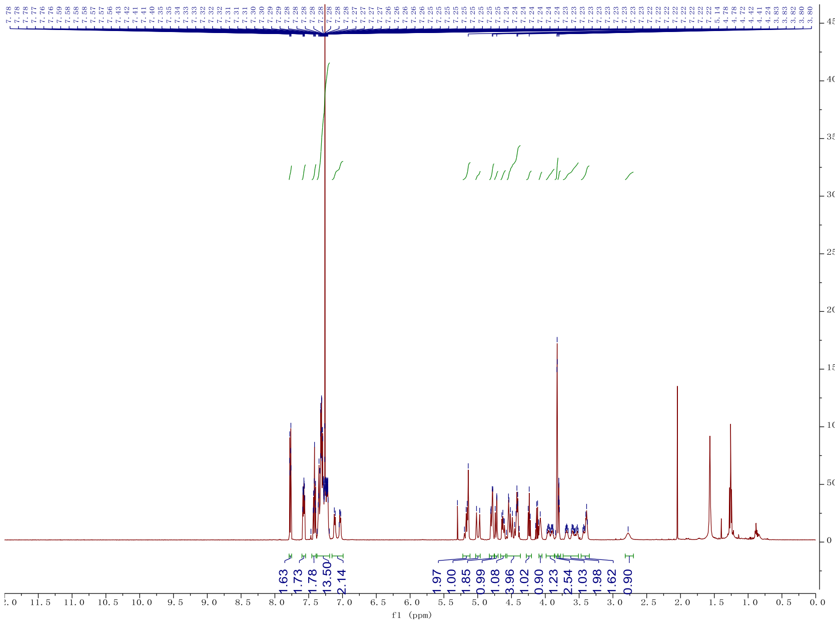

^{1}H-NMR of **11** (500 MHz CDCl$_3$)

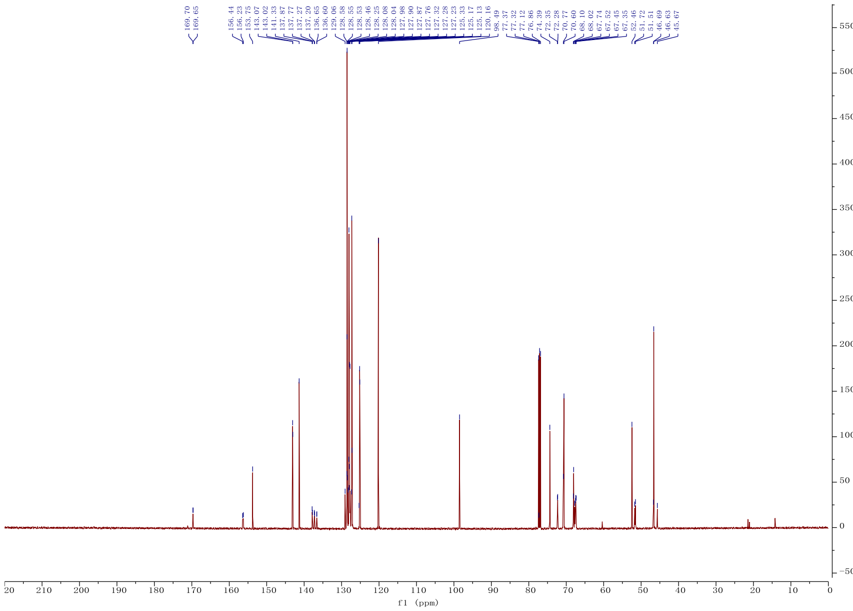

^{13}C-NMR of **11** (125 MHz CDCl$_3$)

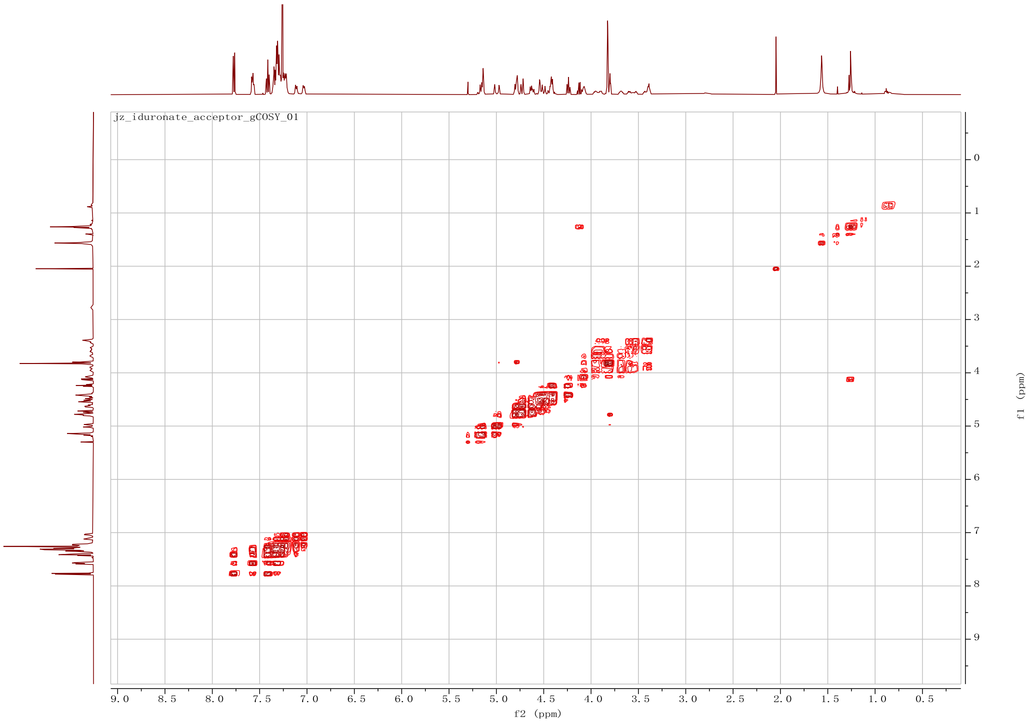

^{1}H-^{1}H gCOSY of **11** (500 MHz $CDCl_3$)

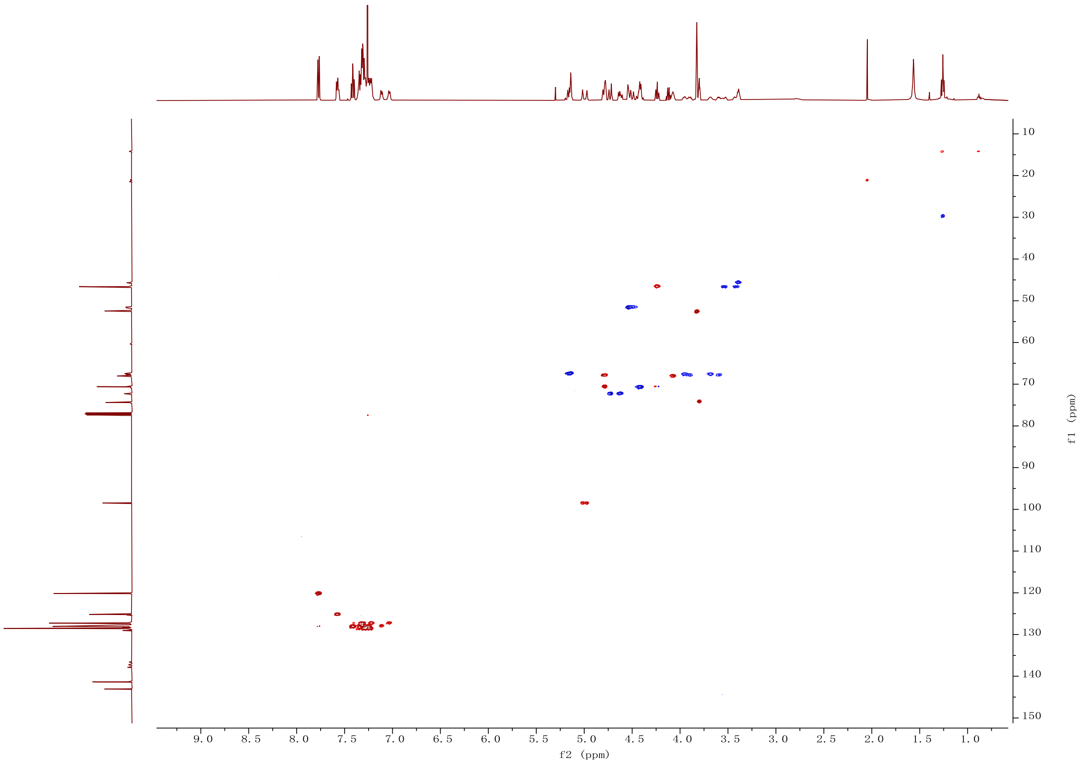

^{1}H-^{13}C gHSQCAD of **11** (500 MHz $CDCl_3$)

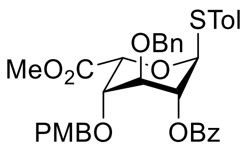

13

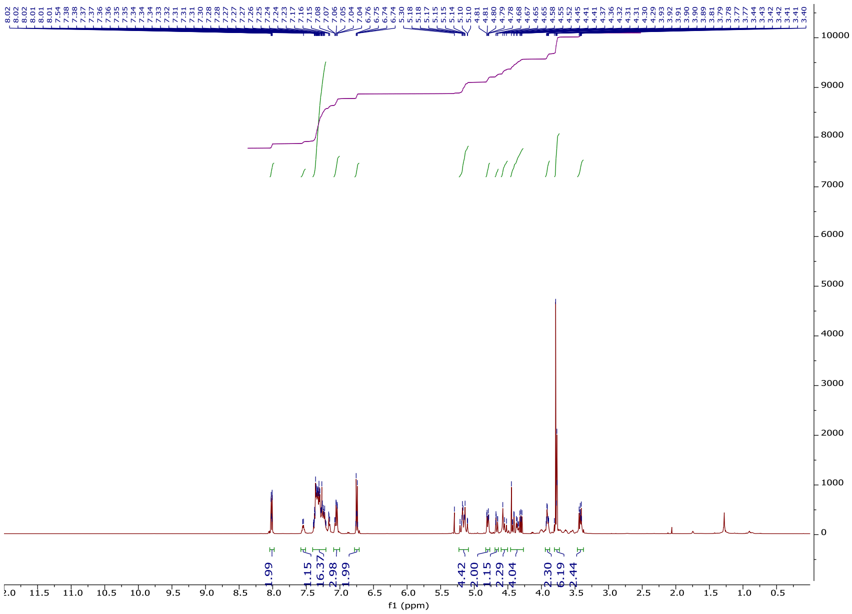

^{1}H-NMR of **13** (500 MHz $CDCl_3$)

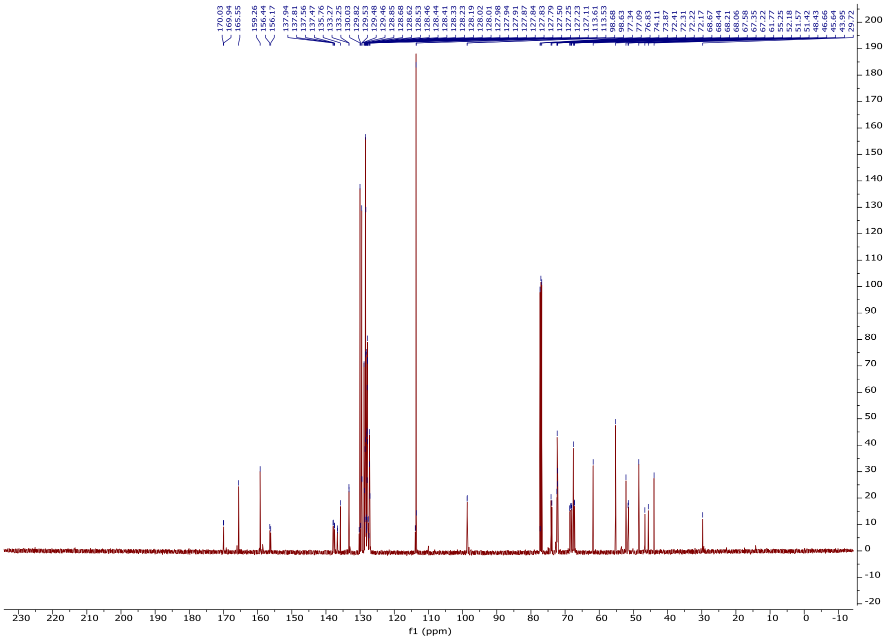

^{13}C-NMR of **13** (125 MHz $CDCl_3$)

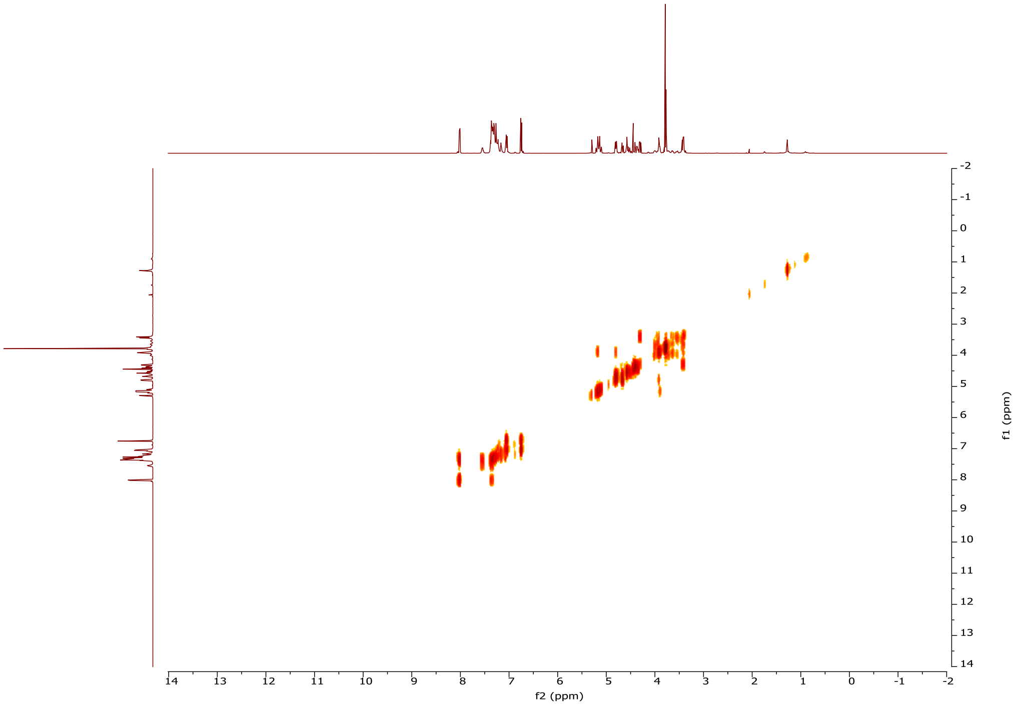

^{1}H-^{1}H gCOSY of **13** (500 MHz $CDCl_3$)

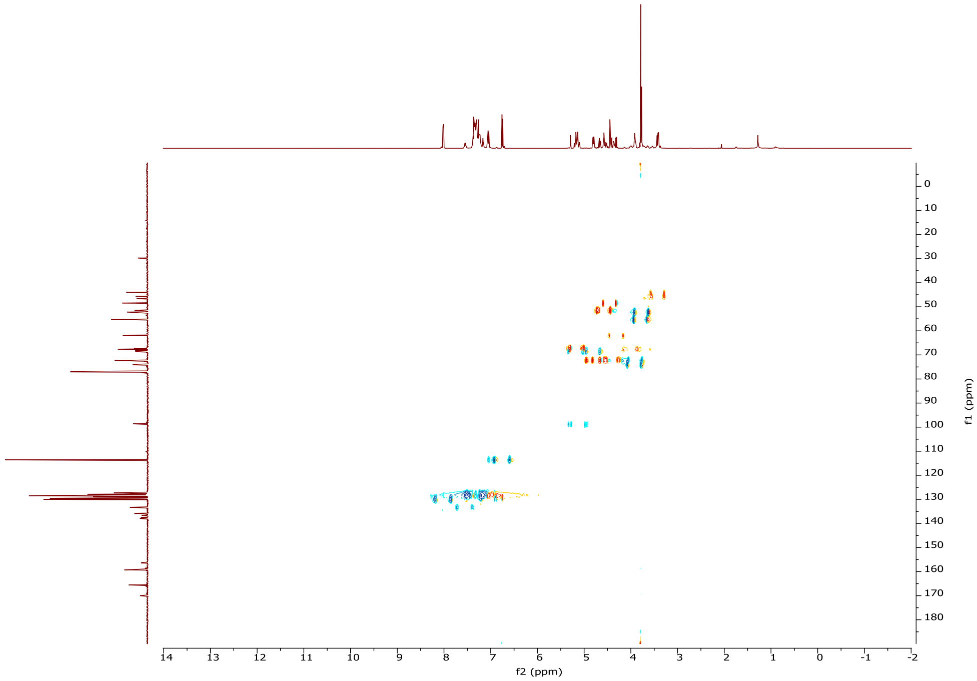

^{1}H-^{13}C gHSQCAD of **13** (500 MHz $CDCl_3$)

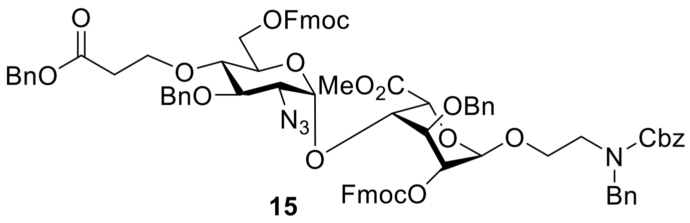

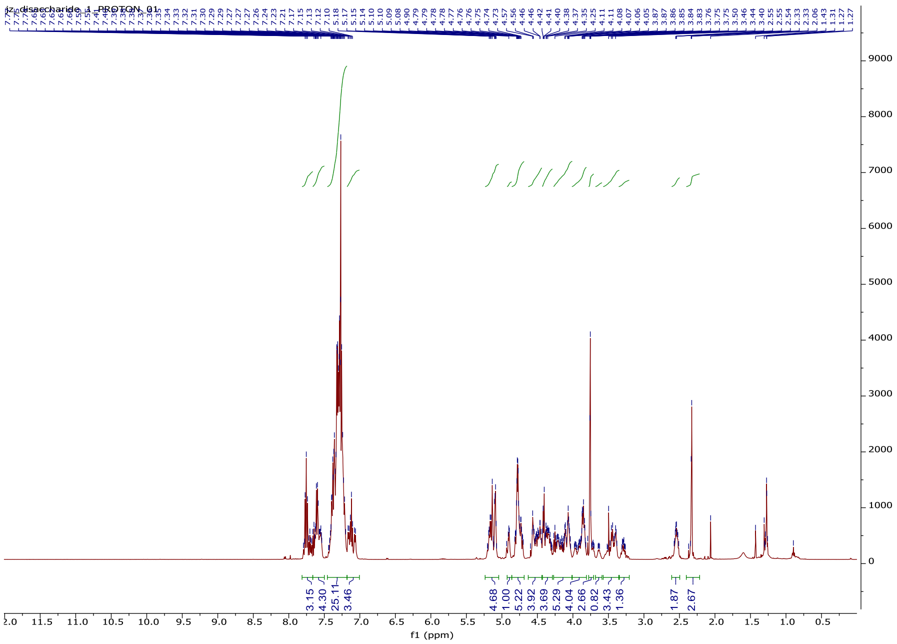

^{1}H-NMR of **15** (500 MHz $CDCl_3$)

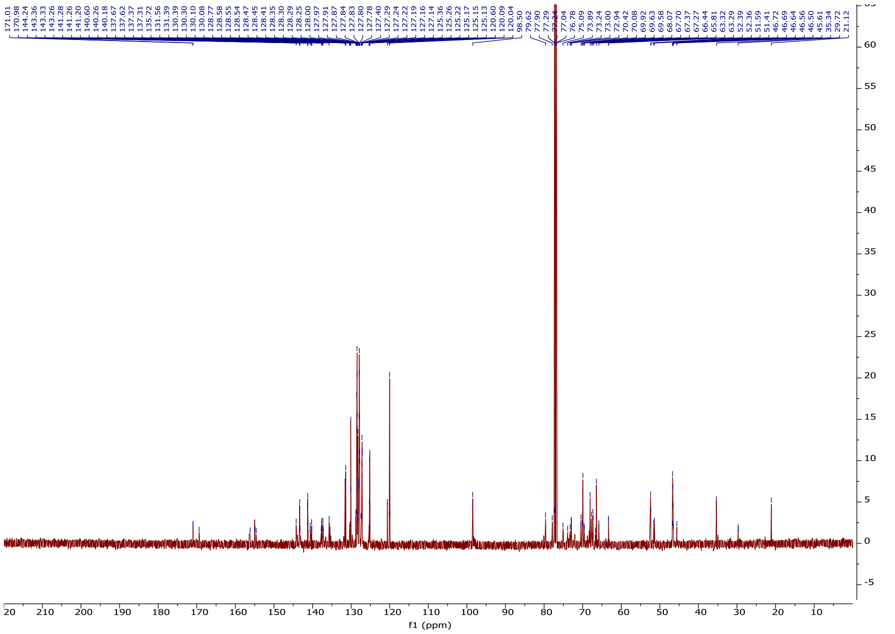

^{13}C-NMR of **15** (125 MHz $CDCl_3$)

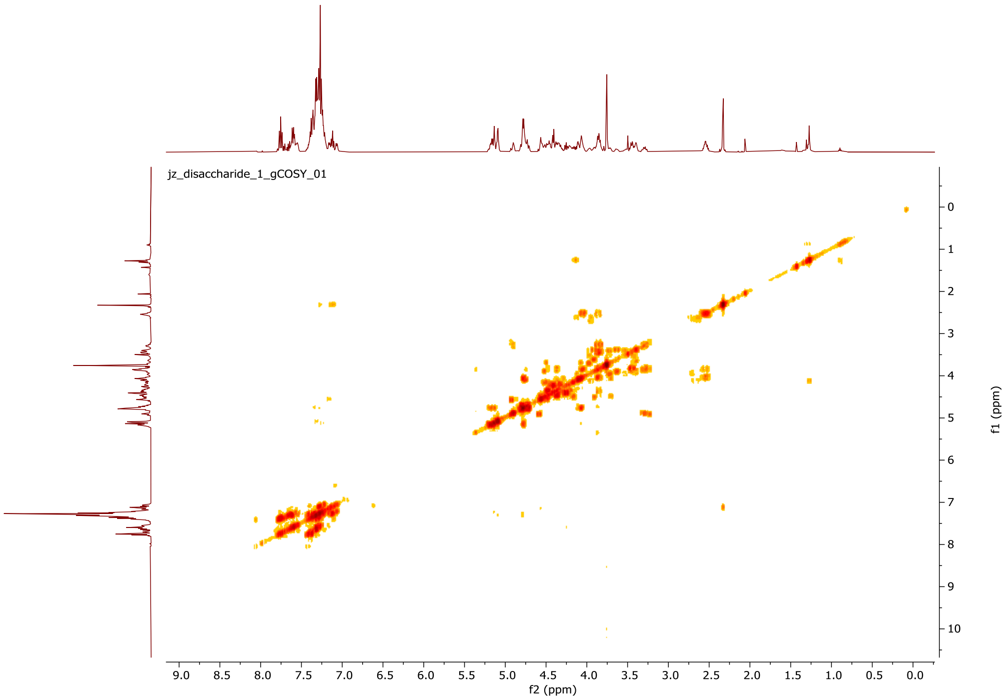

^{1}H-^{1}H gCOSY of **15** (500 MHz $CDCl_3$)

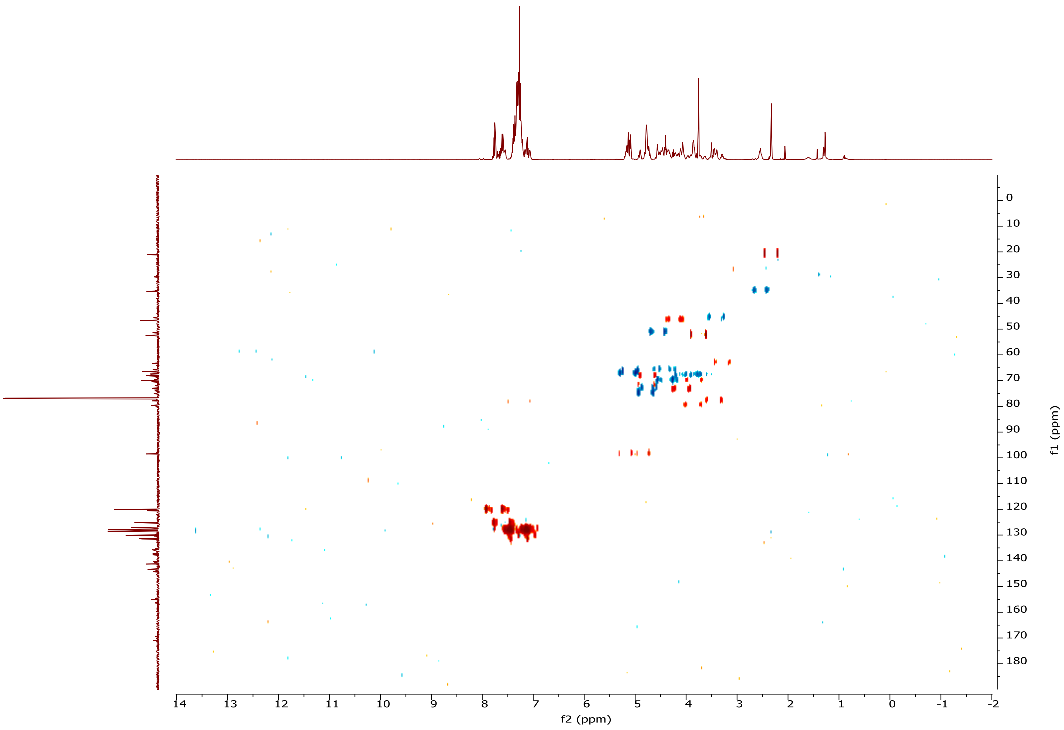

^{1}H-^{13}C gHSQCAD of **15** (500 MHz $CDCl_3$)

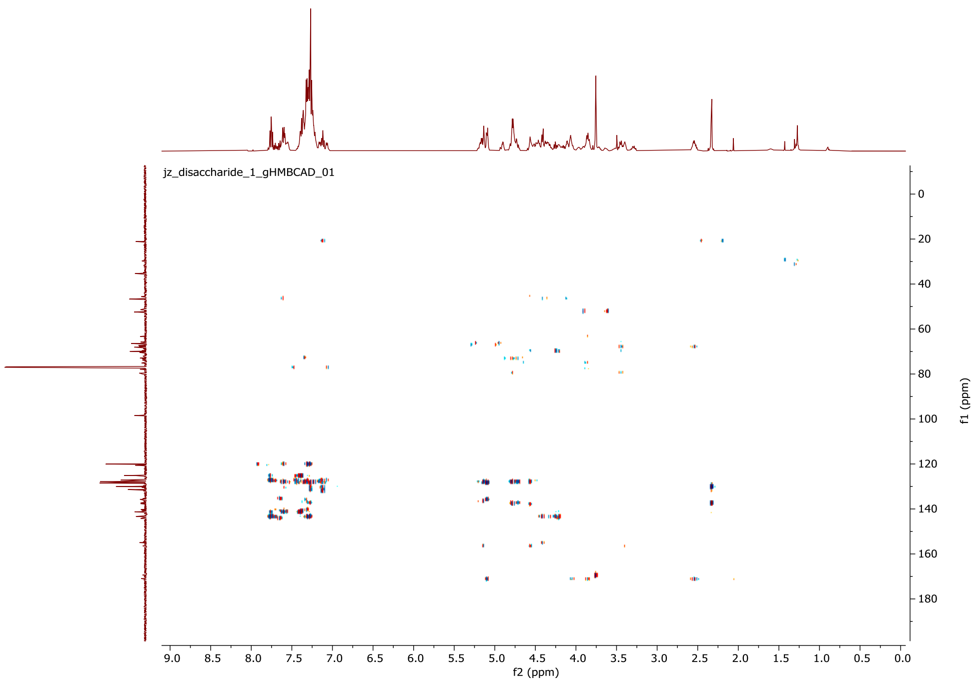

^{1}H-^{13}C gHMBCAD of **15** (500 MHz $CDCl_3$)

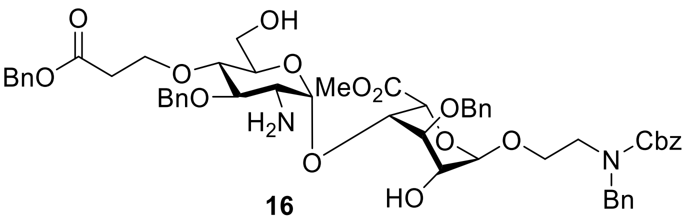

16

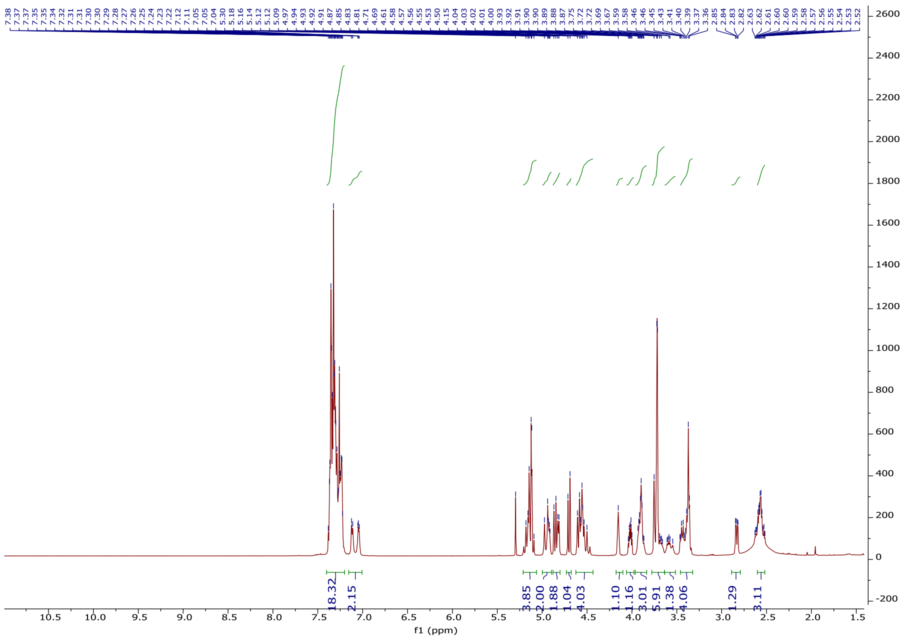

^{1}H-NMR of **16** (500 MHz $CDCl_3$)

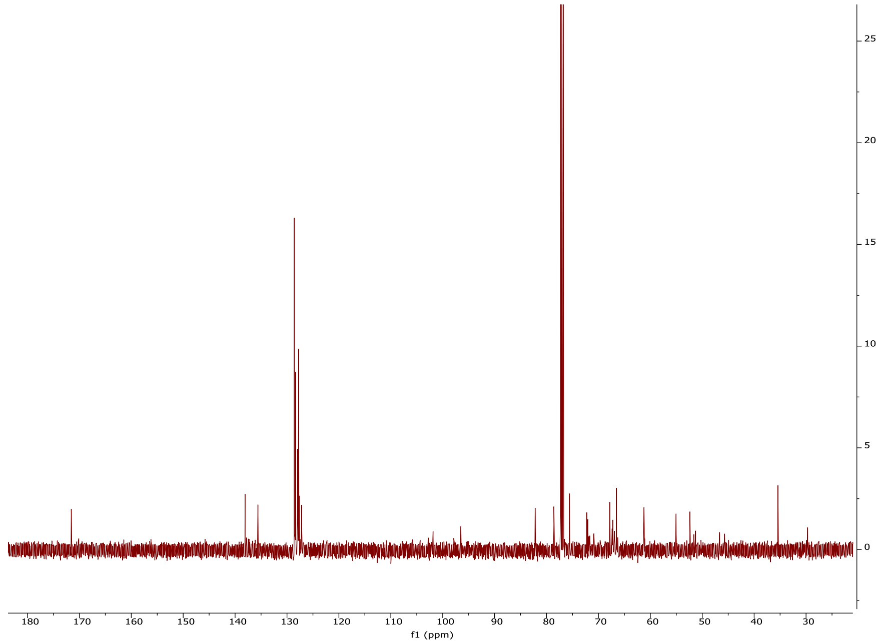

^{13}C-NMR of **16** (125 MHz $CDCl_3$)

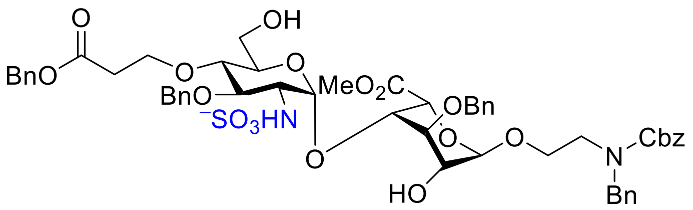

20

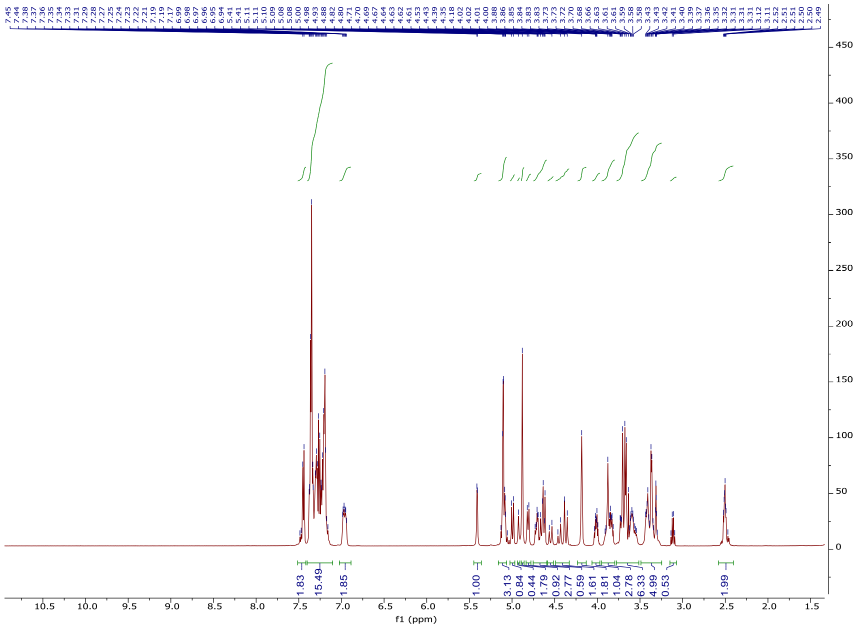

^{1}H-NMR of **20** (500 MHz CD_3OD)

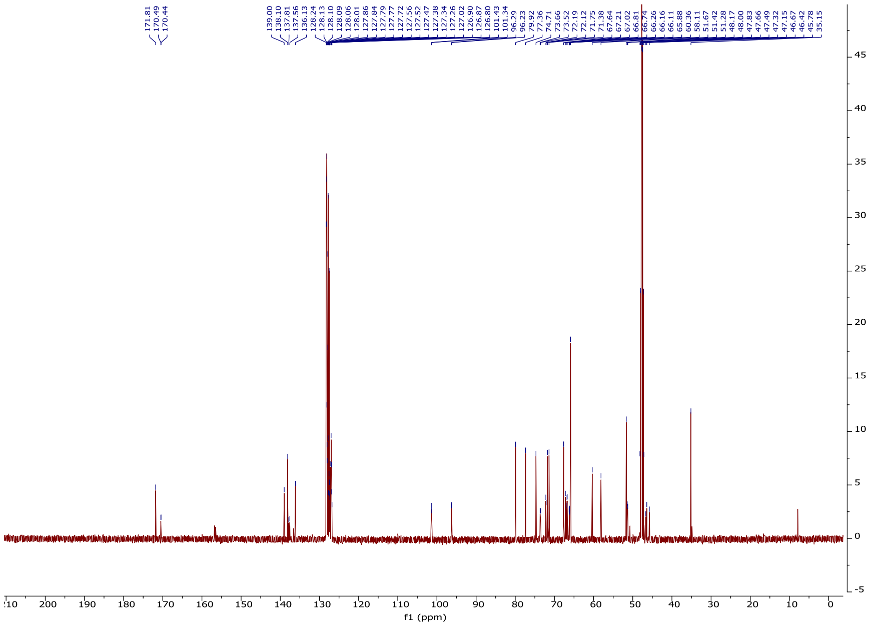

^{13}C-NMR of **20** (125 MHz CD_3OD)

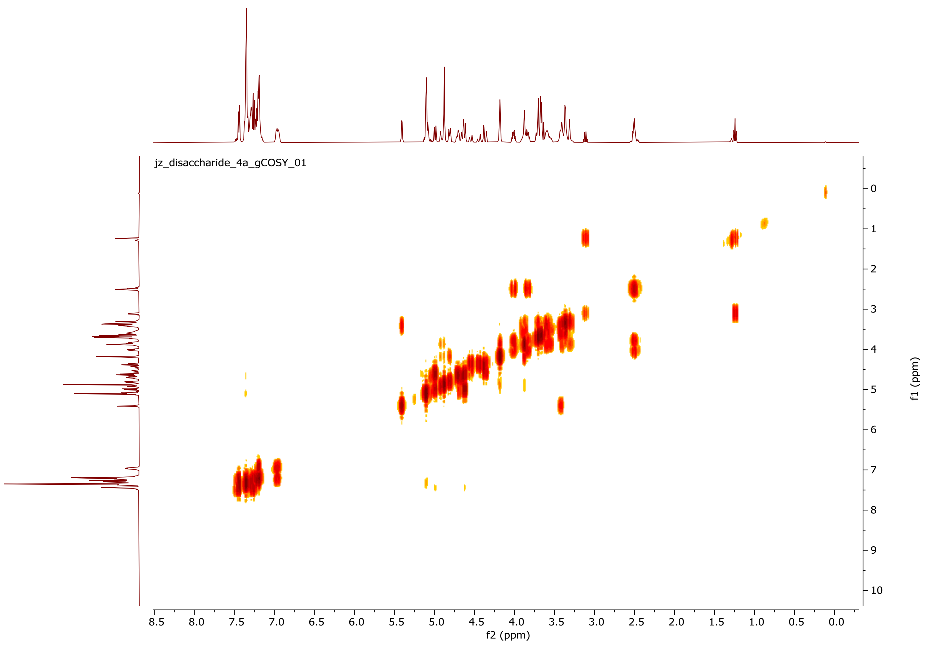

^{1}H-^{1}H gCOSY of **20** (500 MHz CD_3OD)

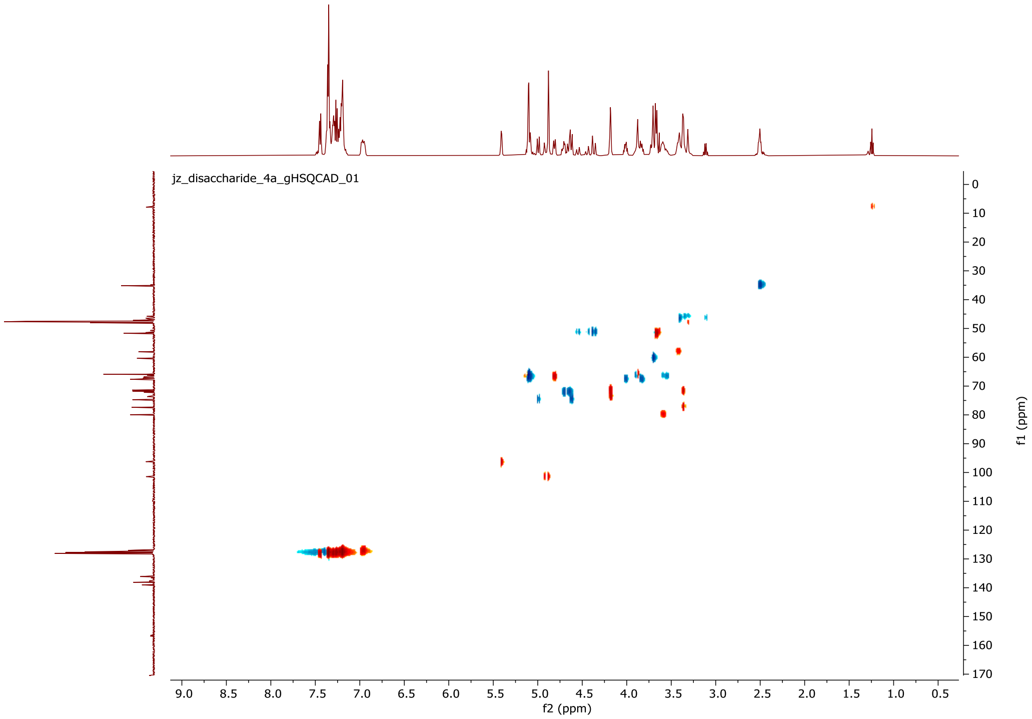

^{1}H-^{13}C gHSQCAD of **20** (500 MHz $CDCl_3$)

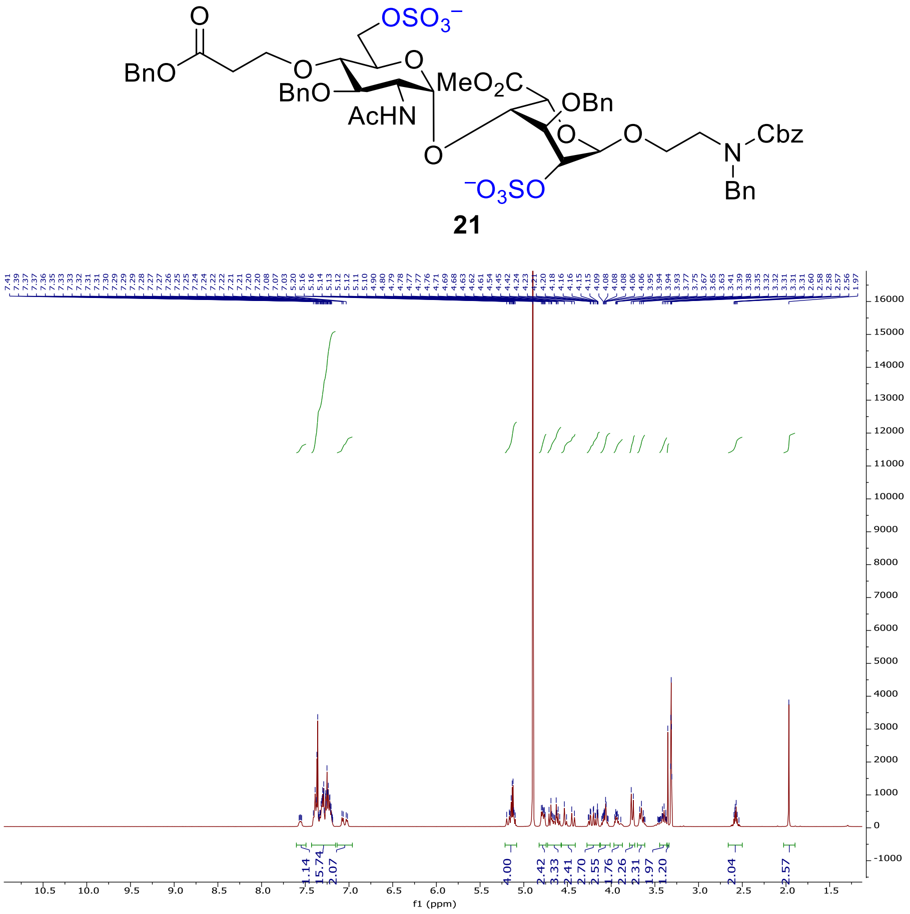

^{1}H-NMR of **21** (500 MHz CD_3OD)

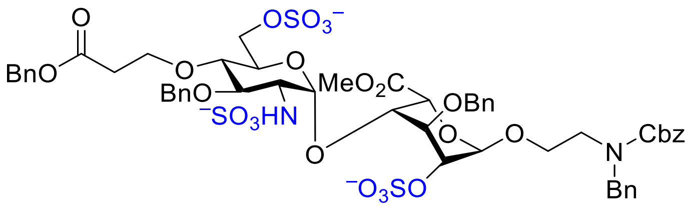

22

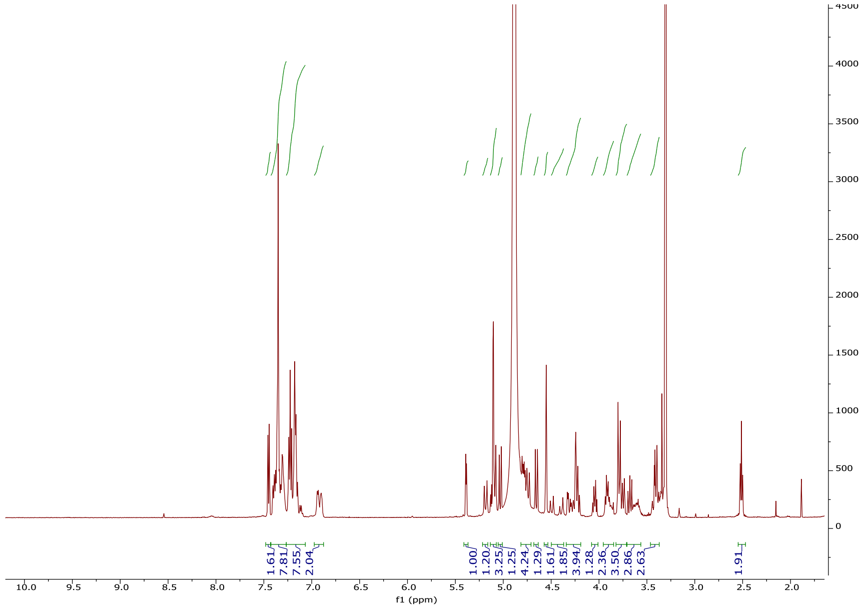

^{1}H-NMR of **22** (500 MHz CD_3OD)

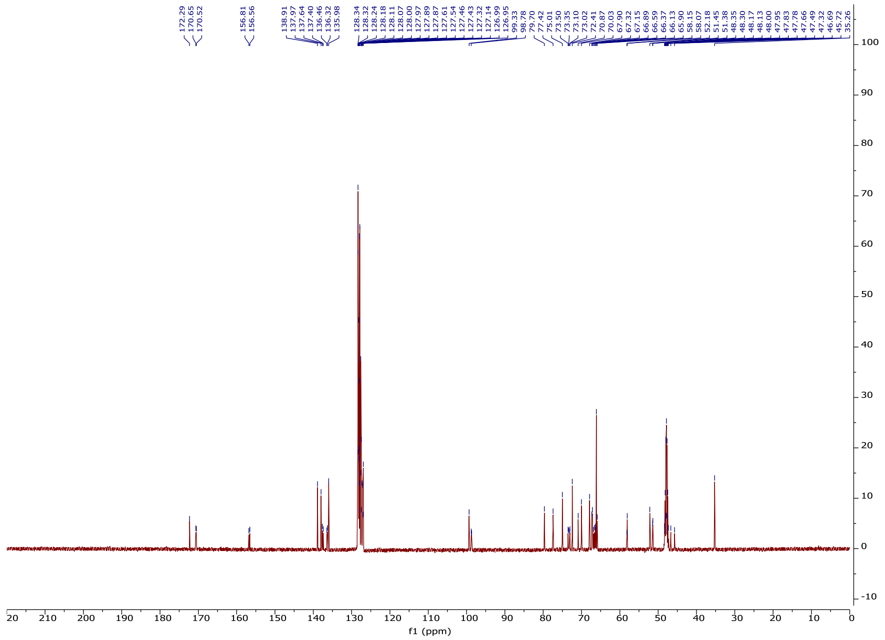

^{13}C-NMR of **22** (125 MHz CD_3OD)

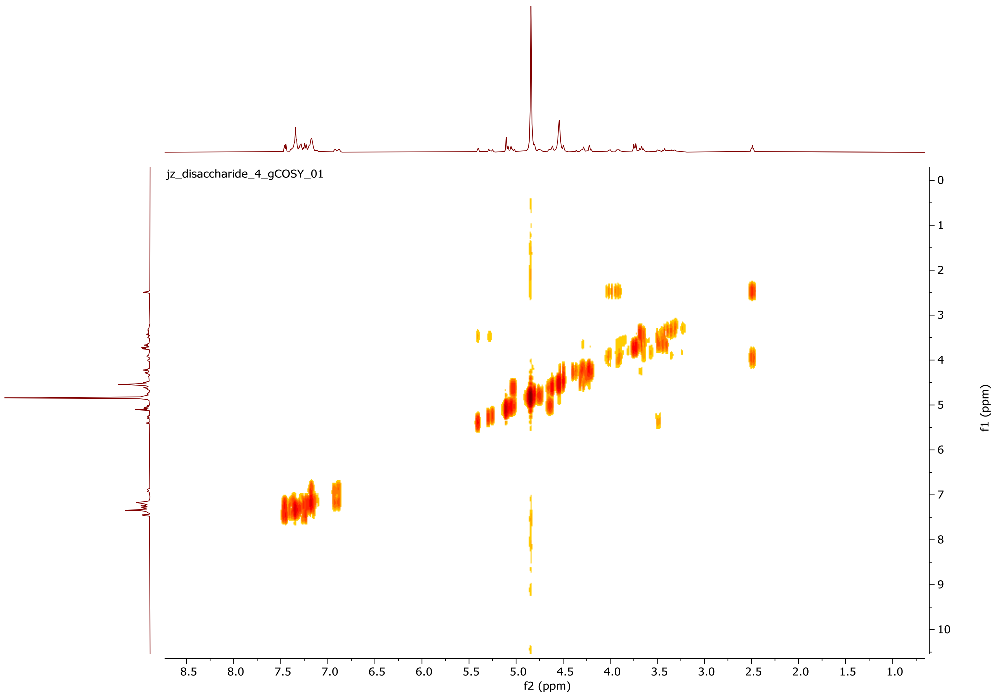

^{1}H-^{1}H gCOSY of **22** (500 MHz CD_3OD)

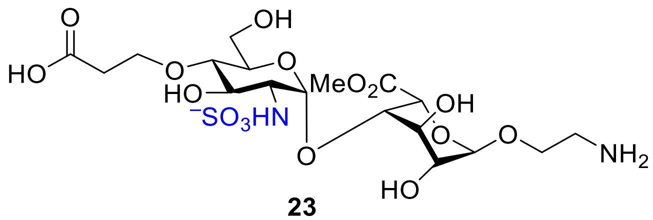

23

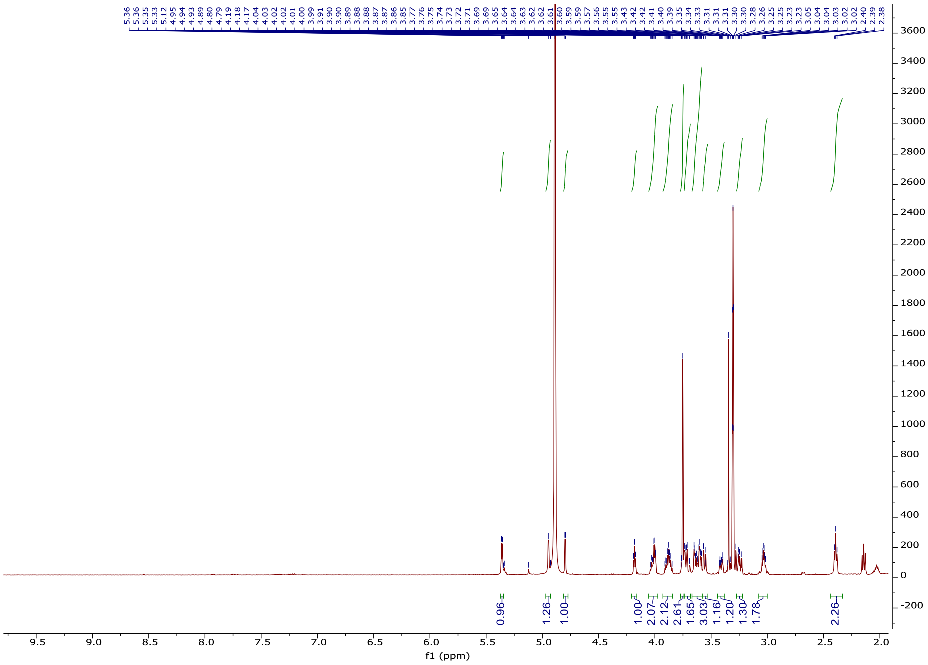

^{1}H-NMR of **23** (500 MHz CD_3OD)

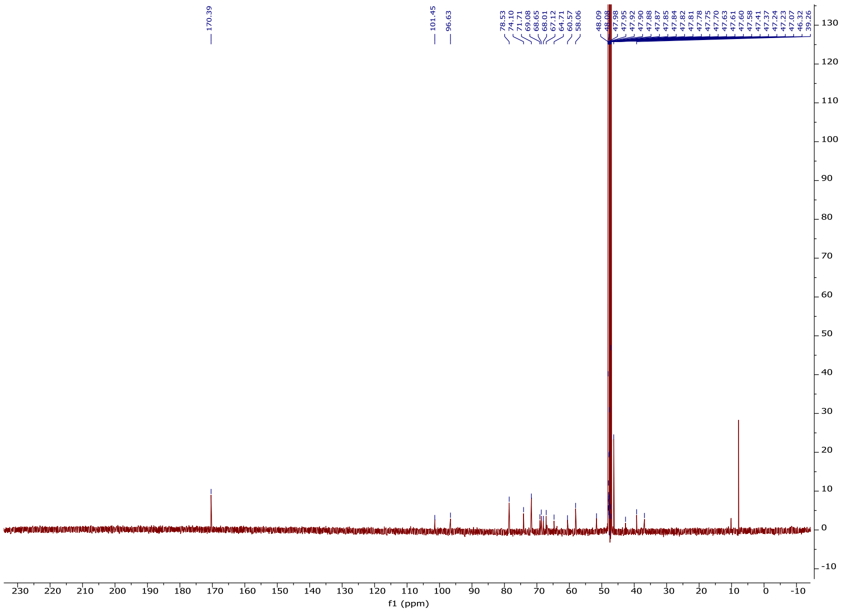

^{13}C-NMR of **23** (125 MHz CD_3OD)

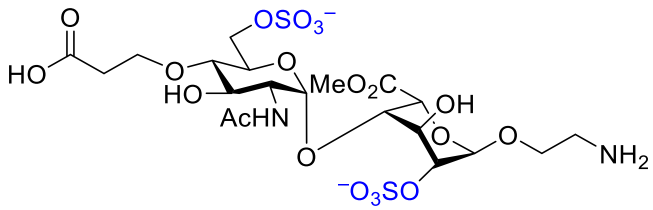

24

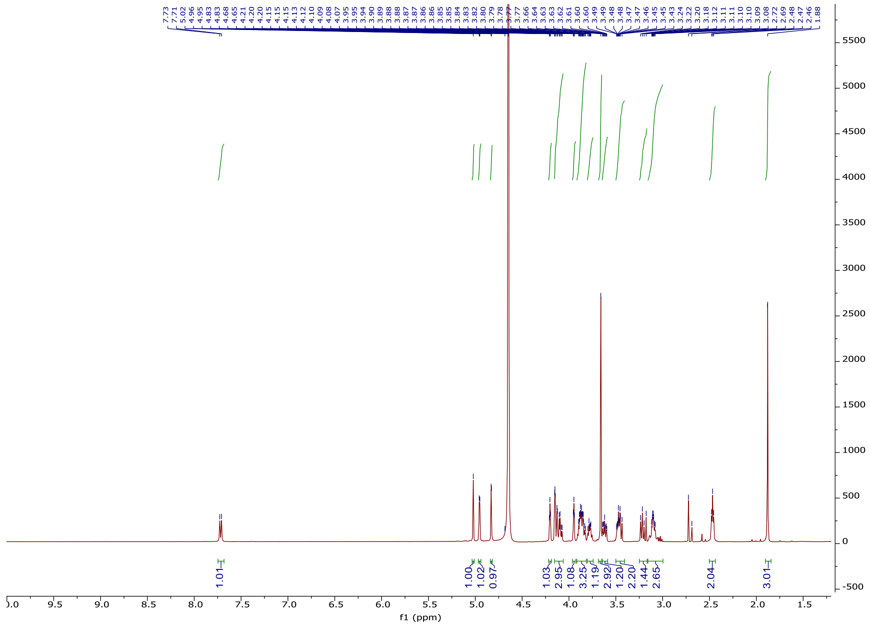

^{1}H-NMR of **24** (500 MHz D_2O)

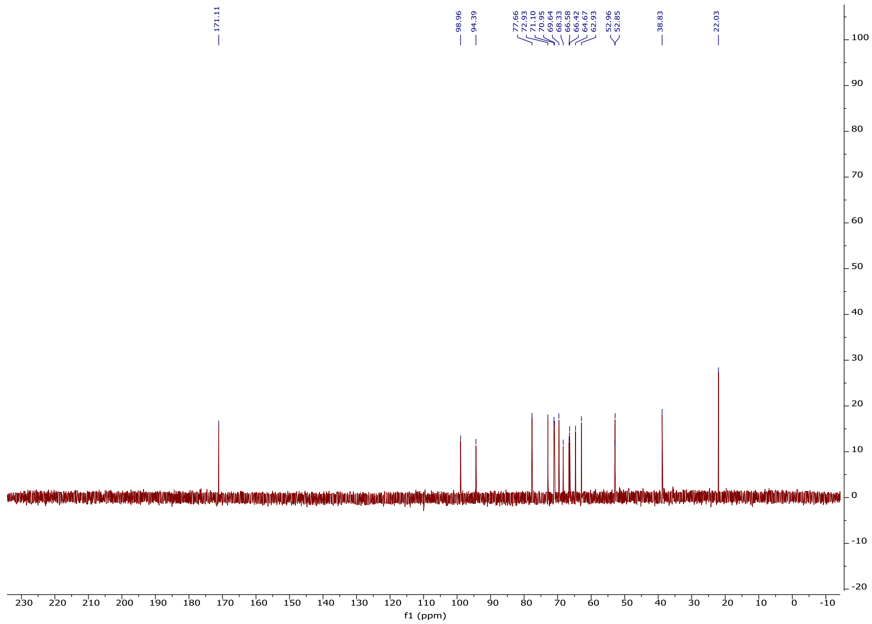

^{13}C-NMR of **24** (125 MHz D_2O)

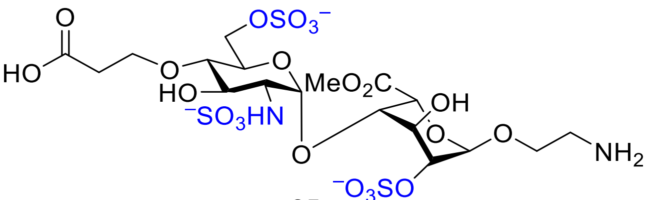

25

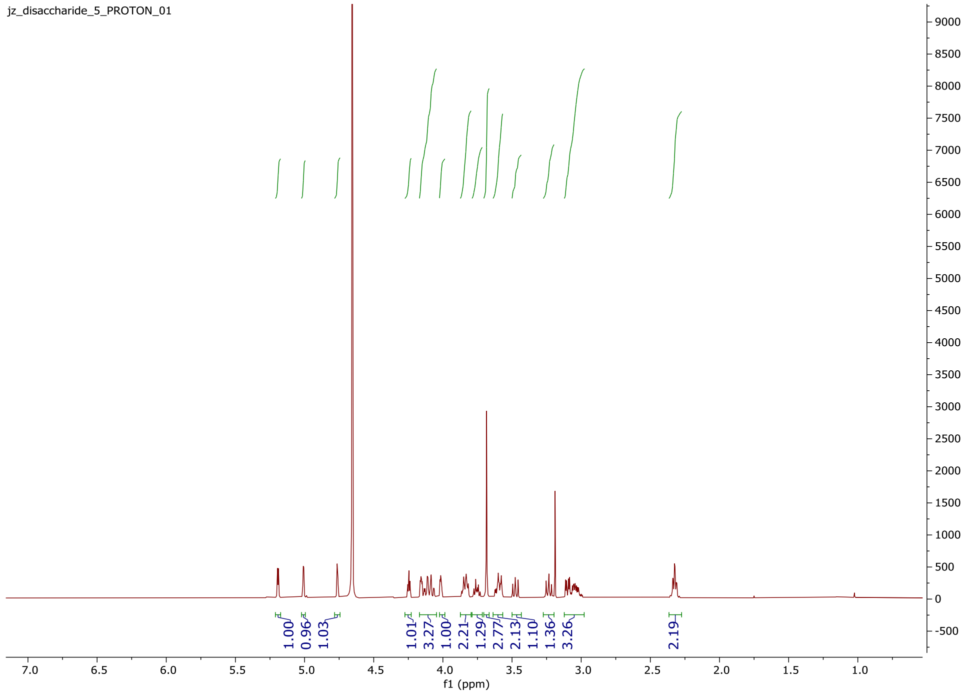

^{1}H-NMR of **25** (500 MHz D_2O)

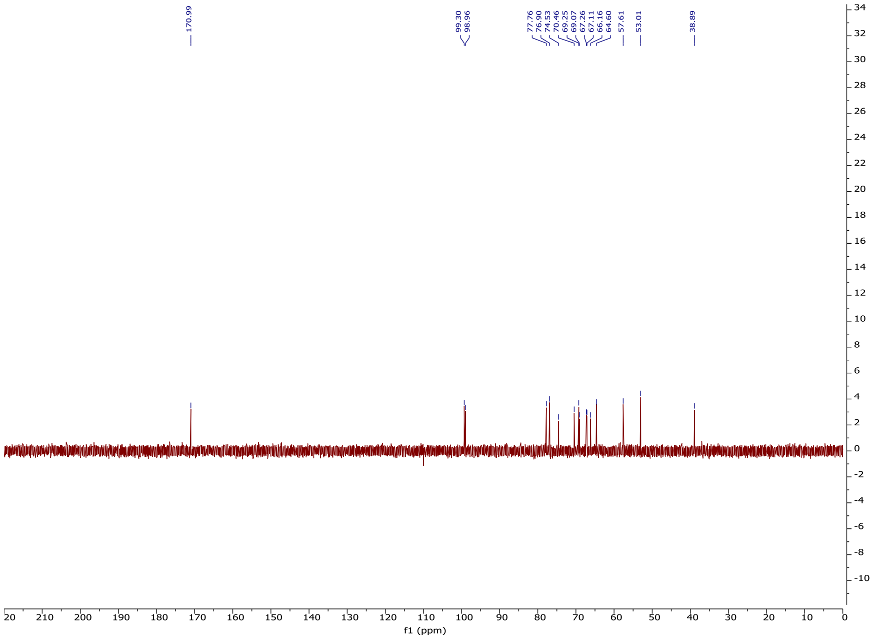

^{13}C-NMR of **25** (125 MHz D_2O)

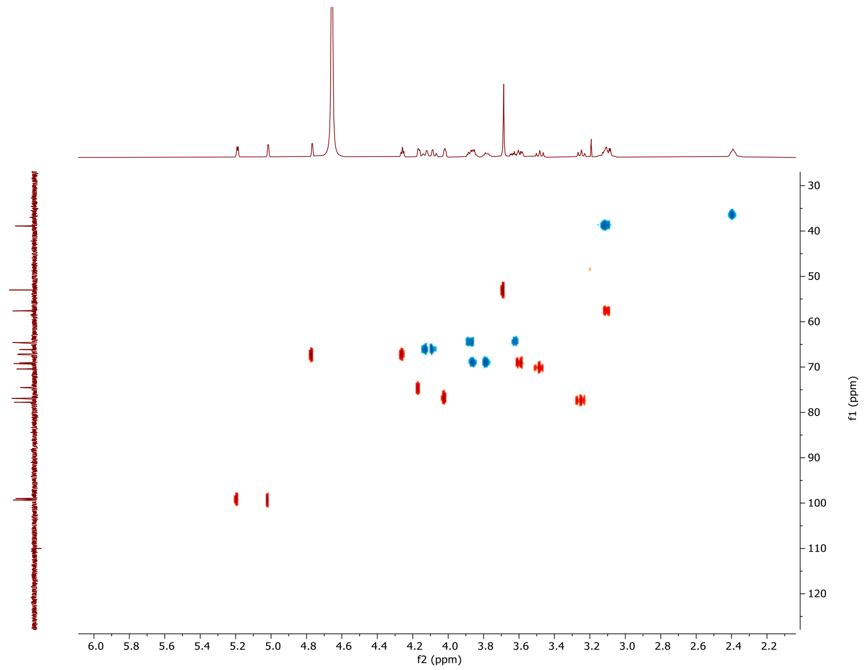

^{1}H-^{13}C gHSQCAD of **25** (500 MHz D_2O)

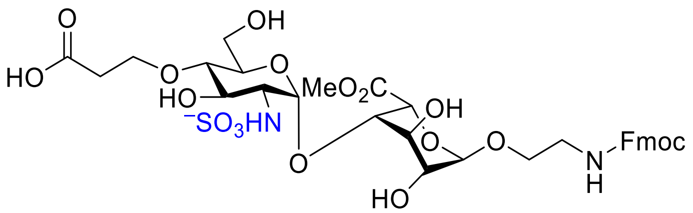

17

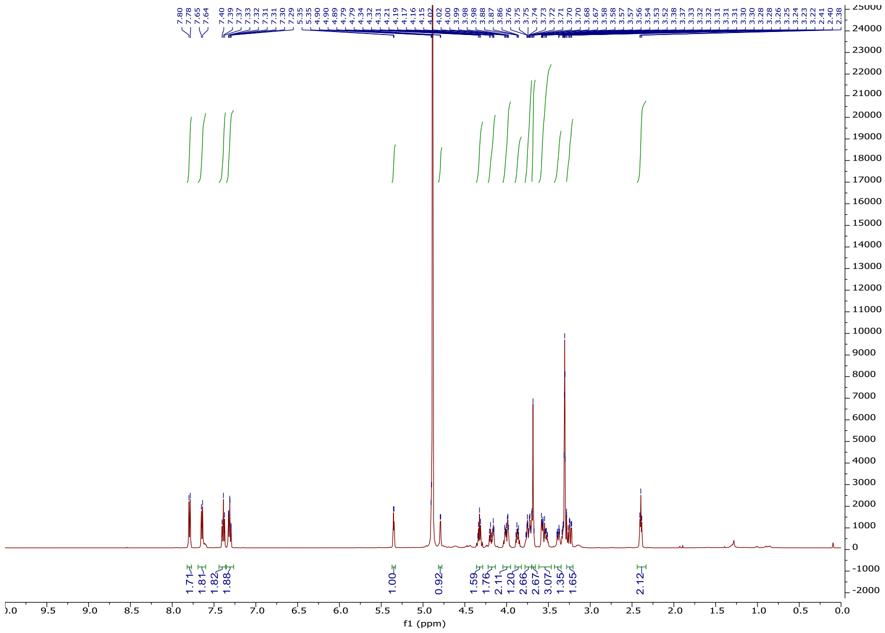

^{1}H-NMR of **17** (500 MHz CD_3OD)

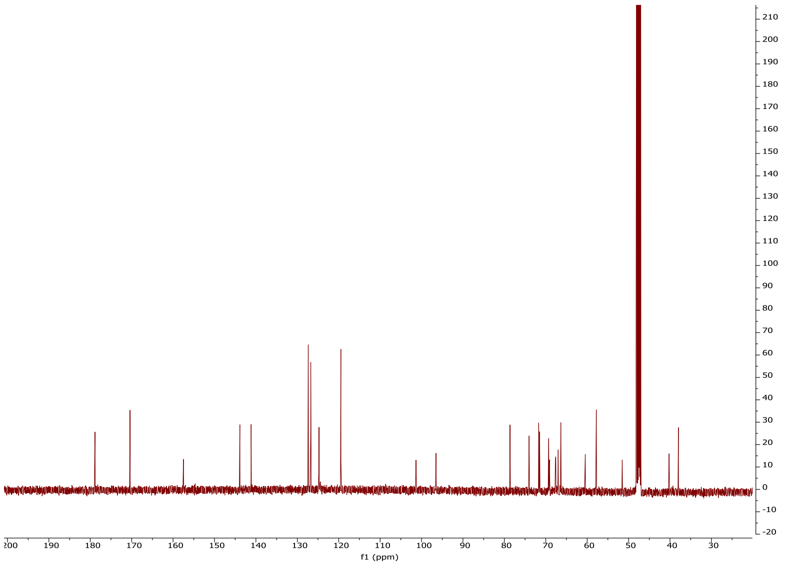

^{13}C-NMR of **17** (125 MHz CD_3OD)

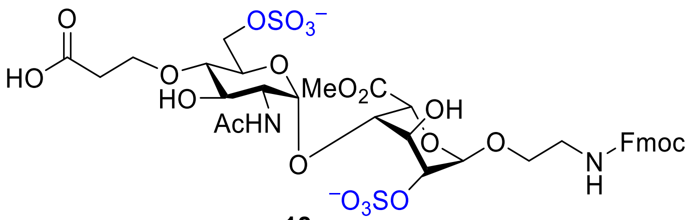

18

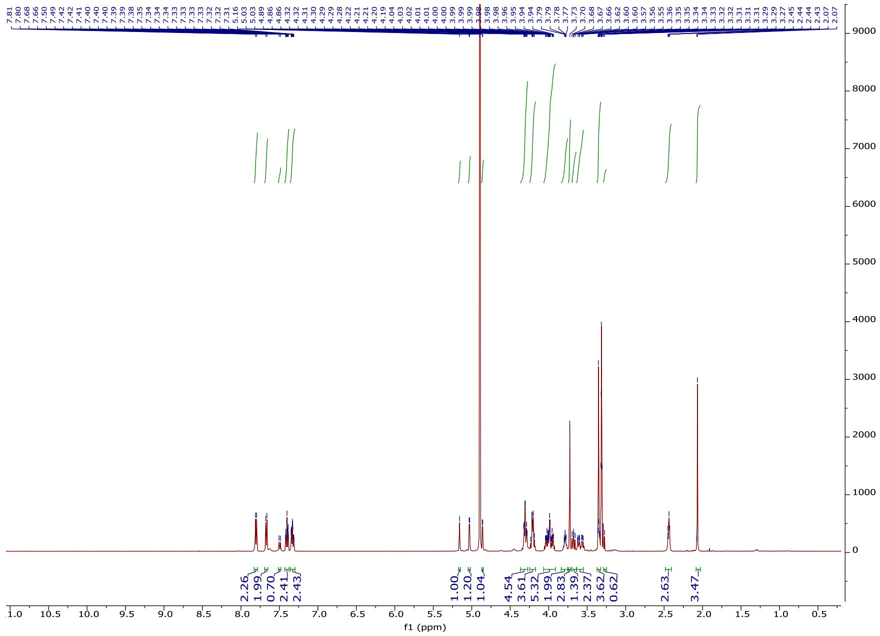

^{1}H-NMR of **18** (500 MHz CD_3OD)

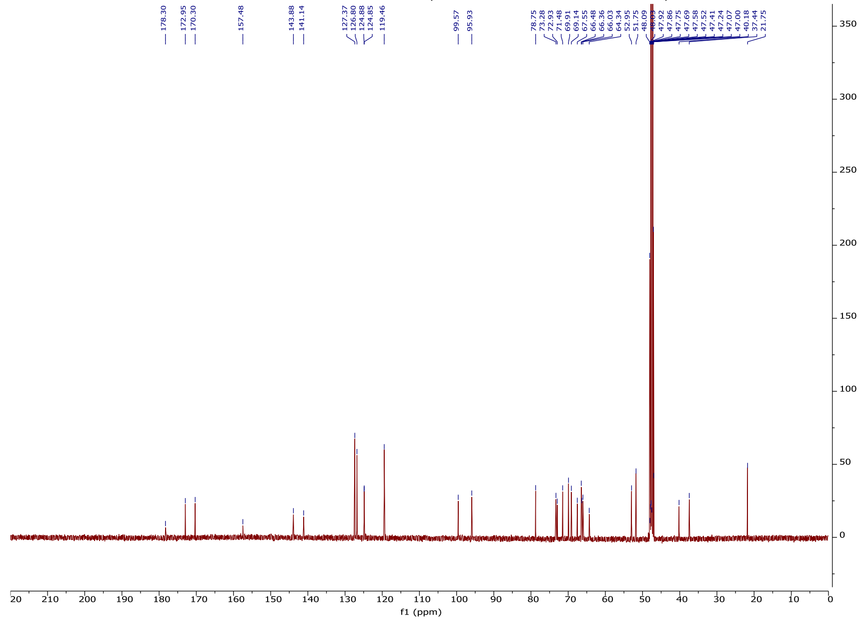

^{13}C-NMR of **18** (125 MHz CD_3OD)

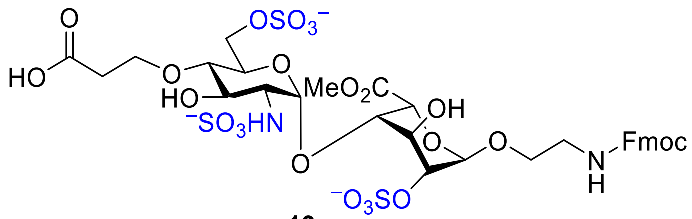

19

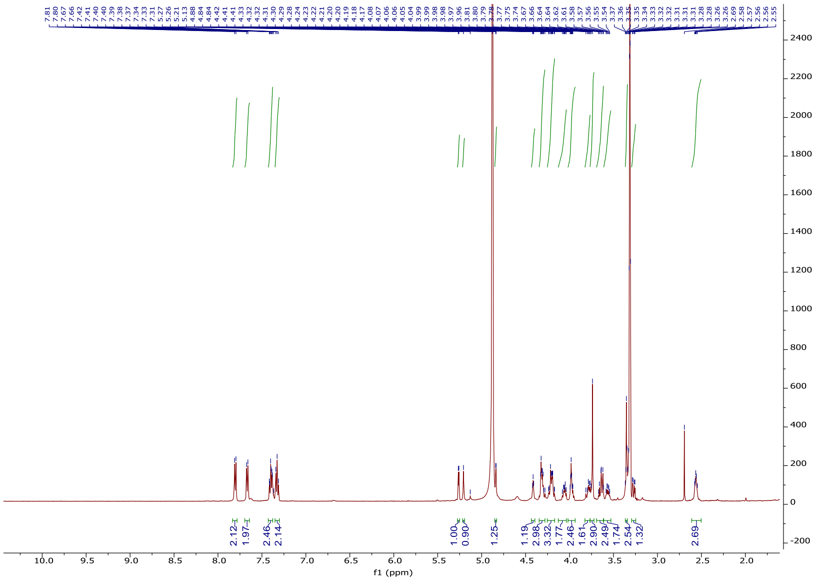

^{1}H-NMR of **19** (500 MHz CD_3OD)

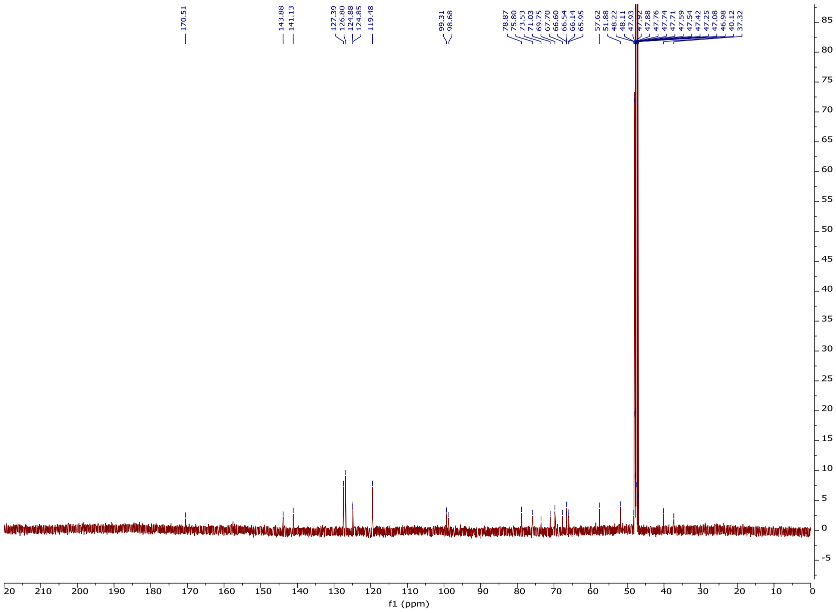

^{13}C-NMR of **19** (125 MHz CD_3OD)

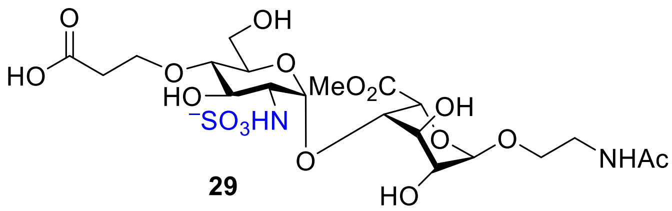

29

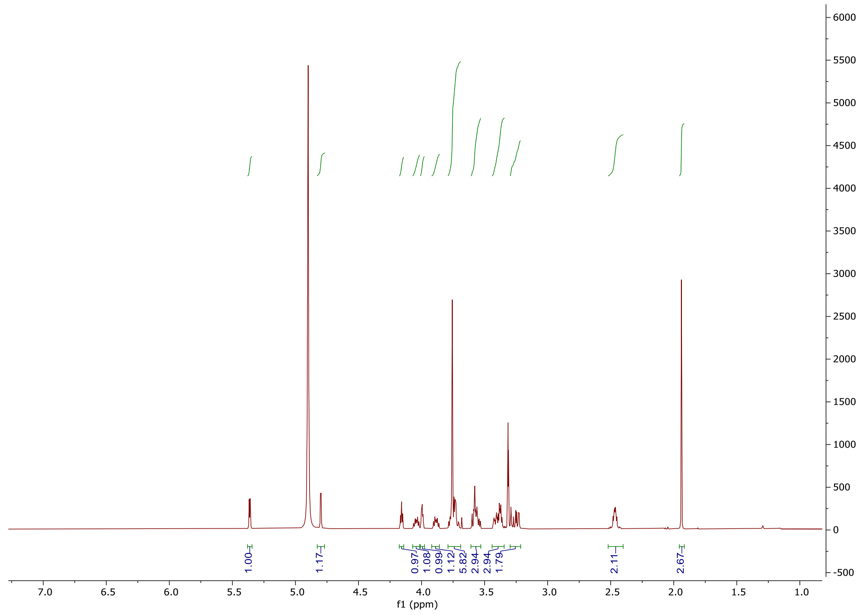

^{1}H-NMR of **29** (500 MHz CD_3OD)

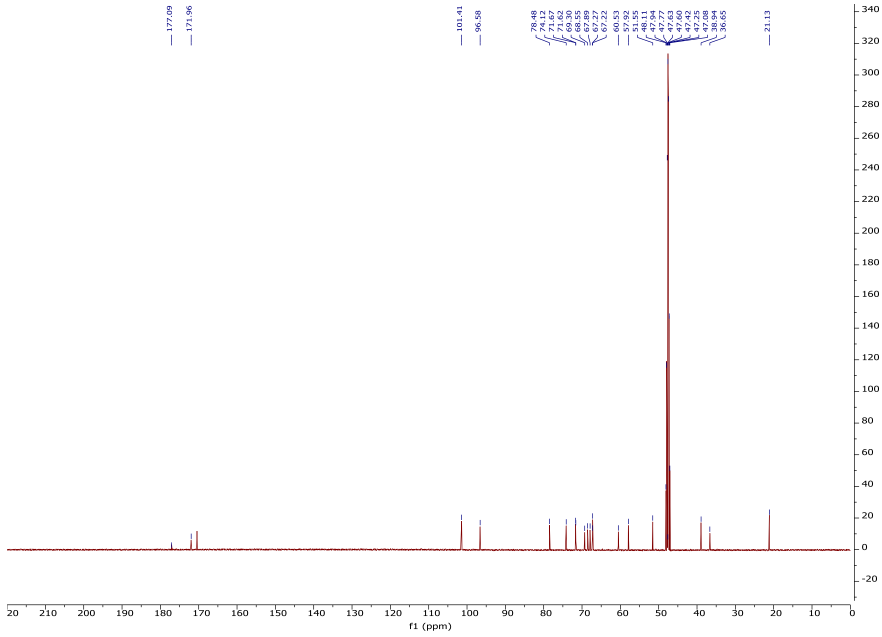

^{13}C-NMR of **29** (125 MHz CD_3OD)

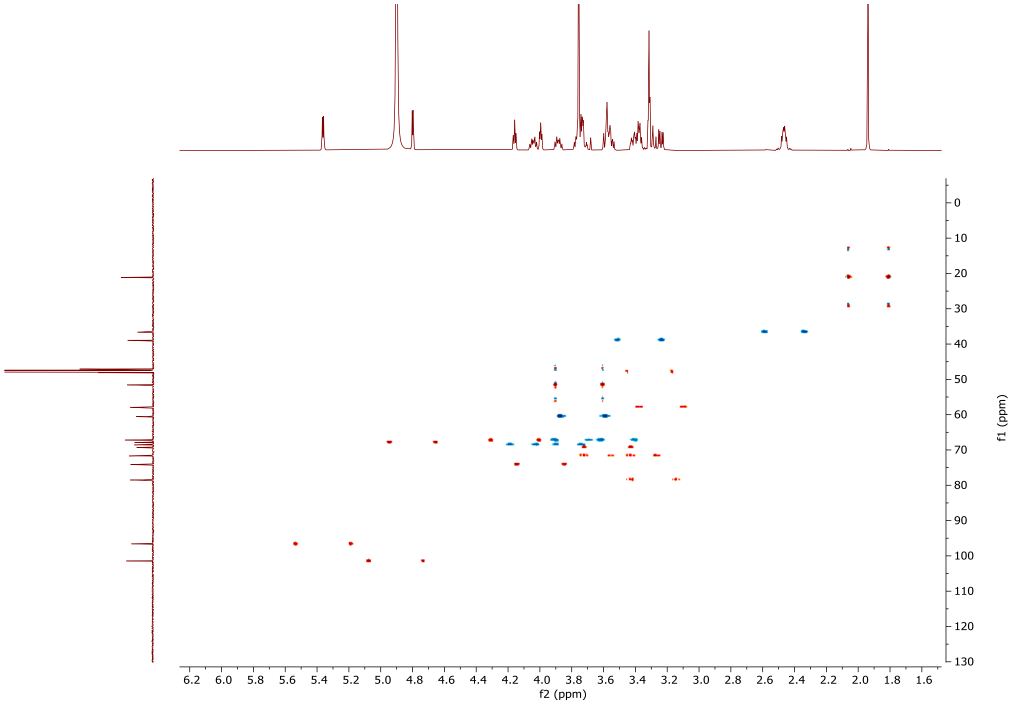

^{1}H-^{13}C gHSQCAD of **29** (500 MHz CD_3OD)

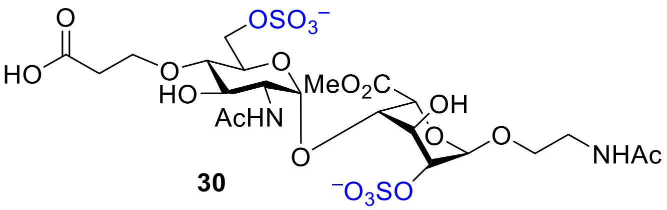

30

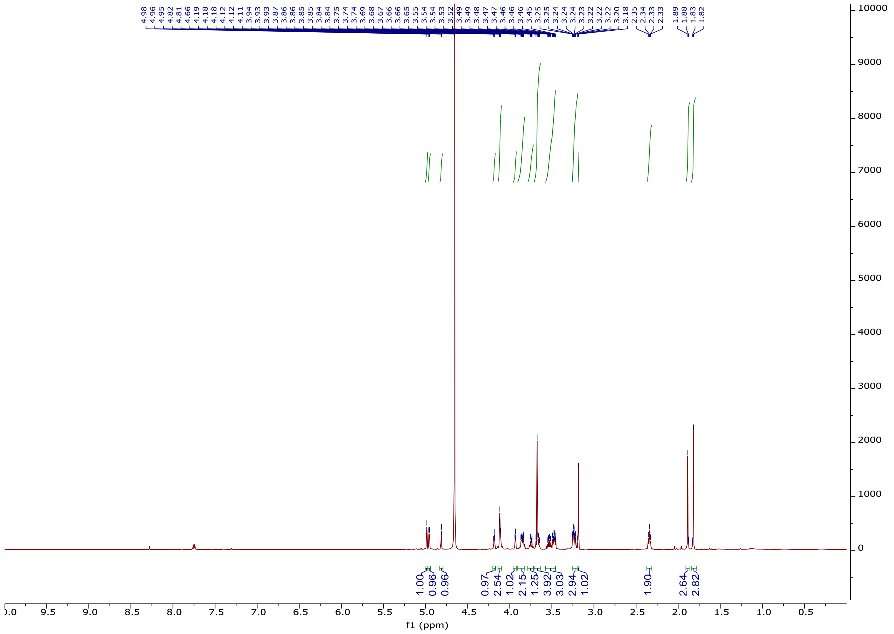

^{1}H-NMR of **30** (500 MHz D_2O)

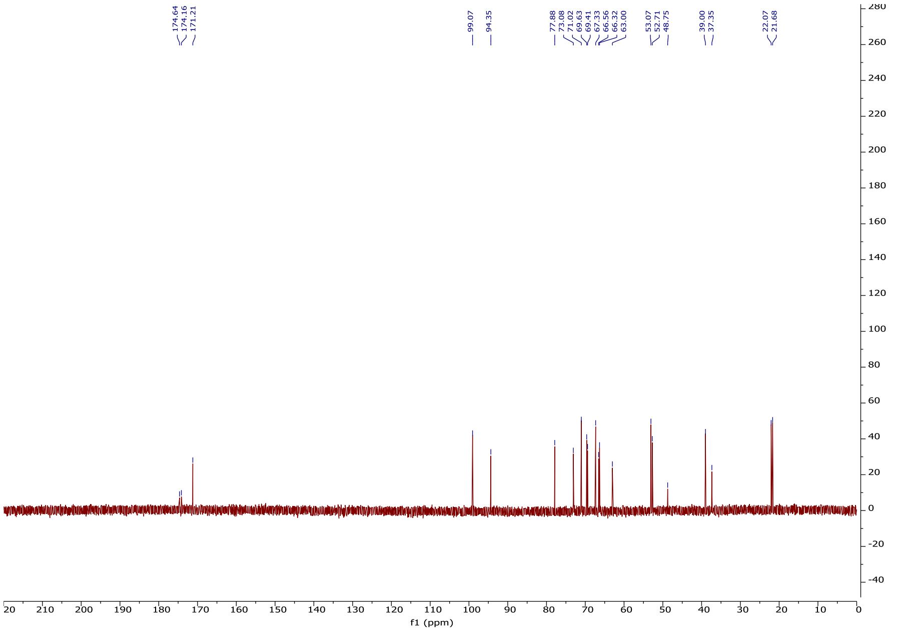

^{13}C-NMR of **30** (125 MHz D_2O)

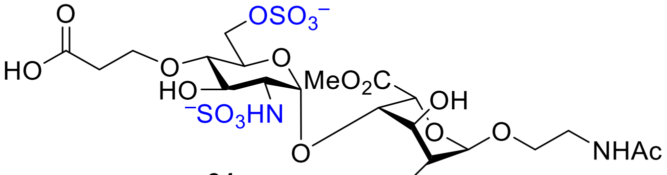

31

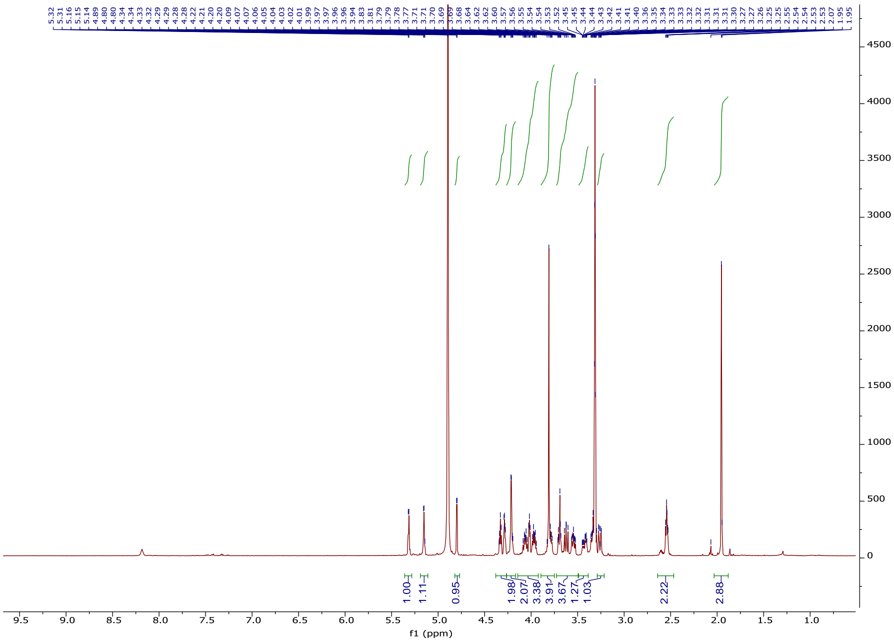

^{1}H-NMR of **31** (500 MHz CD_3OD)

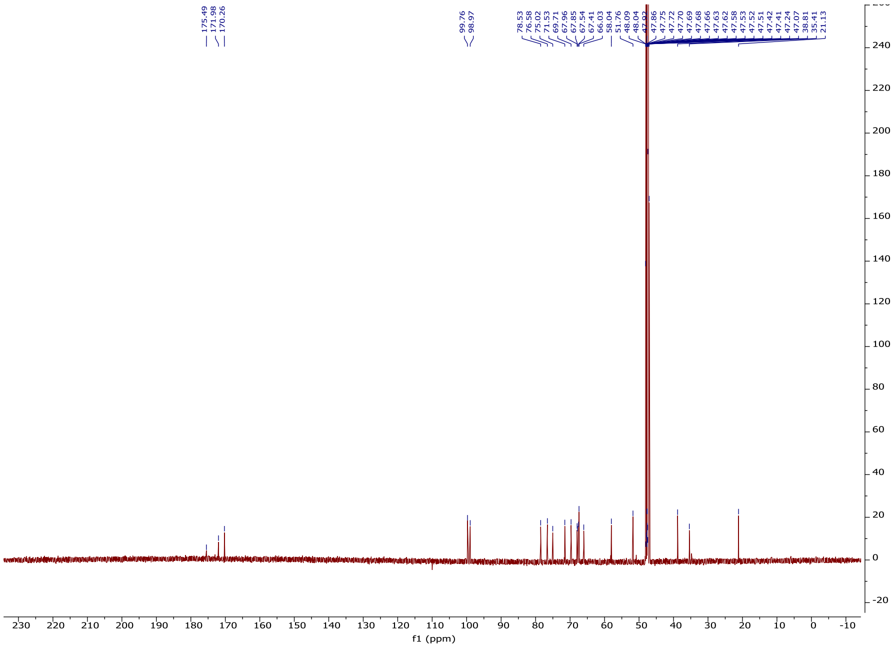

^{13}C-NMR of **31** (125 MHz CD_3OD)

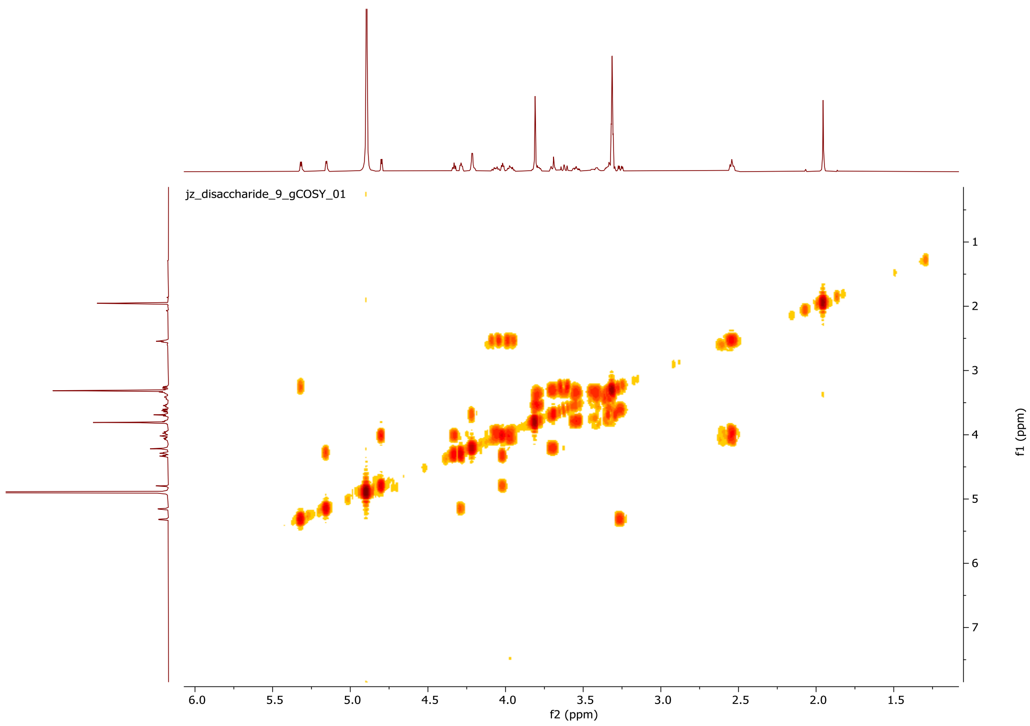

^{1}H-^{1}H gCOSY of **31** (500 MHz CD_3OD)

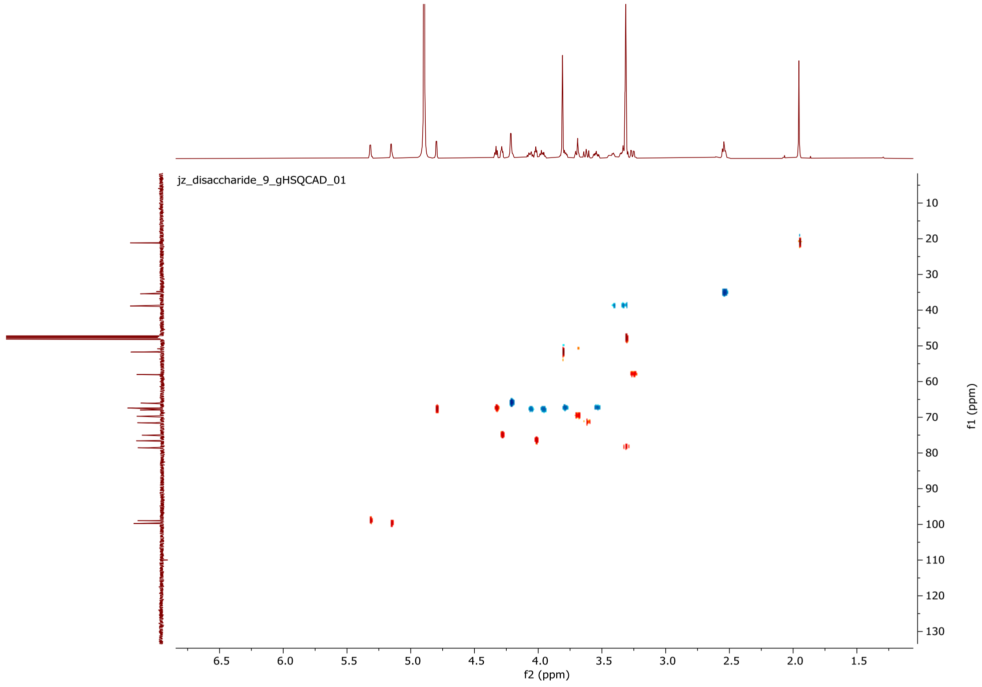

^{1}H-^{13}C gHSQCAD of **31** (500 MHz CD_3OD)

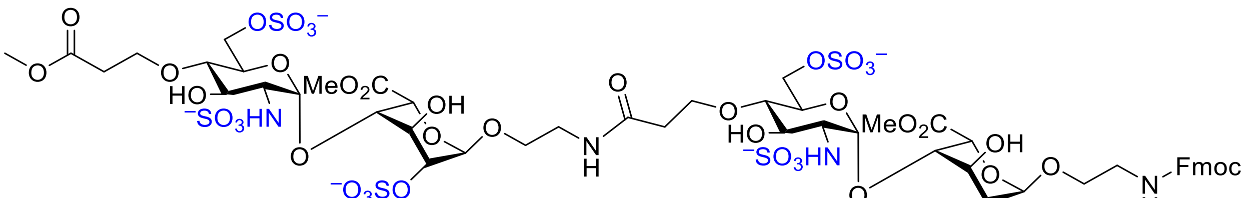

32a

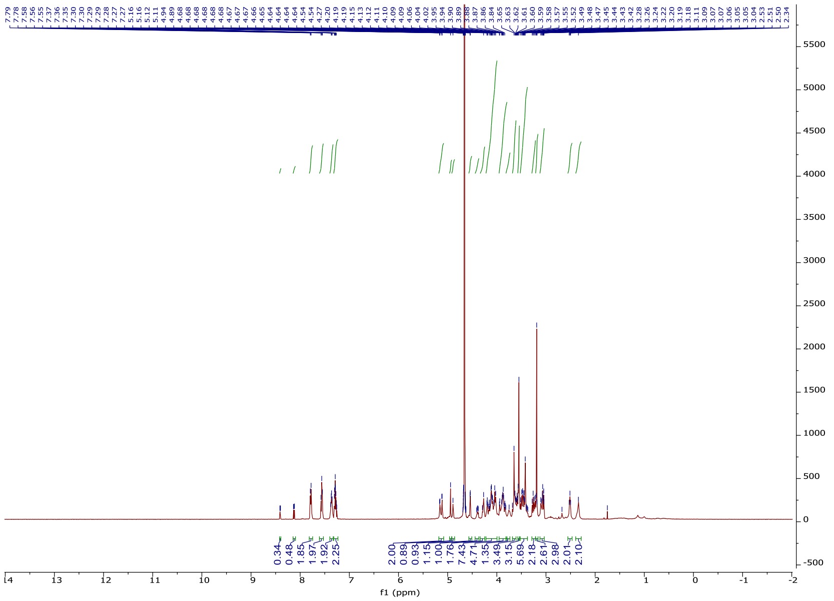

^{1}H-NMR of **32a** (500 MHz D_2O)

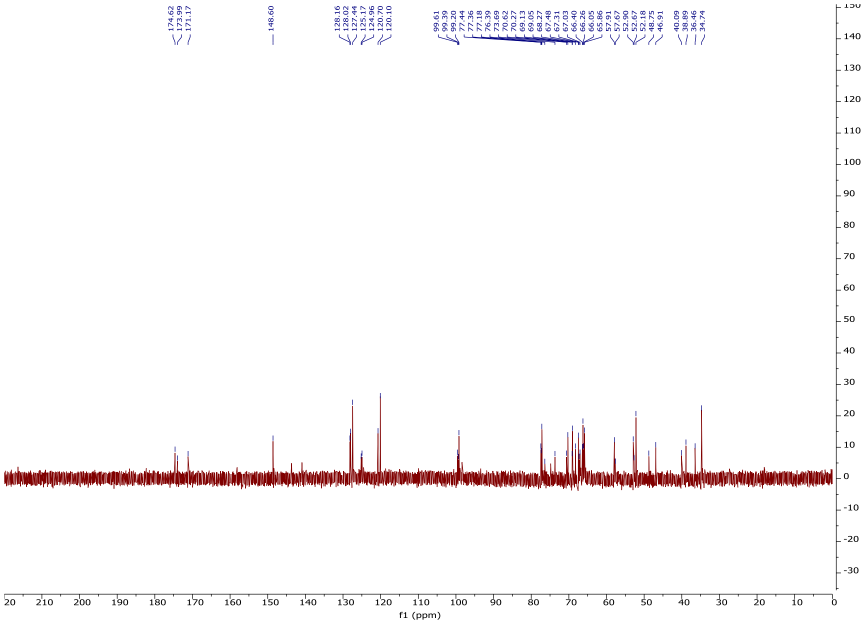

^{13}C-NMR of **32a** (125 MHz D_2O)

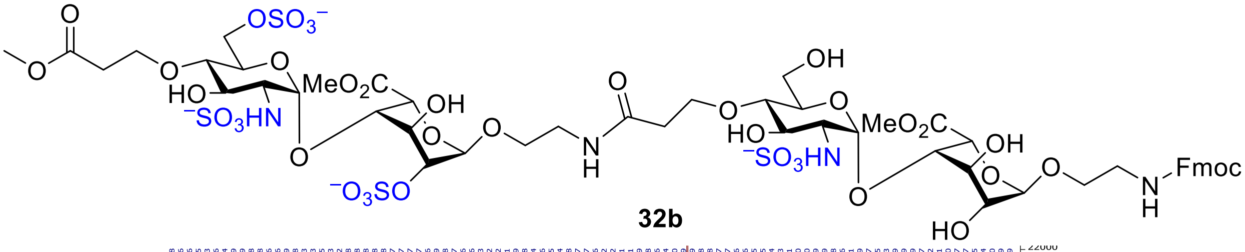

32b

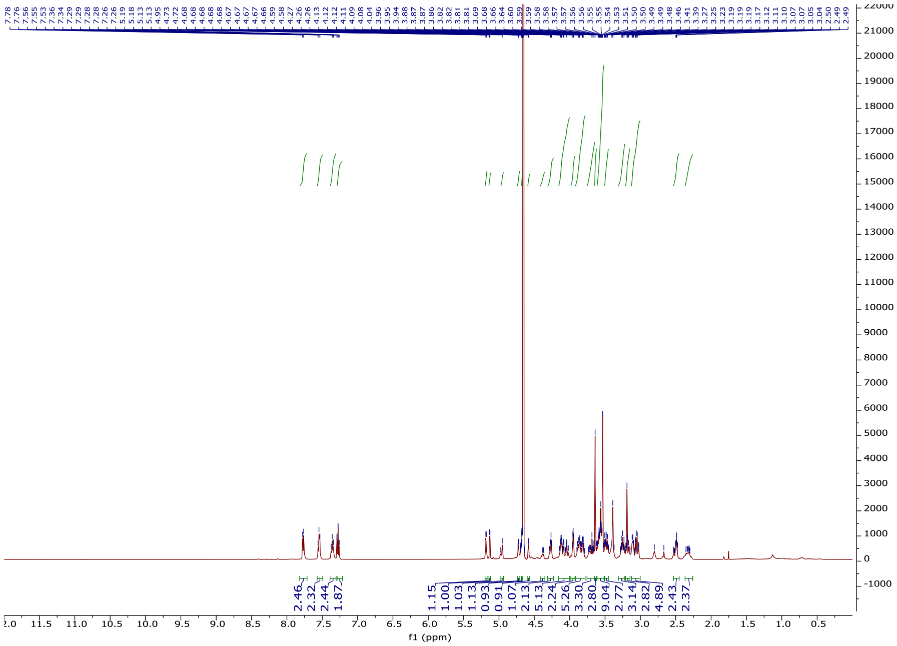

^{1}H-NMR of **32b** (500 MHz D_2O)

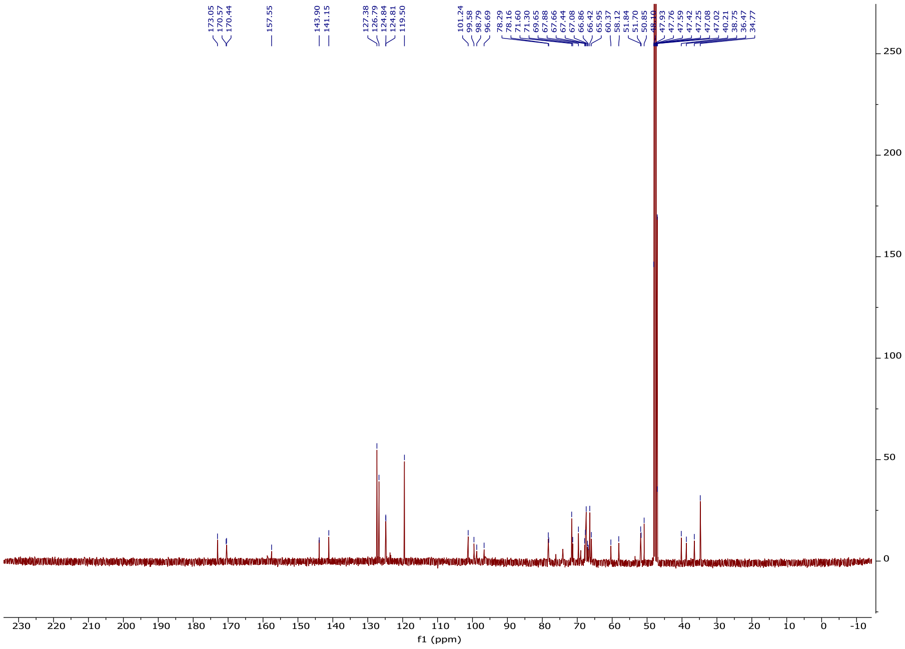

^{13}C-NMR of **32b** (125 MHz D_2O)

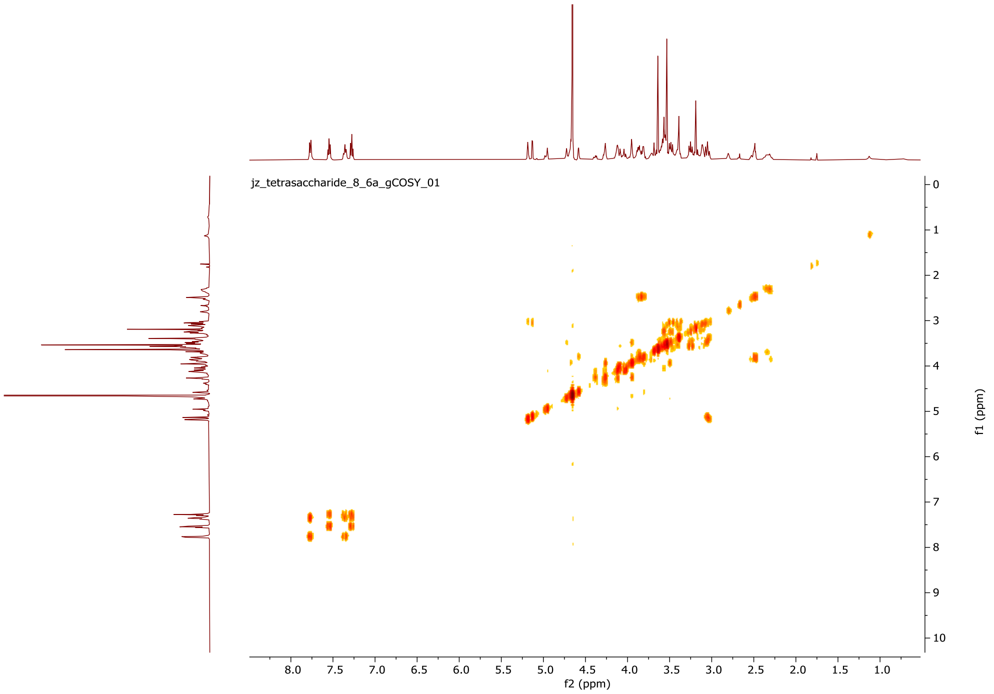

^1H-^1H gCOSY of **32b** (500 MHz D_2O)

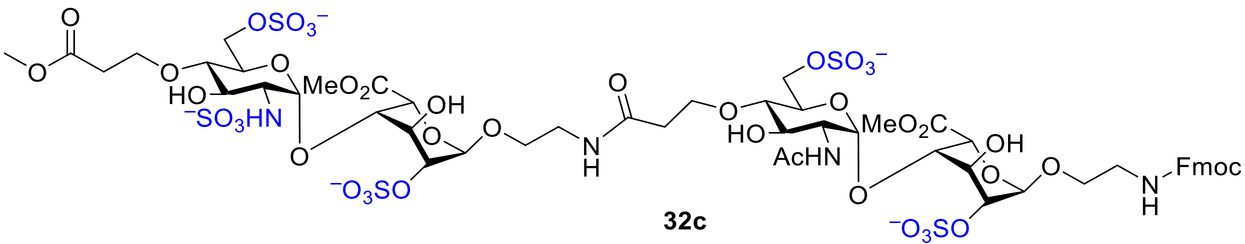

32c

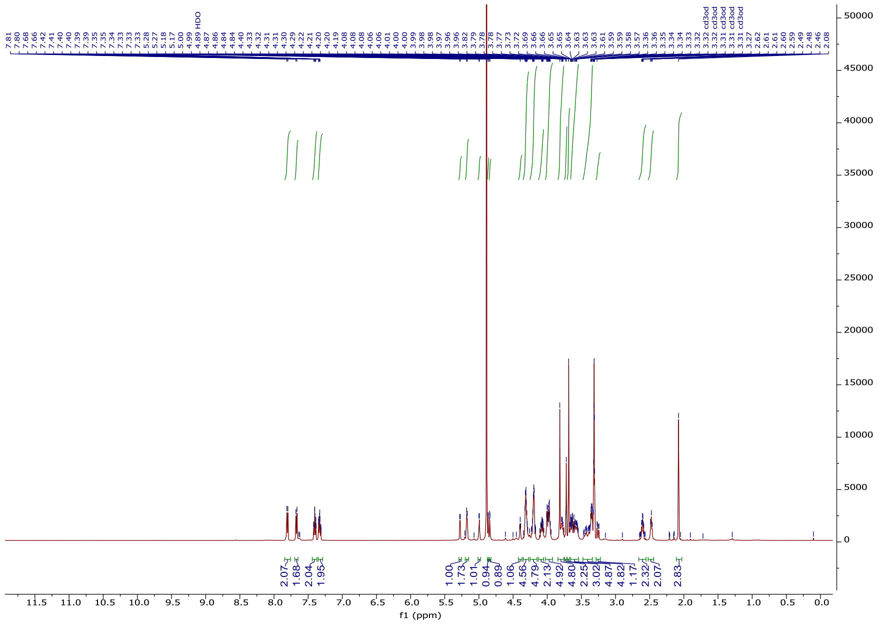

^{1}H-NMR of **32c** (500 MHz CD_3OD)

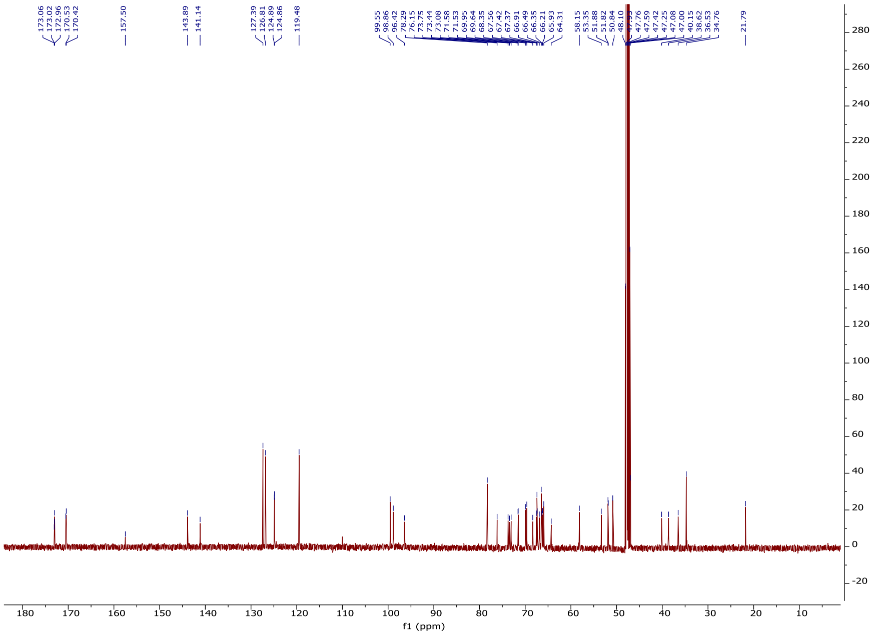

^{13}C-NMR of **32c** (125 MHz CD_3OD)

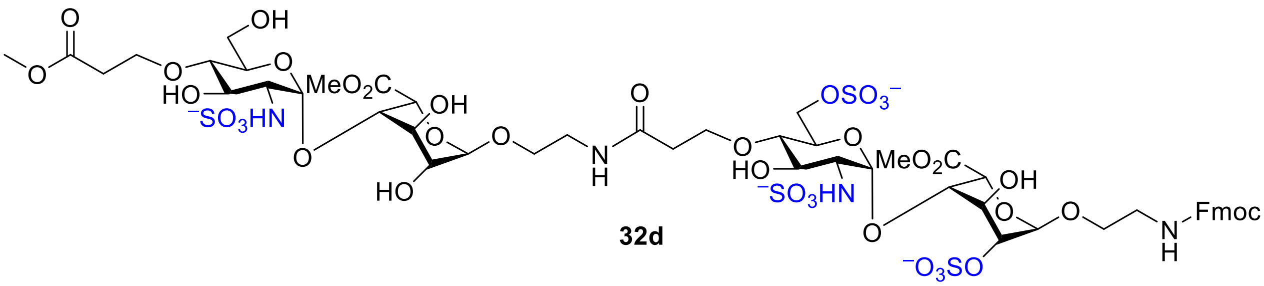

32d

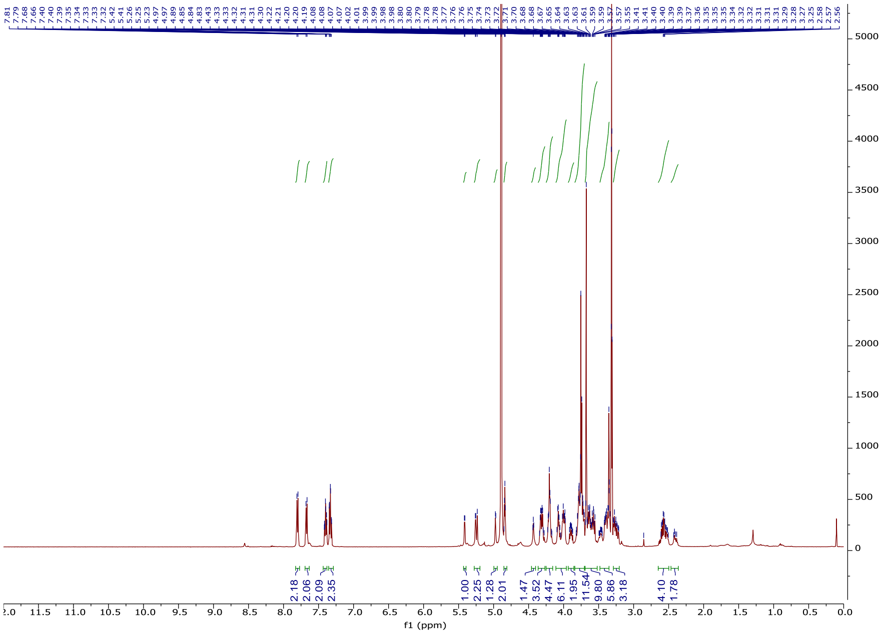

^{1}H-NMR of **32d** (500 MHz CD_3OD)

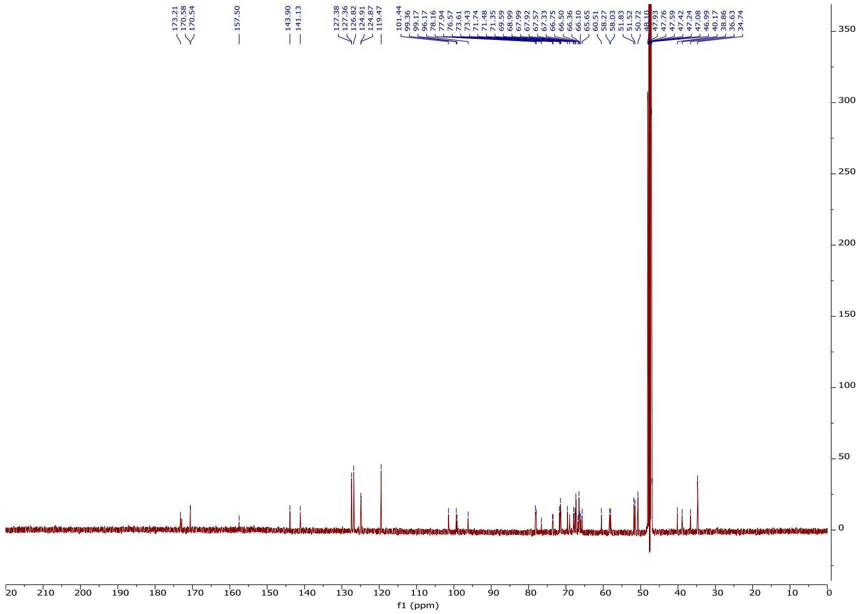

^{13}C-NMR of **32d** (125 MHz CD_3OD)

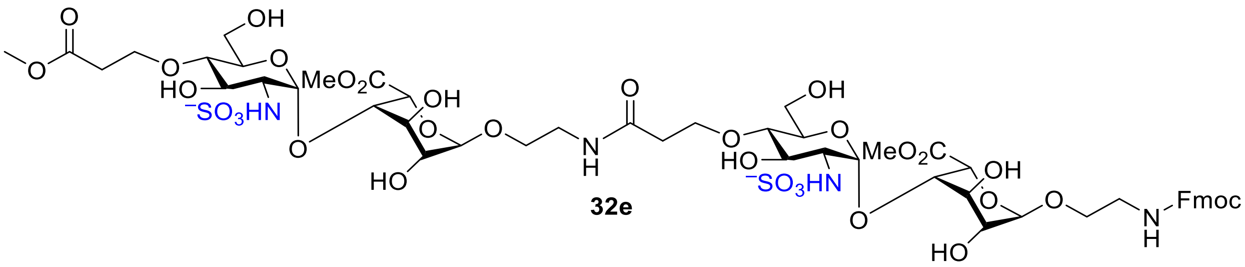

32e

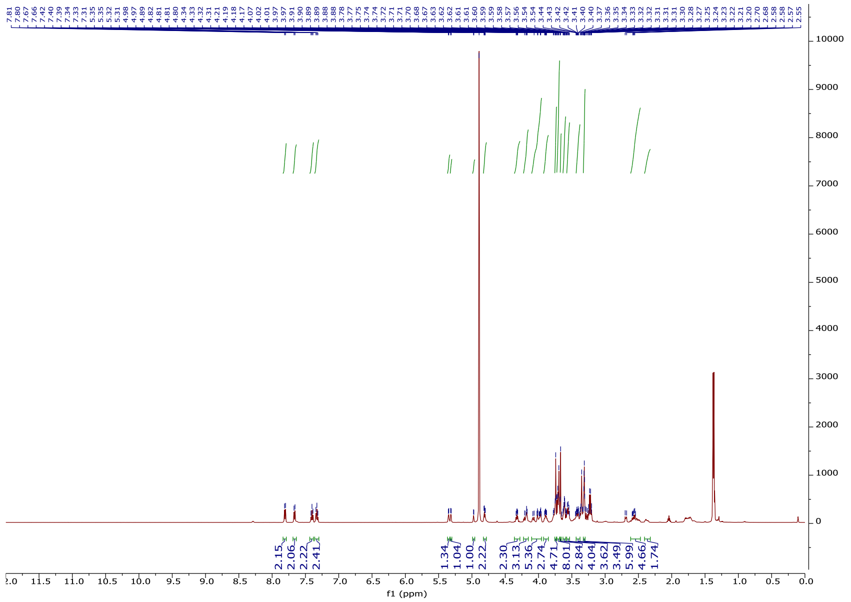

^{1}H-NMR of **32e** (500 MHz CD_3OD)

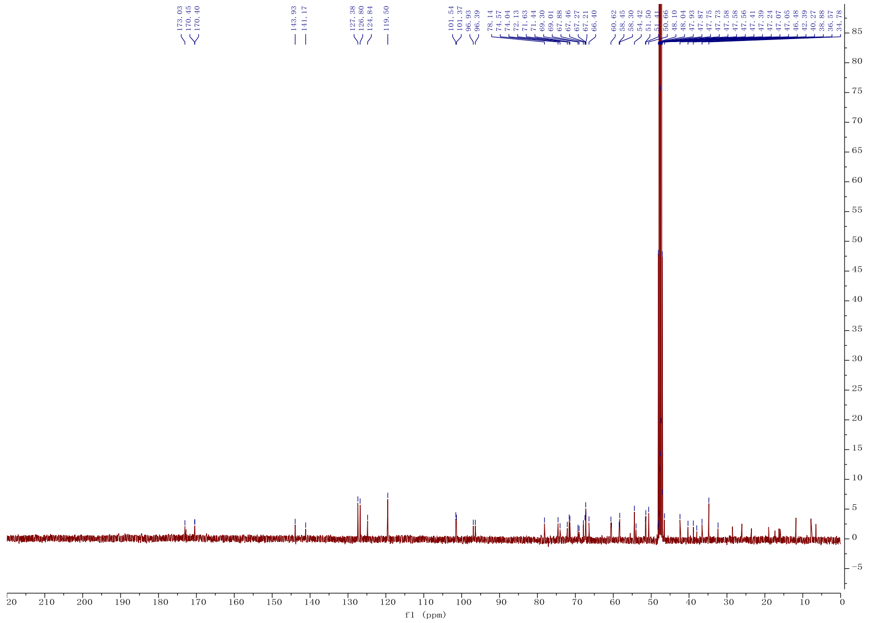

^{13}C-NMR of **32e** (125 MHz CD_3OD)

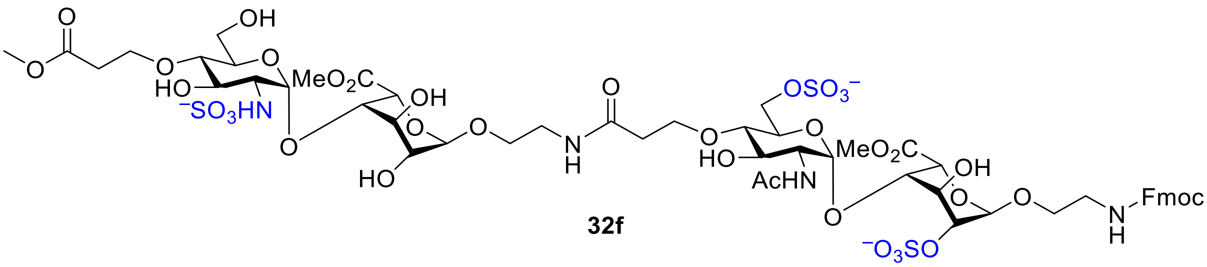

32f

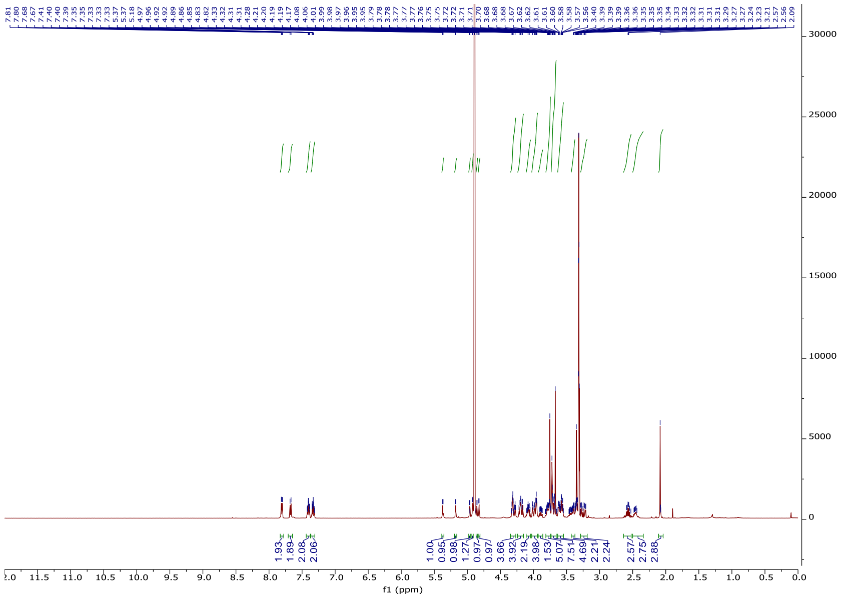

^{1}H-NMR of **32f** (500 MHz CD_3OD)

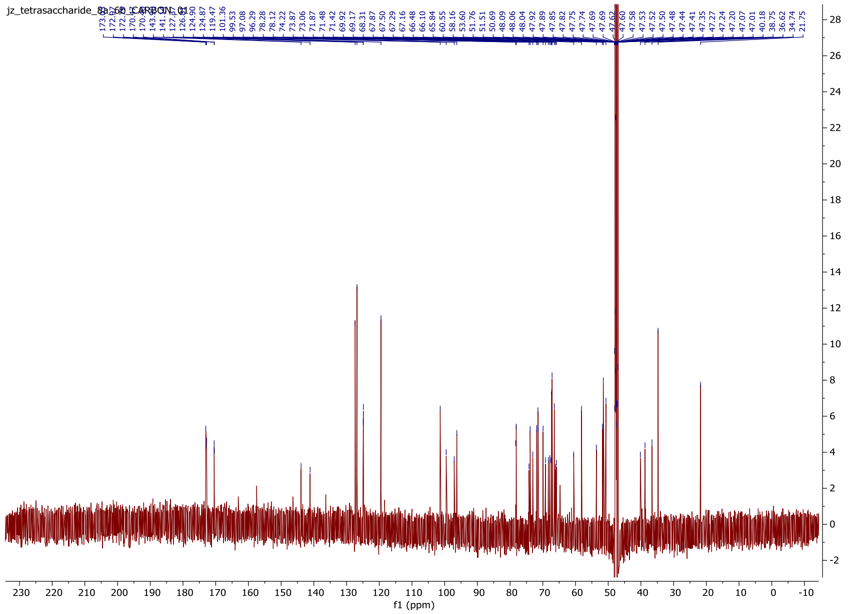

^{13}C-NMR of **32f** (125 MHz CD_3OD)

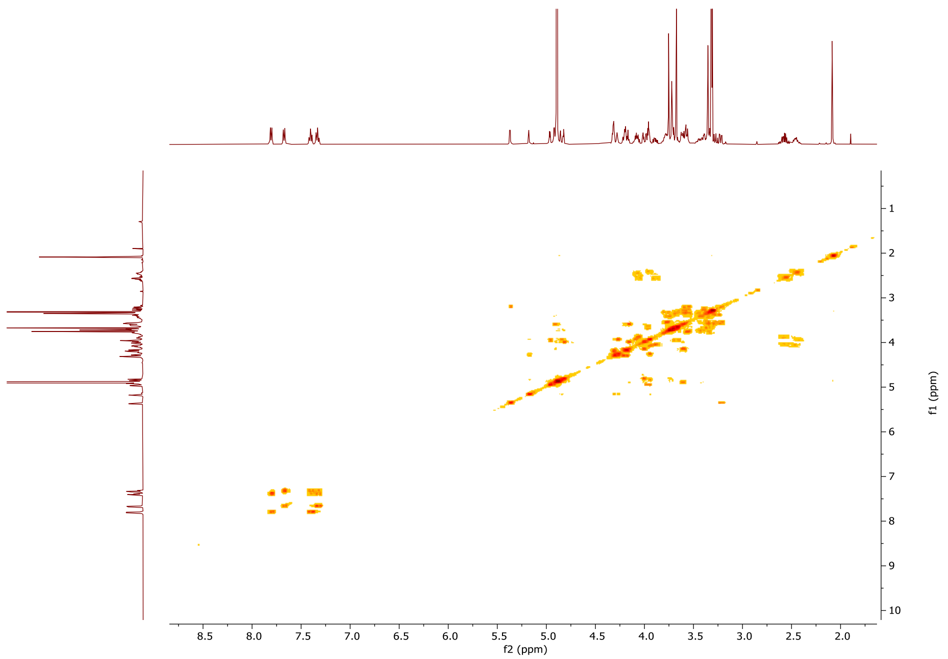

^{1}H-^{1}H gCOSY of **32f** (500 MHz CD_3OD)

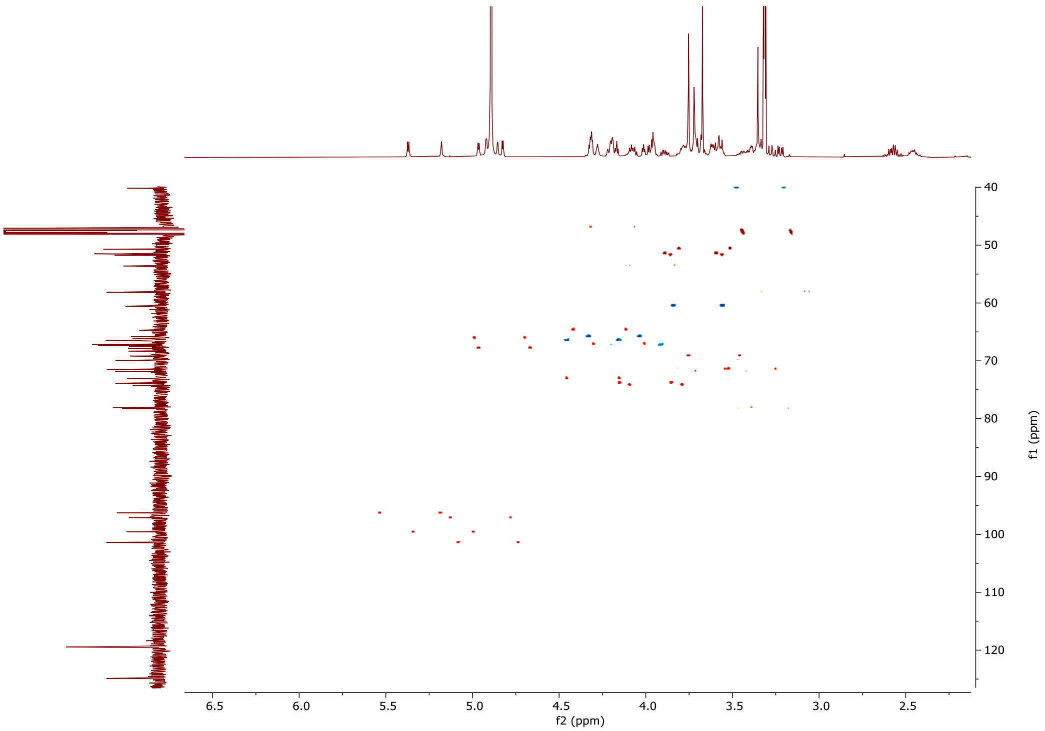

^{1}H-^{13}C gHSQCAD of **32f** (500 MHz CD_3OD)

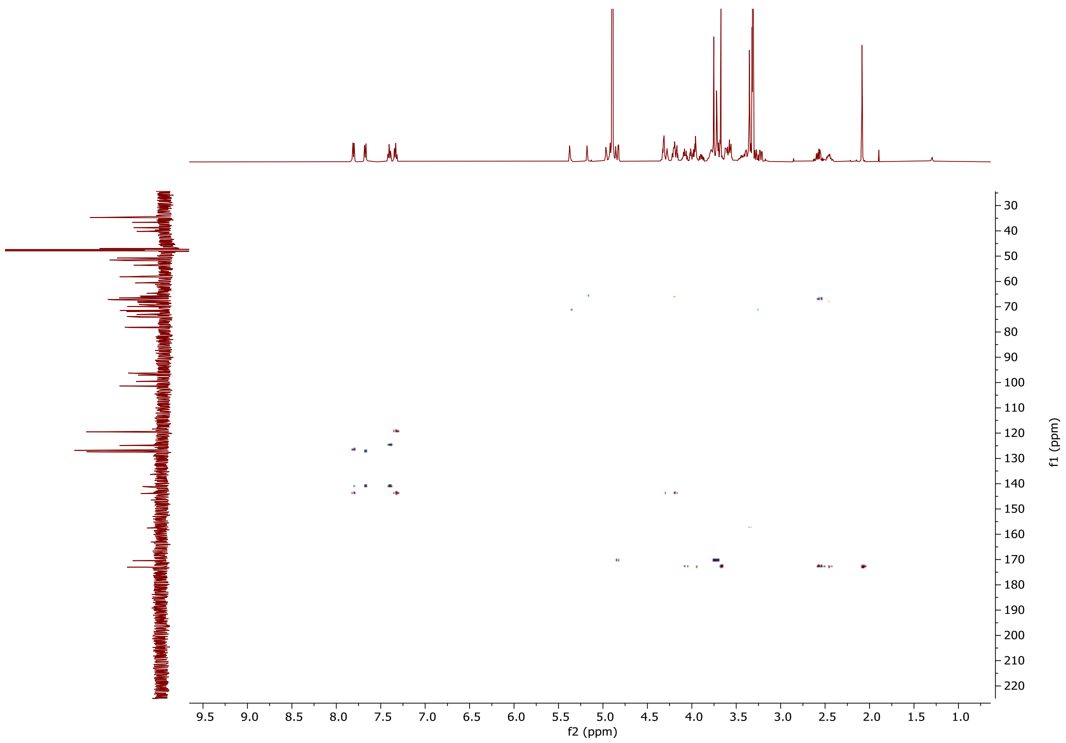

^1H-^{13}C gHMBCAD of **32f** (500 MHz CD_3OD)

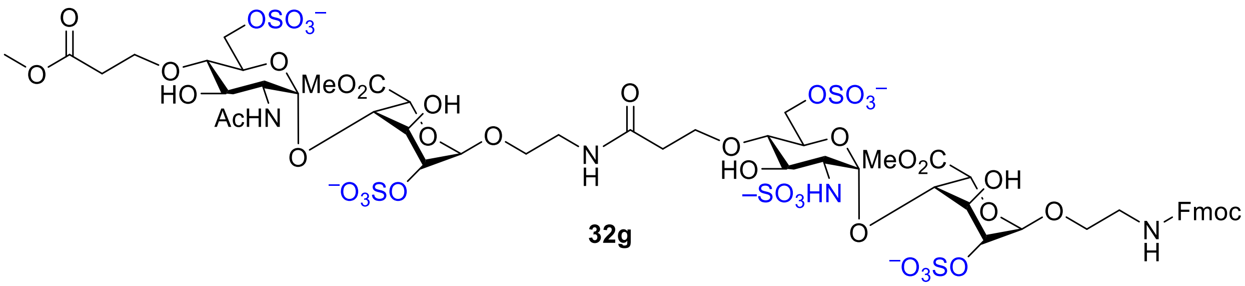

32g

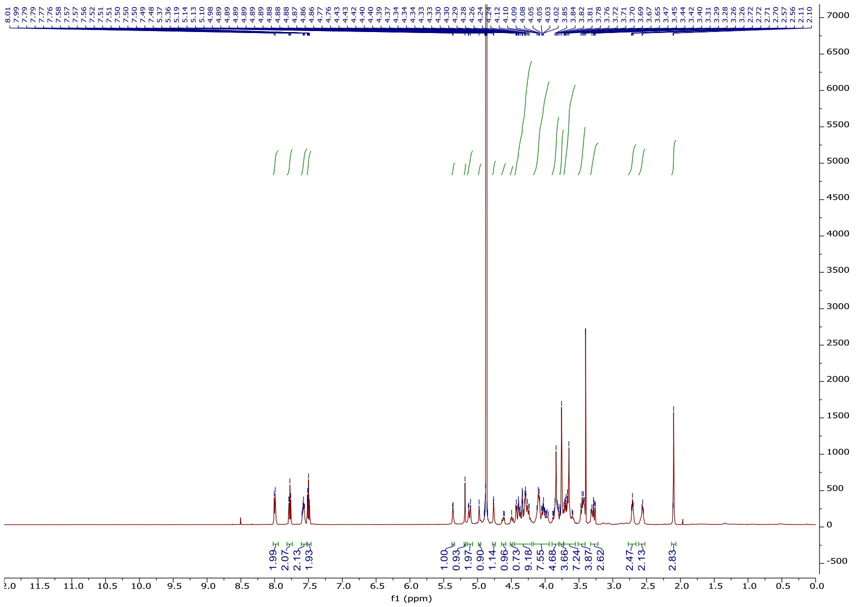

^{1}H-NMR of **32g** (500 MHz CD_3OD)

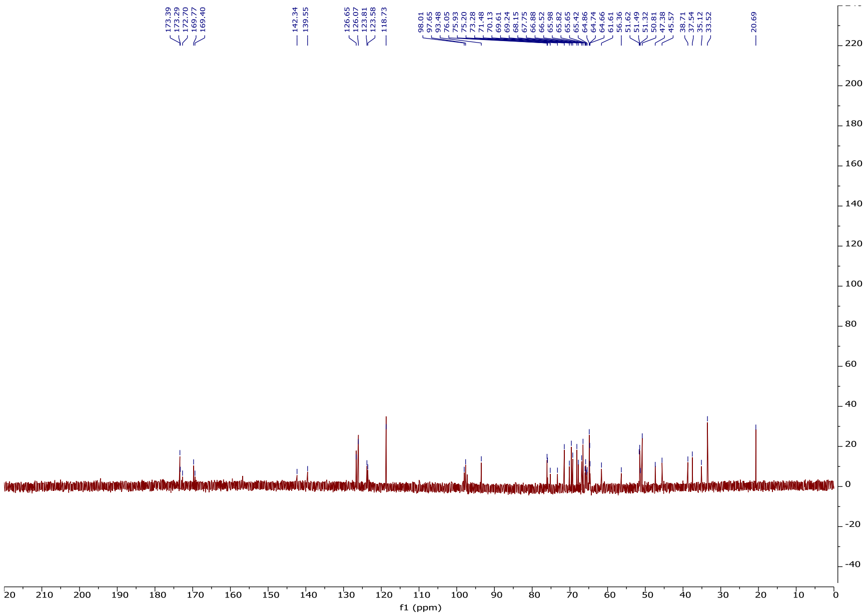

^{13}C-NMR of **32g** (125 MHz CD_3OD)

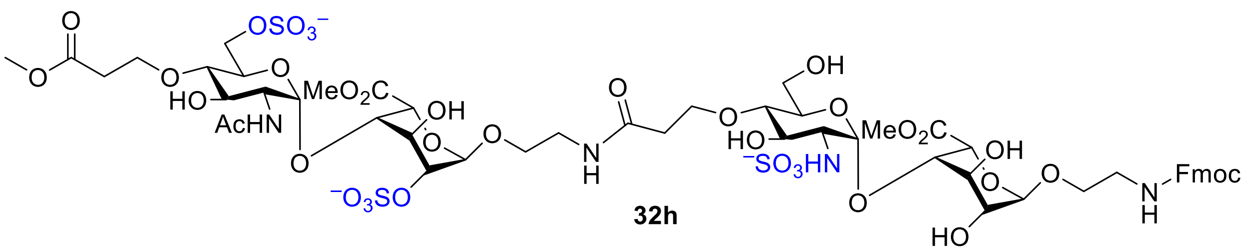

32h

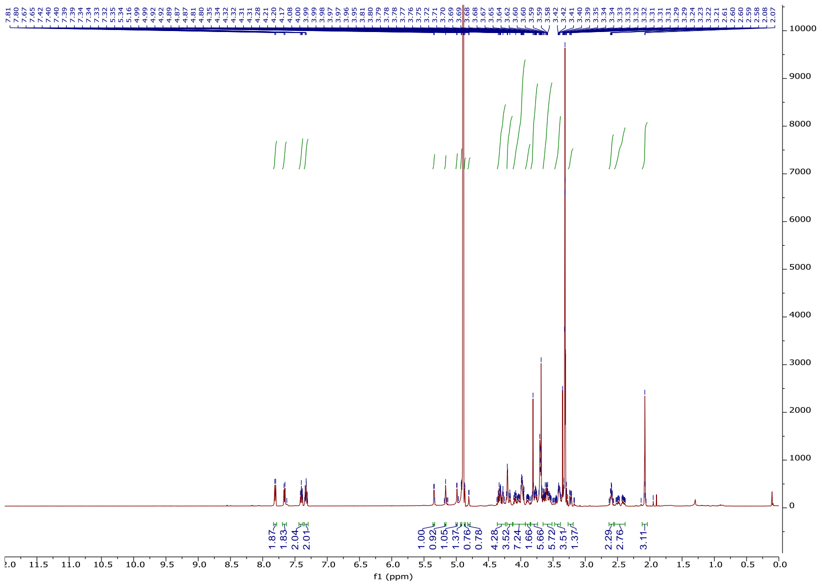

^{1}H-NMR of **32h** (500 MHz CD_3OD)

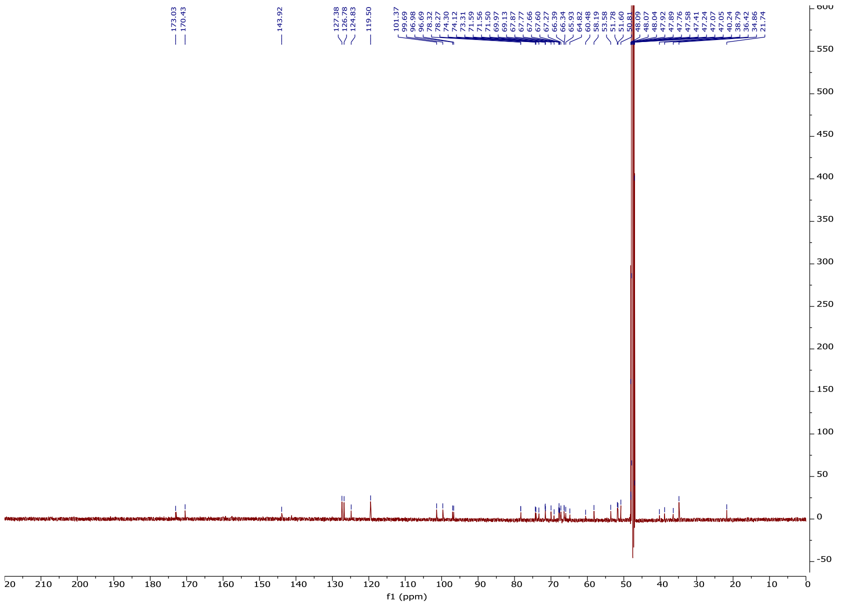

^{13}C-NMR of **32h** (125 MHz CD_3OD)

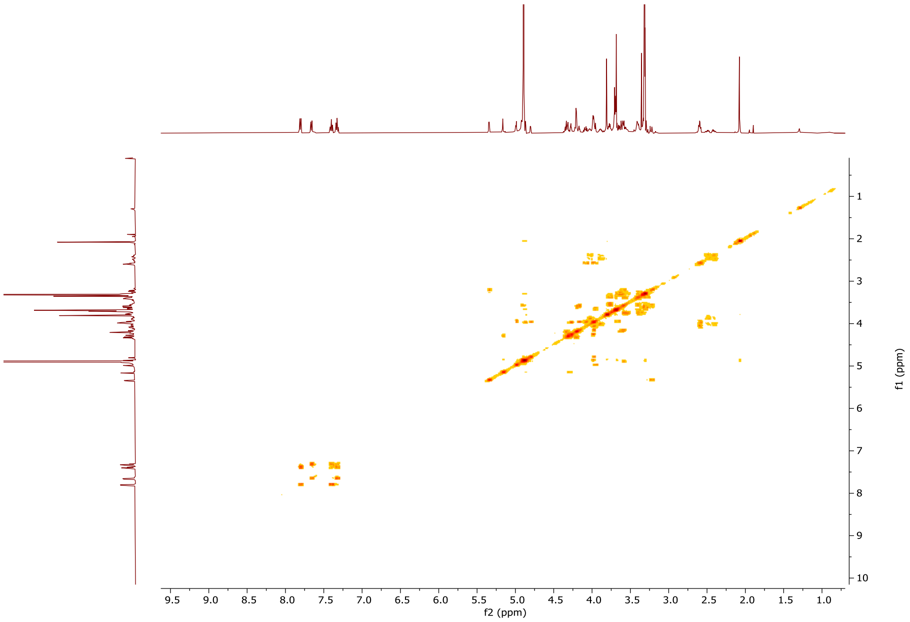

^{1}H-^{1}H gCOSY of **32h** (500 MHz CD_3OD)

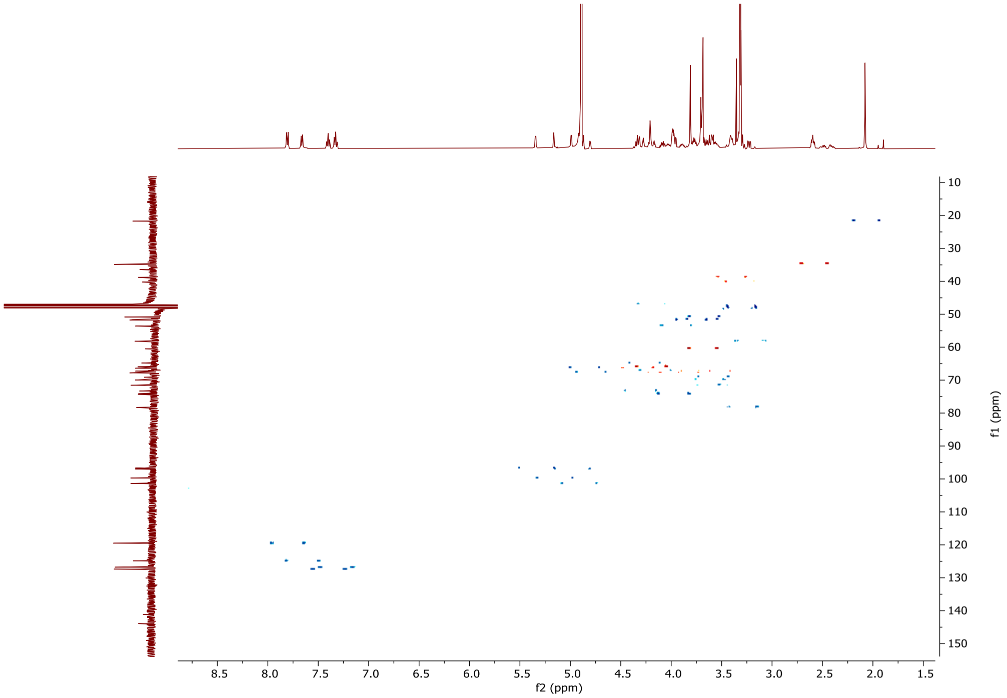

^{1}H-^{13}C gHSQCAD of **32h** (500 MHz CD_3OD)

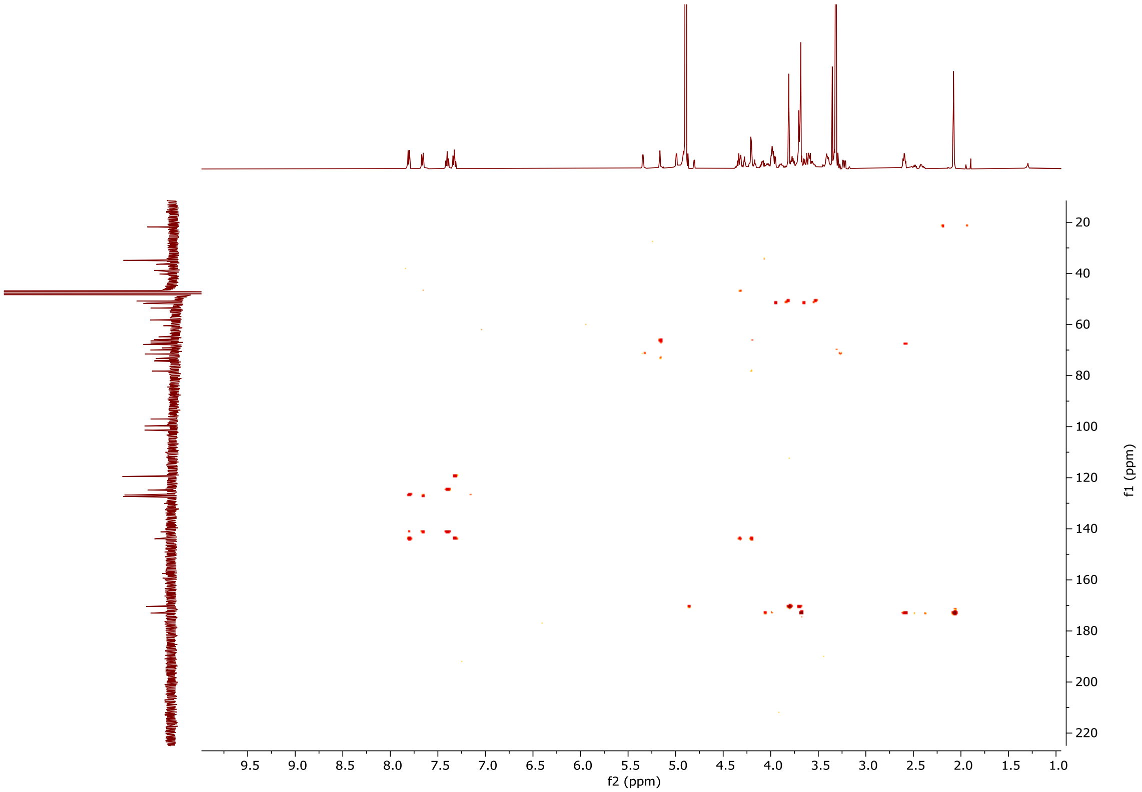

^{1}H-^{13}C gHMBCAD of **32h** (500 MHz CD_3OD)

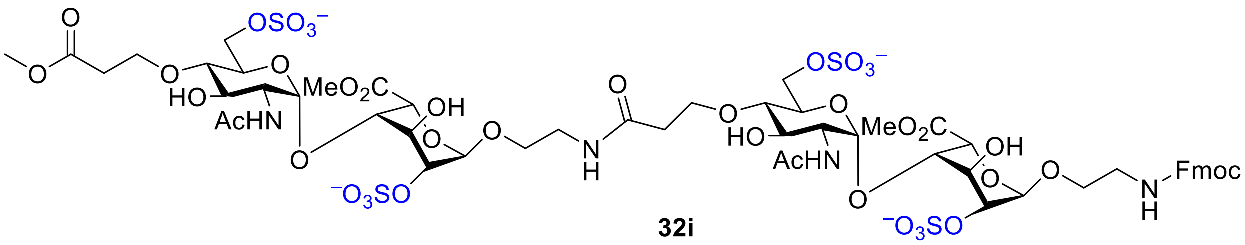

32i

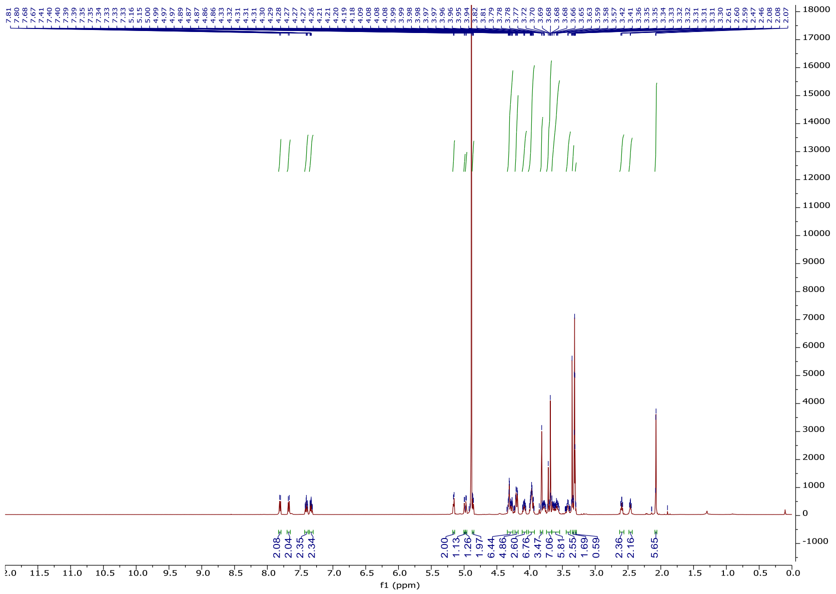

^{1}H-NMR of **32i** (500 MHz CD_3OD)

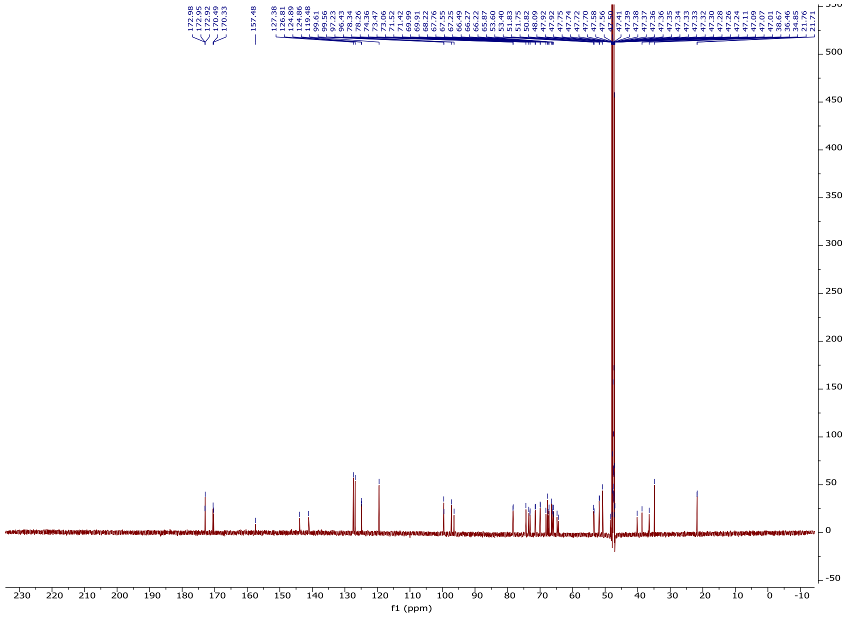

^{13}C-NMR of **32i** (125 MHz CD_3OD)

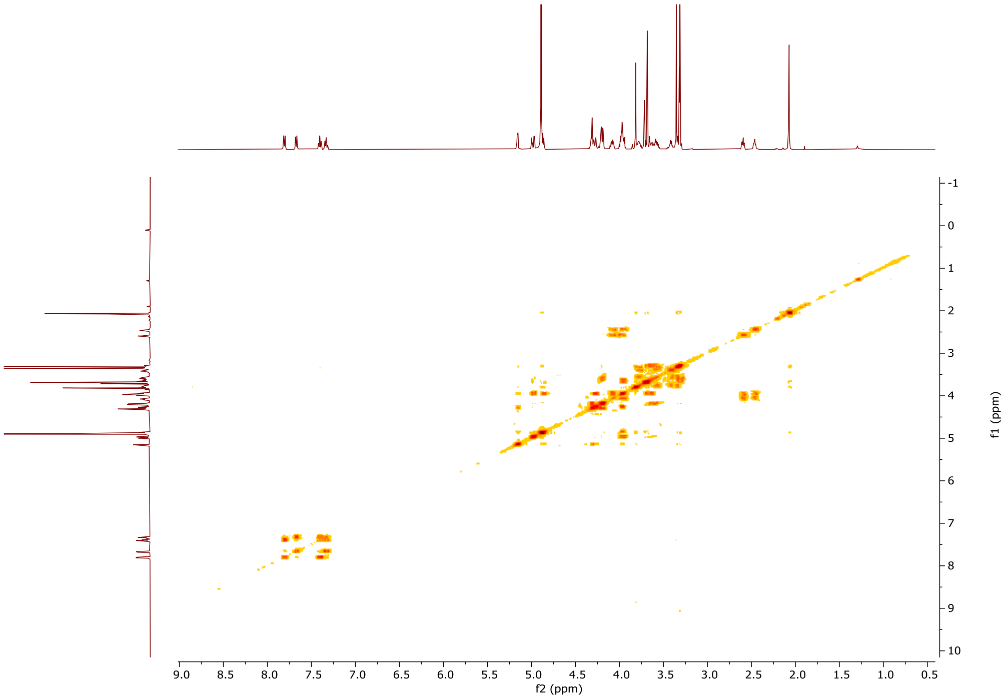

^{1}H-^{1}H gCOSY of **32i** (500 MHz CD_3OD)

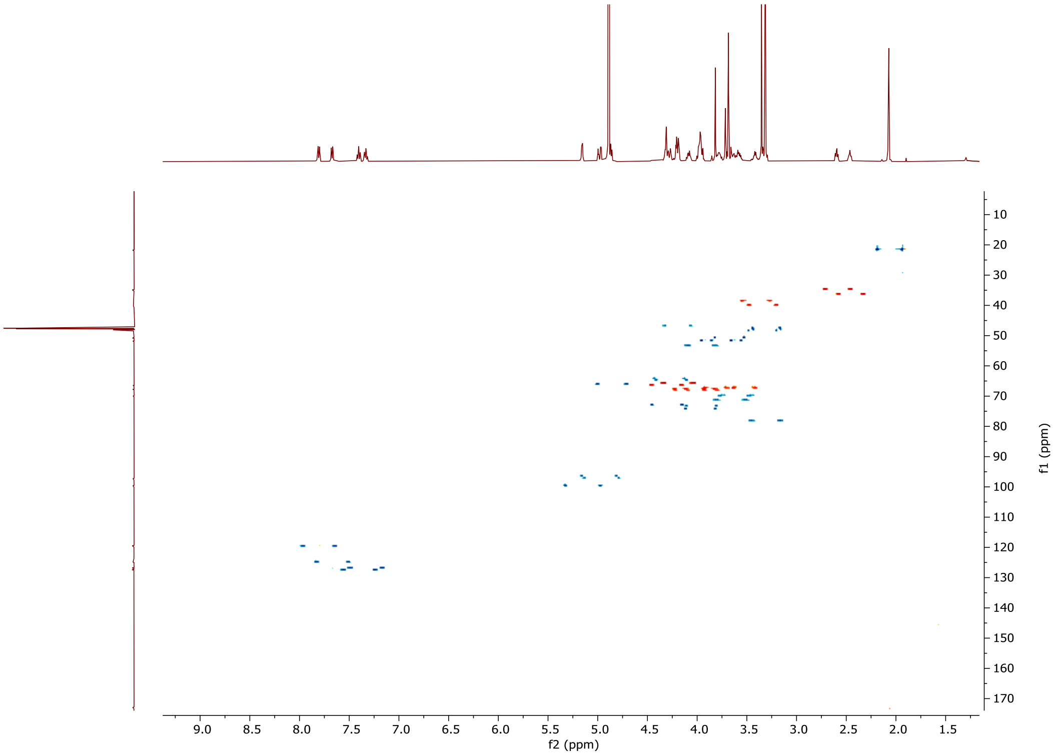

^{1}H-^{13}C gHSQCAD of **32i** (500 MHz CD_3OD)

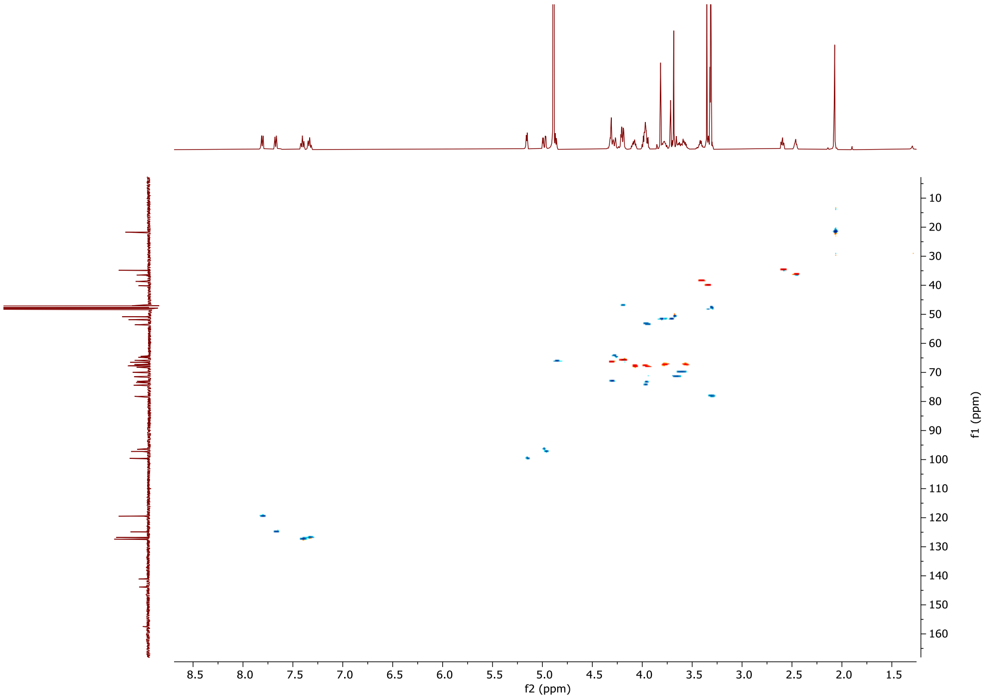

^{1}H-^{13}C gHSQCAD of **32i** (500 MHz CD_3OD)

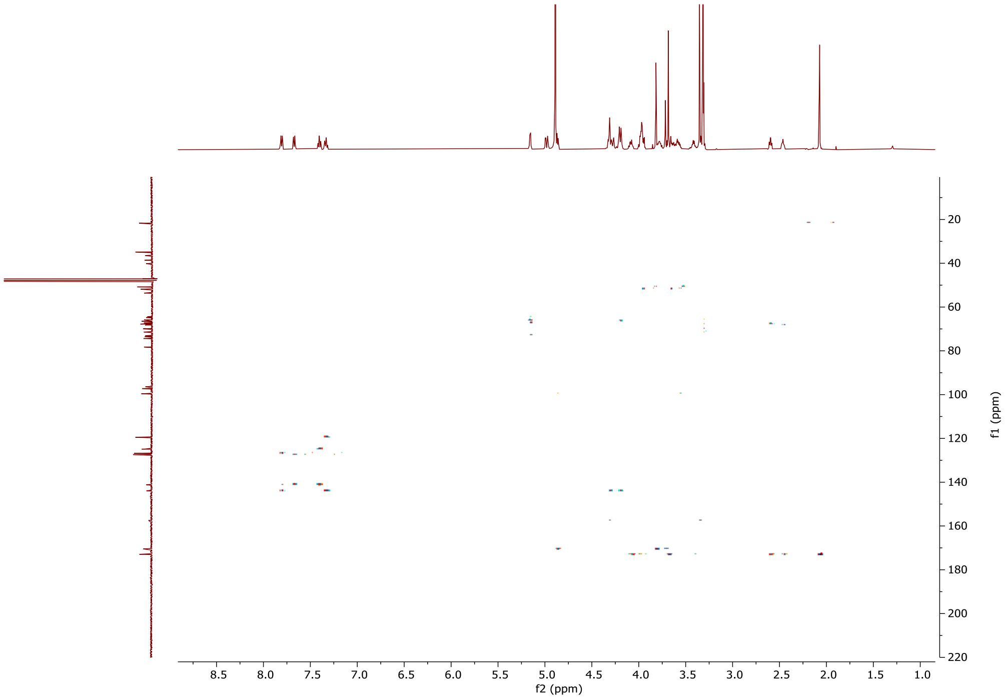

^{1}H-^{13}C gHMBCAD of **32i** (500 MHz CD_3OD)

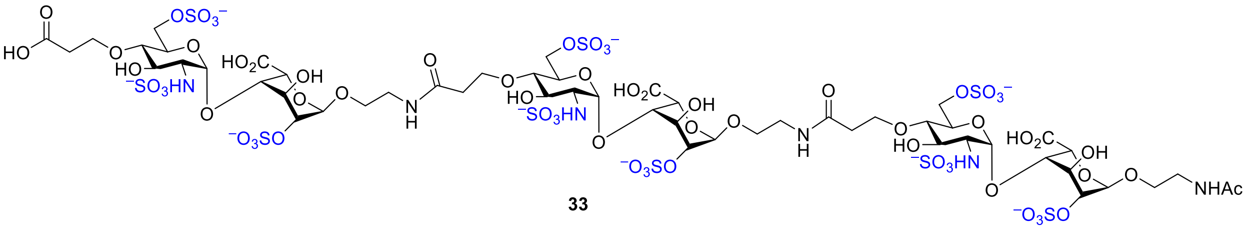

33

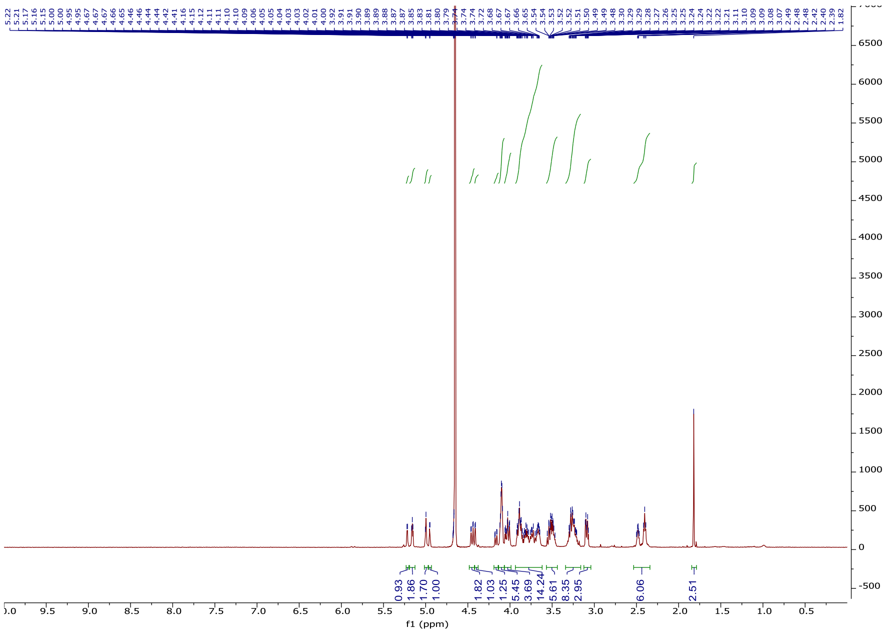

^{1}H-NMR of **33** (500 MHz D_2O)

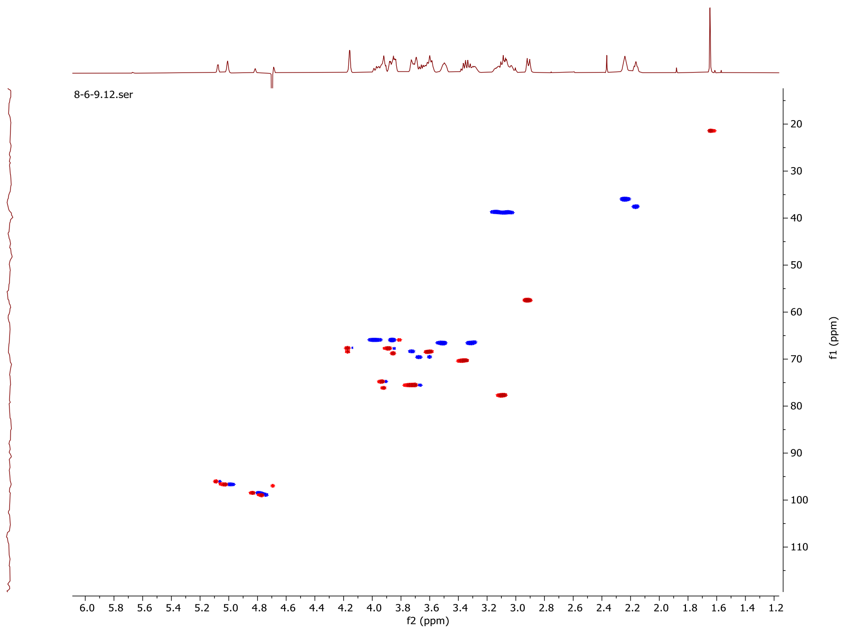

^{1}H-^{13}C gHSQCAD of **33** (600 MHz D_2O)

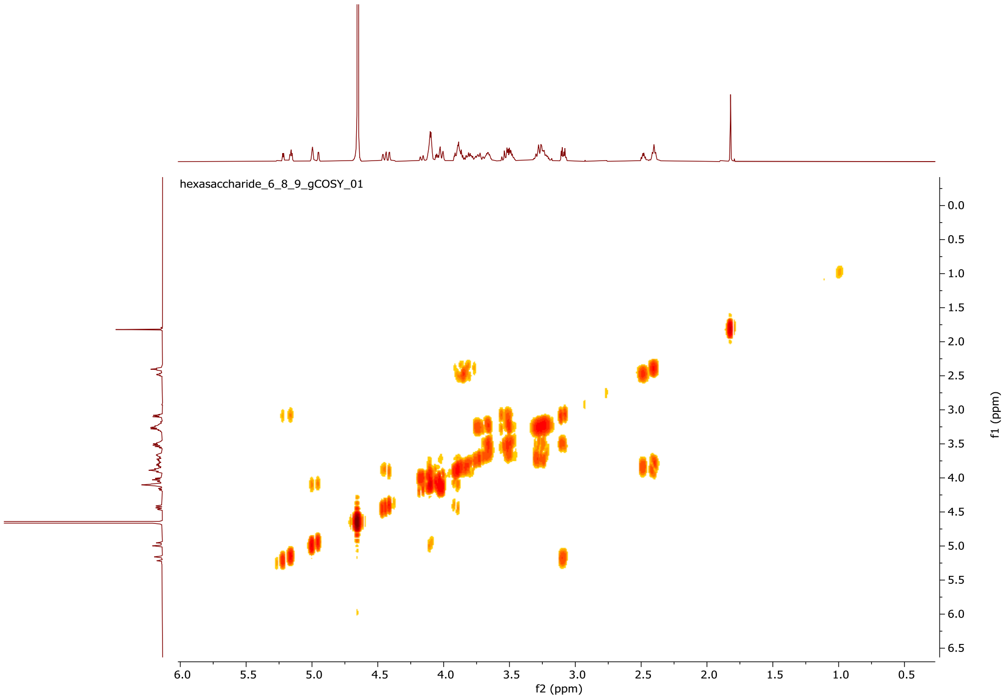

^1H-^1H gCOSY of **33** (500 MHz D_2O)

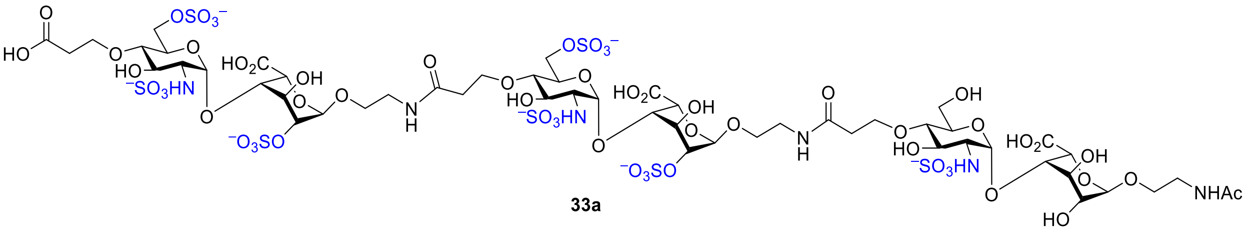

33a

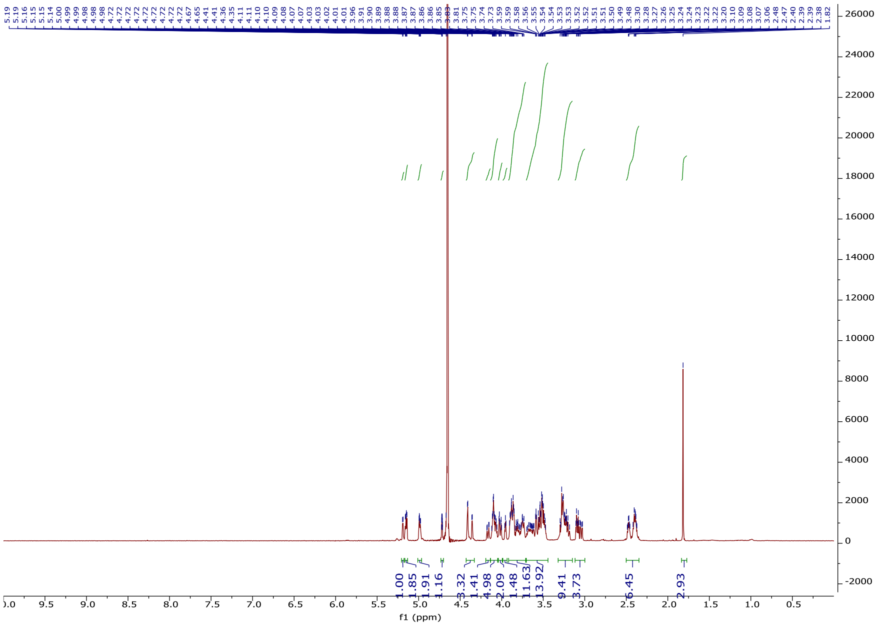

^{1}H-NMR of **33a** (500 MHz D_2O)

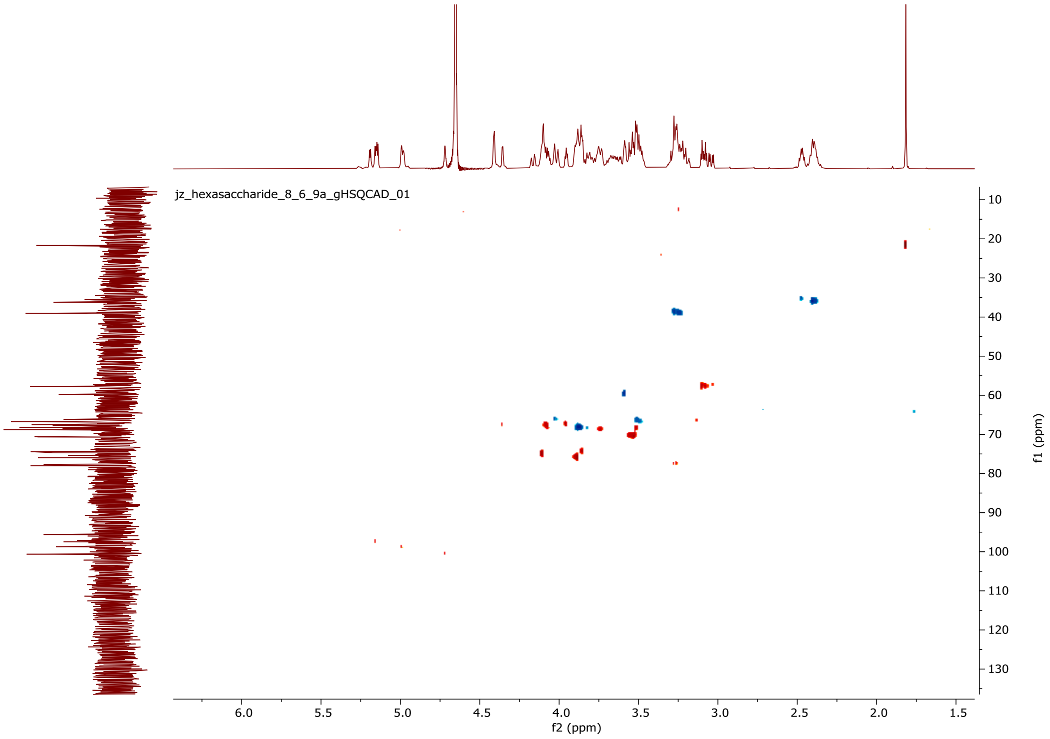

^{1}H-^{13}C gHSQCAD of **33a** (500 MHz D_2O)

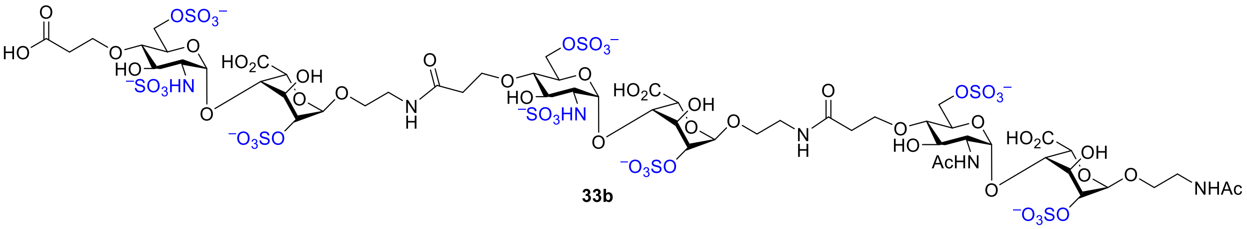

33b

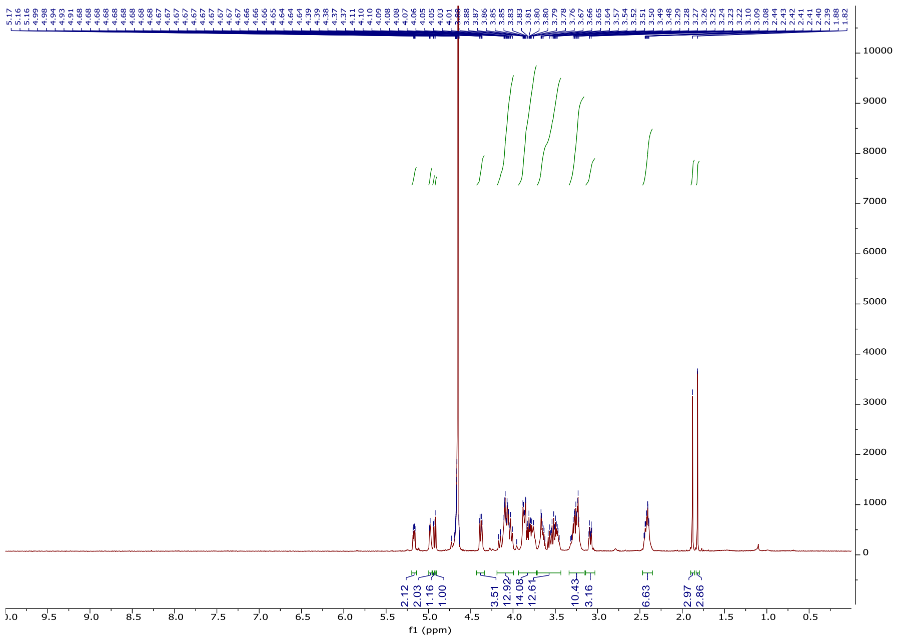

^{1}H-NMR of **33b** (500 MHz D_2O)

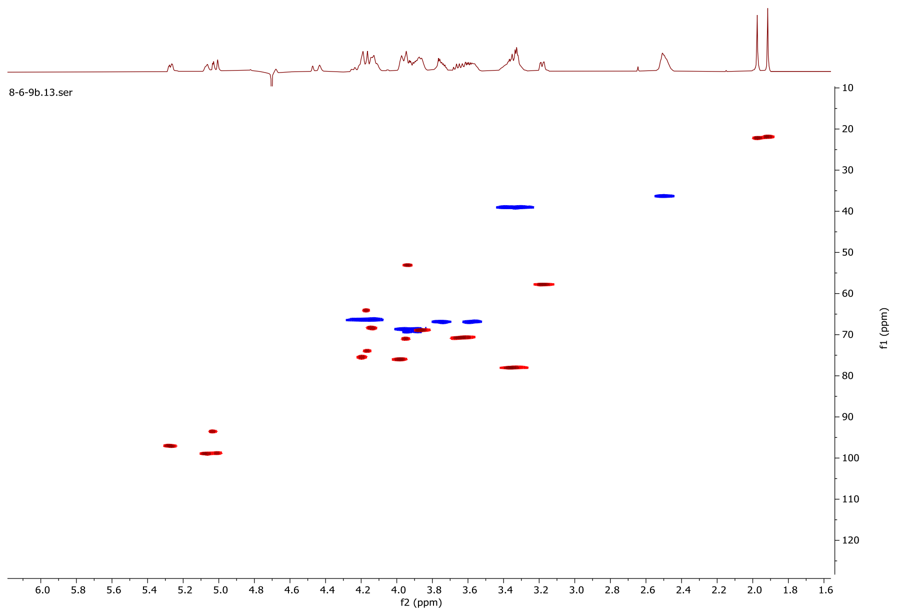

^{1}H-^{13}C gHSQCAD of **33b** (600 MHz D_2O)

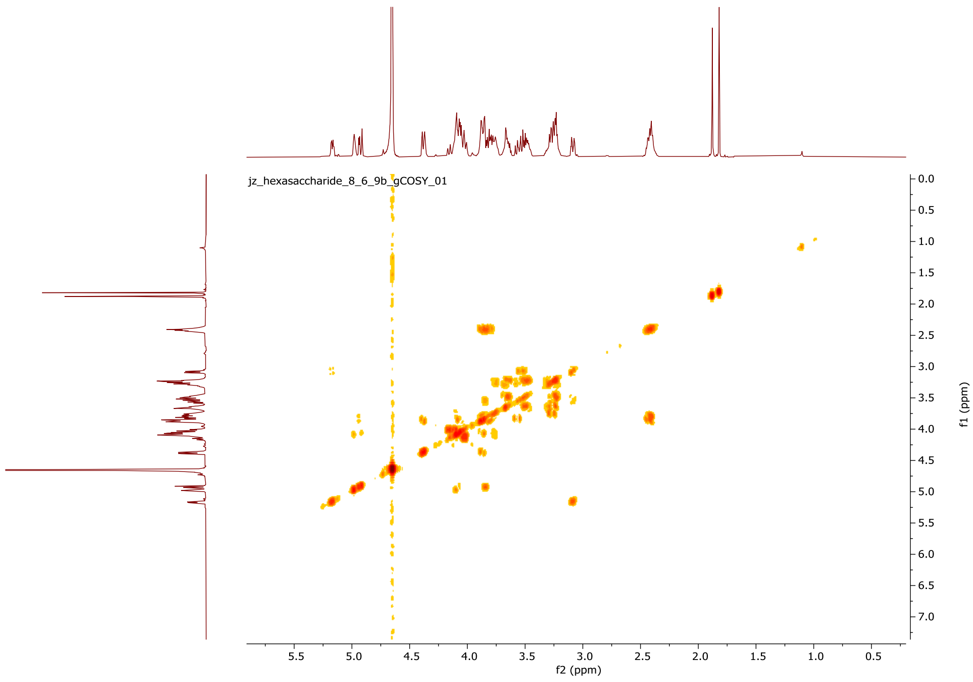

^{1}H-^{1}H gCOSY of **33b** (500 MHz D_2O)

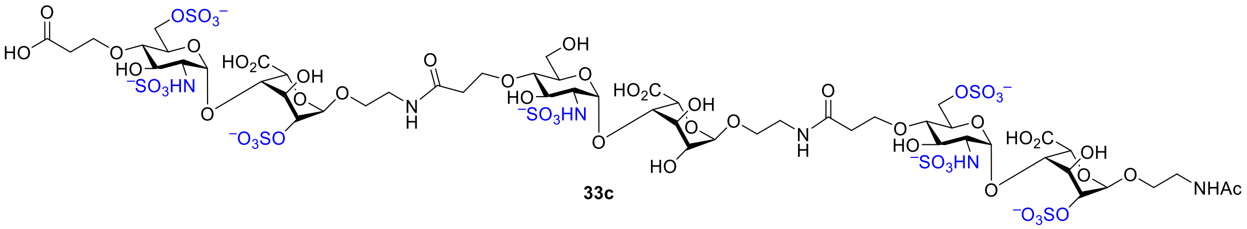

33c

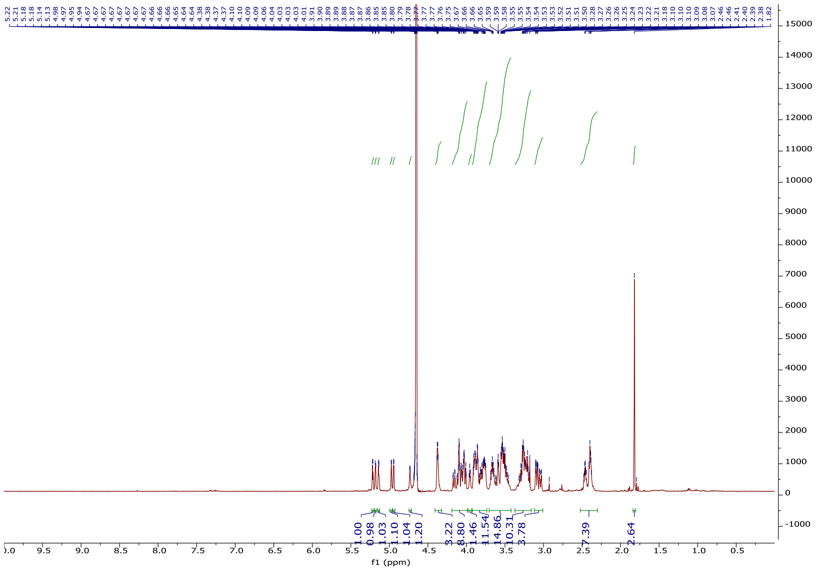

^{1}H-NMR of **33c** (500 MHz D_2O)

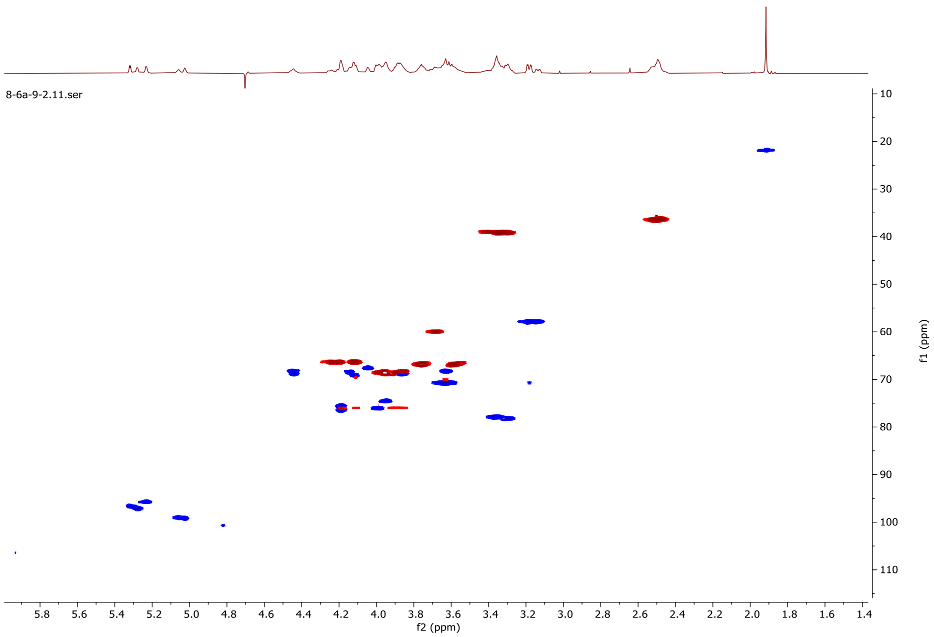

^{1}H-^{13}C gHSQCAD of **33c** (600 MHz D_2O)

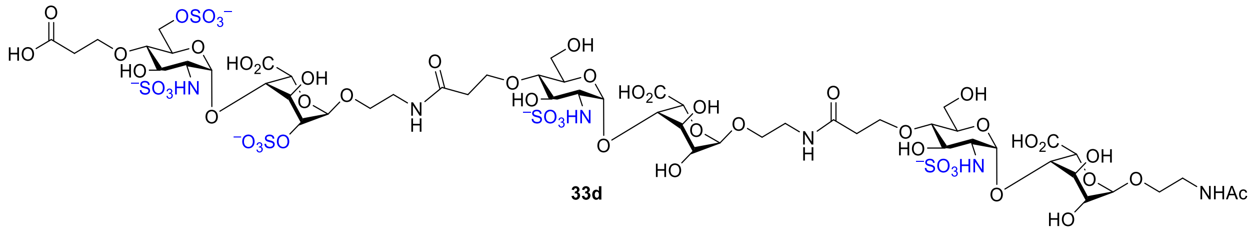

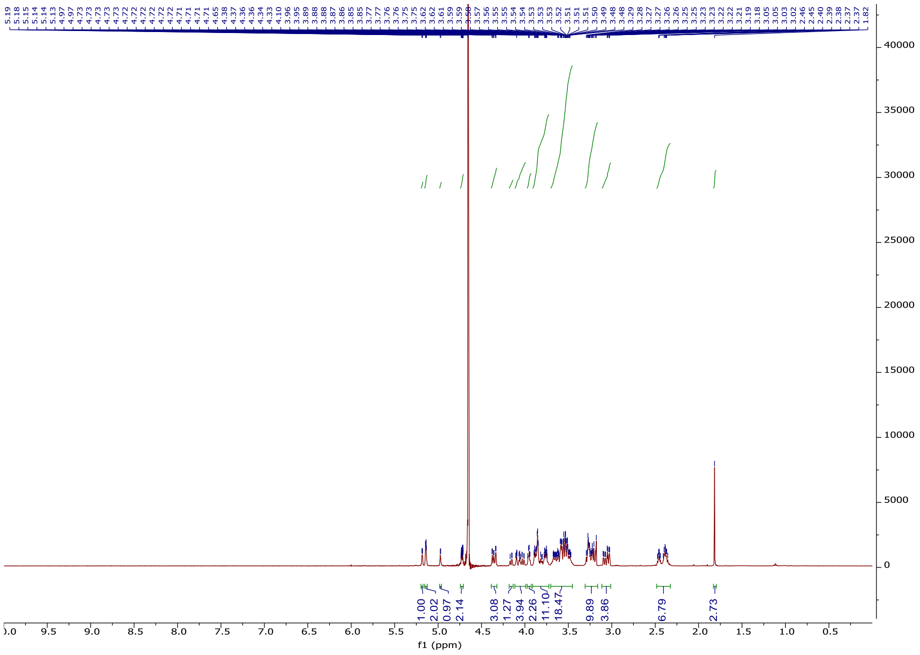

^{1}H-NMR of **33d** (500 MHz D_2O)

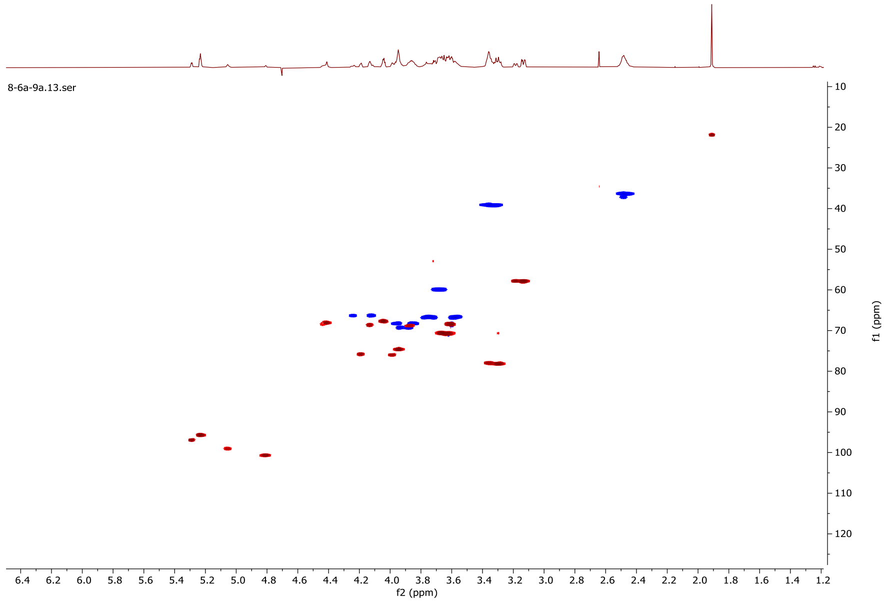

^{1}H-^{13}C gHSQCAD of **33d** (600 MHz D_2O)

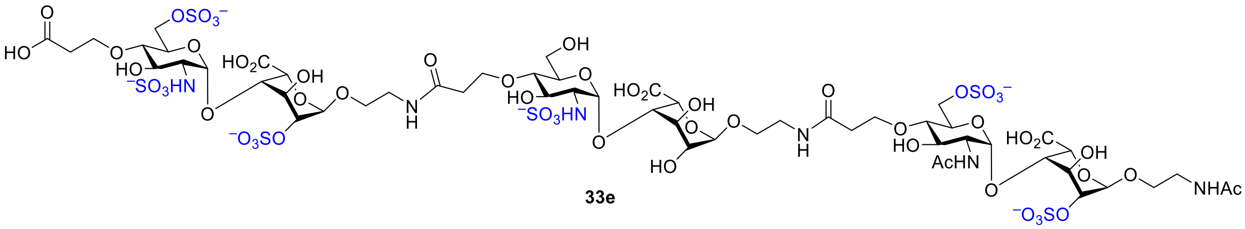

33e

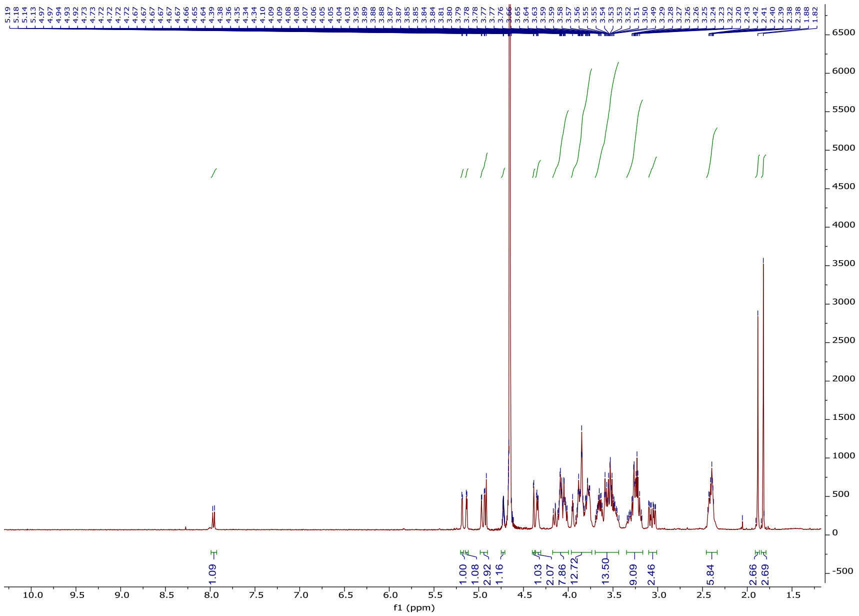

^{1}H-NMR of **33e** (500 MHz D_2O)

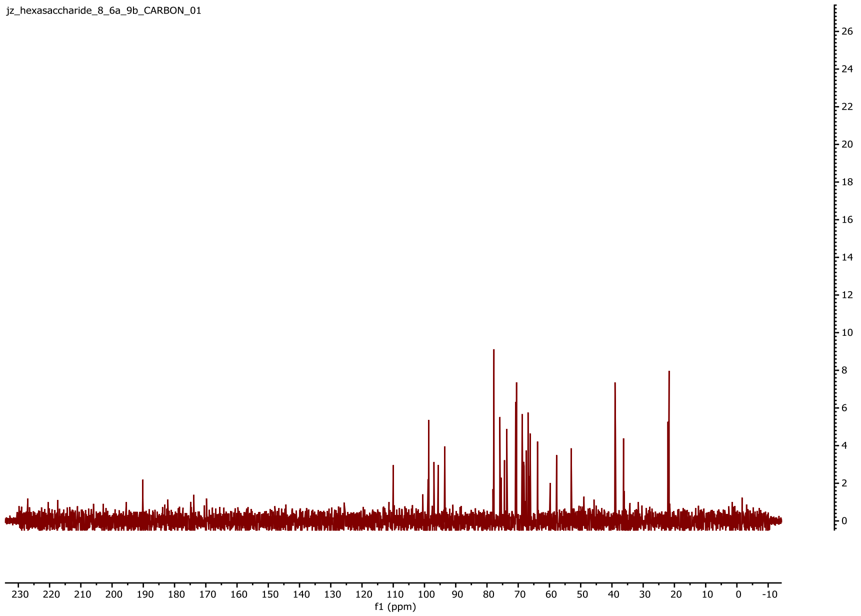

^{13}C-NMR of **33e** (125 MHz D_2O)

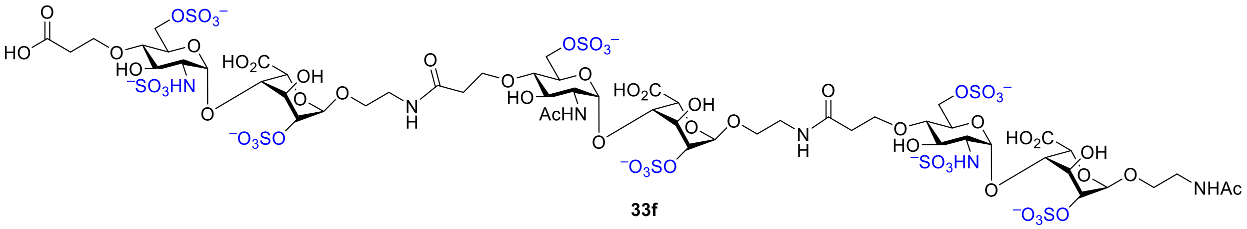

33f

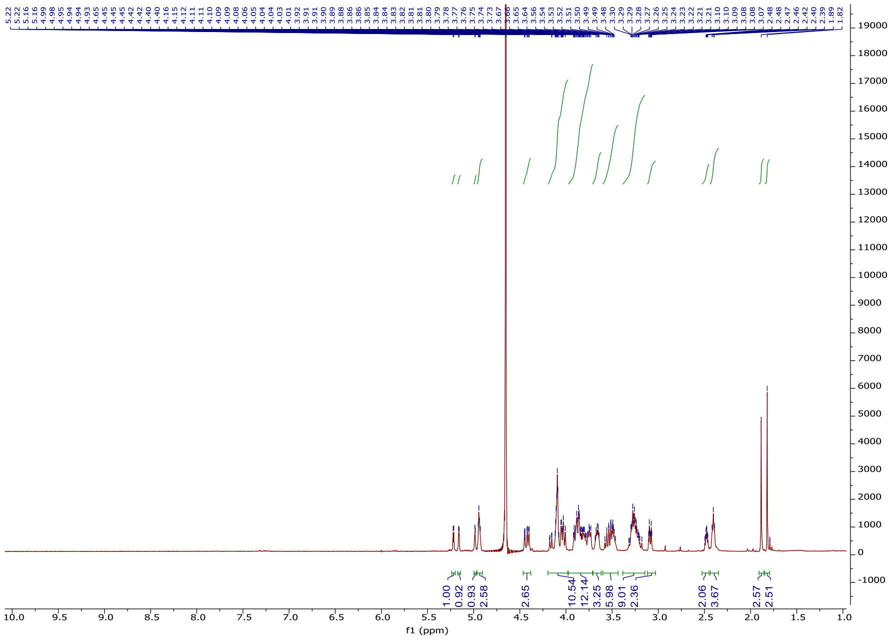

^{1}H-NMR of **33f** (500 MHz D_2O)

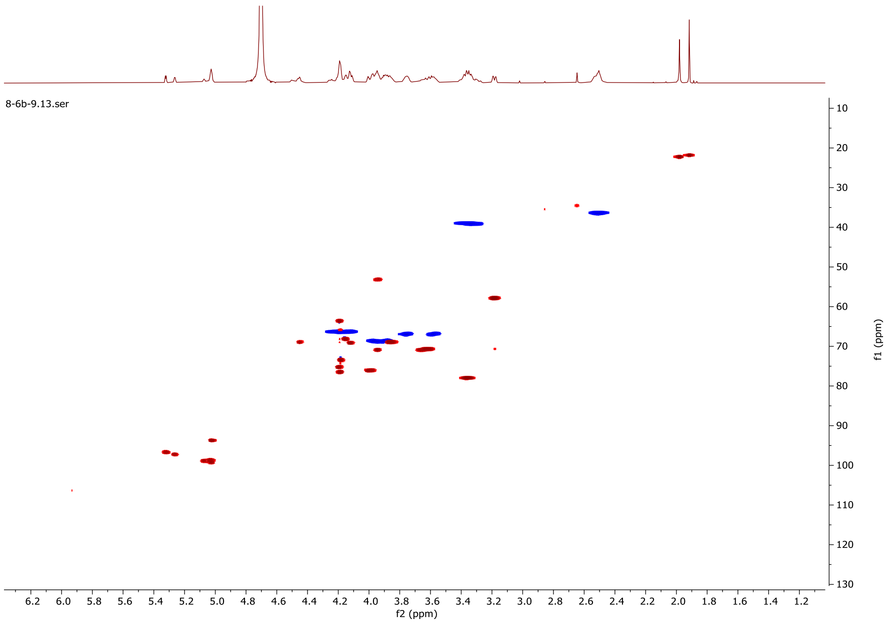

^{1}H-^{13}C gHSQCAD of **33f** (600 MHz D_2O)

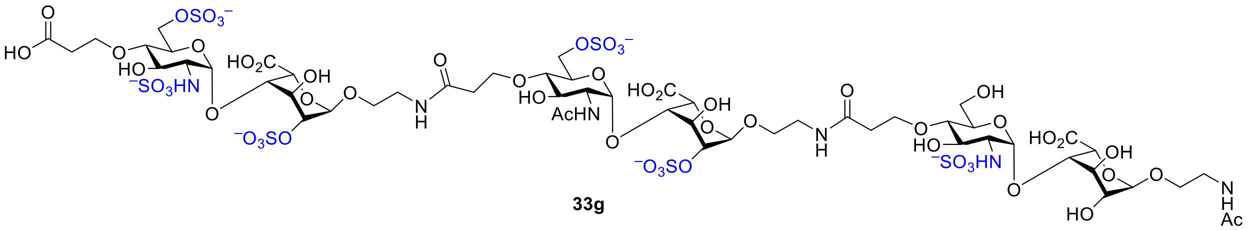

33g

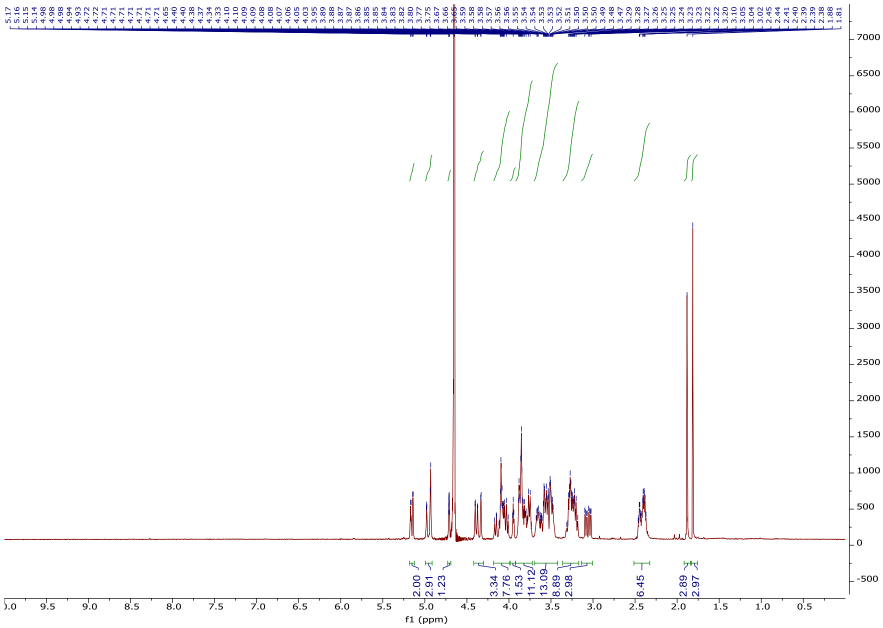

^{1}H-NMR of **33g** (500 MHz D_2O)

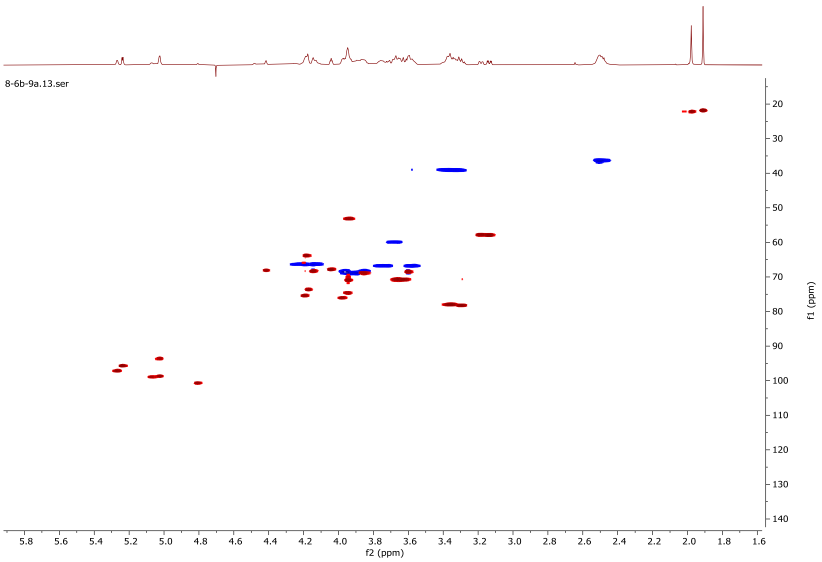

^{1}H-^{13}C gHSQCAD of **33g** (600 MHz D_2O)

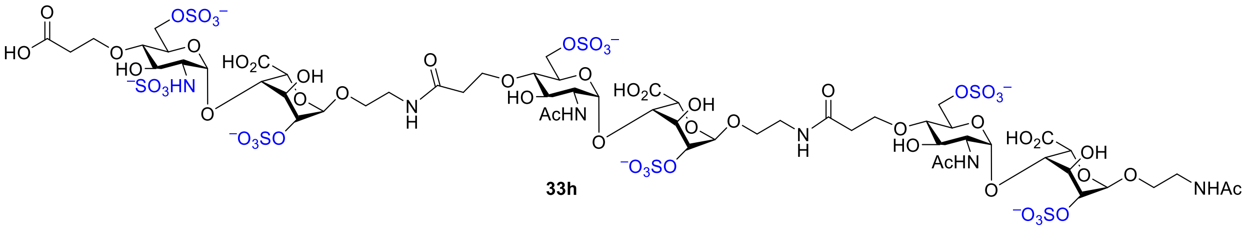

33h

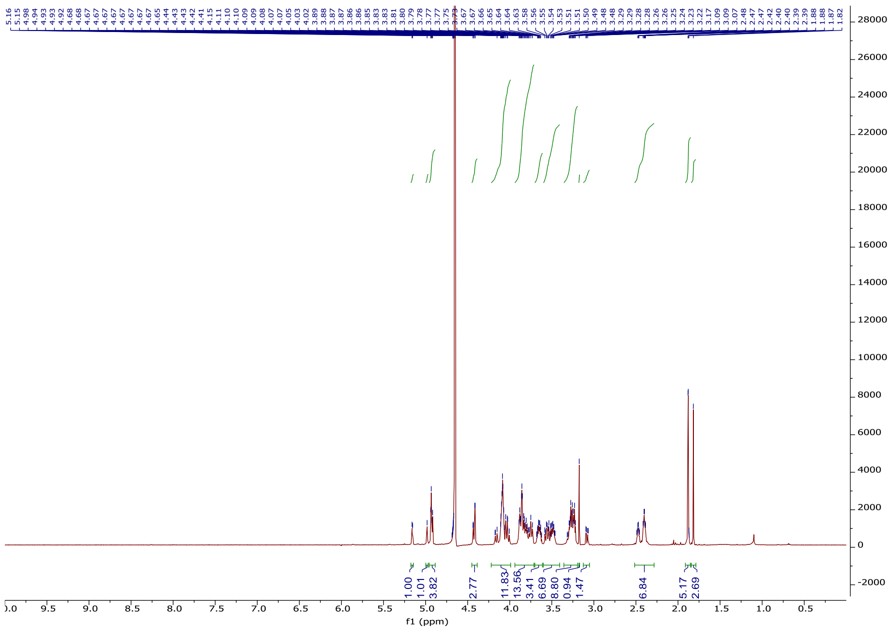

^{1}H-NMR of **33h** (500 MHz D_2O)

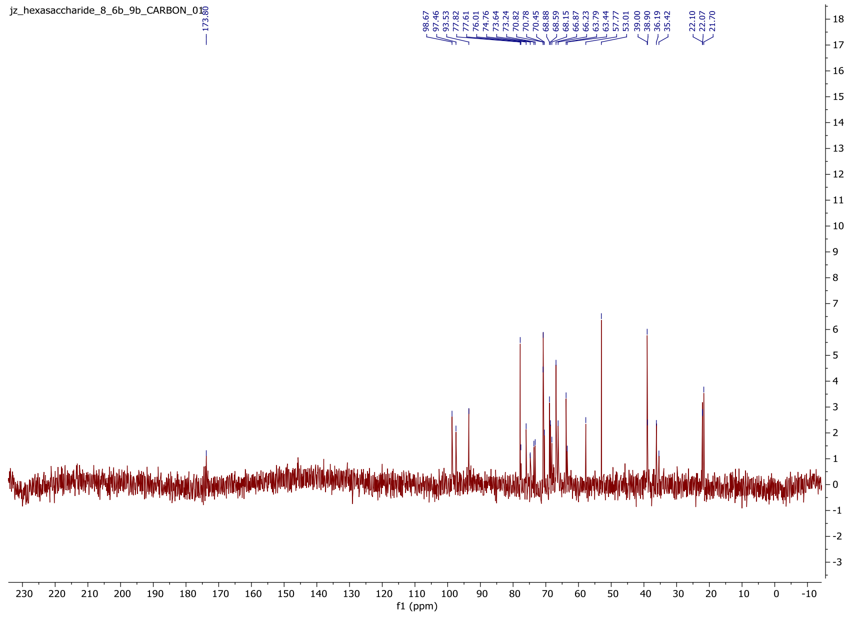

^{13}C-NMR of **33h** (125 MHz D_2O)

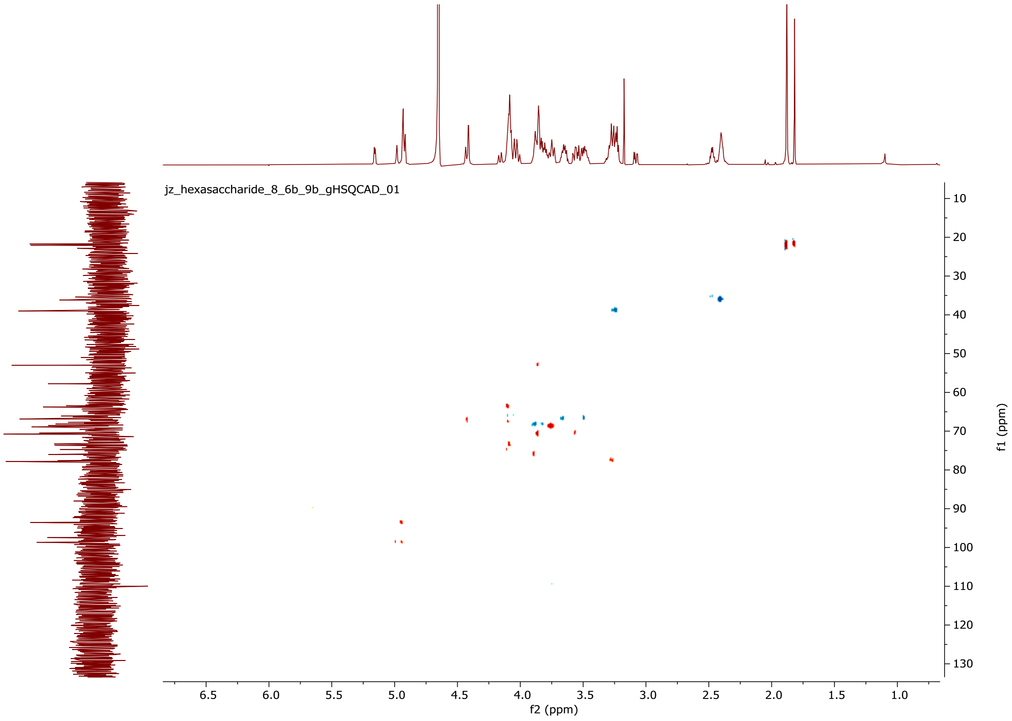

^{1}H-^{13}C gHSQCAD of **33h** (500 MHz D_2O)

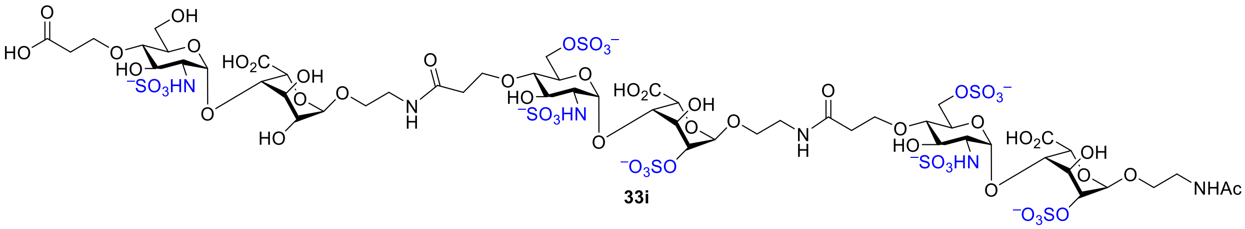

33i

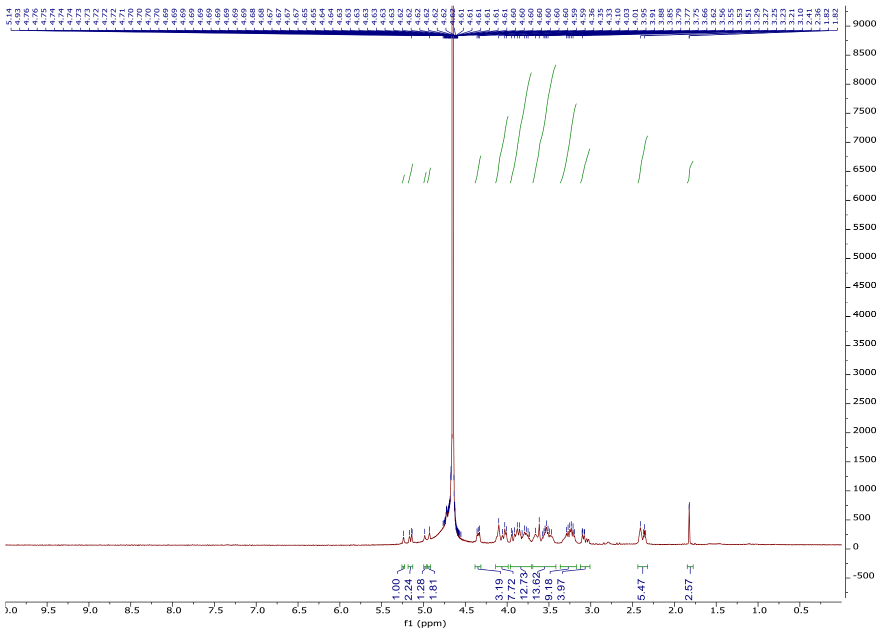

^{1}H-NMR of **33i** (500 MHz D_2O)

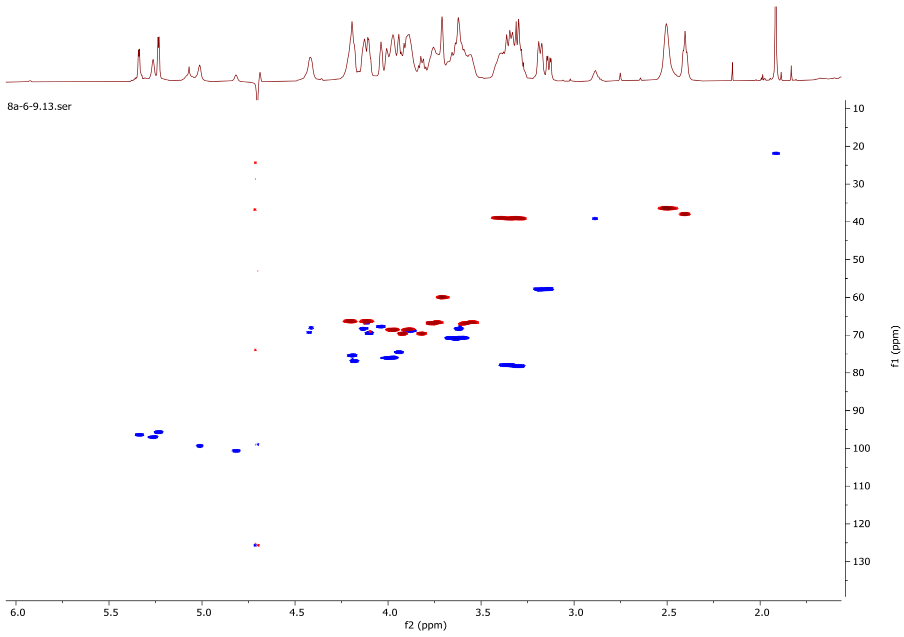

^{1}H-^{13}C gHSQCAD of **33i** (600 MHz D_2O)

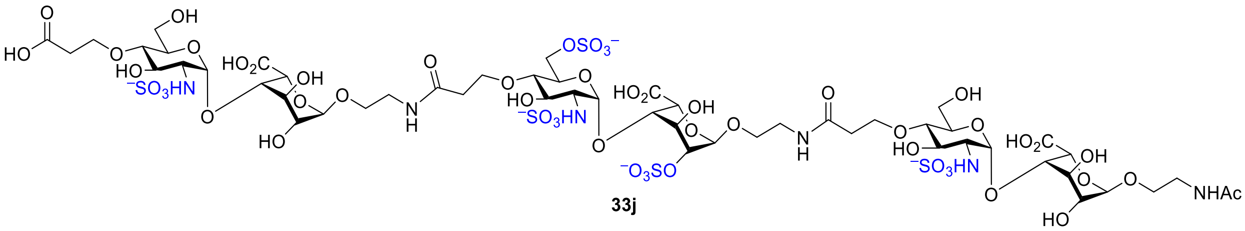

33j

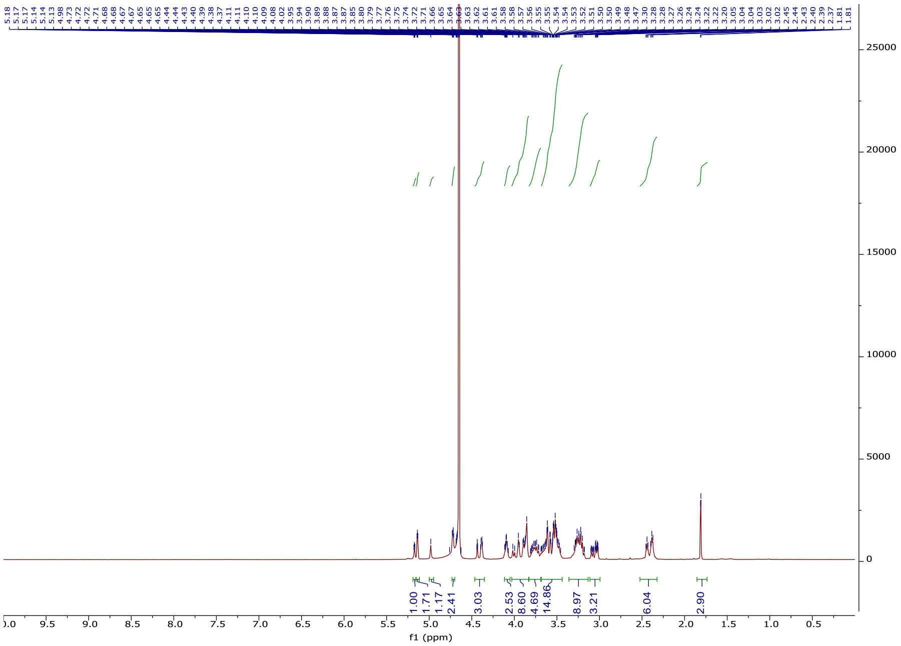

^{1}H-NMR of **33j** (500 MHz D_2O)

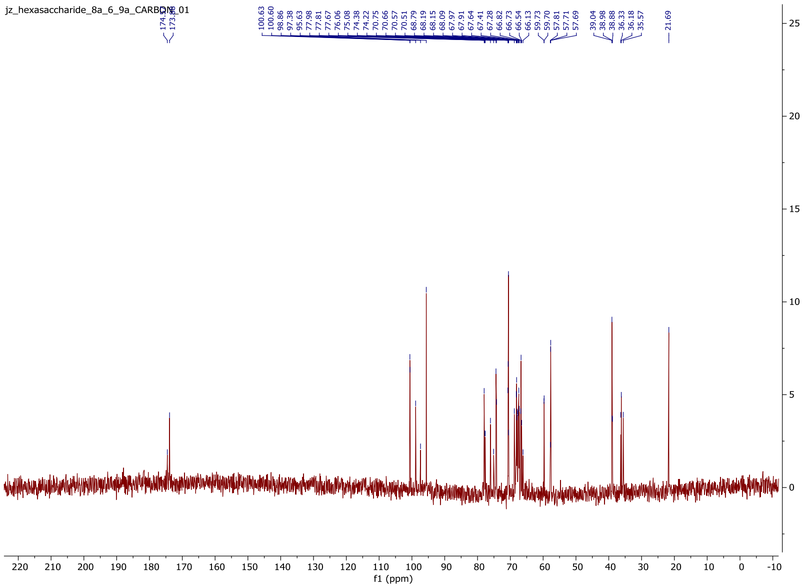

^{13}C-NMR of **33j** (125 MHz D_2O)

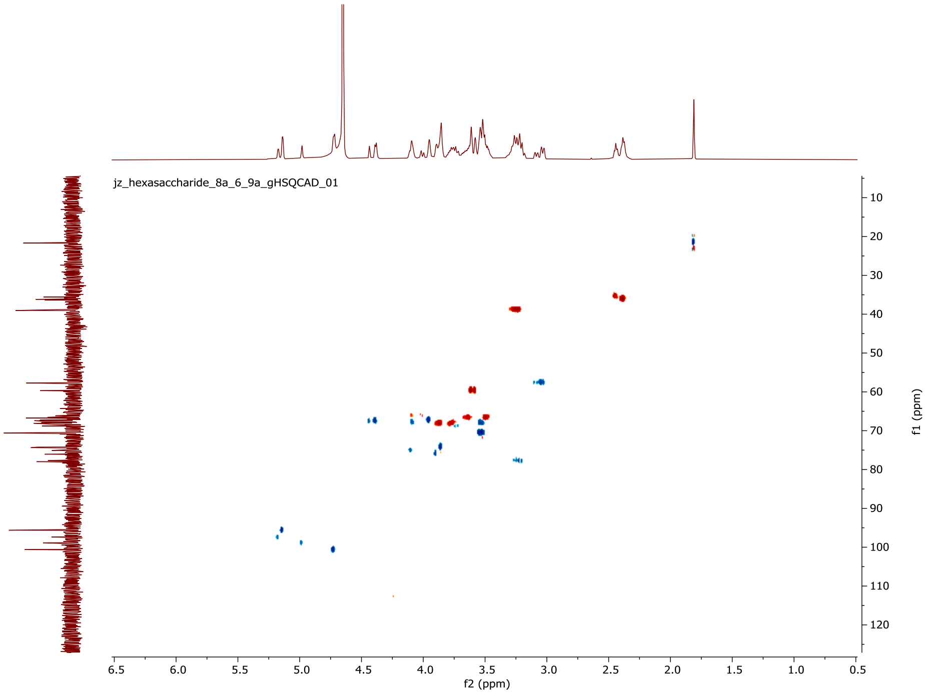

^1H-^{13}C gHSQCAD of **33j** (500 MHz D_2O)

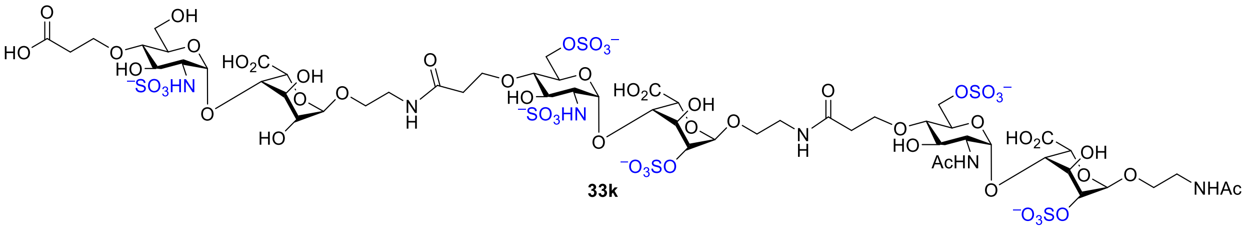

33k

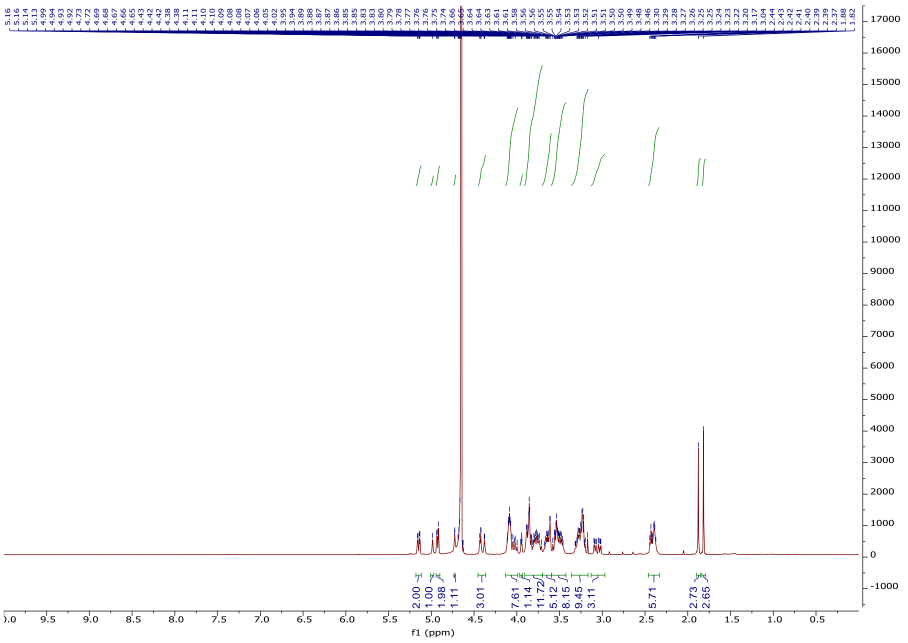

^{1}H-NMR of **33k** (500 MHz D_2O)

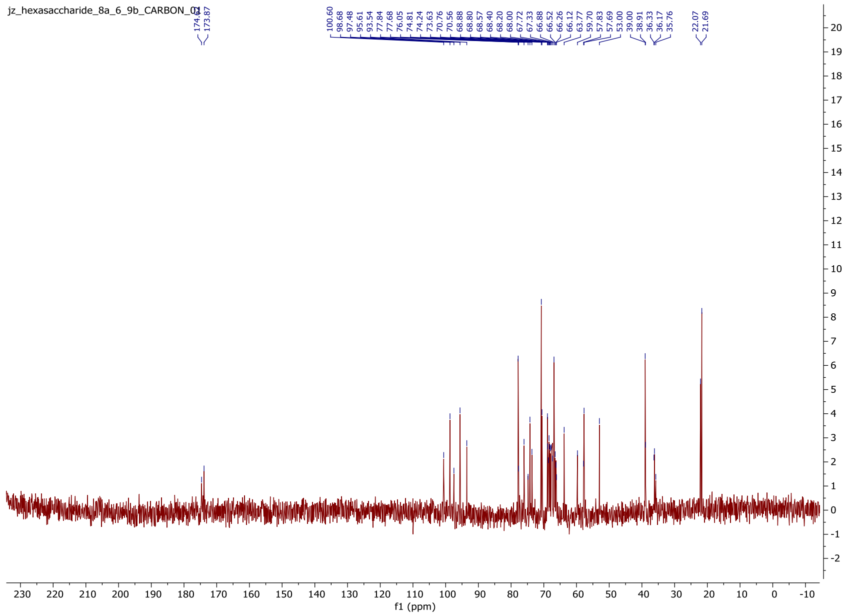

^{13}C-NMR of **33k** (125 MHz D_2O)

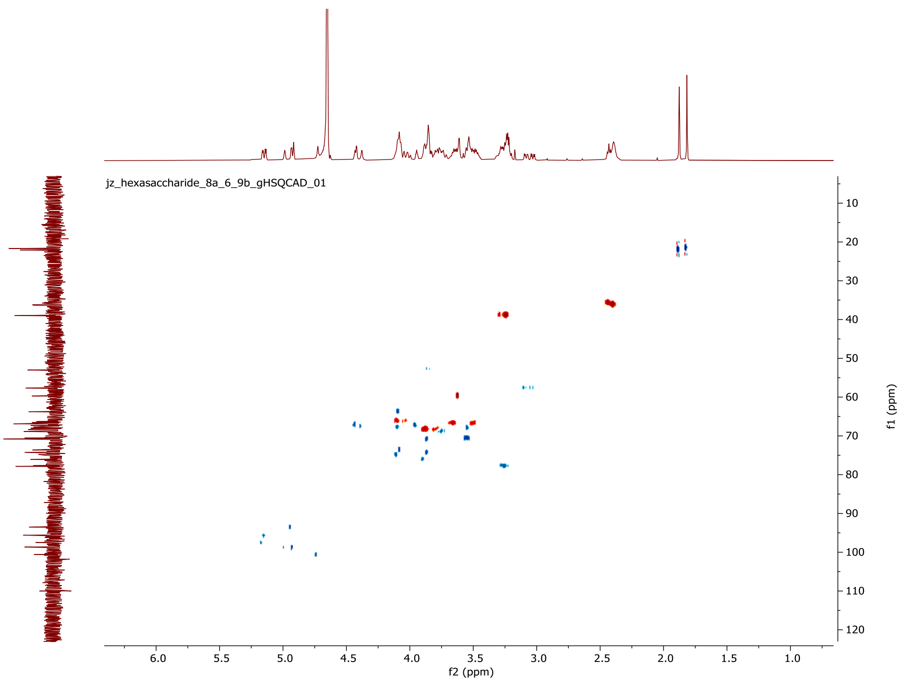

^{1}H-^{13}C gHSQCAD of **33k** (500 MHz D_2O)

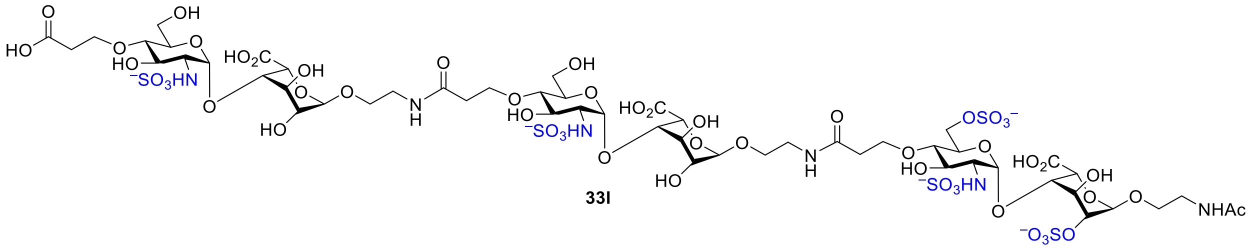

33l

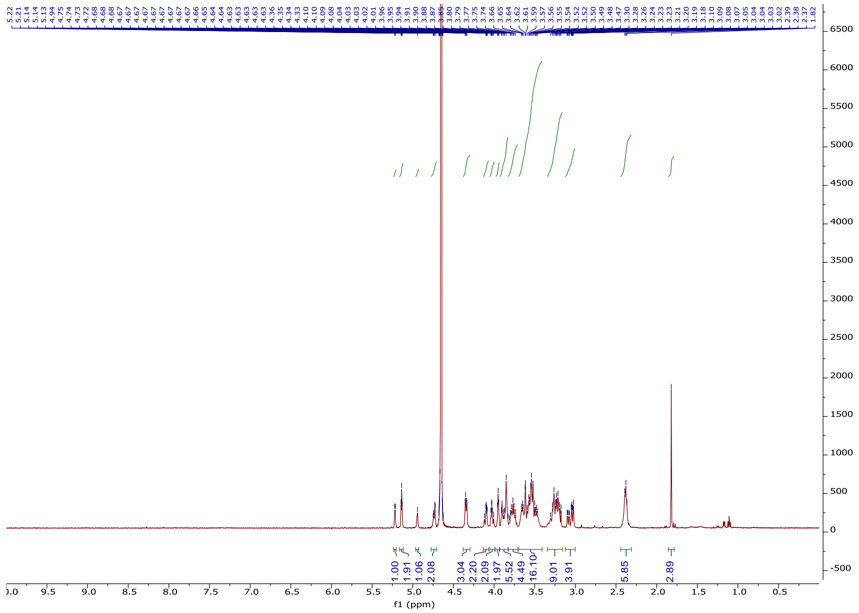

^{1}H-NMR of **33l** (500 MHz D_2O)

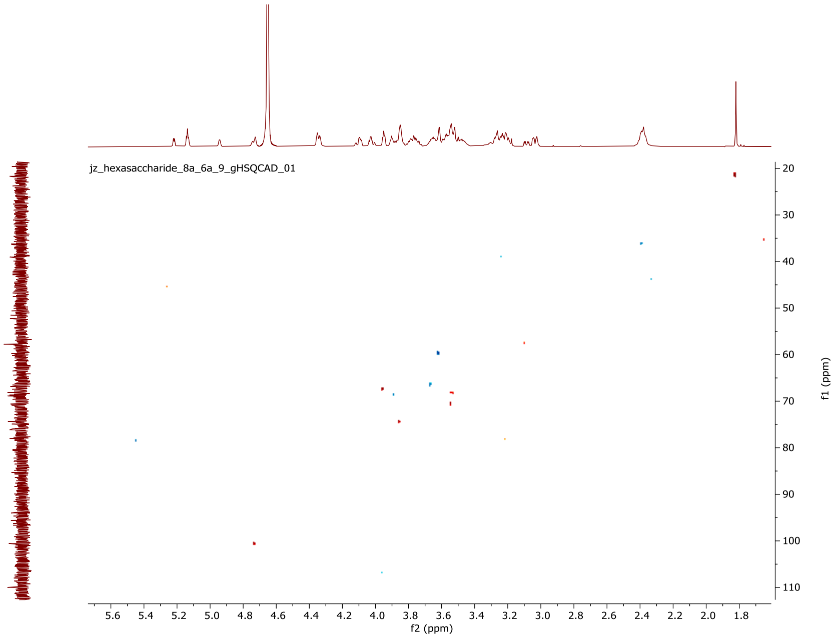

^{1}H-^{1}H gCOSY of **33l** (500 MHz D_2O)

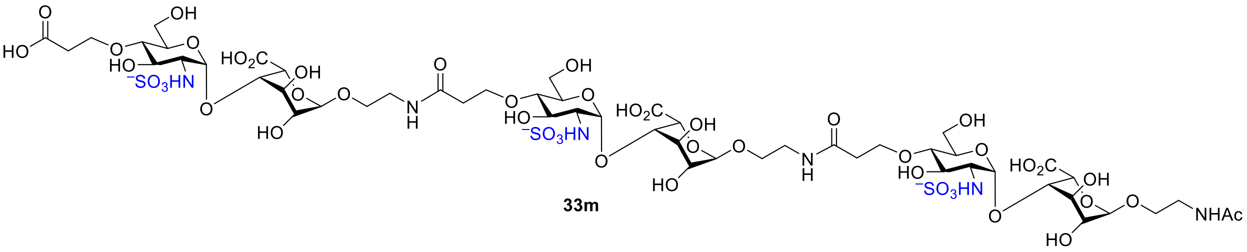

33m

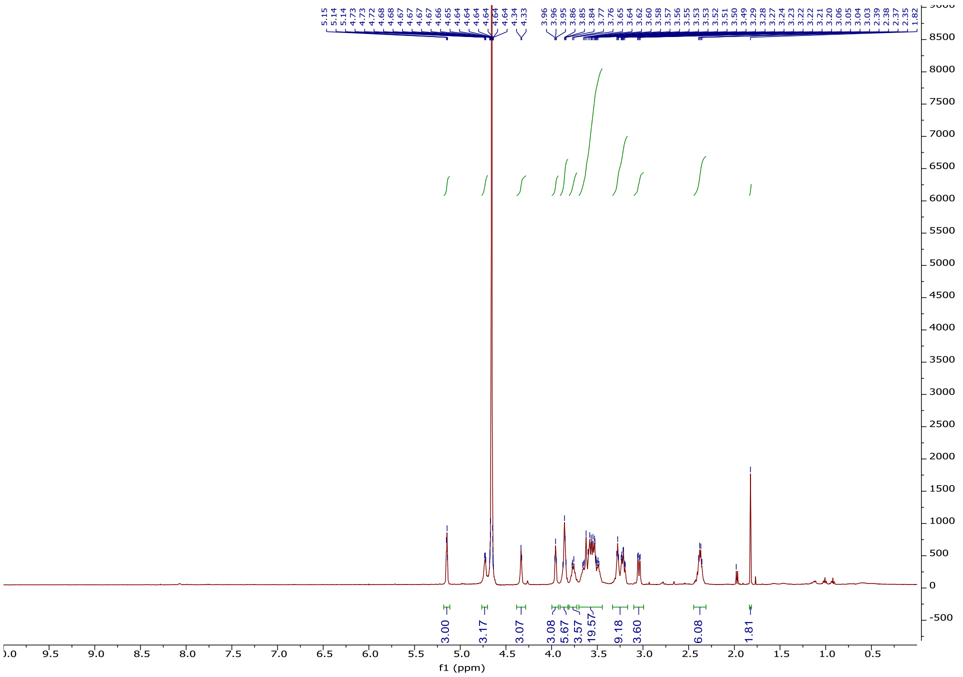

^{1}H-NMR of **33m** (500 MHz D_2O)

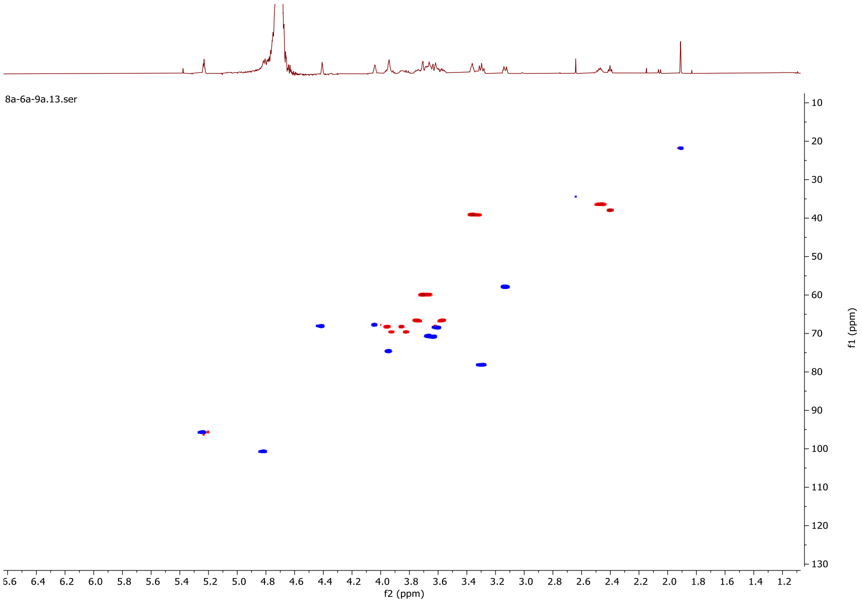

^{1}H-^{13}C gHSQCAD of **33m** (600 MHz D_2O)

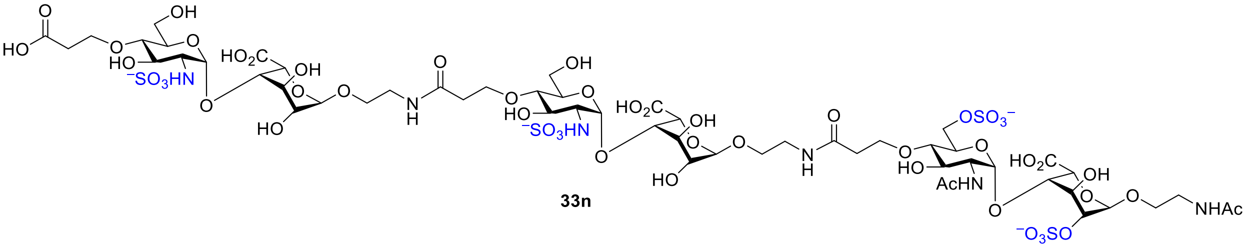

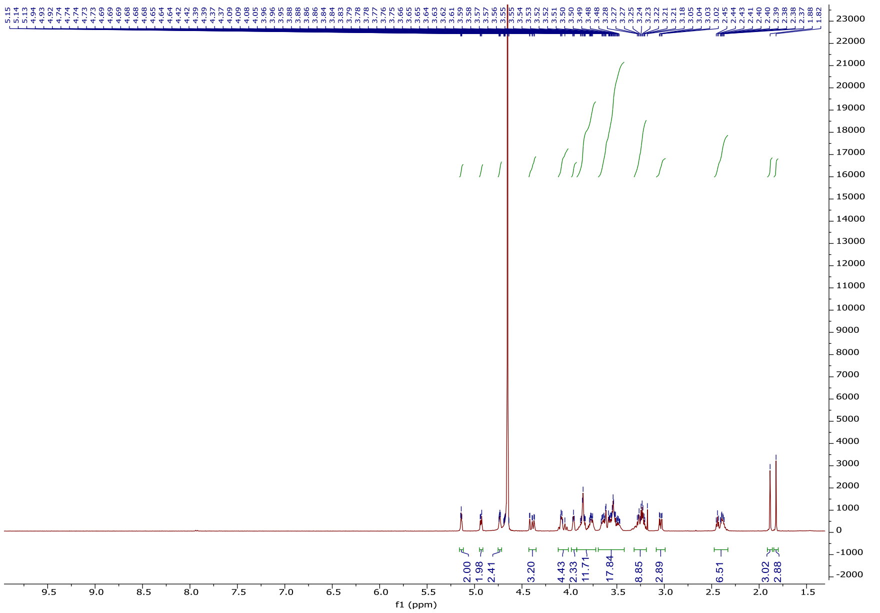

^{1}H-NMR of **33n** (500 MHz D_2O)

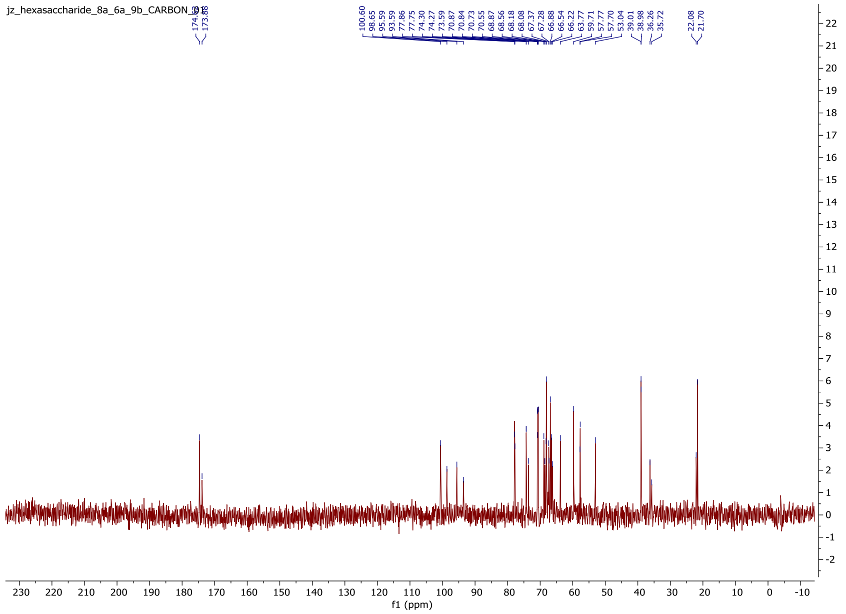

^{13}C-NMR of **33n** (125 MHz D_2O)

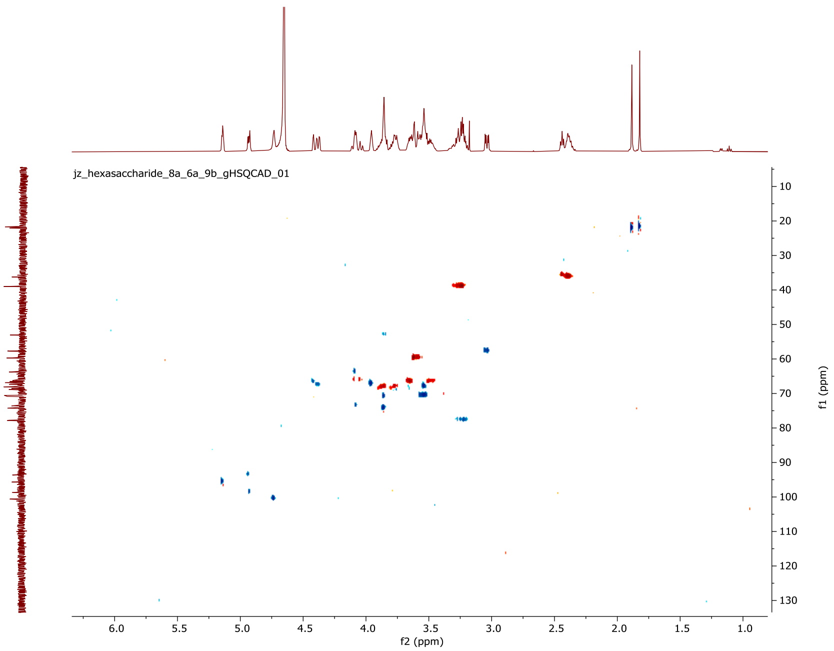

^{1}H-^{13}C gHSQCAD of **33n** (500 MHz D_2O)

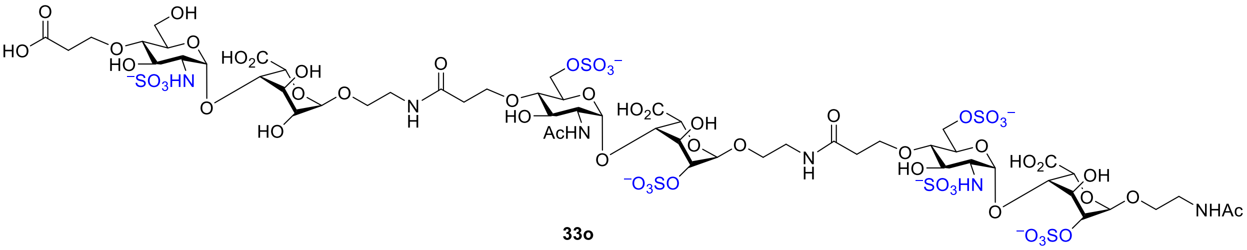

33o

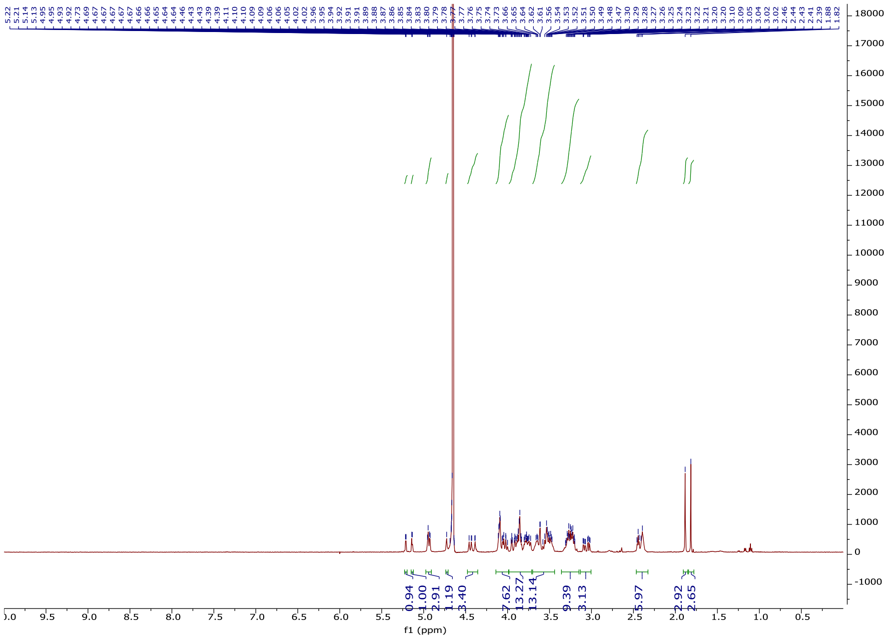

^{1}H-NMR of **33o** (500 MHz D_2O)

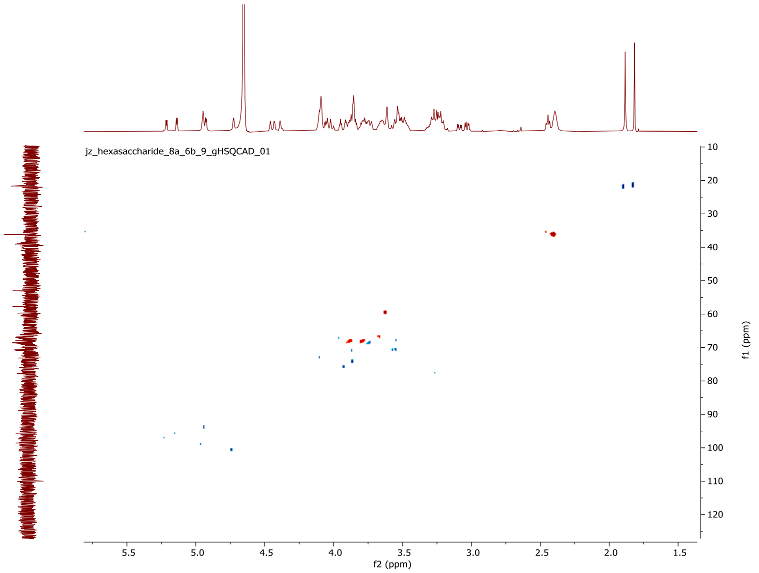

^{1}H-^{13}C gHSQCAD of **33o** (500 MHz D_2O)

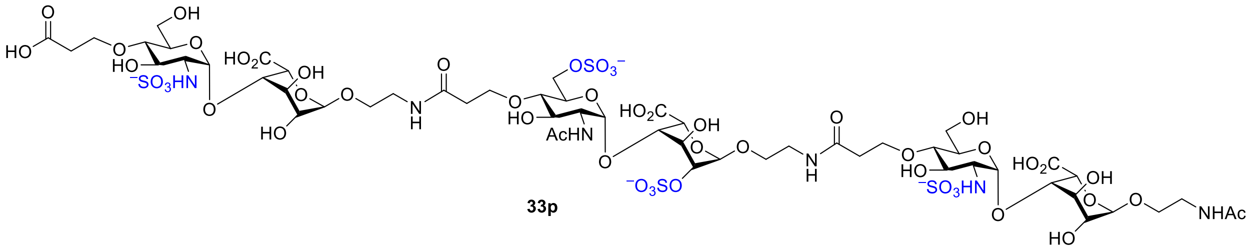

33p

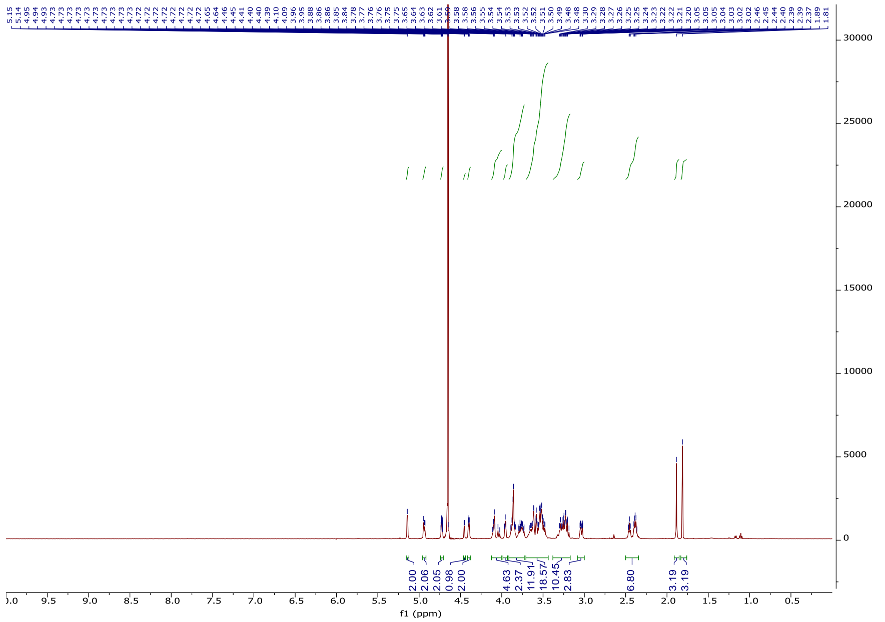

^{1}H-NMR of **33p** (500 MHz D_2O)

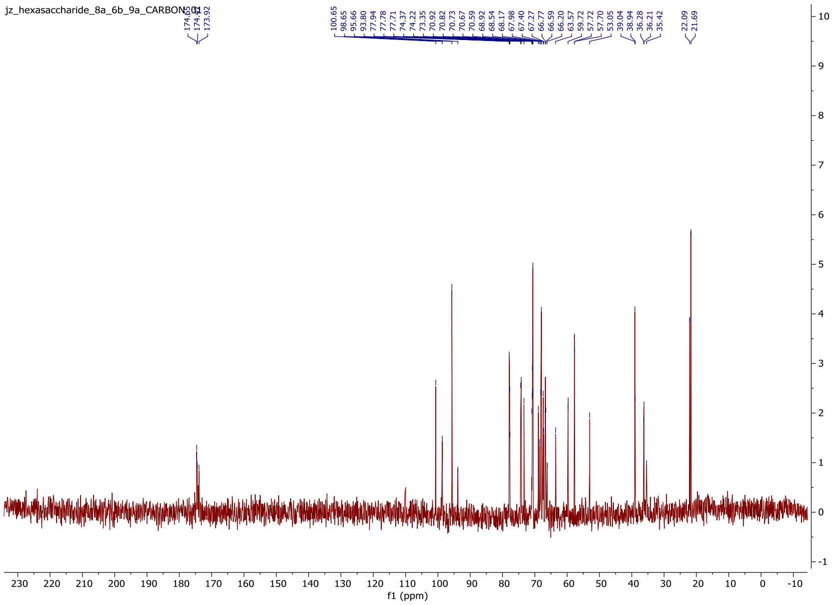

^{13}C-NMR of **33p** (125 MHz D_2O)

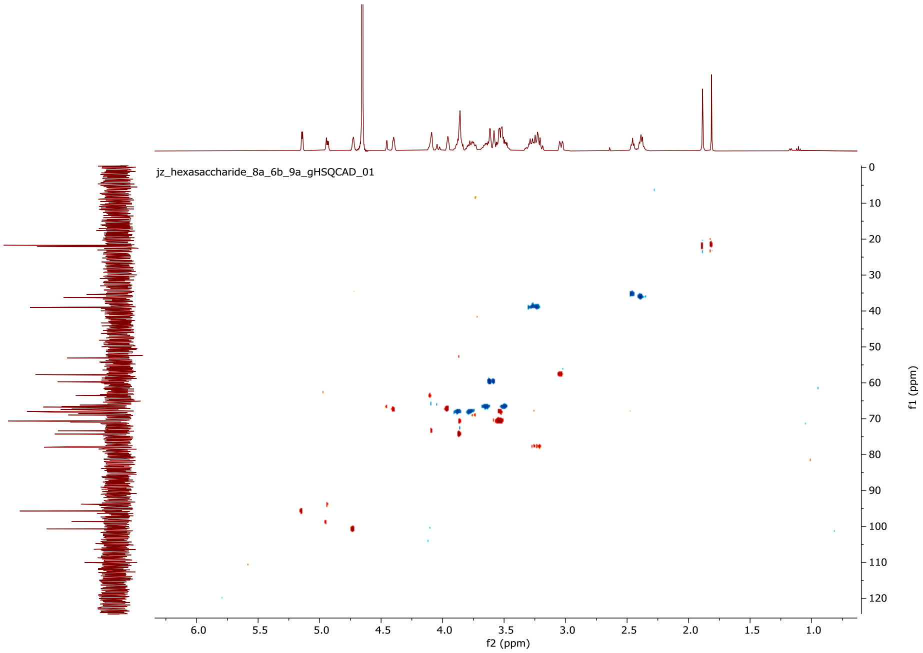

^{1}H-^{13}C gHSQCAD of **33p** (500 MHz D_2O)

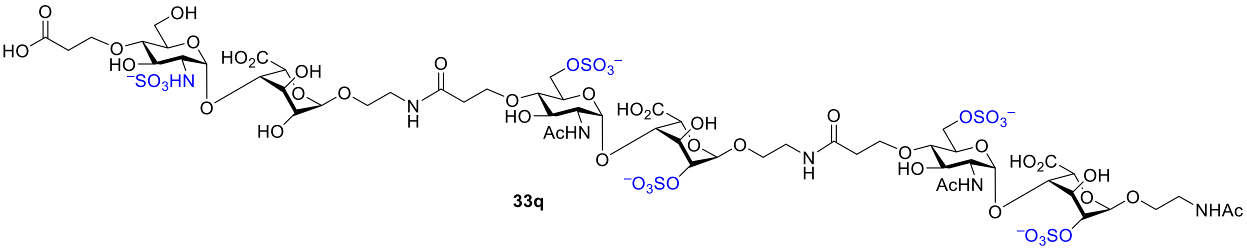

33q

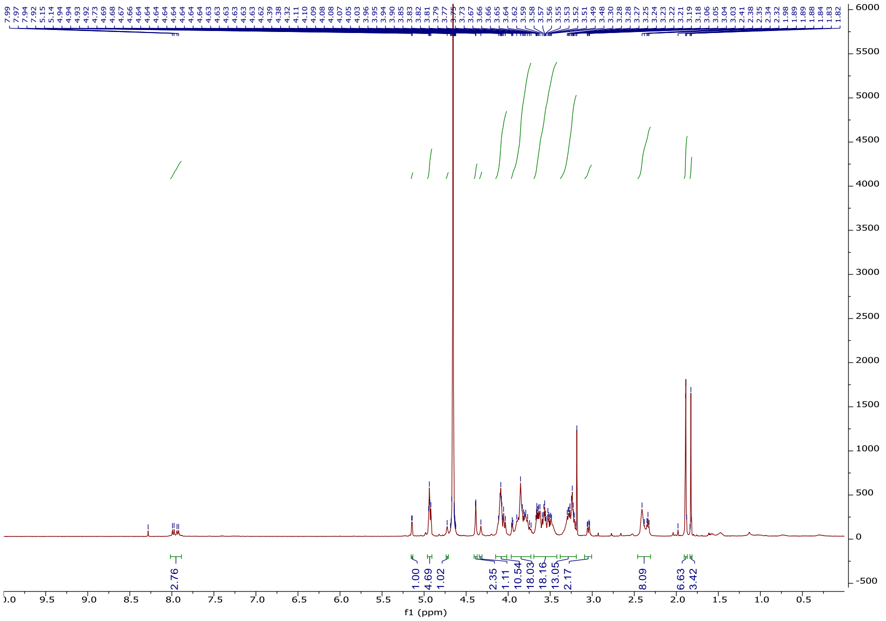

^{1}H-NMR of **33q** (500 MHz D_2O)

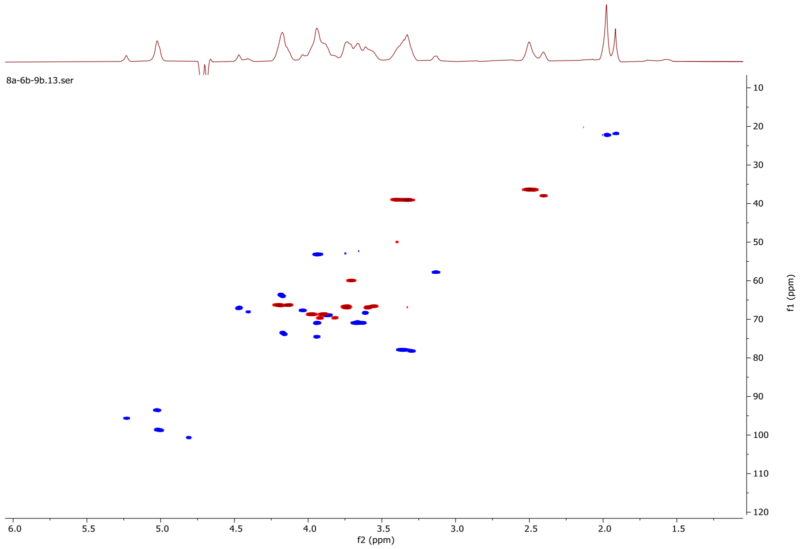

^{1}H-^{13}C gHSQCAD of **33q** (600 MHz D_2O)

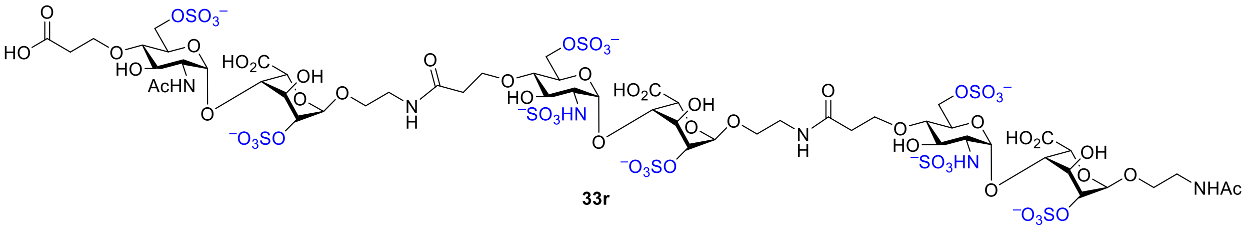

33r

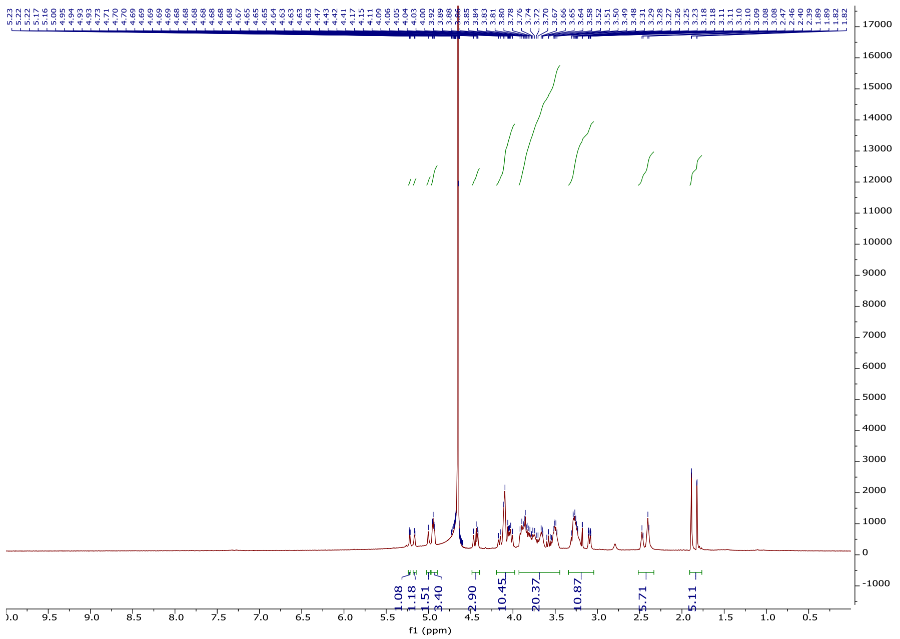

^{1}H-NMR of **33r** (500 MHz D_2O)

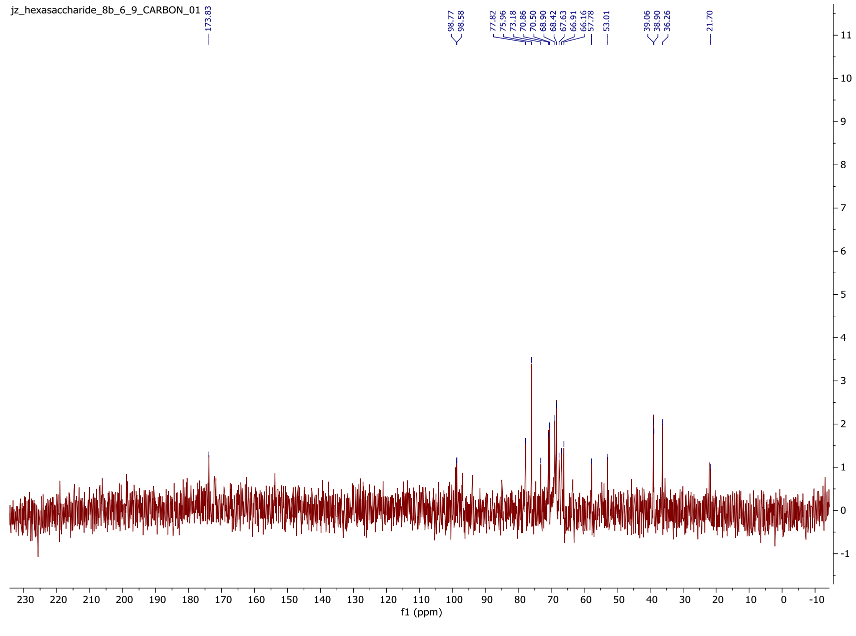

^{13}C-NMR of **33r** (125 MHz D_2O)

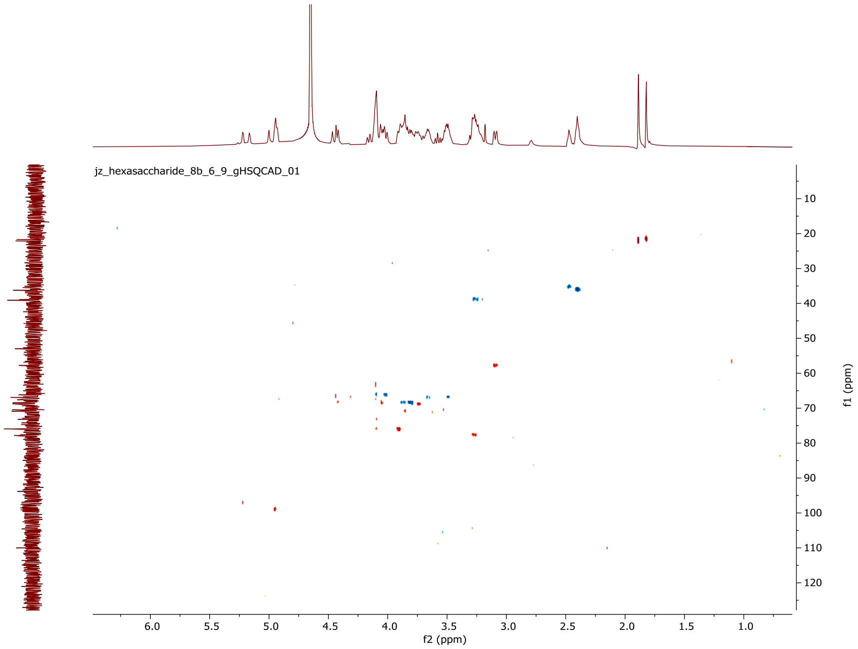

^{1}H-^{13}C gHSQCAD of **33r** (500 MHz D_2O)

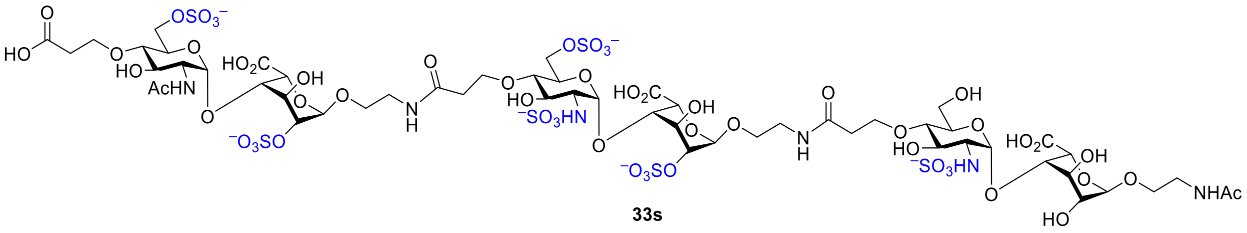

33s

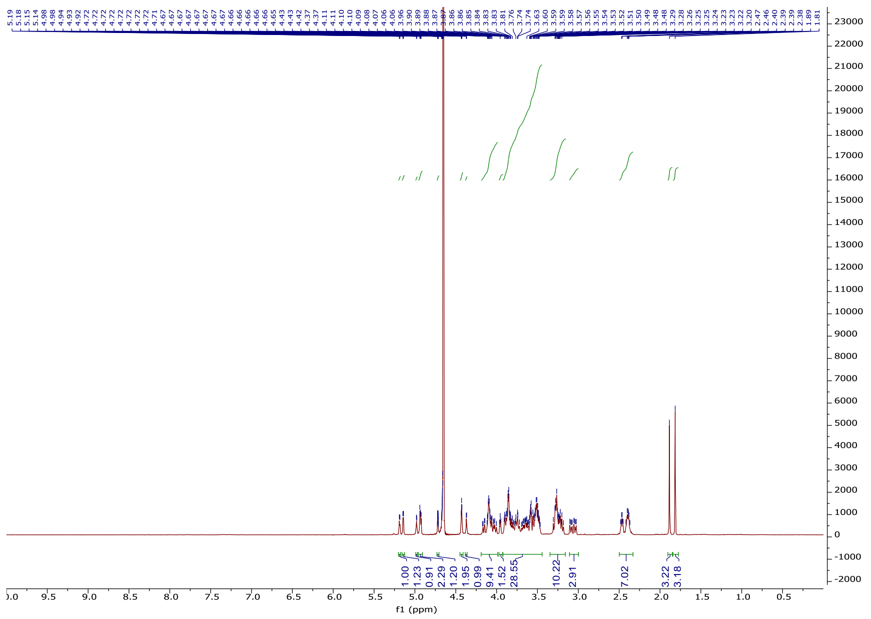

^{1}H-NMR of **33s** (500 MHz D_2O)

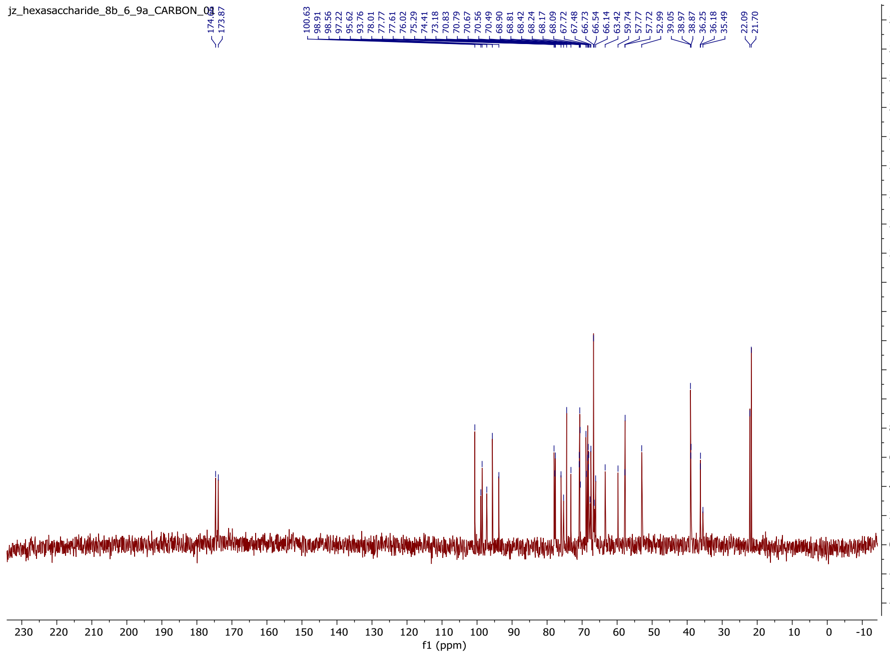

^{13}C-NMR of **33s** (125 MHz D_2O)

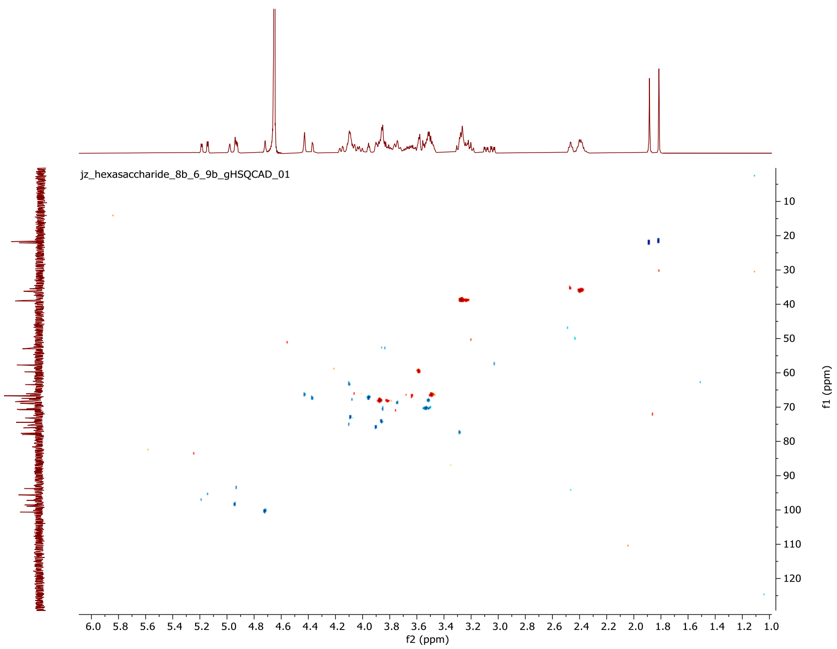

^{1}H-^{13}C gHSQCAD of **33s** (500 MHz D_2O)

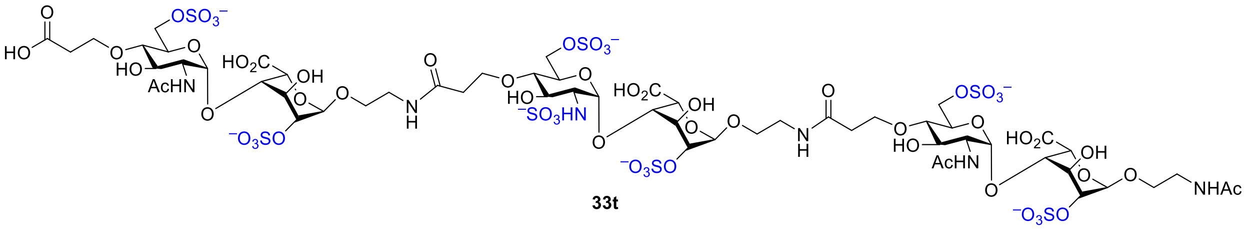

33t

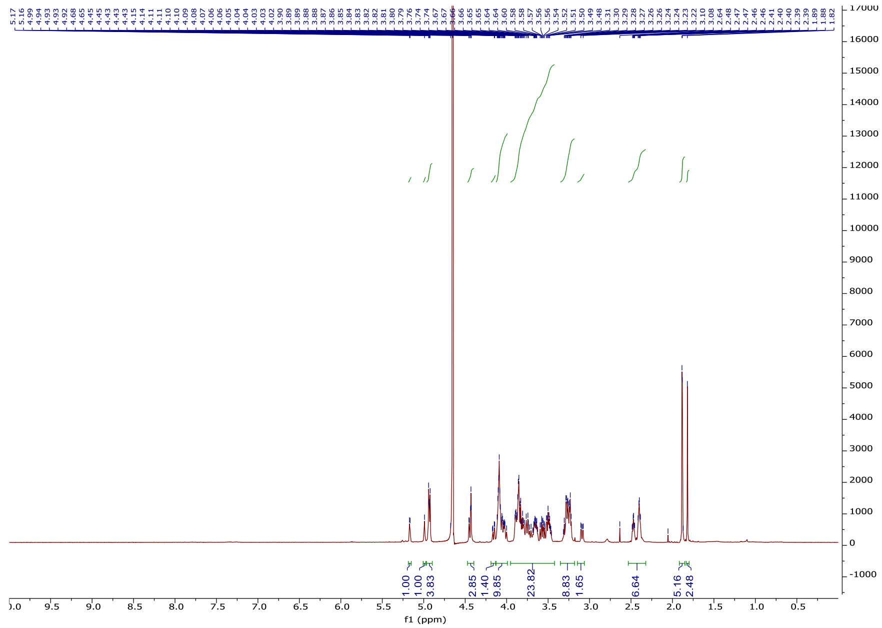

^{1}H-NMR of **33t** (500 MHz D_2O)

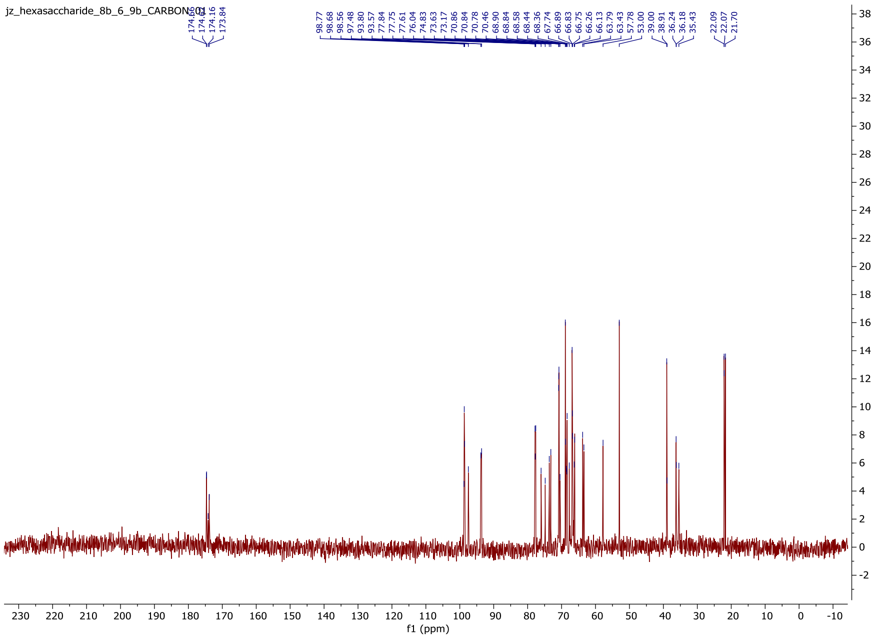

^{13}C-NMR of **33t** (125 MHz D_2O)

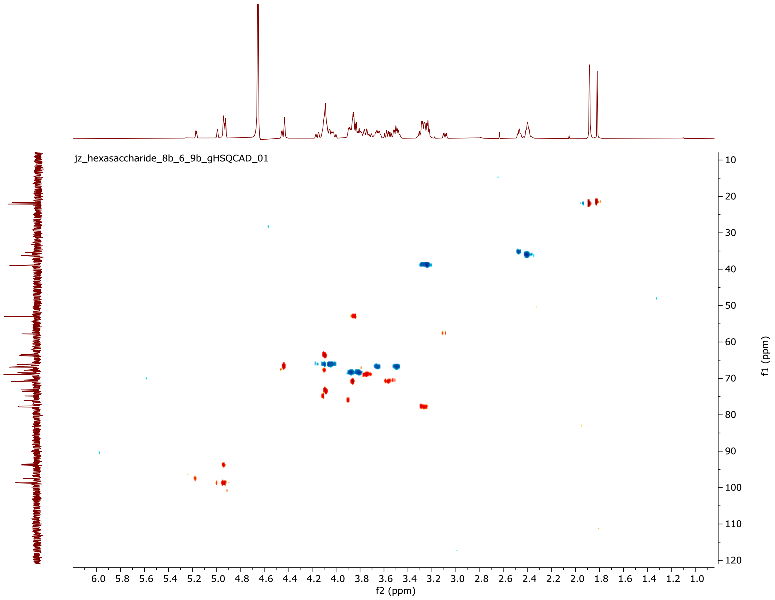

^{1}H-^{13}C gHSQCAD of **33t** (500 MHz D_2O)

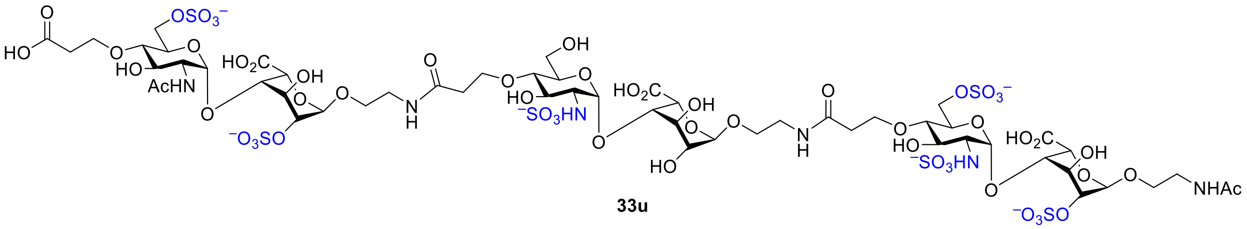

33u

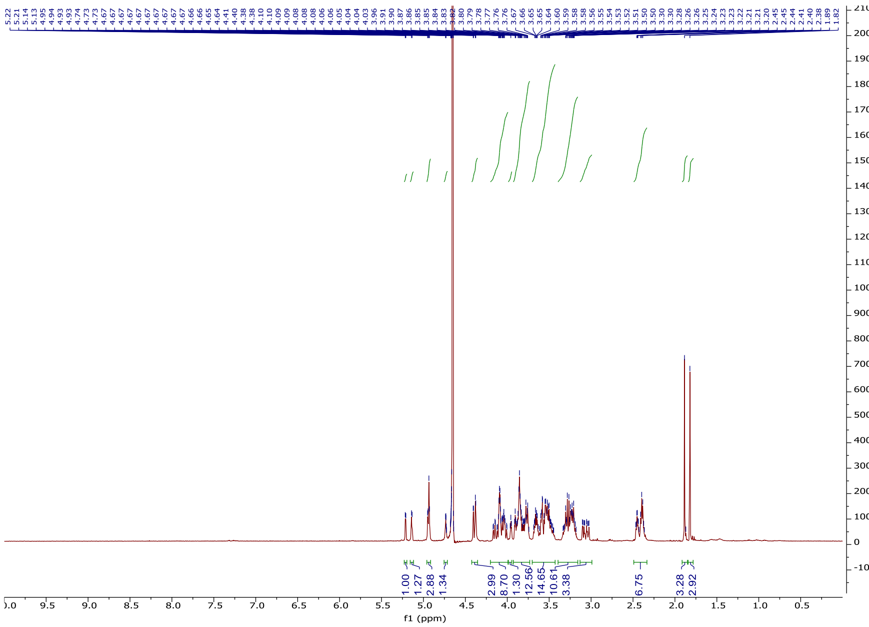

^{1}H-NMR of **33u** (500 MHz D_2O)

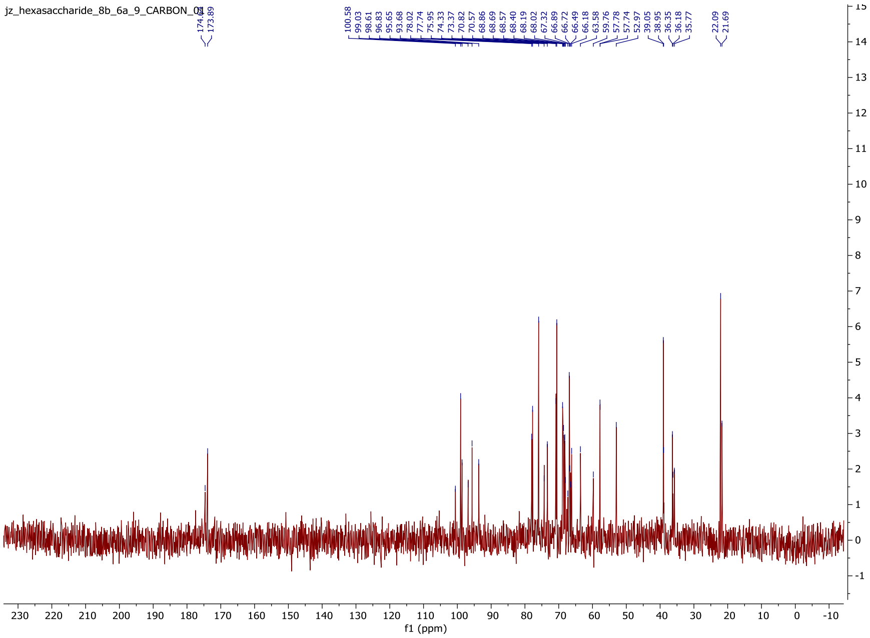

^{13}C-NMR of **33u** (125 MHz D_2O)

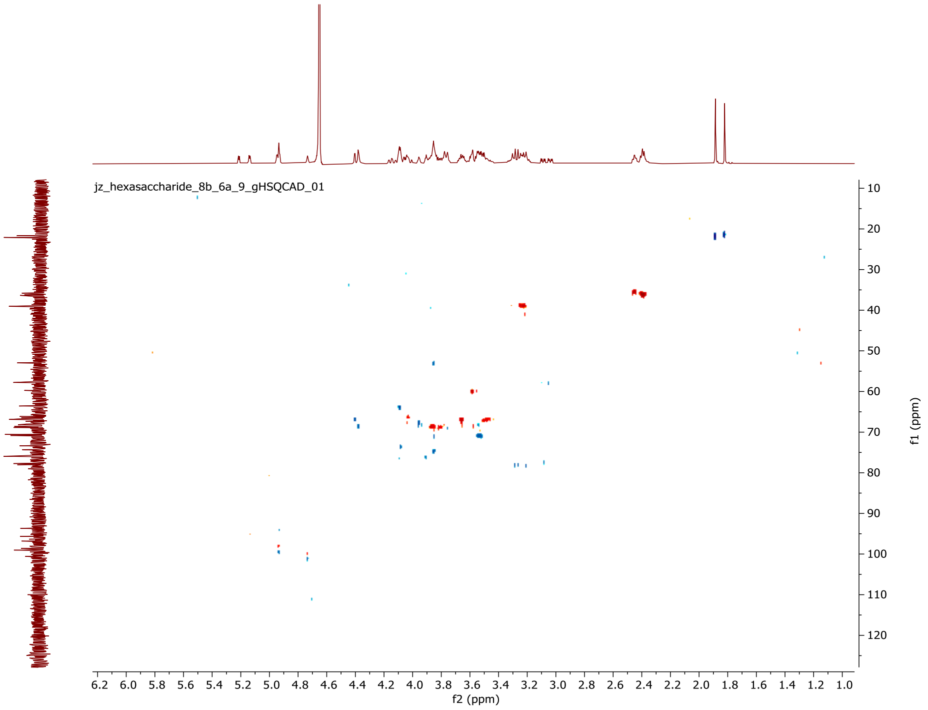

^{1}H-^{13}C gHSQCAD of **33u** (500 MHz D_2O)

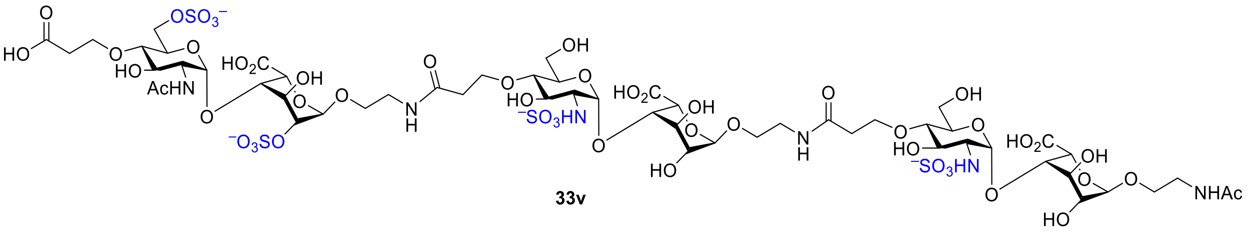

33v

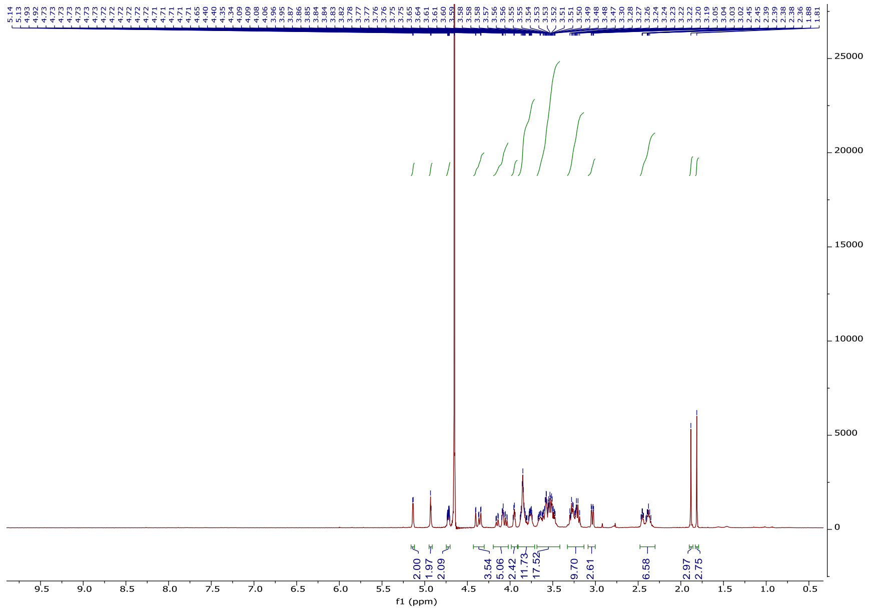

^{1}H-NMR of **33v** (500 MHz D_2O)

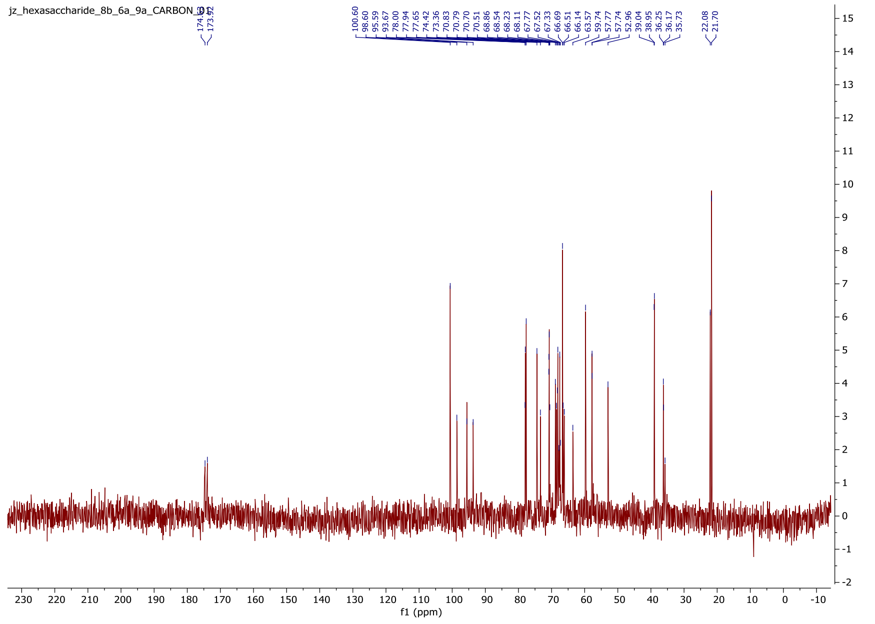

^{13}C-NMR of **33v** (125 MHz D_2O)

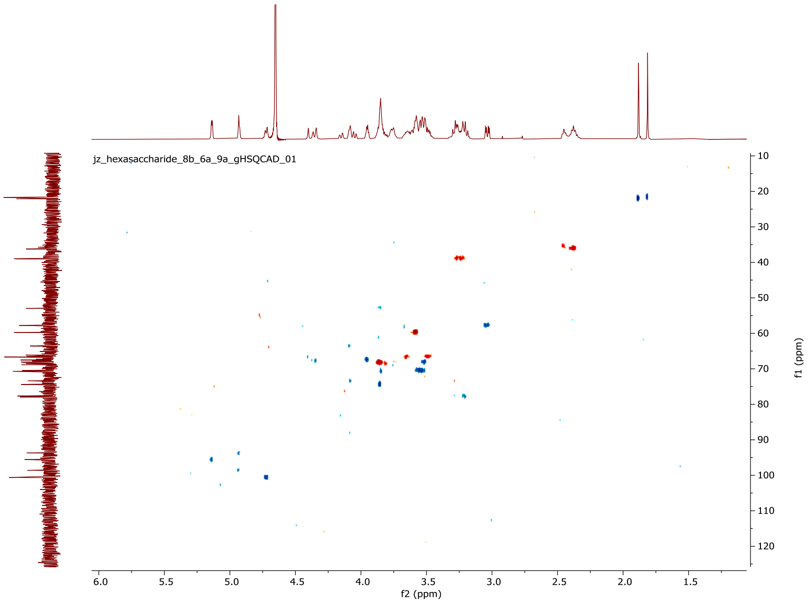

^{1}H-^{13}C gHSQCAD of **33v** (500 MHz D_2O)

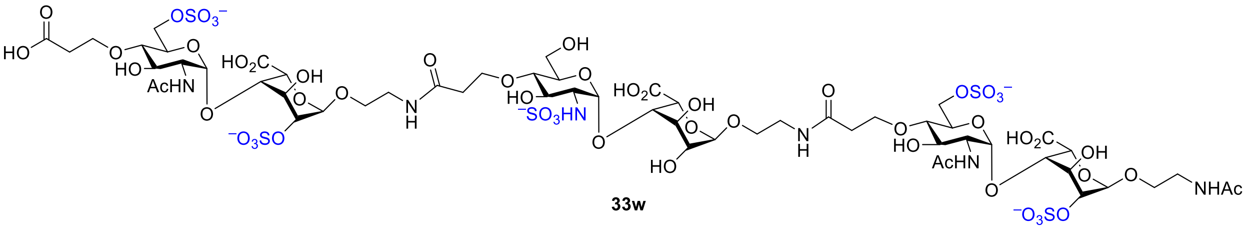

33w

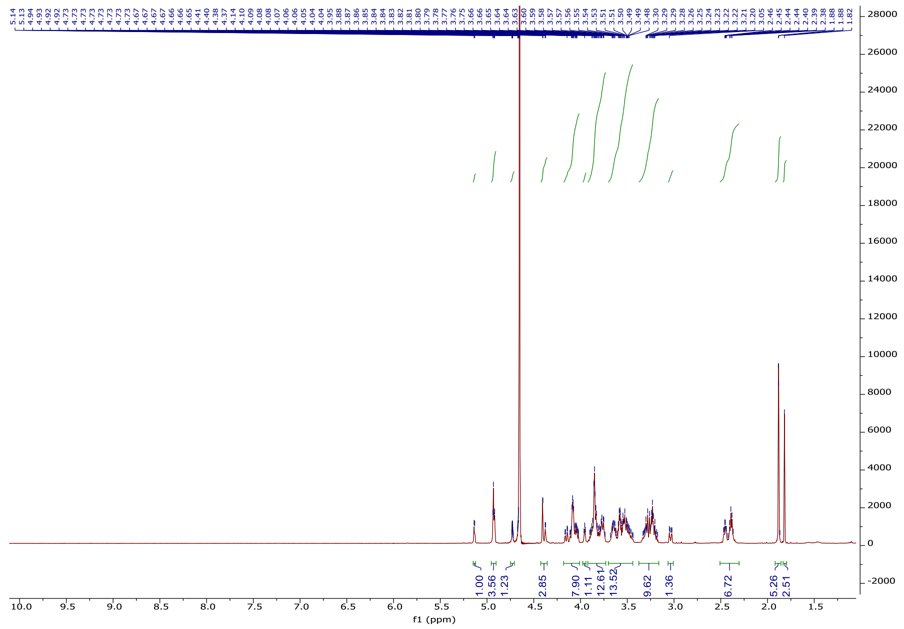

^{1}H-NMR of **33w** (500 MHz D_2O)

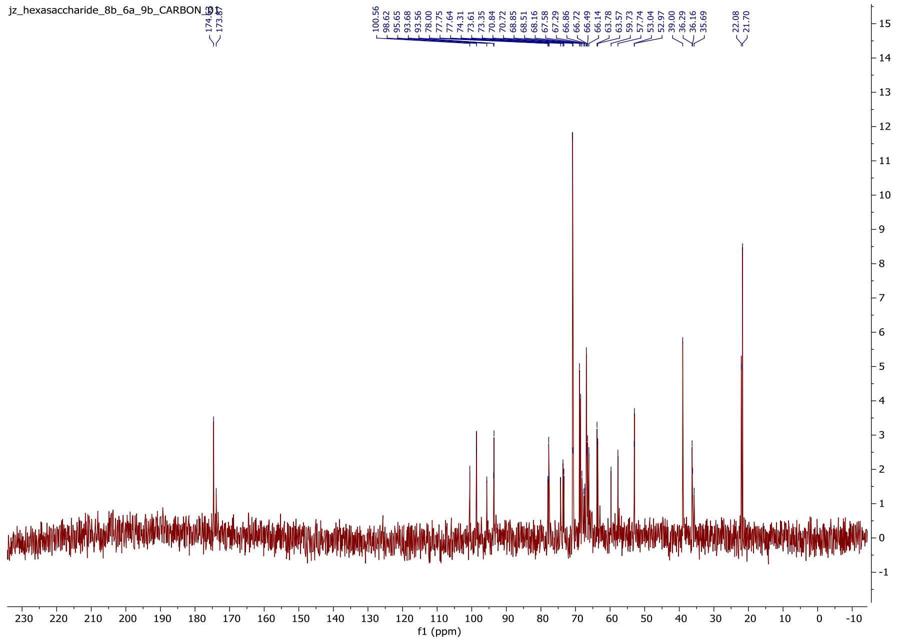

^{13}C-NMR of **33w** (125 MHz D_2O)

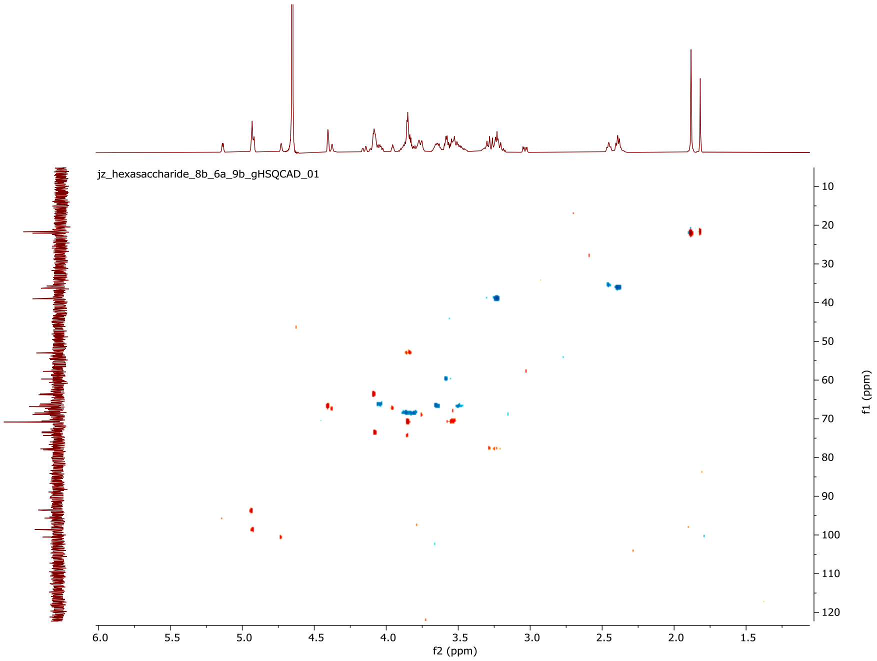

^{1}H-^{13}C gHSQCAD of **33w** (500 MHz D_2O)

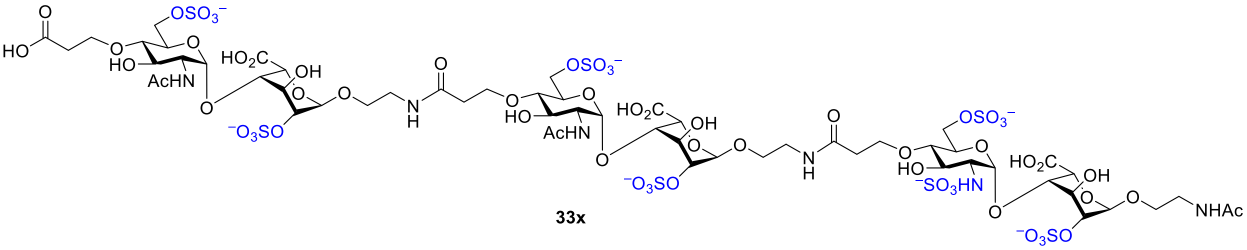

33x

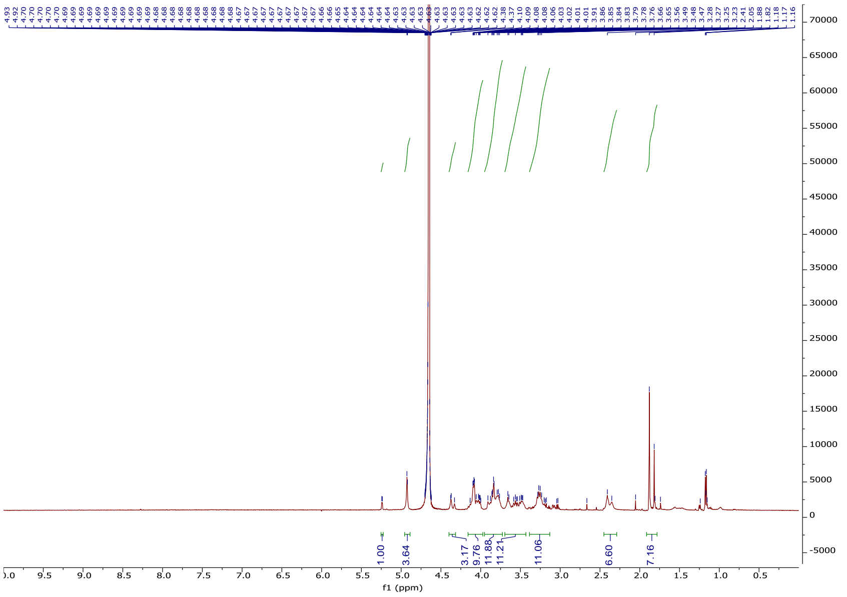

^{1}H-NMR of **33x** (600 MHz D_2O)

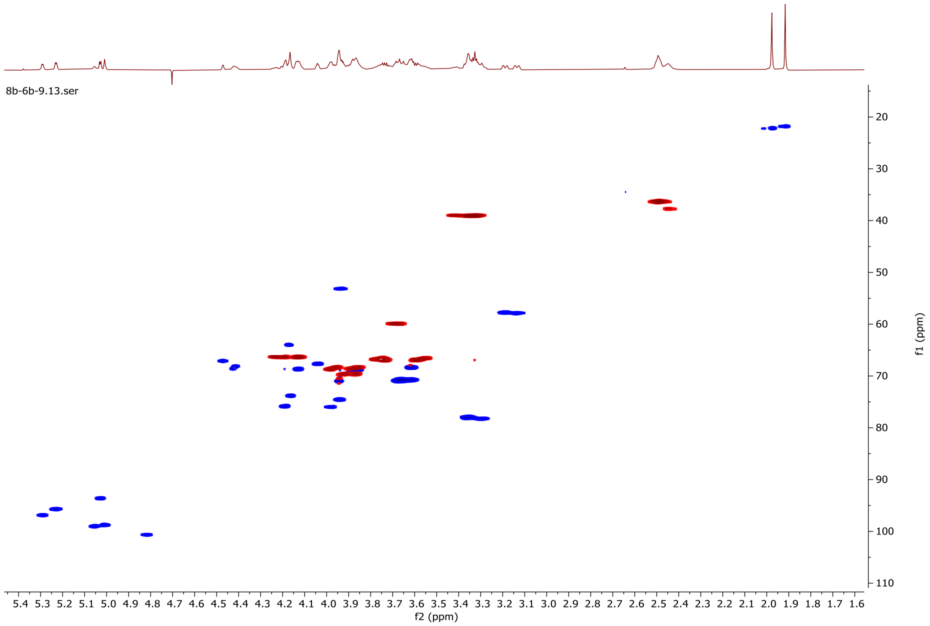

^{1}H-^{13}C gHSQCAD of **33x** (600 MHz D_2O)

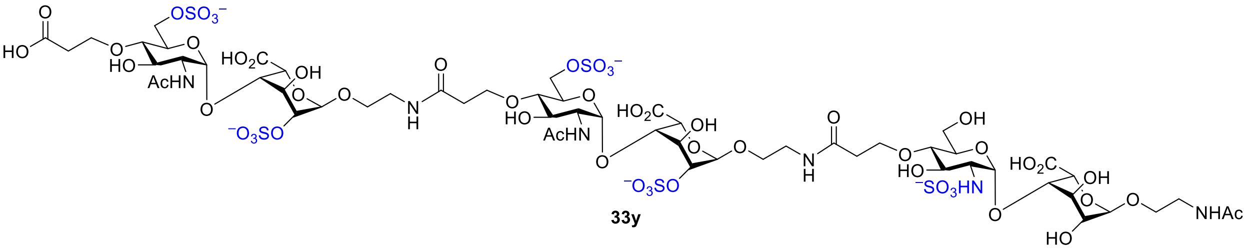

33y

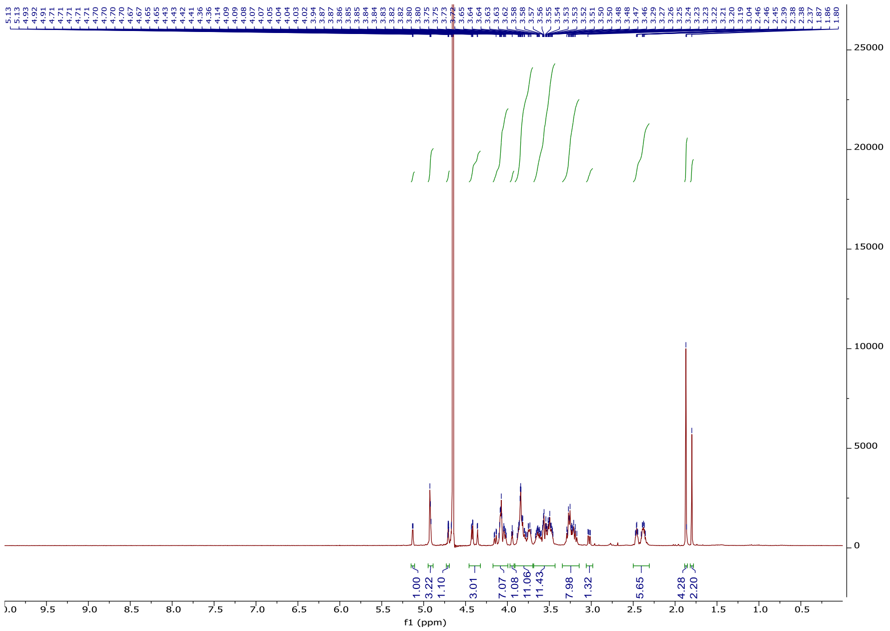

^{1}H-NMR of **33y** (500 MHz D_2O)

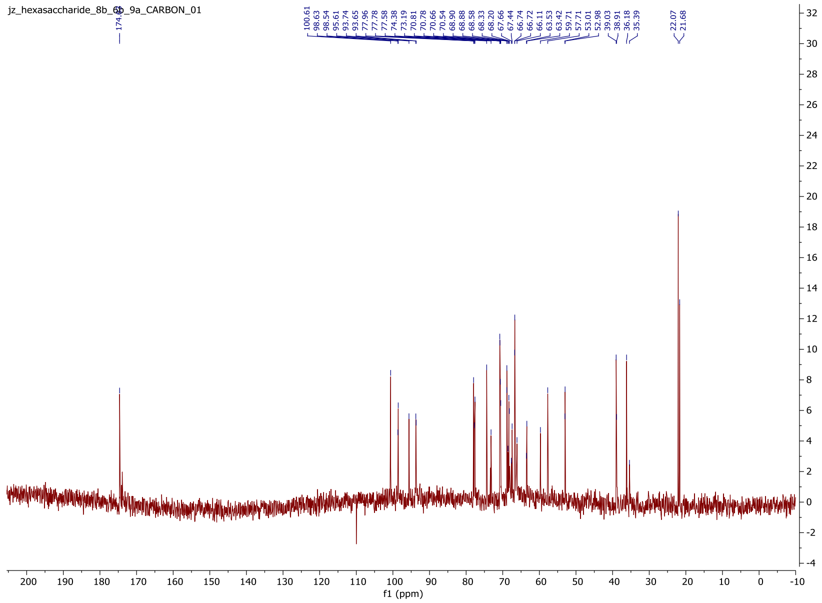

^{13}C-NMR of **33y** (125 MHz D_2O)

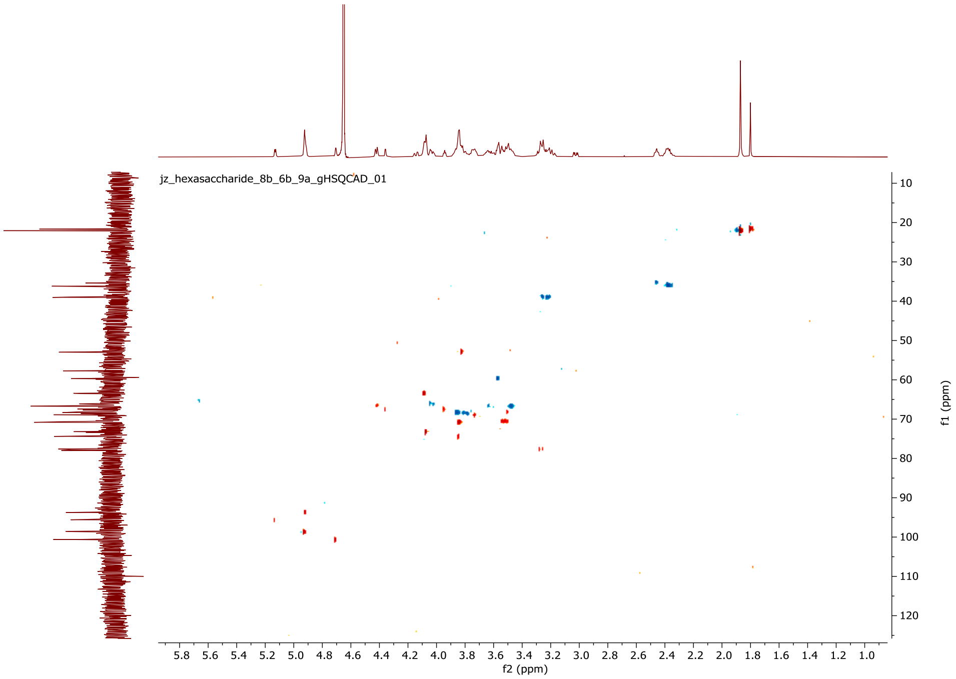

^{1}H-^{13}C gHSQCAD of **33y** (500 MHz D_2O)

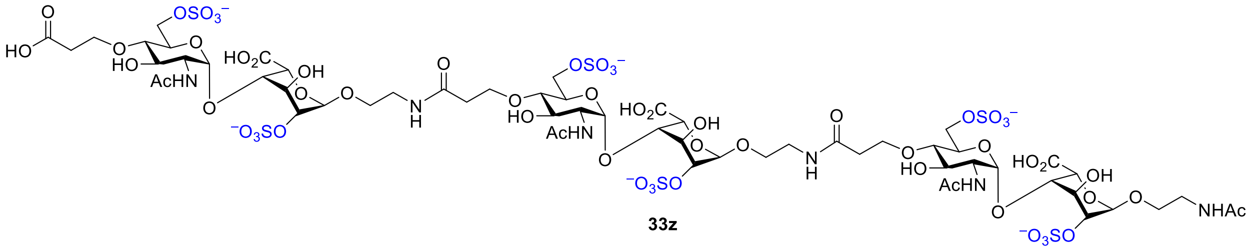

33z

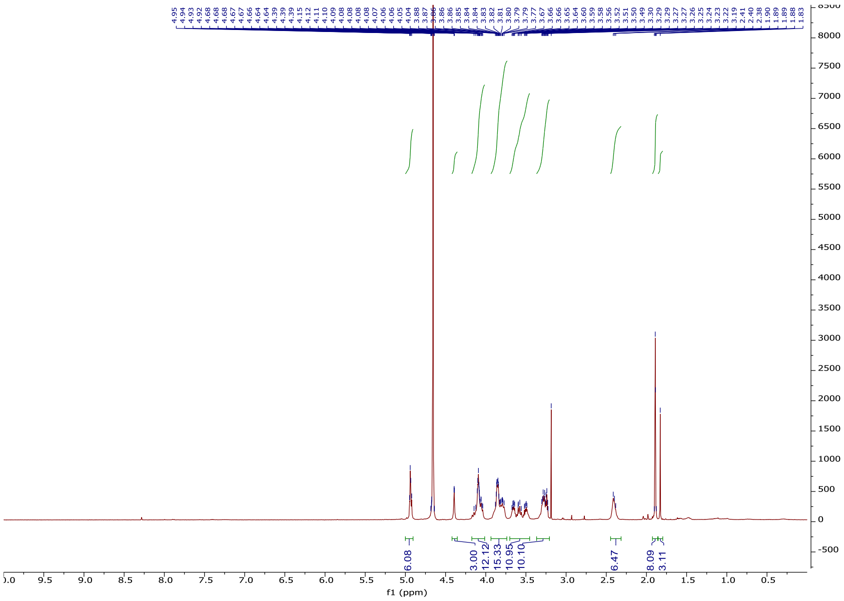

^{1}H-NMR of **33z** (500 MHz D_2O)

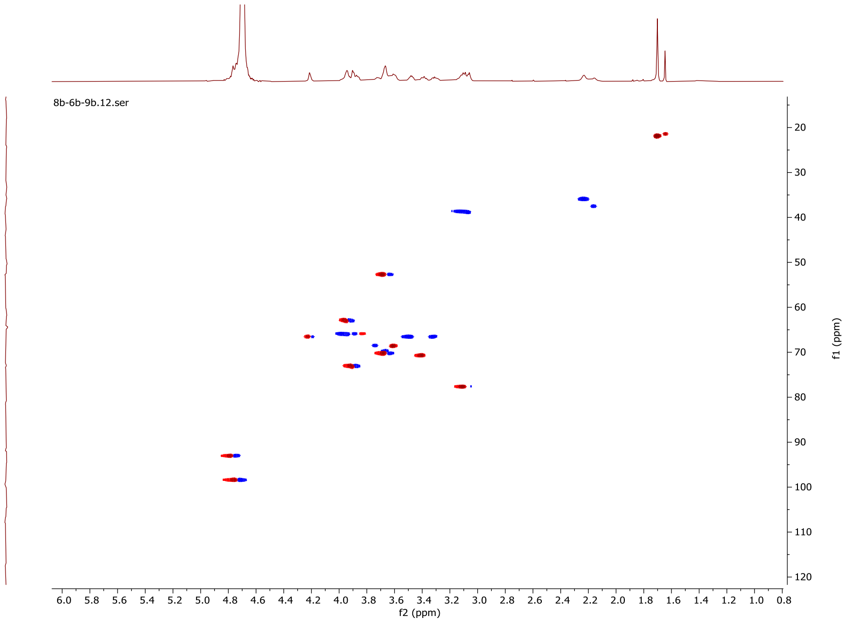

^{1}H-^{13}C gHSQCAD of **33z** (600 MHz D_2O)
